# Synthesis of fluorine-containing bicyclo[4.1.1]octenes *via* photocatalyzed defluorinative (4 + 3) annulation of bicyclo[1.1.0]butanes with *gem*-difluoroalkenes†

**DOI:** 10.1039/d4sc07243j

**Published:** 2024-12-13

**Authors:** Kuan Zhang, Zhengyang Gao, Yan Xia, Pengfei Li, Pin Gao, Xin-Hua Duan, Li-Na Guo

**Affiliations:** a Department of Chemistry, School of Chemistry, Xi'an Key Laboratory of Sustainable Energy Material Chemistry, Engineering Research Center of Energy Storage Materials and Devices, Ministry of Education, Xi'an Jiaotong University Xi'an 710049 China guoln81@xjtu.edu.cn; b Frontier Institute of Science and Technology, State Key Laboratory for Mechanical Behavior of Materials, Xi'an Jiaotong University Xi'an 710049 China

## Abstract

Although bicyclo[4.1.1] systems are privileged scaffolds in many natural products and drug molecules, efficient synthetic approaches to these systems remain underdeveloped. In this work, we disclose a photoredox-catalyzed defluorinative (4 + 3) annulation of bicyclo[1.1.0]butanes with *gem*-difluoroalkenes, which provides practical and straightforward access to the fluorine-containing bicyclo[4.1.1]octenes. Our protocol is characterized by mild conditions, broad substrate scope, excellent functional group tolerance and good to excellent yields. Notably, the ease and variety of product derivatizations further enrich the diversity and complexity of the fluorine-containing bicyclo[4.1.1] systems.

## Abstract 2

This manuscript presents a photoredox-catalyzed defluorinative (4 + 3) annulation of bicyclo[1.1.0]butanes with *gem*-difluoroalkenes, providing practical and straightforward access to the fluorine-containing bicyclo[4.1.1]octenes.

## Graphical abstract

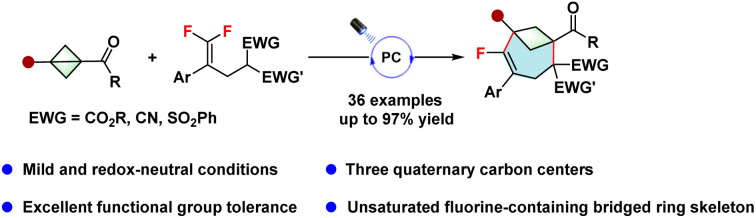

## Introduction

**Scheme 1:**
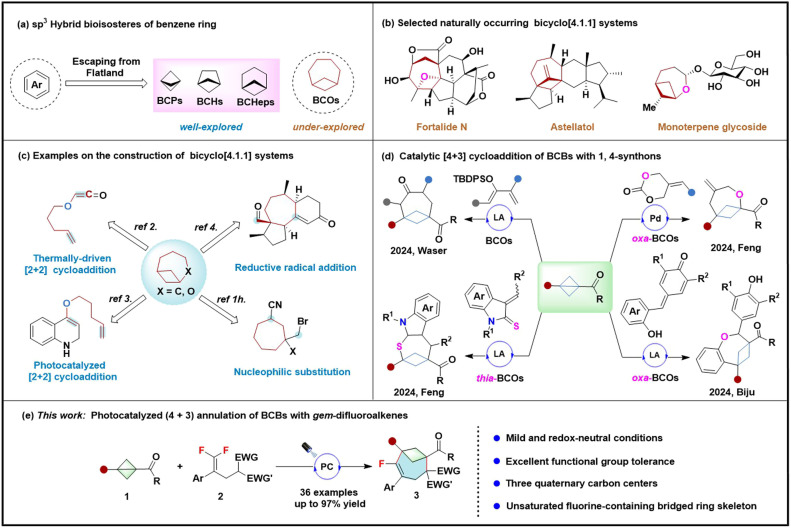
Importance and synthetic strategies of the bicyclo[*n*.1.1] systems.

In the realm of drug development, the concept of “escaping flatland” is gaining prominence, as sp^3^-hybridized bioisosteres such as bicyclo[1.1.1]pentanes (BCPs), bicyclo[2.1.1]hexanes (BCHs), and bicyclo[3.1.1]heptanes (BCHeps) can improve the pharmacokinetic properties of drug candidates by replacing benzene rings. Additionally, Grygorenko has reported that bicyclo[*n*.1.1] bridged cycloalkane derivatives (*n* > 3) are potential bioisosteres of disubstituted benzene rings. Among these, the bicyclo[4.1.1] systems are particularly significant scaffolds found in natural products and drug molecules (Scheme 1a and 1b).^1^ However, efficient synthetic strategies to access these scaffolds remain a challenge and are underdeveloped. Intramolecular cyclization represents an efficient strategy for the construction of bicyclic[4.1.1] systems (Scheme 1c). Oku and co-workers^2^ reported the synthesis of oxa-bicyclo[4.1.1]octanes (oxa-BCOs) by thermally-driven [2 + 2] cycloaddition. Bach and co-workers^3^ disclosed a photocatalyzed [2 + 2] cycloaddition to access these oxa-BCOs. Recently, Xu's group^4^ reported a SmI_2_-mediated reductive radical 1,6-addition strategy for constructing bicyclo[4.1.1]octane (BCO) scaffolds. Grygorenko and co-workers^1*h*^ reported an intramolecular nucleophilic substitution strategy for the BCO backbones. However, the requirement for complex substrates and the lack of generality have limited the applicability of this monomolecular reaction strategy. Given the significance of bicyclo[4.1.1] systems, there is still an urgent and ongoing need to develop universal and rapid synthetic methods for obtaining complex BCOs.

Bicyclo[1.1.0]butanes (BCBs) are the smallest fused carbocycles, possessing a strain energy of 267 kJ mol^−1^.^5^ Consequently, they are highly reactive and valuable synthons in many chemical transformations that involve the release of ring strain.^6^ Since Blanchard's pioneering work on the thermally induced [3 + 2] cycloaddition of BCBs with olefins, the cycloadditions of BCBs have garnered significant interests from chemists.^7^ A variety of [3 + 2] and [3 + 3] cycloadditions of BCBs have been developed through visible light-mediated energy transfer,^8^ visible light photoredox catalysis,^9^ transition metal catalysis,^10^ boron radical catalysis^11^ and Lewis acid catalysis,^12^ providing efficient strategies for the synthesis of bicyclo[2.1.1] and bicyclo[3.1.1] systems. However, there have been only a few reports on the construction of bicyclo[4.1.1] systems (Scheme 1d).^13^ Waser and co-workers demonstrated a Lewis acid-catalyzed [4 + 3] cycloaddition of BCBs with dienol ethers to afford the BCOs.^13*a*^ Feng and co-workers reported a Lewis acid-catalyzed [4 + 3] cycloaddition of BCBs with 3-benzylideneindoline-2-thiones to give the thia-BCOs.^13*b*^ They also reported the palladium-catalyzed decarboxylative [4 + 3] cycloaddition of BCBs with 2-alkylidenetrimethylene carbonates to yield the oxa-BCOs.^13*c*^ Biju and co-workers presented a Lewis acid-catalyzed (4 + 3) annulation of BCBs with *para*-quinone methides to give the oxa-BCOs.^13*d*^ Despite these fascinating achievements, the synthesis of unsaturated bicyclo[4.1.1]octenes has never been reported. Therefore, there is a significant need to explore new catalytic systems and unsaturated π systems for the annulation reactions of BCBs, which could greatly enrich the structural diversity of bicyclo[*n*.1.1] systems.

Due to the unique properties of the fluorine atom, organic fluorides represent an attractive class of candidates in the pharmaceutical, agrochemical and material sciences. It has been documented that 15–20% of the newly marketed drugs are organic fluorinated compounds.^14^ Thus, the incorporation of the fluorine atom into the bridged ring skeletons is an important but challenging task. In this context, the groups of Ma and Mykhailiuk separately reported a [3 + 1] cycloaddition of BCBs with difluorocarbenes, providing an intriguing strategy for the synthesis of 2,2-difluorobicyclo[1.1.1]pentanes (BCPs).^15^ However, the construction of the fluorinated bicyclo[*n*.1.1]alkanes (*n* ≥ 2) remains undeveloped. *gem*-Difluoroalkenes, as readily accessible fluorine-containing building blocks, have shown excellent performance in the construction of complex fluorinated compounds.^16^ We wonder whether *gem*-difluoroalkenes can serve as 4C synthons in the annulation reactions of BCBs to construct fluorine-containing bicyclo[4.1.1] systems (Scheme 1e). In this study, we report a visible light photoredox defluorinative (4 + 3) annulation of BCBs with *gem*-difluoroalkenes, which results in the formation of monofluorinated bicyclo[4.1.1]octenes. This protocol is characterized by readily available starting materials, mild reaction conditions and excellent functional group tolerance, providing a facile approach to the bicyclo[4.1.1]octenes featuring one fluorine atom and three quaternary carbon centers.

## Results and discussion

Initially, BCB 1a and *gem*-difluoroalkene 2a were chosen as model substrates to determine the optimal reaction conditions (Table 1). Fortunately, the (4 + 3) annulation of 1a with 2a proceeded efficiently using Ru(bpy)_3_Cl_2_ as photocatalyst and Cs_2_CO_3_ as base in CH_3_CN under 30 W blue LEDs irradiation for 12 h, yielding the desired monofluorinated bicyclo[4.1.1]octene 3aa in 72% NMR yield (entry 1). Other organic and Ir-based photocatalysts were also tested for this transformation, with Ru(bpy)_3_Cl_2_ still being the best (entries 2 and 3). Solvent screening showed that CH_3_CN was still the optimal solvent (entries 4 and 5). Screening of inorganic and organic bases showed that K_2_CO_3_ and K_3_PO_4_ were also effective, but both gave relatively lower yields than Cs_2_CO_3_ (entries 6 and 7). Satisfyingly, increasing the amount of 2a and Cs_2_CO_3_ to 1.5 equiv. improved the yield of 3aa to 80% (entry 8). Extending the reaction time from 12 h to 18 h further improved the yield of 3aa up to 88% (entry 9). Reducing the catalyst loading to 1 mol% still gave a comparable yield of 3aa (entry 10). Finally, control experiments indicated that the photocatalyst, base and visible light irradiation were all essential for this conversion (entry 11).

**Table 1:** Optimization of reaction conditions^a^

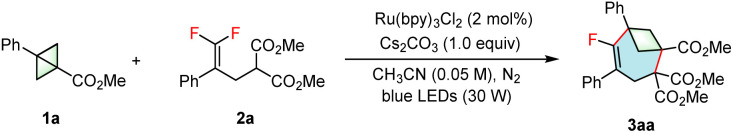		
Entry	Variation from standard conditions	Yield
1	None	72
2	4CzIPN, [Acr^+^-Mes]ClO_4_^−^	60, trace
3	Ir[dF(CF_3_)ppy_2_(dtbbpy)]PF_6_	68
4	Acetone or DCE as solvent	58, 37
5	THF or toluene as solvent	36, 65
6	K_2_CO_3_ or K_3_PO_4_ as base	36, 34
7	Et_3_N as base	Trace
8	1.5 equiv. of 2a and Cs_2_CO_3_	80
9	18 h	88
10	1 mol% Ru(bpy)_3_Cl_2_	89 (86)^b^
11	No PC or no base or no *hv*	0

a Reaction conditions: 1a (0.2 mmol, 1.0 equiv.), 2a (0.24 mmol, 1.2 equiv.), PC (2 mol%), Cs_2_CO_3_ (0.2 mmol, 1.0 equiv.), in CH_3_CN (0.05 M), blue LEDs (30 W), rt, for 12 h, under N_2_. Yields were determined by ^1^H NMR using 1,3,5-trimethoxybenzene as an internal standard.

b Isolated yield.

**Fig. 1:**
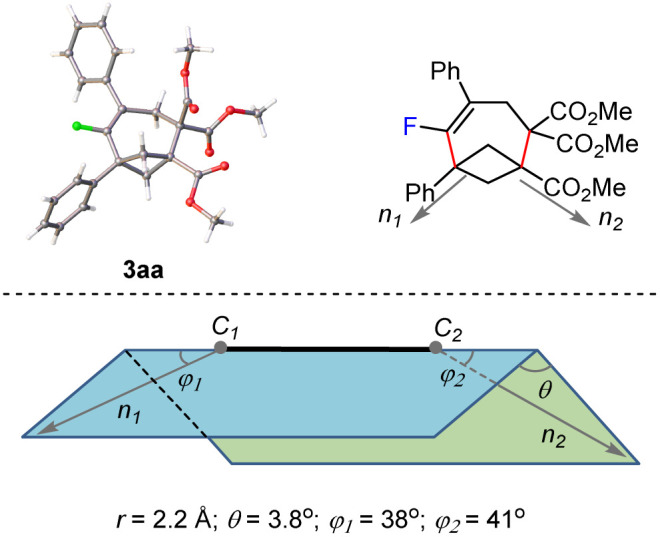
The corresponding values of the geometrical parameters associated with the exit vectors *n*_1_ and *n*_2_ of 3aa; geometrical definition of exit vectors and associated parameters.

With the optimal conditions in hand, the generality and limitations of the BCBs 1 were first evaluated using the *gem*-difluoroalkene 2a as a model substrate (Table 2). A variety of 1,3-disubstituted BCBs 1 bearing an ester group reacted efficiently with *gem*-difluoroalkene 2a to yield the desired fluorinated products 3aa–3pa in good to excellent yields. Among these, the structure of 3aa was further confirmed by single crystal X-ray diffraction. Meanwhile, we carried out a crystallographic analysis of the ORTEP diagram for 3aa and provided the values of the geometrical parameters (*r*, *θ*, *φ*_1_, *φ*_2_) associated with the exit vectors. As noted by Grygorenko and coworkers,^1*h*^ all these values fall within the β region of the exit vector plot, and therefore, can be considered as bioisosteres of *meta*-disubstituted benzene (Fig. 1). The electronic effect on the benzene ring didn't show any significant influence on the reaction efficiency. BCBs with electron-withdrawing or -donating groups at the *para*- and *meta*-positions of the benzene ring all reacted well to afford the desired products 3aa–3ha in excellent yields. Functional groups, such as Br (3da) and OCF_3_ (3ea) were fully compatible with this reaction. The BCB with an *ortho*-Me group on the benzene ring gave a relatively lower yield (3ia, 53% *vs.*3ba, 95%), probably due to steric hindrance. The 2-naphthyl substituted BCB 1j also worked efficiently and gave a moderate yield of 3ja. In addition to the methyl ester, BCBs containing various ester groups, including alkyl esters (1k and 1l), cycloalkyl esters (1m and 1n), benzyl ester (1o) and furan-2-ylmethyl ester (1p) all gave the corresponding products 3ka–3pa in 65–94% yields. The steric hindrance of the ester group did not affect the reaction efficiency. Remarkably, the BCBs obtained from complex natural alcohols such as menthol and citronellol were also applicable to this reaction, giving products 3qa and 3ra in 92% and 88% yields, respectively. The BCB containing an amide group (1s) was also a viable substrate, giving the product 3sa in 58% yield. Unfortunately, the substituted BCBs containing a carbonyl (1t and 1u) or amide group (1v) did not yield the expected products.

**Table 2:** Scope of BCBs^a^

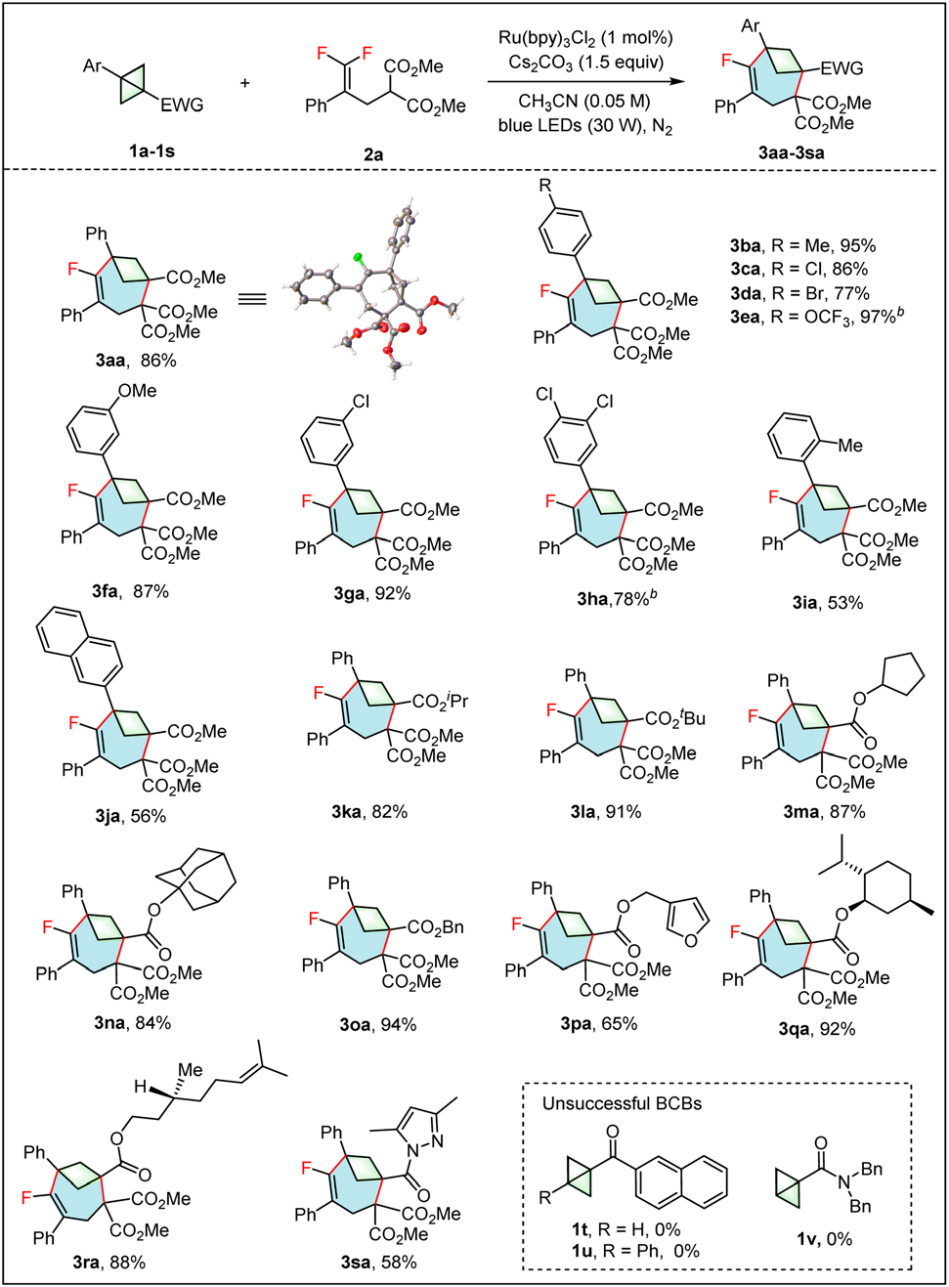

a Reaction conditions: 1 (0.2 mmol, 1.0 equiv.), 2a (0.3 mmol, 1.5 equiv.), Ru(bpy)_3_Cl_2_ (1 mol%), Cs_2_CO_3_ (0.3 mmol, 1.5 equiv.), CH_3_CN (0.05 M), under N_2_. Isolated yield. n. r. = no reaction.

b Using Ir[dF(CF_3_)ppy_2_(dtbbpy)]PF_6_ instead of Ru(bpy)_3_Cl_2_.

The scope of *gem*-difluoroalkenes 2 was then investigated using 1a as a model substrate (Table 3). A number of aryl *gem*-difluoroalkenes containing electron-donating and -withdrawing groups on the benzene ring were well engaged in this annulation reaction, giving the desired products 3ab–3an in good to excellent yields. Functional groups, such as OMe (3ae and 3ak), TMS (3af), Br (3ai) and acetyl (3al) all survived well in this transformation, providing opportunities for further product modification. The *gem*-difluoroalkene with a dibenzo[*b*,*d*]thiophen-3-yl group was also amenable, yielding the product 3an in 63% yield. In addition, the *gem*-difluoroalkene with two isopropyl ester groups also worked, but gave the product 3ao in only 38% yield due to the low conversion. Satisfactorily, the *gem*-difluoroalkene with two benzyl ester groups gave the target product 3ap in 85% yield. As anticipated, the *gem*-difluoroalkene with one ester group (2s) didn't engage in this annulation reaction, probably due to the polarity mismatch. Replacement of one ester group with a sulfonyl (2q) or cyano (2r) group also successfully led to the corresponding products 3aq and 3ar in good yields. However, no reaction was observed when both ester groups were replaced by cyano and sulfonyl groups (2t). The *gem*-difluoroalkene with a 1,3-benzodioxole substituent (2u) also failed to undergo this (4 + 3) annulation reaction due to the polarity mismatch. Unfortunately, the *gem*-difluoroalkene 2v, with an extended carbon chain, was incompatible with the reaction.

**Table 3:** Scope of *gem*-difluoroalkenes^a^

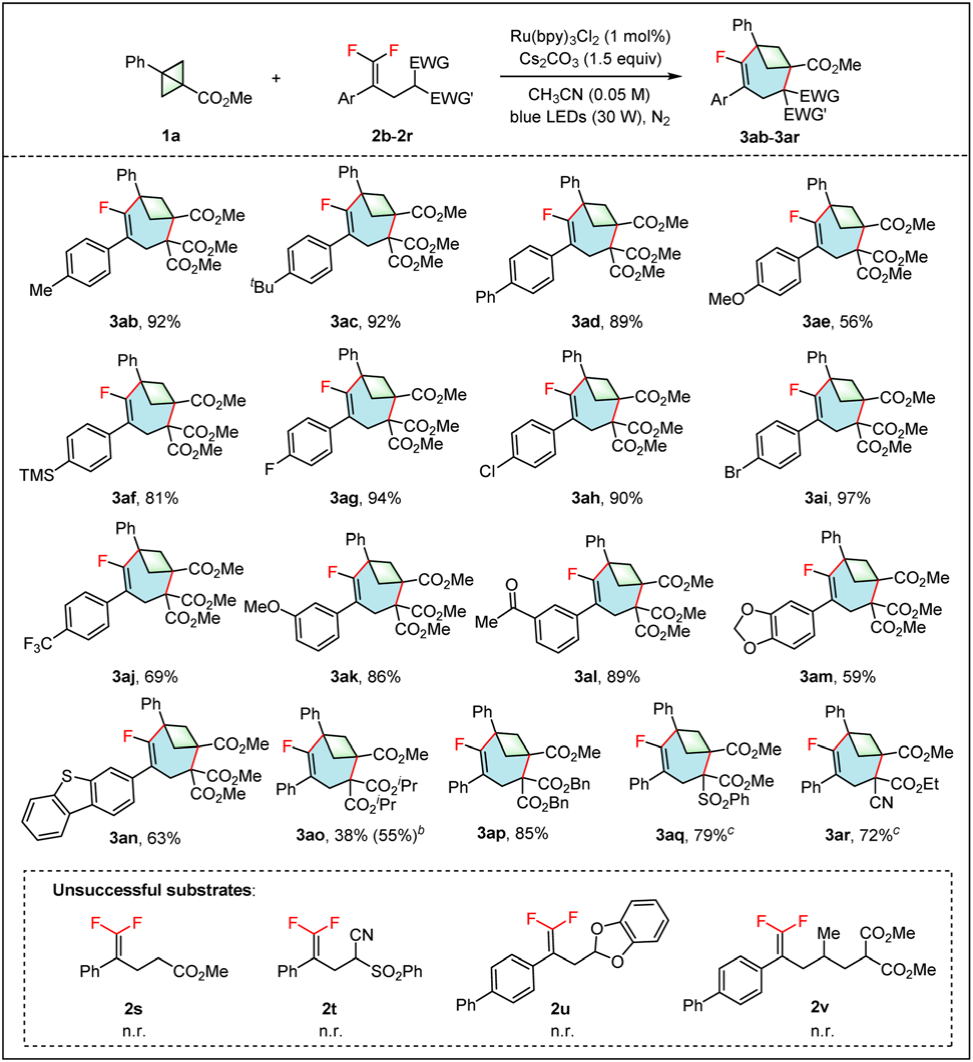

a Reaction conditions: 1a (0.2 mmol, 1.0 equiv.), 2 (0.3 mmol, 1.5 equiv.), Ru(bpy)_3_Cl_2_ (1 mol%), Cs_2_CO_3_ (0.3 mmol, 1.5 equiv.), CH_3_CN (0.05 M), under N_2_. Isolated yield. n. r. = no reaction.

b Recovery of 1a was given in parentheses.

c Using K_2_CO_3_ instead of Cs_2_CO_3_.

To demonstrate the potential application of this reaction, large-scale synthesis and derivatizations of products were carried out (Table 4). When the reaction was scaled up to 2.0 mmol, the monofluorinated bicyclo[4.1.1]octene 3aa was obtained in 83% isolated yield. Treatment of 3aa with LiCl and H_2_O at 140 °C afforded the decarboxylated product 4aa in 76% yield. Reduction of 3aa with LiAlH_4_ followed by treatment of the crude product with PTSA and cyclohexanone led to an unexpected product 5aa in 45% yield. When 3ka was treated with 2.5 equiv. of MeMgBr, two different lactones 6ka and 6ka′ were formed in 55% and 34% yields respectively. The *tert*-butyl ester group in 3la could be hydrolyzed using TFA to yield the product 7la in an 88% yield. The acid 7la could be reacted with α-CF_3_ alkene under photoredox catalysis to give the *gem*-difluoroalkene 8la in a 75% yield through a decarboxylation/radical addition/β-fluorine elimination cascade. Notably, the decarboxylative halogenations of 7la could also be achieved *via* an iron-catalyzed LMCT strategy, leading to the formation of the chloride 9la and bromide 10la in yields of 52% and 55%, respectively.

**Table 4:** Scale-up synthesis and derivatizations^a^

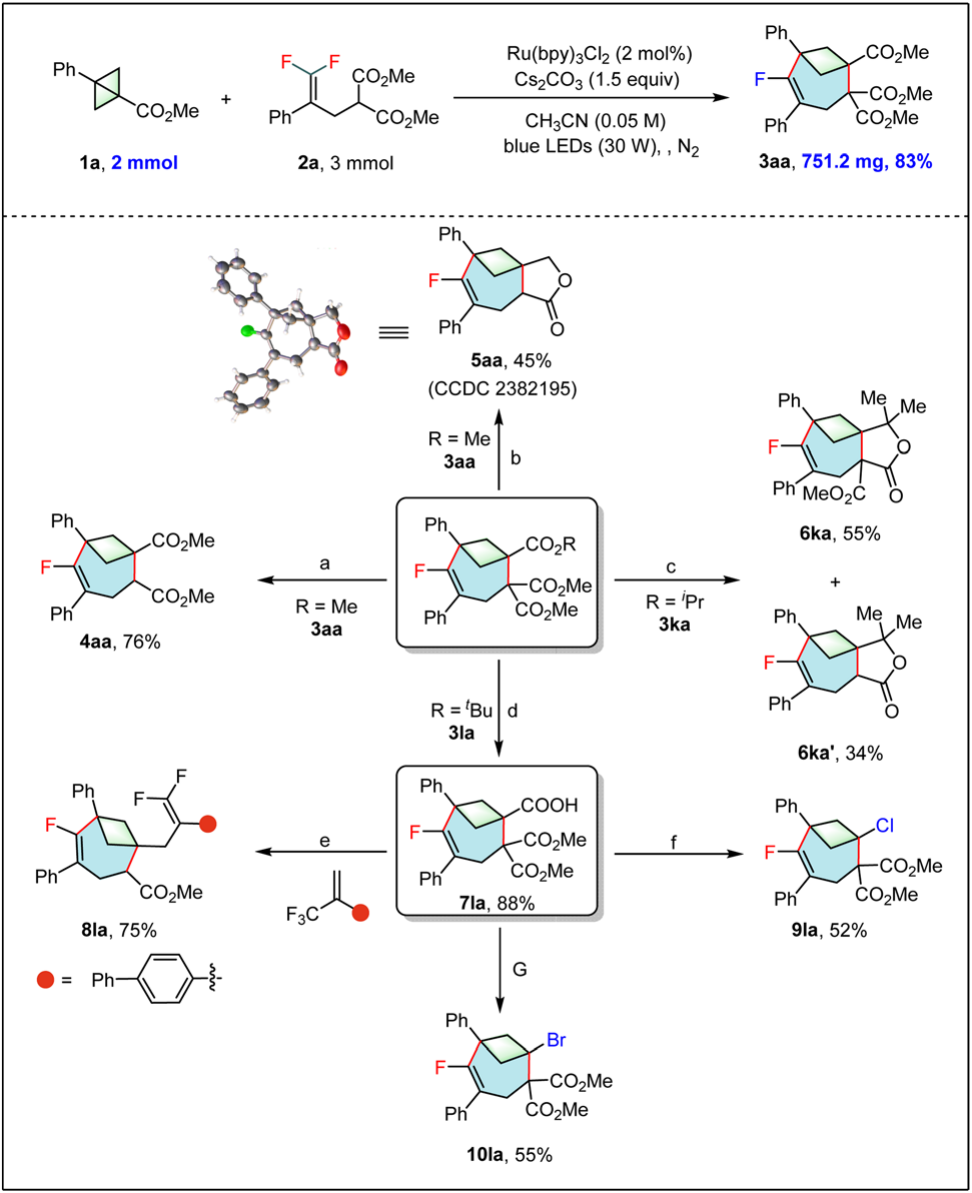

a Reaction conditions: (a) LiCl (1.0 equiv.), H_2_O (1.0 equiv.), DMSO, 140 °C; (b) LiAlH_4_ (5.0 equiv.), THF, 0 °C, then cyclohexanone (1.0 equiv.), PTSA (20 mol%), toluene, reflux; (c) MeMgBr (2.5 equiv.), THF, rt; (d) TFA (5.0 equiv.), DCM, rt; (e) 4CzIPN (2 mol%), Cs_2_CO_3_ (1.0 equiv.), DMSO, blue LEDs (30 W); (f) NCS (2.1 equiv.), Fe(OAc)_2_ (10 mol%), 4,4′-dimethoxy-2,2′-bipyridine (10 mol%), 2,4,6-collidine (1.8 equiv.), CH_3_CN, purple LEDs (390 nm); (g) NBS (2.1 equiv.), Fe(OAc)_2_ (10 mol%), 4,4′-dimethoxy-2,2′-bipyridine (10 mol%), 2,4,6-collidine (1.8 equiv.), CH_3_CN, purple LEDs (390 nm).

**Scheme 2:**
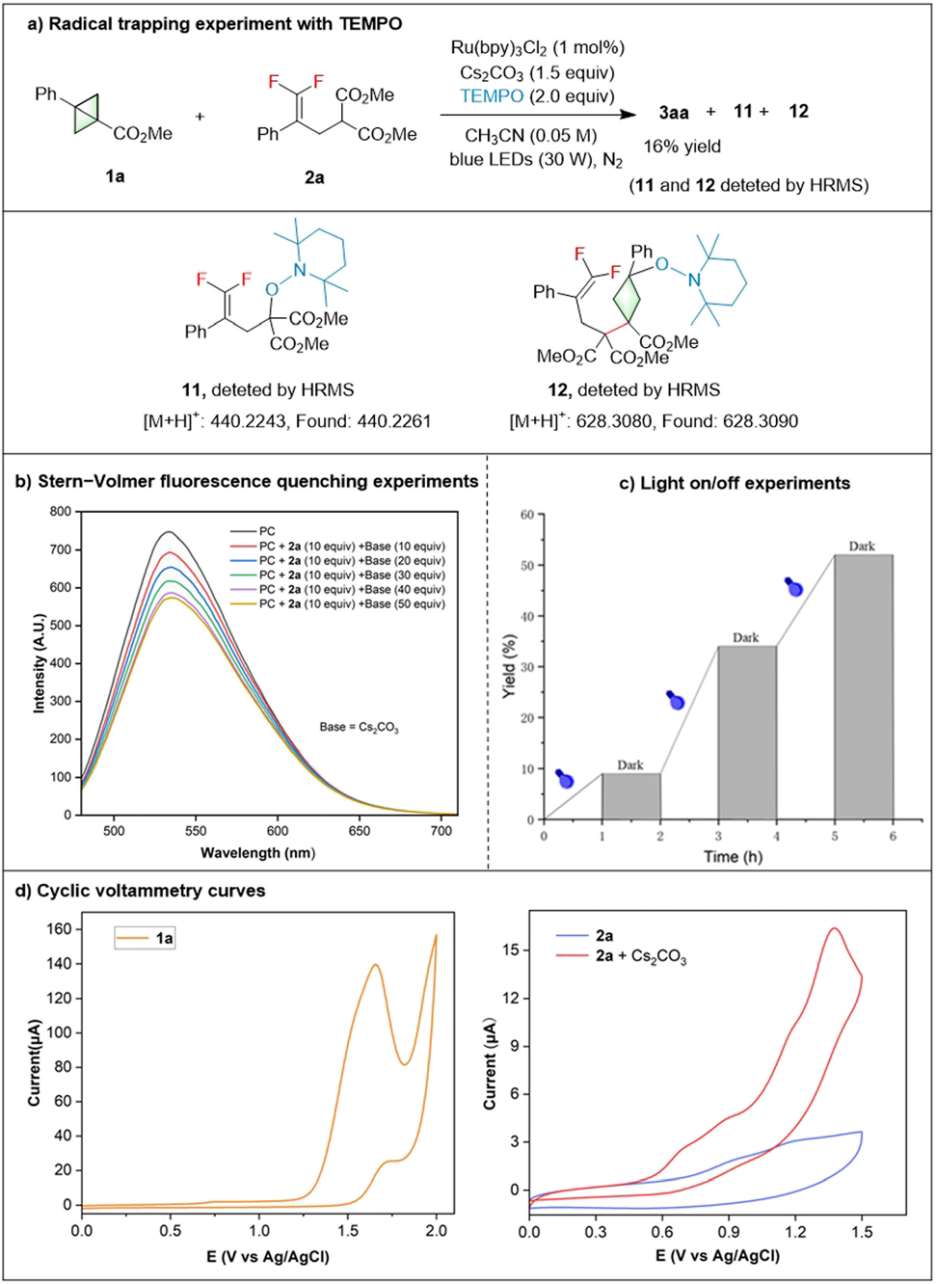
Mechanism studies.

To shed light on the mechanism of this annulation reaction, some control experiments were carried out (Scheme 2). Firstly, when 2.0 equiv. of TEMPO, a well-known radical scavenger, was added to the system, the model reaction was significantly inhibited, with the yield reduced from 89% to 16%. In addition, alkyl-TEMPO adducts 11 and 12 were detected by HRMS, indicating that the reaction proceeded *via* a radical pathway. A series of fluorescence quenching experiments were then carried out using 4CzIPN as the photosensitizer.^17^ It was found that the 4CzIPN* can be efficiently quenched by the anionic 2a, suggesting that an excited state charge transfer occurred between the 4CzIPN* and the anionic 2a. The light on/off experiments showed that continuous visible light irradiation was essential for this conversion. Finally, cyclic voltammetry (CV) experiments were conducted. BCB 1a exhibited an oxidation peak at +1.4 V *versus* the saturated calomel electrode (SCE), which is considered beyond the reach of the Ru(bpy)_3_Cl_2_ excited state at +0.77 V *versus* SCE. The oxidation potential of compound 2a was effectively lowered from 0.69 V to 0.56 V *versus* SCE in the presence of a base. These findings suggest that the oxidation of 2a by the Ru(bpy)_3_Cl_2_ excited state is thermodynamically favorable.

**Scheme 3:**
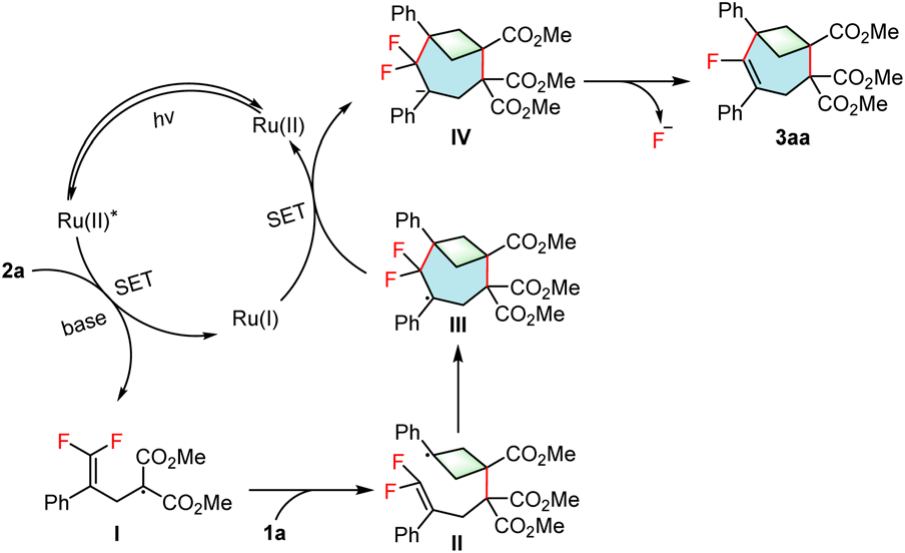
Proposed mechanism.

Based on the above results, a plausible pathway is proposed in Scheme 3. First, the ground state Ru(ii) is irradiated with visible light to form the excited state Ru(ii)*. A SET event then occurs between the Ru(ii)* and the deprotonated 2a to form the electrophilic radical intermediate I and Ru(i). Intermediate I then undergoes regioselective addition to BCB 1a to give the alkyl radical intermediate II. Intermediate II undergoes intramolecular radical addition to give the benzyl radical intermediate III, which is reduced by Ru(i) to give the carbanion intermediate IV and regenerate the Ru(ii) species. Finally, β-fluorine elimination takes place to give the target product 3aa.

## Conclusions

In summary, we have developed a photoredox (4 + 3) annulation of bicyclo[1.1.0]butanes (BCBs) with *gem*-difluoroalkenes that provides a facile approach to the all-carbon bicyclo[4.1.1]octenes with one fluorine atom and three quaternary carbon centers. This protocol features readily available starting materials, mild conditions, excellent functional group tolerance and good to excellent yields. Remarkably, the ease of large-scale synthesis and derivatizations highlights its potential application in organic synthesis. The incorporation of fluorine atom may further modify the physicochemical properties of this all-carbon bicyclo[4.1.1] system and would be valuable for the development of new drugs.

## Data availability

The authors confirm that the data supporting the findings of this study are available within the ESI.†

## Author contributions

K. Z. performed all the experiments and prepared the manuscript and ESI.† Z. G. and Y. X. performed the preparation of raw materials. P. L., P. G., X.-H. D. and L.-N. G. directed this project and revised the manuscript.

## Conflicts of interest

There are no conflicts to declare.

## Supplementary Material

SC-OLF-D4SC07243J-s001

SC-OLF-D4SC07243J-s002
