# Supplementary material for: Synthesis of fluorine-containing bicyclo[4.1.1]octenes via photocatalyzed defluorinative (4 + 3) annulation of bicyclo[1.1.0]butanes with gem-difluoroalkenes
Source: Chem Sci. 2024 Dec 13;16(3):1411–6. doi: 10.1039/d4sc07243j (PMC11659875; doi:10.1039/d4sc07243j)
Supplement: SC-016-D4SC07243J-s001 [file SC-016-D4SC07243J-s001.pdf]

Supplementary Information (SI) for Chemical Science.
This journal is © The Royal Society of Chemistry 2024

Synthesis of fluorine-containing bicyclo[4.1.1]octenes via photocatalyzed defluorinative (4+3) annulation of bicyclo[1.1.0]butanes with *gem*-difluoroalkenes

Kuan Zhang,[a] Zhengyang Gao,[a] Yan Xia,[a] Pengfei Li,[a, b] Pin Gao,[a] Xin-Hua Duan,[a] Li-Na Guo*[a]

[a]Department of Chemistry, School of Chemistry, Xi'an Key Laboratory of Sustainable Energy Material Chemistry and Engineering Research Center of Energy Storage, Materials and Devices, Ministry of Education, Xi'an Jiaotong University, Xi'an 710049, China

[b]Frontier Institute of Science and Technology and State Key Laboratory for Mechanical Behavior of Materials, Xi'an Jiaotong University, Xi'an 710049, China

Email: guoln81@xjtu.edu.cn

Table of Contents

1. General Information S3

2. The Light Source and the Material of the Irradiation Vessel S4

3. Starting Materials S5

3.1 Preparation of BCBs (**1n**, **1r**) S5

3.2 Preparation of *gem*-Difluoroalkenes (**2b-2r** and **2t**) S8

4. Detailed Optimization of Reaction Conditions S11

5. Representative Procedure for the Reaction of BCBs **1** and *gem*-Difluoroalkenes **2** S17

6. Scale-Up Synthesis S17

7. Derivatizations of Products S18

7.1 Synthesis of the Compound **4aa**[7] S18

7.2 Synthesis of the Compound **5aa**[8] S18

7.3 Synthesis of the Compound **6ka** and **6ka'**[9] S19

7.4 Synthesis of the Compound **7la**[10] S19

7.5 Synthesis of the Compound **8la**[11] S19

7.6 Synthesis of the Compound **9la**[12] S20

7.7 Synthesis of the Compound **10la**[12] S20

8 Mechanistic Investigation S22

8.1 TEMPO-Trapping Experiment S22

8.2 Stern-Volmer Fluorescence Quenching Experiments S23

8.3 Light On-Off Experiments S25

8.4 Cyclic voltammetry (CV) measurements S26

9 Characterization Date of Products S28

10 Single-crystal X-ray diffraction data for **3aa** and **5aa** S45

11 References S48

12 ^{1}H NMR, ^{19}F NMR and ^{13}C NMR Spectra of Products S49

1. General Information

Unless otherwise noted, all reactions were carried out under nitrogen atmosphere, reagents and solvents were obtained from commercial suppliers and were used without further purification. Analytical TLC: aluminum backed plates pre-coated (0.25 mm) with Merck Silica Gel 60F-254. Column chromatography purifications were carried out using 200-300 mesh silica gel. Melting points were measured using open glass capillaries in a SGW® X-4A apparatus. ^{1}H NMR spectra were recorded on a JNM-ECZ400S/L1 400 MHz spectrometer at ambient temperature. ^{13}C NMR spectra were recorded on a JNM-ECZ400S/L1 100 MHz spectrometer at ambient temperature. ^{19}F NMR spectra were recorded on a JNM-ECZ400S/L1 376 MHz spectrometer at ambient temperature. Coupling constants are reported in Hz with multiplicities denoted as s (singlet), d (doublet), t (triplet), q (quartet), dd (doublet of doublets), td (triplet of doublets), m (multiplet) and br (broad). Infrared spectra were recorded on a Bruker V 70 and only major peaks were reported in cm^{-1}. HRMS were obtained on a WATERS I-Class VION IMS Q-Tof with an ESI source. Some compounds were visualized by exposure to UV-light or by dipping the plates in $KMnO_4$ stain followed by heating. X-ray data were taken on a Bruker D8 VENTURE X-Ray diffractometer.

2. The Light Source and the Material of the Irradiation Vessel

Light Source purchased from XuSheng Electronic Technology.

Broadband source： λ = 455-460 nm

Spectral distribution and intensity：

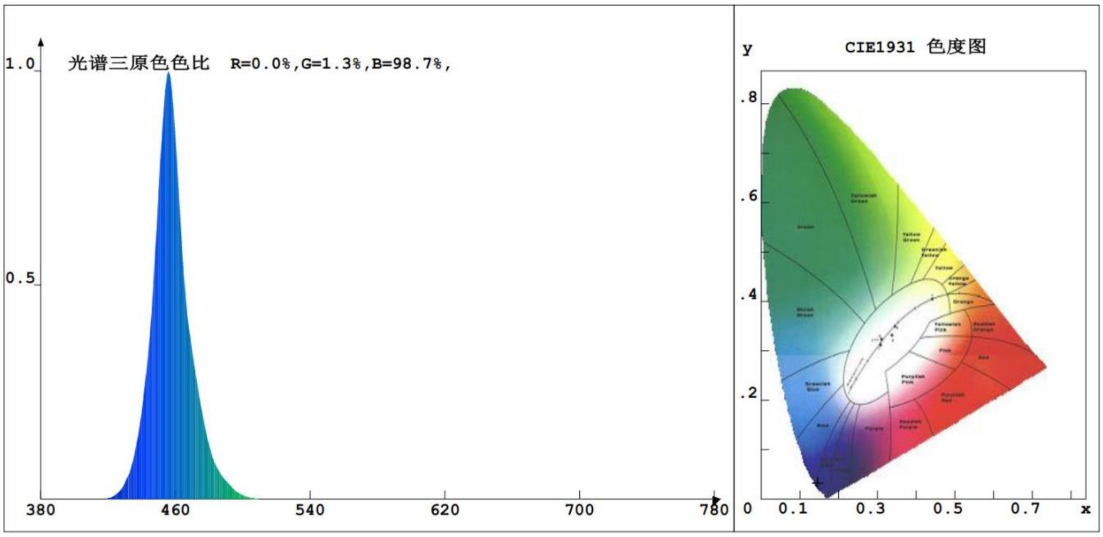

Material of the irradiation vessel: borosilicate reaction tube

Distance from the light source to the irradiation vessel: 3.0 cm

Not use any filters

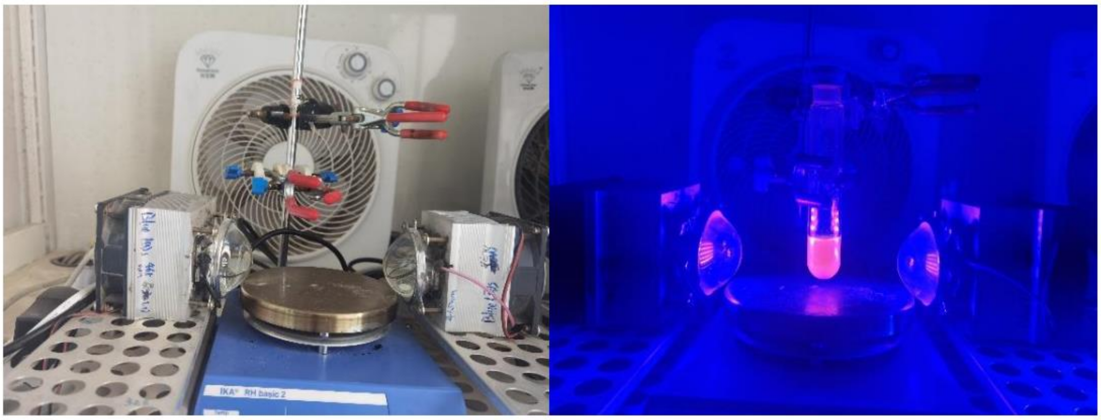

The setting-up reactions.

3. Starting Materials

The bicyclobutanes **1** and *gem*-difluoroalkenes **2** were prepared according to the literature. The NMR spectra of the know compounds were in full accordance with the data in the literatures.

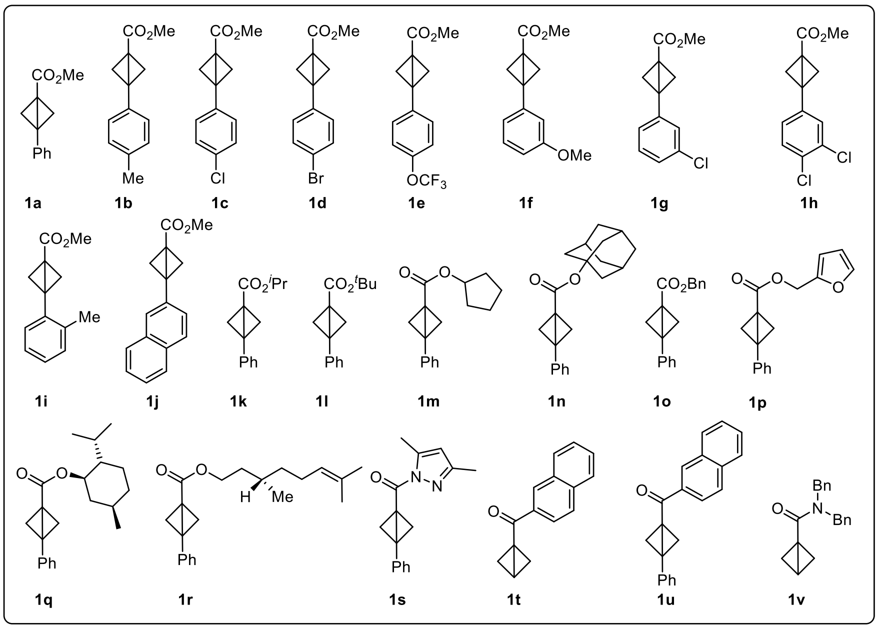

Except for **1n** and **1r**, the above bicyclo[1.1.0]butanes **1a-1m**,[1] **1o-1v**[1,2] are known and were prepared according to corresponding literature.

3.1 Preparation of BCBs (1n, 1r)

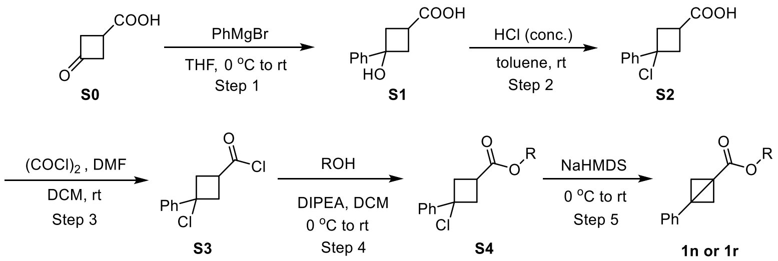

Step 1 (synthesis of S1)

An oven-dried 500 mL round bottom flask equipped with a stir bar was cooled under vacuum. After backfilled with N_2 (3 times) and capped with a septum, 3-oxocyclobutanecarboxylic acid (5.7 g, 50 mmol, 1.00 equiv) and THF (100 mL) were added. The reaction was cooled to 0 ℃ in an ice/water bath and PhMgBr (110 mL, 1.0 M in THF, 110 mmol, 2.2 equiv) was added to the solution by a syringe. The ice/water bath was then removed and the reaction was stirred at room temperature for 12 h before quenched with saturated NH_4Cl solution (50 mL). The aqueous layer was extracted with EtOAc (3 x 50 mL). The combined organic layers were washed with brine (100

mL), dried over anhydrous $MgSO_4$, filtered and concentrated by rotary evaporation. The crude acid was directly used in next reaction without further purification.

Step 2 (synthesis of S2)

A 500 mL round bottom flask equipped with a magnetic stir bar was charged with above crude acid **S1** (assuming 50 mmol, 1.0 equiv), concentrated HCl solution (50 mL) and PhMe (60 mL). The flask was capped with a septum. The reaction was vigorously stirred at room temperature for 12 h. The aqueous layer was extracted with Et_2O (3 x 50 mL). The combined organic layers were washed with brine (100 mL), dried over anhydrous $MgSO_4$, filtered and concentrated by rotary evaporation. The crude acid was directly used in next reaction without further purification.

Step 3 (synthesis of S3)

3-Chloro-3-phenylcyclobutane-1-carboxylic acid **S2** (2.98 g, 15.5 mmol) was added to a 100 mL round bottom flask and dissolved in 20 mL of DCM. A drop of DMF was added to the reaction mixture. Oxalyl chloride (3.5 mL, 38.8 mmol) dissolved in 5 mL DCM was added dropwise over a period of 20 min at room temperature. The reaction mixture was stirred for 24 h. The solvent was removed by vacuum filtration and a brown oil was obtained as a mixture of diastereomers (2.5 g, 70%). The product was used in the next step without further purification.

Step 4 (synthesis of S4)

A solution of alcohol (1.2 equiv) and DIPEA (1.0 equiv) in DCM was added to a vial, and then was cooled to 0 ℃. 3-Chloro-3-phenylcyclobutane-1-carbonyl chloride **S3** (1.0 equiv) dissolved in DCM was added dropwise to the vial. The reaction mixture was then warmed to room temperature and stirred for 2 h. The reaction mixture was washed with water and then the solvent was removed under vacuum to give the crude product. The compound was purified by column chromatography.

Step 5 (synthesis of 1n or 1r)

A solution of 3-chloro-3-arylcyclobutane ester **S4** (1.0 equiv) in THF was added to a vial under a nitrogen atmosphere. NaHMDS (1.0 equiv) was added to the vial and the reaction mixture was stirred at 0 ℃ for 0.5 h. The mixture was warmed to room temperature and stirred for 1.5 h before quenched with saturated NH_4Cl solution. The aqueous layer was extracted with EtOAc, The combined organic layers were washed with brine, dried over anhydrous $MgSO_4$, filtered and concentrated by rotary evaporation, the residue was purified by column chromatography to afford

desired products.

Characterization of Starting Materials

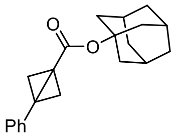

Adamantan-1-yl 3-phenylbicyclo[1.1.0]butane-1-carboxylate (1n): White solid (562 mg, 25% over 5 steps); m.p.: 101-102 ^{o}C; R_f = 0.4 (petroleum ether/EtOAc = 30/1); ^{1}H NMR (400 MHz, $CDCl_3$): δ = 7.31-7.28 (m, 4H), 7.25-7.20 (m, 1H), 2.89 (t, *J* = 1.2 Hz, 2H), 2.0 (s, 3H), 1.75 (d, *J* = 2.8 Hz, 6H), 1.55 (s, 2H), 1.54-1.51 (m, 6H); ^{13}C NMR (100 MHz, $CDCl_3$): δ = 168.6, 133.9, 128.2, 126.6, 125.8, 80.4, 41.1, 36.0, 35.7, 31.9, 30.7, 23.8 ppm; HRMS (ESI) calcd for $C_{21}H_{25}O_2$ $[M+H]^+$ 309.1849, found 309.1843.

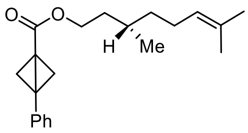

(*S*)-3,7-dimethyloct-6-en-1-yl 3-phenylbicyclo[1.1.0]butane-1-carboxylate (1r): Colorless oil (660 mg, 28% over 5 steps); R_f = 0.4 (petroleum ether/EtOAc = 30/1); ^{1}H NMR (400 MHz, $CDCl_3$): δ = 7.30-7.27 (m, 4H), 7.25-7.20 (m, 1H), 5.06 (t, *J* = 7.2 Hz,1H), 3.91 (t, *J* = 6.8 Hz, 2H), 2.94 (s, 2H), 1.95-1.83 (m, 2H), 1.69 (s, 3H), 1.60 (s, 5H), 1.39-1.28 (m, 2H), 1.23-1.02 (m, 3H), 0.73 (d, *J* = 6.4 Hz, 3H); ^{13}C NMR (100 MHz, $CDCl_3$): δ = 169.7, 133.6, 131.2, 128.4, 126.9, 125.8, 124.6, 62.9, 36.8, 35.7, 35.5, 32.7, 29.1, 25.7, 25.2, 23.3, 19.1, 17.6 ppm; HRMS (ESI) calcd for $C_{21}H_{29}O_2$ $[M+H]^+$ 313.2162, found 313.2158.

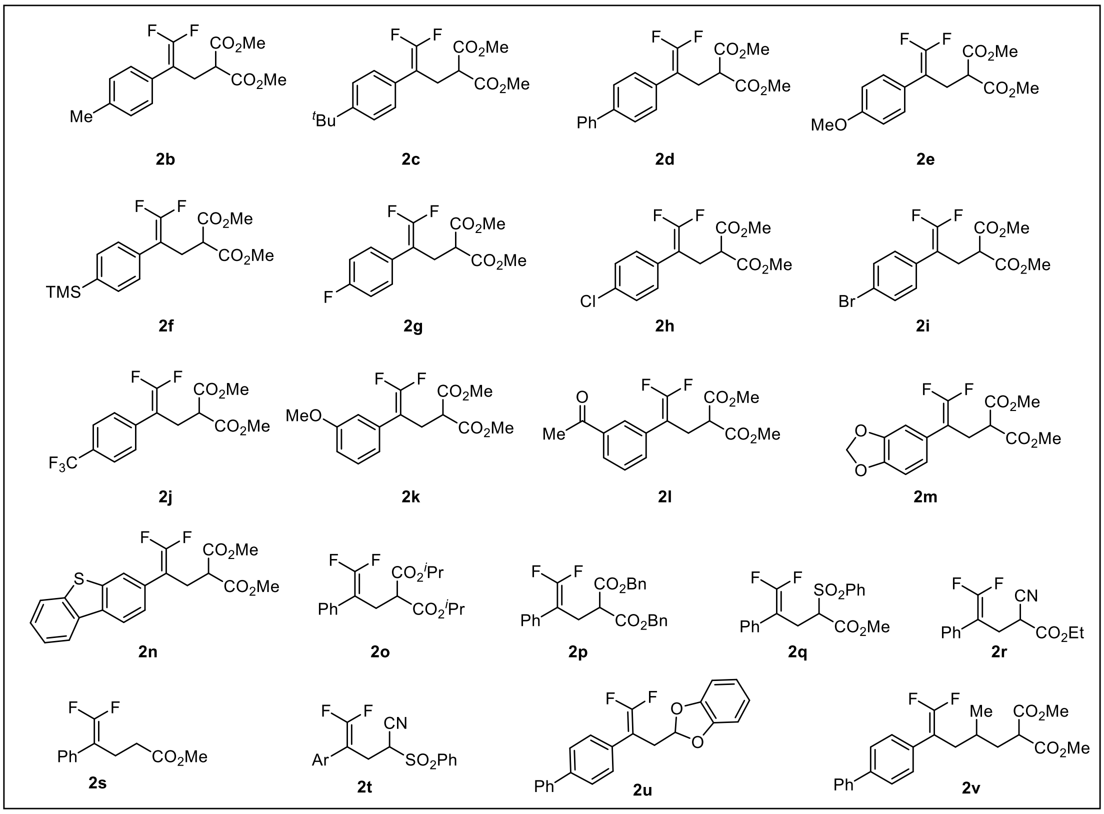

gem-Difluoroalkenes **2b-2r** and **2t** were prepared according to the reported methods.[3] **2s**[4] , **2u**[5]and **2v**[6] were prepared according to the reported methods.

3.2 Preparation of *gem*-Difluoroalkenes (2b-2r and 2t)

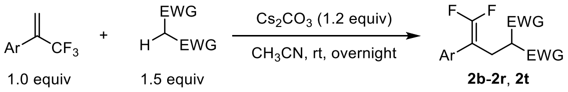

A round bottom flask equipped with a stir bar was charged with Cs_2CO_3 (6.0 mmol, 1.2 equiv), then the flask was evacuated and backfilled with N_2 for 3 times. A solution of nucleophile (7.5 mmol, 1.5 equiv) in CH_3CN (0.2 M) was successively added via a syringe under N_2 atmosphere and stirred at room temperature for 1 hour. Then trifluoromethyl alkene (5.0 mmol, 1.0 equiv) in CH_3CN (0.5 M) was added to the reaction mixture and stirred at room temperature for overnight. After the reaction was completed, H_2O was added to quench the reaction and separated. The aqueous phase was extracted with EtOAc (x 3 times). The combined organic phases were washed with brine and dried over Na_2SO_4. After filtration, the solvent was evaporated under the reduced pressure, the residue was purified by silica gel column chromatography (eluent: PE/EA = 20:1-5:1) to afford the *gem*-difluoroalkenes **2b-2r** and **2t**.

Characterization of Starting Materials

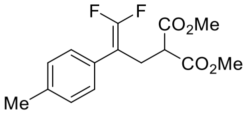

Dimethyl 2-(3,3-difluoro-2-(*p*-tolyl)allyl)malonate (2b): colorless oil (81%, 1.20 g); R_f = 0.3 (petroleum ether/EtOAc = 10/1); ^{1}H NMR (400 MHz, $CDCl_3$): δ = 7.18-7.14 (m, 4H), 3.66 (s, 6H), 3.36 (t, *J* = 8.0 Hz, 1H), 3.01 (d, *J* = 7.6 Hz, 2H), 2.34 (s, 3H); ^{19}F NMR (376 MHz, $CDCl_3$): δ = -89.59 (d, *J* = 38.7 Hz), -89.94 (d, *J* = 38.4 Hz); ^{13}C NMR (100 MHz, $CDCl_3$): δ = 168.9, 153.9 (t, *J* = 288.3 Hz), 137.6, 129.3, 128.9, 128.3, 89.1 (dd, *J* = 20.5, 17.1 Hz), 52.5, 49.8, 27.4, 21.1 ppm; HRMS (ESI) calcd for $C_{15}H_{17}F_2O_4$ $[M+H]^+$ 299.1089, found 299.1085.

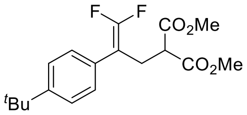

Dimethyl 2-(2-(4-(tert-butyl)phenyl)-3,3-difluoroallyl)malonate (2c): colorless oil (76%, 1.29 g); R_f = 0.4 (petroleum ether/EtOAc = 20/1); ^{1}H NMR (400 MHz, $CDCl_3$): δ = 7.37 (d, *J* = 8.0 Hz, 2H), 7.20 (d, *J* = 8.0 Hz, 2H), 3.66 (s, 6H), 3.38 (t, *J* = 8.0 Hz, 1H), 3.02 (dt, *J* = 7.6, 2.0 Hz, 2H), 1.31 (s, 9H); ^{19}F NMR (376 MHz, $CDCl_3$): δ = -89.44 (d, *J* = 39.1 Hz), -89.74 (d, *J* = 38.7 Hz); ^{13}C NMR (100 MHz, $CDCl_3$): δ = 168.9, 153.9 (t, *J* = 288.3 Hz), 150.7, 128.8, 128.1, 125.5, 89.0 (dd, *J* = 20.8, 17.2 Hz), 52.5, 49.9, 34.5, 31.2, 27.4. ppm; HRMS (ESI) calcd for $C_{18}H_{23}F_2O_4$ $[M+H]^+$ 341.1559, found 341.1552.

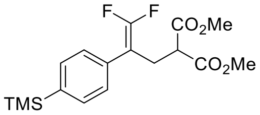

Dimethyl 2-(3,3-difluoro-2-(4-(trimethylsilyl)phenyl)allyl)malonate (2f): colorless oil (68%, 1.21 g); R_f = 0.4 (petroleum ether/EtOAc = 20/1); ^{1}H NMR (400 MHz, $CDCl_3$): δ = 7.51 (d, *J* = 8.0 Hz, 2H), 7.25 (d, *J* = 8.0 Hz, 2H), 3.66 (s, 6H), 3.40-3.35 (m, 1H), 3.07-3.02 (m, 2H), 0.27 (s, 9H); ^{19}F NMR (376 MHz, $CDCl_3$): δ = -88.91 (d, *J* = 36.8 Hz), -89.17 (d, *J* = 36.5 Hz); ^{13}C NMR (100 MHz, $CDCl_3$): δ = 168.9, 154.0 (t, *J* = 289.2 Hz), 140.2, 133.6, 132.3, 127.6, 89.3 (dd, *J* = 20.1, 16.9 Hz), 52.6, 49.8, 27.3, -1.3 ppm; HRMS (ESI) calcd for $C_{17}H_{23}F_2O_4Si$ $[M+H]^+$ 357.1328, found 357.1328.

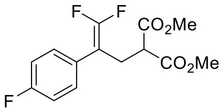

Dimethyl 2-(3,3-difluoro-2-(4-fluorophenyl)allyl)malonate (2g): colorless oil (51%, 0.77 g); R_f = 0.3 (petroleum ether/EtOAc = 10/1); ^{1}H NMR (400 MHz, $CDCl_3$): δ = 7.26-7.22 (m, 2H), 7.08-7.03 (m,

2H), 3.66 (s, 6H), 3.36-3.32 (m, 1H), 3.02-2.98 (m, 2H); ^{19}F NMR (376 MHz, $CDCl_3$): δ = -89.15 (d, *J* = 38.4 Hz), -89.36 (d, *J* = 38.0 Hz), -113.5--113.6 (m); ^{13}C NMR (100 MHz, $CDCl_3$): δ = 168.7, 162.1 (d, *J* = 246.4 Hz), 153.9 (t, *J* = 289.3 Hz), 130.3 (d, *J* = 7.9 Hz), 127.9, 115.6 (d, *J* = 21.3 Hz), 88.5 (dd, *J* = 21.3, 17.3 Hz), 52.6, 49.8, 27.5 ppm; HRMS (ESI) calcd for $C_{14}H_{14}F_3O_4$ $[M+H]^+$ 303.0839, found 303.0846.

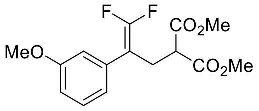

Dimethyl 2-(3,3-difluoro-2-(3-methoxyphenyl)allyl)malonate (2k): colorless oil (53%, 0.83 g); R_f = 0.3 (petroleum ether/EtOAc = 10/1); ^{1}H NMR (400 MHz, $CDCl_3$): δ = 7.27 (t, *J* = 8.0 Hz, 1H), 6.86-6.80 (m, 3H), 3.80 (s, 3H), 3.66 (s, 6H), 3.37 (t, *J* = 8.4 Hz, 1H), 3.01 (d, *J* = 7.6 Hz, 2H); ^{19}F NMR (376 MHz, $CDCl_3$): δ = -88.49 (d, *J* = 36.8 Hz), -89.29 (d, *J* = 36.8 Hz); ^{13}C NMR (100 MHz, $CDCl_3$): δ = 168.8, 159.6, 153.9 (t, *J* = 288.9 Hz), 133.3, 129.6, 120.7, 114.3, 113.2, 89.2 (dd, *J* = 20.9, 16.5 Hz), 55.2, 52.6, 49.8, 27.4 ppm; HRMS (ESI) calcd for $C_{15}H_{17}F_2O_5$ $[M+H]^+$ 315.1039, found 315.1043.

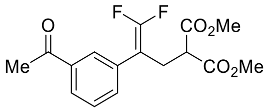

Dimethyl 2-(2-(3-acetylphenyl)-3,3-difluoroallyl)malonate (2l): colorless oil (39%, 0.63 g); R_f = 0.2 (petroleum ether/EtOAc = 5/1); ^{1}H NMR (400 MHz, $CDCl_3$): δ = 7.88-7.85 (m, 2H), 7.46 (d, *J* = 4.8 Hz, 2H), 3.65 (s, 6H), 3.33 (t, *J* = 8.0 Hz, 1H), 3.05 (d, *J* = 7.6 Hz, 2H), 2.60 (s, 3H); ^{19}F NMR (376 MHz, $CDCl_3$): δ = -88.21 (s); ^{13}C NMR (100 MHz, $CDCl_3$): δ = 197.6, 168.6, 154.1 (t, *J* = 289.3 Hz), 137.4, 133.1, 132.7, 129.0, 128.2, 127.8, 88.9 (t, *J* = 18.9 Hz), 52.6, 49.8, 27.3, 26.6 ppm; HRMS (ESI) calcd for $C_{16}H_{17}F_2O_5$ $[M+H]^+$ 327.1039, found 327.1031.

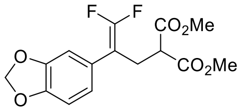

Dimethyl 2-(2-(benzo[*d*][1,3]dioxol-5-yl)-3,3-difluoroallyl)malonate (2m): colorless oil (66%, 1.08 g); R_f = 0.2 (petroleum ether/EtOAc = 5/1); ^{1}H NMR (400 MHz, $CDCl_3$): δ = 6.79-6.70 (m, 3H), 5.95 (s, 2H), 3.67 (s, 6H), 3.36 (t, *J* = 8.0 Hz, 1H), 2.95 (dt, *J* = 8.0, 2.4 Hz, 2H); ^{19}F NMR (376 MHz, $CDCl_3$): δ = -89.46 (d, *J* = 38.7 Hz), -90.16 (d, *J* = 38.4 Hz); ^{13}C NMR (100 MHz, $CDCl_3$): δ = 168.8, 153.9 (t, *J* = 287.8 Hz), 147.8, 147.1, 125.4 (dd, *J* = 4.3, 2.6 Hz), 122.0 (t, *J* = 2.9 Hz), 108.9 (t, *J* = 3.2 Hz), 108.4, 101.2, 89.0 (dd, *J* = 21.4, 17.0 Hz), 52.5, 49.7 (t, *J* = 2.9 Hz), 27.7 (d, *J* = 2.1 Hz) ppm;

HRMS (ESI) calcd for $C_{15}H_{15}F_2O_6$ $[M+H]^+$ 329.0831, found 329.0835.

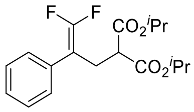

Diisopropyl 2-(3,3-difluoro-2-phenylallyl)malonate (2o): colorless oil (88%, 1.50 g); R_f = 0.5 (petroleum ether/EtOAc = 10/1); ^{1}H NMR (400 MHz, $CDCl_3$): δ = 7.32-7.22 (m, 5H), 4.99-4.90 (m, 2H), 3.19 (t, *J* = 8.0 Hz, 1H), 2.95 (dt, *J* = 8.0, 2.4 Hz, 2H), 1.16 (d, *J* = 2.4 Hz, 6H), 1.14 (d, *J* = 2.4 Hz, 6H); ^{19}F NMR (376 MHz, $CDCl_3$): δ = -89.60 (d, *J* = 38.4 Hz), -89.80 (d, *J* = 38.4 Hz); ^{13}C NMR (100 MHz, $CDCl_3$): δ = 168.0, 153.8 (t, *J* = 288.3 Hz), 132.1, 128.6, 128.0, 127.7, 89.4 (dd, *J* = 20.5, 16.6 Hz), 69.0, 50.4, 27.1, 21.5, 21.4 ppm; HRMS (ESI) calcd for $C_{18}H_{22}F_2NaO_4$ $[M+Na]^+$ 363.1378, found 363.1363.

4. Detailed Optimization of Reaction Conditions

A 10 mL oven-dried Schlenk tube equipped with a magnetic stirrer was charged with BCB **1a** (0.2 mmol, 1.0 equiv), *gem*-difluoroalkene **2a** (0.3 mmol, 1.5 equiv), base (0.3 mmol, 1.5 equiv) and PC (2 mol%). Then the tube was evacuated and backfilled with nitrogen for three times., dry solvent (4 mL) was added by a syringe. The reaction mixture was then irradiated with 30 W blue LEDs (distance app. 3.0 cm from the bulb) at room temperature for 12 h. The solvent was removed in vacuo, 1,3,5-trimethoxybenzene (32.8 mg, 0.2 mmol, 1.0 equiv) was added as an internal standard, and yield was determined by ^{1}H NMR analysis of the crude mixture. The results are compiled in **Table S1**.

Table S1 Optimization of the Reaction of BCB 1a and *gem*-Difluoroalkene 2a[a]

Catalyst

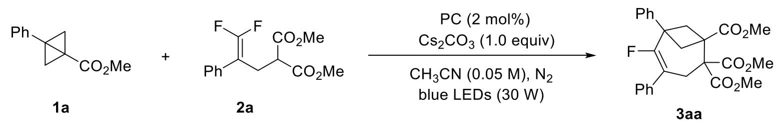

entry	PC	yield[a] (%)
1	4CzIPN	60
2	TPT	trace
3	$[Acr^{+}\text{-Mes}]ClO_4^{-}$	trace
4	$Ir[dF(CF_3)ppy_2(dtbbpy)]PF_6$	68
5	$Ru(bpy)_3Cl_2 \cdot 6H_2O$	63
**6**	**$Ru(bpy)_3Cl_2$**	**72**
7	$Ir(ppy)_3$	61

[a]Reaction conditions: **1a** (0.2 mmol, 1.0 equiv), **2a** (0.24 mmol, 1.2 equiv), PC (2 mol%), Cs_2CO_3 (0.2 mmol, 1.0 equiv), in CH_3CN (0.05 M), blue LEDs (30 W), rt, for 12 h, under N_2. TPT=2,4,6-triphenylpyrylium tetrafluoroborate. Yields were determined by ^1H NMR using 1,3,5-trimethoxybenzene as an internal standard.

Solvent

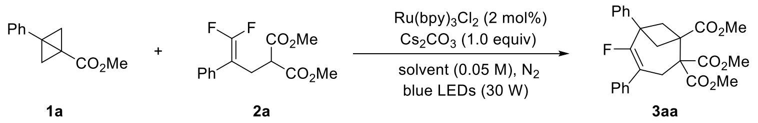

entry	solvent	yield[a] (%)
**1**	**CH_3CN**	**72**
2	acetone	58
3	1,4-dioxane	52
4	THF	36
5	toluene	65
6	DCE	37

[a]Reaction conditions: **1a** (0.2 mmol, 1.0 equiv), **2a** (0.24 mmol, 1.2 equiv), $Ru(bpy)_3Cl_2$ (2 mol%) , Cs_2CO_3 (0.2 mmol, 1.0 equiv), in solvent (0.05 M), blue LEDs (30 W), rt, for 12 h, under N_2. Yields were determined by ^1H NMR using 1,3,5-trimethoxybenzene as an internal standard.

Base

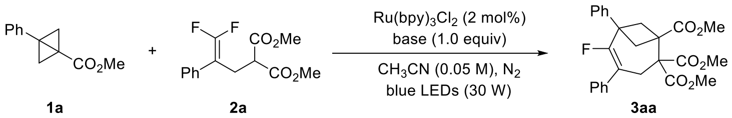

entry	base	yield[a] (%)
**1**	**Cs_2CO_3**	**72**
2	K_2CO_3	36
3	K_3PO_4	34
4	$NaHCO_3$	trace
5	DMAP	trace
6	Et_3N	trace

[a]Reaction conditions: **1a** (0.2 mmol, 1.0 equiv), **2a** (0.24 mmol, 1.2 equiv), $Ru(bpy)_3Cl_2$ (2 mol%) , base (0.2 mmol, 1.0 equiv), in CH_3CN (0.05 M), blue LEDs (30 W), rt, for 12 h, under N_2. Yields were determined by ^1H NMR using 1,3,5-trimethoxybenzene as an internal standard.

Ratio of 1a and 2a

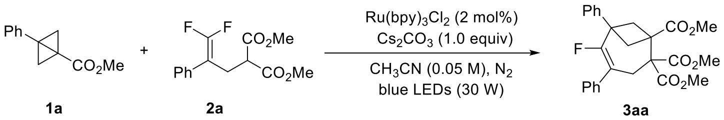

entry	**1a** : **2a**	yield[a] (%)
1	1 : 1	63
2	1 : 1.2	72 (75)[b]
**3**[c]	**1 : 1.5**	**80**
4	1.2 : 1	72
5	1.5 : 1	60
6	2 : 1	55

[a]Reaction conditions: **1a, 2a**, $Ru(bpy)_3Cl_2$ (2 mol%), Cs_2CO_3 (0.2 mmol, 1.0 equiv), in CH_3CN (0.05 M), blue LEDs (30 W), rt, for 12 h, under N_2. Yields were determined by ^1H NMR using 1,3,5-trimethoxybenzene as an internal standard. [b]Cs_2CO_3 (0.24 mmol, 1.2 equiv), [c] Cs_2CO_3 (0.3 mmol, 1.5 equiv).

Time

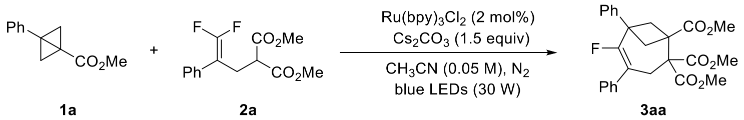

entry	time (h)	yield[a] (%)
1	12	80
**2**	**18**	**88**
3	24	87

[a]Reaction conditions: **1a** (0.2 mmol, 1.0 equiv), **2a** (0.3 mmol, 1.5 equiv), Ru(bpy)$_3$Cl$_2$ (2 mol%) , Cs_2CO_3 (0.3 mmol, 1.5 equiv), in CH_3CN (0.05 M), blue LEDs (30 W), rt, for x h, under N_2. Yields were determined by ^{1}H NMR using 1,3,5-trimethoxybenzene as an internal standard.

Light Source

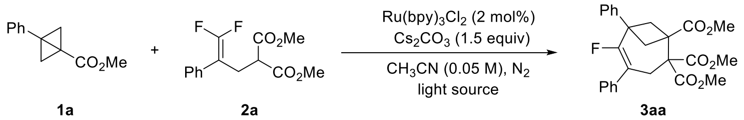

entry	light source	yield[a] (%)
**1**	**blue LEDs (30 W, 460-465 nm)**	**88**
2	purple LEDs (30 W, 390 nm)	80
3	white light (15 W, 390-780 nm)	36

[a]Reaction conditions: **1a** (0.2 mmol, 1.0 equiv), **2a** (0.3 mmol, 1.5 equiv), Ru(bpy)$_3$Cl$_2$ (2 mol%) , Cs_2CO_3 (0.3 mmol, 1.5 equiv), in CH_3CN (0.05 M), light source (x W), rt, for 18 h, under N_2. Yields were determined by ^{1}H NMR using 1,3,5-trimethoxybenzene as an internal standard.

Wattage of Blue LEDs

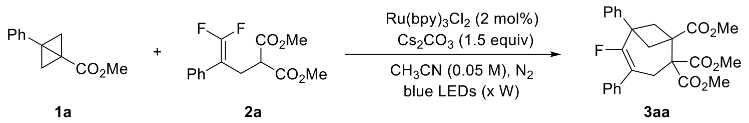

entry	blue LEDs (x W)	yield[a] (%)
1	5	60
2	10	72
3	20	75
**4**	**30**	**88**
5	45	89

[a]Reaction conditions: **1a** (0.2 mmol, 1.0 equiv), **2a** (0.3 mmol, 1.5 equiv), $Ru(bpy)_3Cl_2$ (2 mol%) , Cs_2CO_3 (0.3 mmol, 1.5 equiv), in CH_3CN (0.05 M), blue LEDs (x W), rt, for 18 h, under N_2. Yields were determined by ^1H NMR using 1,3,5-trimethoxybenzene as an internal standard.

Ratio of Ru(bpy)$_3$Cl$_2$

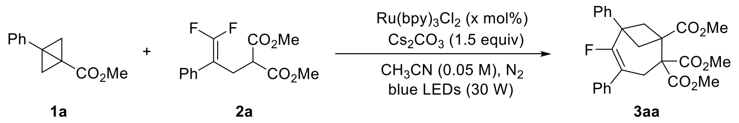

entry	x	yield[a] (%)
**1**	**1**	**89 (86)[b]**
2	2	88

[a]Reaction conditions: **1a** (0.2 mmol, 1.0 equiv), **2a** (0.3 mmol, 1.5 equiv), $Ru(bpy)_3Cl_2$ (x mol%) , Cs_2CO_3 (0.2 mmol, 1.5 equiv), in CH_3CN (0.05 M), blue LEDs (30 W), rt, for 18 h, under N_2. Yields were determined by ^1H NMR using 1,3,5-trimethoxybenzene as an internal standard. [b]Isolated yield.

Controlled Experiments

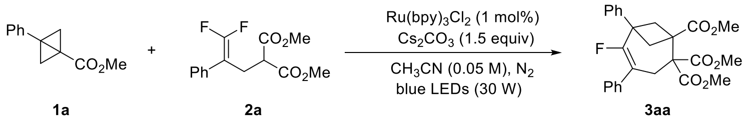

entry	deviation from standard conditions	yield[a] (%)
**1**	**Standard conditions**	**89 (86)**[b]
2	Without $Ru(bpy)_3Cl_2$	0
3	Without Cs_2CO_3	0
4	Without light	0
5	Under air atmosphere	0

[a]Reaction conditions: **1a** (0.2 mmol, 1.0 equiv), **2a** (0.3 mmol, 1.5 equiv), $Ru(bpy)_3Cl_2$ (1 mol%) , Cs_2CO_3 (0.3 mmol, 1.5 equiv), in CH_3CN (0.05 M), blue LEDs (30 W), rt, for 18 h, under N_2. Yields were determined by ^1H NMR using 1,3,5-trimethoxybenzene as an internal standard. [b]Isolated yield.

5. Representative Procedure for the Reaction of BCBs 1 and *gem*-Difluoroalkenes 2

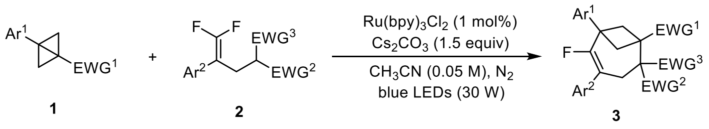

A 10 mL oven-dried Schlenk tube equipped with a magnetic stirrer was charged with BCBs **1** (0.2 mmol, 1.0 equiv), *gem*-difluoroalkenes **2** (0.3 mmol, 1.5 equiv), Cs_2CO_3 (0.3 mmol, 1.5 equiv) and $Ru(bpy)_3Cl_2$ (1 mol%). Then the tube was evacuated and backfilled with nitrogen for three times, dry solvent (4 mL) was added by a syringe. The reaction mixture was then irradiated with 30 W blue LEDs at room temperature for 12 h. After that, the solvent was removed in vacuo. The final product **3** was obtained by silica gel column chromatography using petroleum ether/EtOAc as eluent (petroleum ether/EtOAc = 5/1 to 3/1).

6. Scale-Up Synthesis

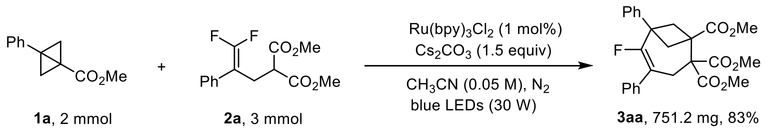

A 100 mL oven-dried Schlenk tube equipped with a magnetic stirrer was charged with BCB **1a** (376.5 mg, 2 mmol, 1.0 equiv), *gem*-difluoroalkene **2a** (852.8 mg, 3 mmol, 1.5 equiv), Cs_2CO_3 (977.4 mg, 3 mmol, 1.5 equiv) and $Ru(bpy)_3Cl_2$ (12.8 mg, 1 mol%). Then the tube was evacuated and backfilled with nitrogen for three times., dry solvent (40 mL) was added by a syringe. The reaction mixture was then irradiated with 30 W blue LEDs at room temperature for 24 h. After that, the solvent was removed in vacuo. The final product was obtained by silica gel column chromatography using petroleum ether/EtOAc as eluent (petroleum ether/EtOAc = 5/1 to 3/1) to afford the desired product **3aa** (83%, 751.2 mg).

7. Derivatizations of Products

7.1 Synthesis of the Compound 4aa[7]

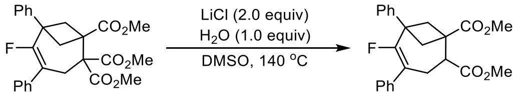

3aa 4aa, 76%

To a suspension of **3aa** (90.4 mg, 0.2 mmol, 1.0 equiv) in DMSO (1.0 mL) were added LiCl (17.0 mg, 0.4 mmol, 2.0 equiv) and H_2O (3.6 μL, 0.2 mmol, 1.0 equiv). The reaction mixture was heated to 140 ℃ and stirred for 4 h. Water (5.0 mL) was added and the resultant mixture was extracted with EtOAc for three times. The combined organic phase was dried over anhydrous Na_2SO_4, filtered and concentrated under reduced pressure. The residue was purified by silica gel column chromatography (petroleum ether/EtOAc = 5/1) to afford product **4aa** (60.0 mg, 76%) as colorless oil.

7.2 Synthesis of the Compound 5aa[8]

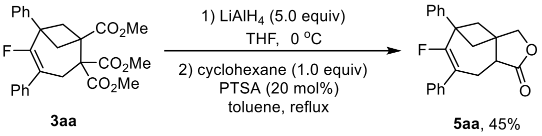

3aa 5aa, 45%

To a suspension of **3aa** (116.5 mg, 0.3 mmol, 1.0 equiv) in THF (2.0 mL) at 0 ℃ was added $LiAlH_4$ (57.0 mg, 1.5 mmol, 5.0 equiv). The resulting mixture was stirred at room temperature for 1 h, cool to 0 ℃, and quenched successively with H_2O (60.0 μL), 15% aqueous NaOH (60.0 μL) and H_2O (180.0 μL). The mixture was stirred for 30 min, then anhydrous Na_2SO_4 was added. After stirring for another 30 min, the mixture was filtered and concentrated under reduced pressure to give crude product, which was directly used in the next step without further purification.

To a suspension of crude product in toluene (2.0 mL) was added cyclohexanone (31.1 μL, 0.3 mmol, 1.0 equiv), *p*-toluenesulfonic acid (5.2 mg, 0.03 mmol, 0.1 equiv). The reaction mixture was refluxed for 8 h, then the solvent was removed under reduced pressure. The residue was purified by silica gel column chromatography (petroleum ether/EtOAc = 10/1) to afford product **5aa** (45.1 mg, 45%) as colorless oil.

7.3 Synthesis of the Compound 6ka and 6ka’[9]

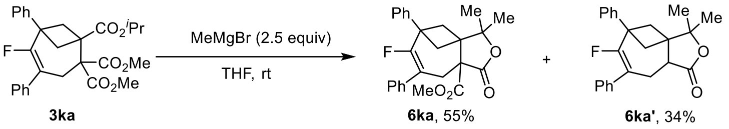

To an oven-dried 10 mL Schlenk tube equipped with a Teflon coated magnetic stir bar was added **3ka** (96.0 mg, 0.2 mmol, 1.0 equiv). The tube was evacuated and backfilled with nitrogen (3 times). Subsequently, THF (2 mL) was added. A solution of methylmagnesium bromide in THF (0.5 mmol, 2.5 equiv, 3.0 M) was added dropwise into the reaction vessel under nitrogen. After stirring at room temperature for 3 h, the reaction was quenched with saturated NH_4Cl aqueous solution. The aqueous layer was extracted with ethyl acetate, the combined organic layer was dried over Na_2SO_4, filtered and concentrated under the reduced pressure, the mixture was purified by silica gel column chromatography (petroleum ether/EtOAc = 5/1) to afford product **6ka** (46.2 mg, 55%) as colorless oil and **6ka’** (24.7 mg, 34%) as a white solid.

7.4 Synthesis of the Compound 7la[10]

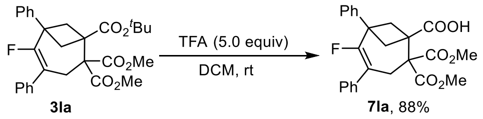

Compound **3la** (98.9 mg, 0.2 mmol, 1.0 equiv) was dissolved in anhydrous DCM (2 mL) and was treated with TFA (77.0 μL, 1.0 mmol, 5.0 equiv) at 0 ℃. The reaction mixture was stirred at room temperature overnight. After that, the reaction mixture was cooled in ice-water bath and was quenched by saturated aqueous $NaHCO_3$ and was extracted with DCM. The combined organic layer was dried over anhydrous Na_2SO_4, followed by filtration and evaporation of the solvent to give product **7la** (87.7 mg, 88%) as a white solid.

7.5 Synthesis of the Compound 8la[11]

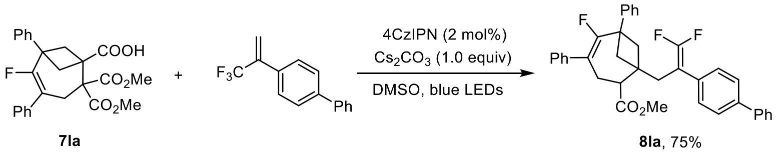

A 10 mL oven-dried Schlenk-tube equipped with a magnetic stirrer was charged with Compound **7la** (43.8 mg, 0.1 mmol, 1.0 equiv), α-CF_3 alkenes (49.7 mg, 0.2 mmol, 2.0 equiv),

4CzIPN (1.58 mg, 2 mol%), Cs_2CO_3 (65.2 mg, 0.2 mmol, 2.0 equiv). Then, the tube was evacuated and backfilled with nitrogen (three times). Subsequently, dry DMSO (1.0 mL) was added by a syringe. The reaction mixture was stirred under the irradiation of a 30 W Blue LED (λ = 455-460 nm) for 18 h. After that, the resulting mixture was quenched with H_2O and extracted with EtOAc (3 × 10 mL). The combined organic layer was dried over anhydrous Na_2SO_4 and concentrated under reduced pressure. The crude product was purified by silica gel column chromatography (petroleum ether/EtOAc = 10:1 to 5:1) to afford product **8la** (42.3 mg, 75%) as a white solid.

7.6 Synthesis of the Compound 9la[12]

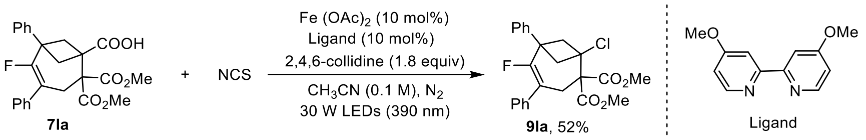

An oven-dried 10 mL Schlenk tube equipped with a magnetic stir bar was charged with **7la** (43.8 mg, 0.1 mmol, 1.0 equiv.), ligand (10 mol%, 8.6 mg), NCS (*N*-chlorosuccinimide) (28.0 mg, 0.21 mmol, 2.1 equiv.). Then the Schlenk-tube was transferred to nitrogen-filled glovebox to add $Fe(OAc)_2$ (6.9 mg, 10 mol%,) and anhydride MeCN (1 mL). After that, the Schlenk-tube was sealed with rubber plug and taken out of the glovebox. The reaction mixture was added 2,4,6-collidine (24 μl, 0.18 mmol, 1.8 equiv.) via syringe. Finally, the reaction mixture was stirred and irradiated for 12 h under LEDs (30 W, 390 nm), cooling with a fan. Then the solvent was removed under reduced pressure. The residue was purified by silica gel column chromatography (petroleum ether/EtOAc = 10/1) to afford product **9la** (22.3 mg, 52%) as colorless oil.

7.7 Synthesis of the Compound 10la[12]

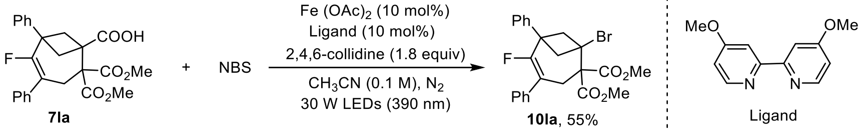

An oven-dried 10 mL Schlenk tube equipped with a magnetic stir bar was charged with **7la** (43.8 mg, 0.1 mmol, 1.0 equiv.), ligand (10 mol%, 8.6 mg), NBS (*N*-Bromosuccinimide) (37.4 mg, 0.21 mmol, 2.1 equiv.). Then the Schlenk-tube was transferred to nitrogen-filled glovebox to add $Fe(OAc)_2$ (6.9 mg, 10 mol%,) and anhydride MeCN (1 mL). After that, the Schlenk tube was

sealed with rubber plug and taken out of the glovebox. The reaction mixture was added 2,4,6-collidine (24 μl, 0.18 mmol, 1.8 equiv.) via syringe. Finally, the reaction mixture was stirred and irradiated for 12 h under LEDs (30 W, 390 nm), cooling with a fan. Then the solvent was removed under reduced pressure. The residue was purified by silica gel column chromatography (petroleum ether/EtOAc = 10/1) to afford product **10la** (26.1 mg, 55%) as colorless oil.

8 Mechanistic Investigation

8.1 TEMPO-Trapping Experiment

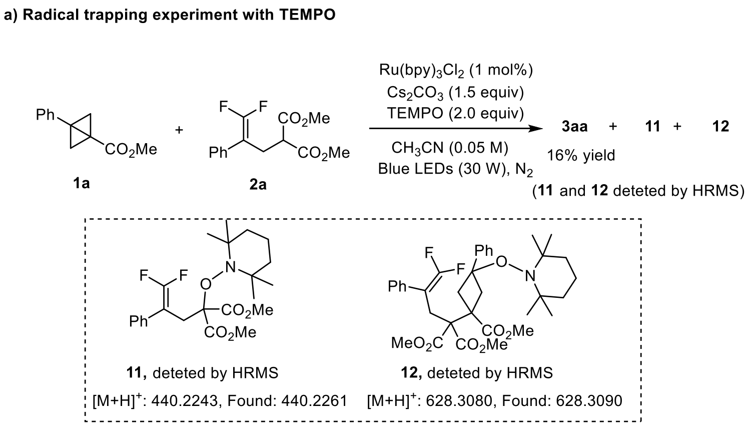

A 10 mL oven-dried Schlenk tube equipped with a magnetic stirrer was charged with BCB **1a** (0.2 mmol, 1.0 equiv), *gem*-difluoroalkene **2a** (0.3 mmol, 1.5 equiv), Cs_2CO_3 (0.3 mmol, 1.5 equiv), $Ru(bpy)_3Cl_2$ (1 mol%) and TEMPO (0.4 mmol, 2.0 equiv). Then the tube was evacuated and backfilled with nitrogen for three times., dry solvent (4 mL) was added by a syringe. The reaction mixture was then irradiated with 30 W blue LEDs at room temperature for 12 h. After that, a 16% yield of **3aa** was obtained, along with the TEMPO adduct **11** and TEMPO adduct **12** detected by HRMS. This result indicated that a radical pathway might be involved in this transformation.

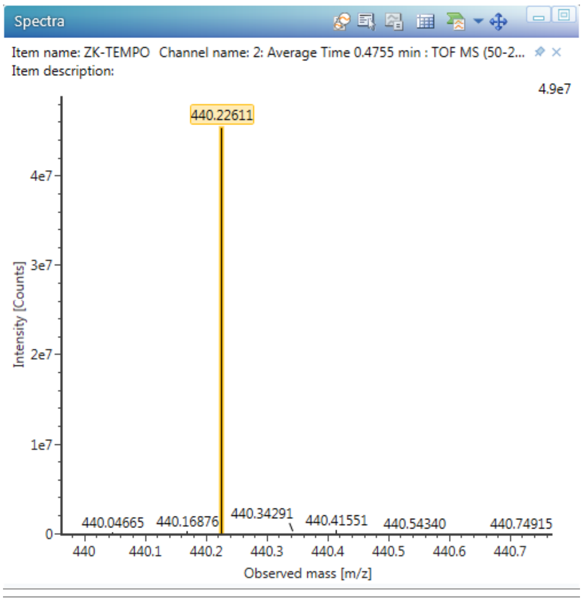

Figure S1. TEMPO adduct **11** detected by HRMS

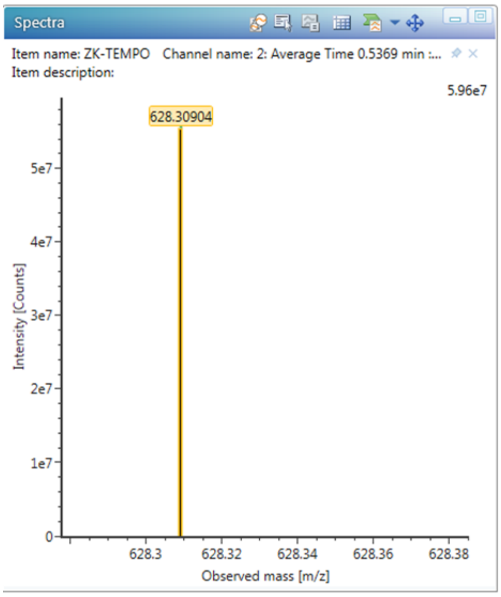

Figure S2. TEMPO adduct **12** detected by HRMS

8.2 Stern-Volmer Fluorescence Quenching Experiments

To a solution of 4CzIPN in anhydrous, N_2-saturated DMSO (5.0×10^{-4} mol/L) in a quartz cuvette, different amounts of BCB **1a**, and *gem*-difluoroalkene **2a**, Cs_2CO_3, *gem*-difluoroalkene **2a** + Cs_2CO_3 were added, respectively, and the resulting changes in fluorescence intensity were collected. The emission intensity at 525 nm was collected with excited wavelength of 470 nm. The results are shown in Figure S3, S4, S5 and S6.

According to the results as well as the corresponding Stern-Volmer plots (Figure S3, S4, S5 and S6), the BCB **1a**, **2a** and Cs_2CO_3 did not show an obvious quenching effect to the fluorescence intensity of 4CzIPN. While the *gem*-difluoroalkene **2a** + Cs_2CO_3 showed an obvious quenching to the fluorescence intensity of 4CzIPN.

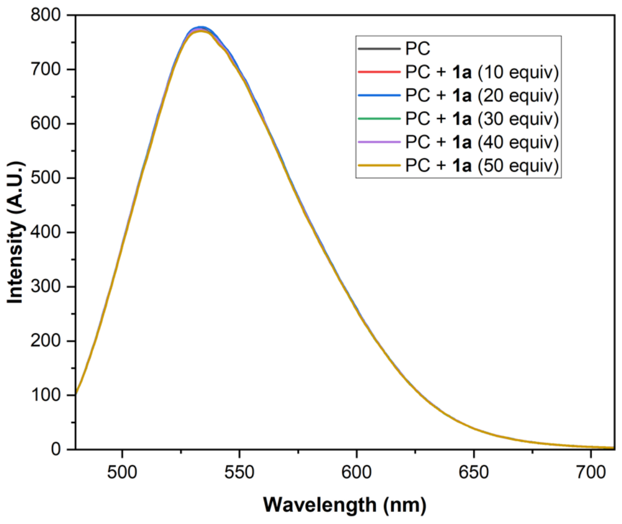

Figure S3 Fluorescence quenching with **1a**

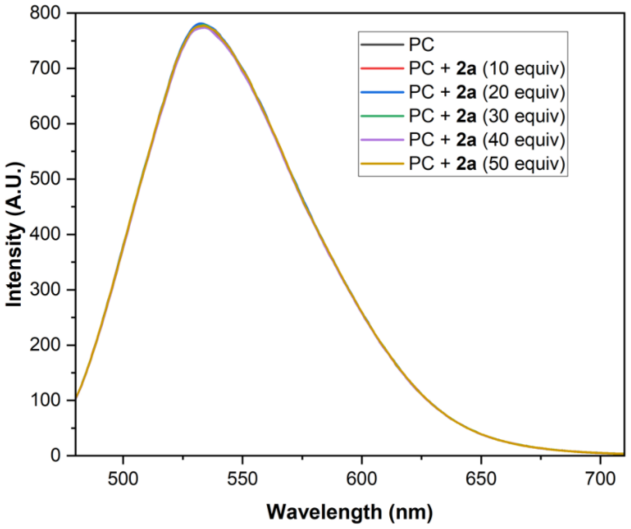

Figure S4 Fluorescence quenching with **2a**

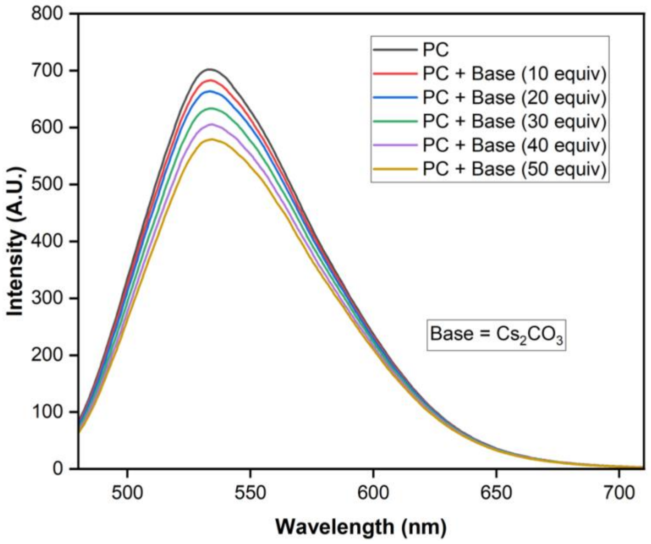

Figure S5 Fluorescence quenching with **Cs_2CO_3**

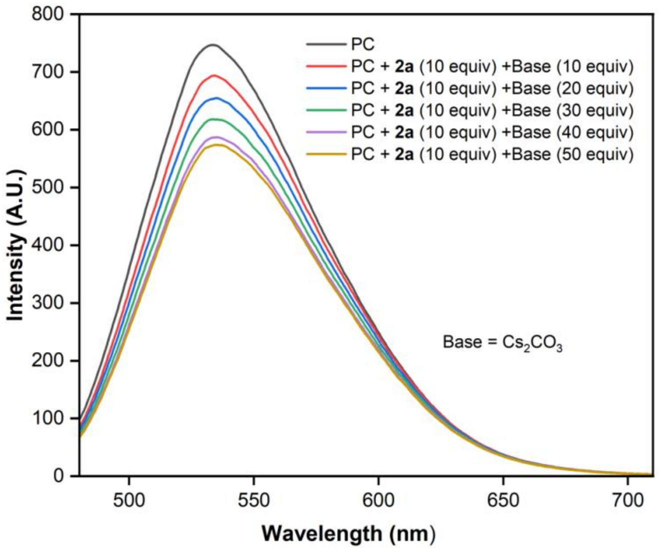

Figure S6 Fluorescence quenching with **2a** + **Cs_2CO_3**

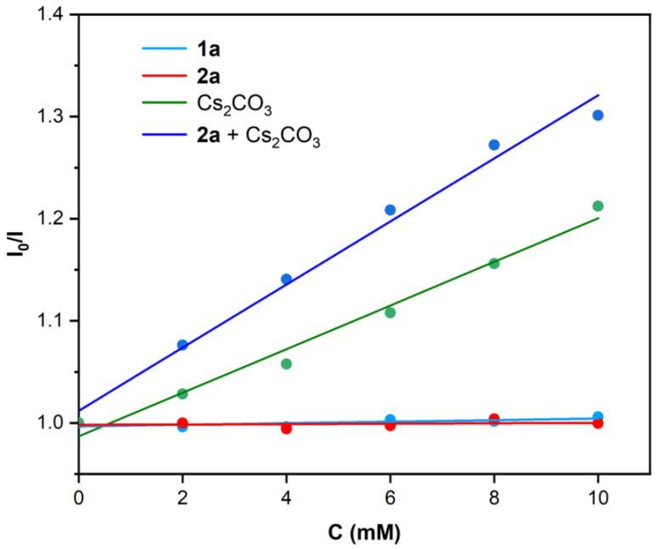

Figure S7 Stern-Volmer Plots of **1a**, **2a**, Cs_2CO_3 and **2a** + Cs_2CO_3

8.3 Light On-Off Experiments

To further examine the impact of light, we conducted experiments under alternating periods of irradiation and darkness. The yield of **3aa** was determined by crude ^{1}H NMR spectra using 1,3,5-trimethoxybenzene as an internal standard. The results are shown in **Figure S4**.

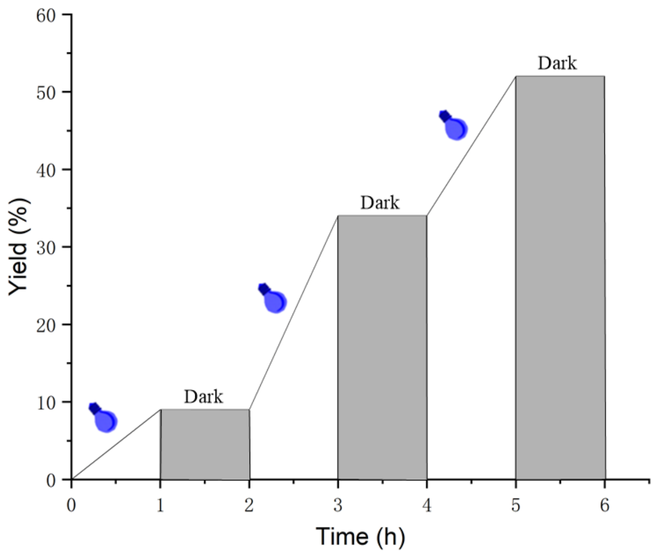

Figure S8. Light On-Off Experiments

The results of light on-off experiments showed that the reaction proceed only during the visible light irradiation, suggesting that the reaction maybe proceed by a catalytic process rather than by a radical chain process.

8.4 Cyclic voltammetry (CV) measurements

Cyclic voltammetry was conducted on an Electrochemical Workstation using a 3-electrode cell configuration. A glassy carbon working electrode was employed alongside a platinum wire counter electrode and a Ag/AgCl reference electrode. CH_3CN was degassed by bubbling N_2 prior to measurements. 0.01 M solutions of **1a** or **2a** in CH_3CN were freshly prepared along with 0.1 M of *n*-Bu_4NPF_6 as supporting electrolyte and were examined at a scan 100 mV/s. The results of cyclic voltammetry experiments indicated that the oxidation potential of BCB **1a** ($E_{1/2}^{ox}$ = +1.4 V vs SCE) inaccessible for the $Ru(bpy)_3Cl_2$ excited state ($E_{1/2}^{ox}$ = +0.77 V vs SCE). The oxidation potential of compound **2a** can be effectively reduced from $E_{1/2}^{ox}$ = 0.69 V (vs SCE) to $E_{1/2}^{ox}$ = 0.56 V (vs SCE) in the presence of base. which is considered the oxidation of **2a** by the $Ru(bpy)_3Cl_2$ is thermodynamically feasible.

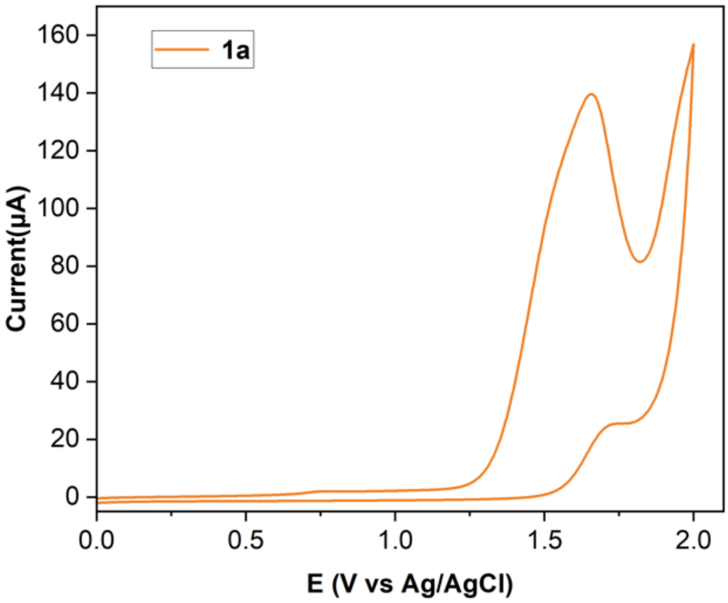

Figure S9. Cyclic voltammogram of **1a** in CH_3CN.

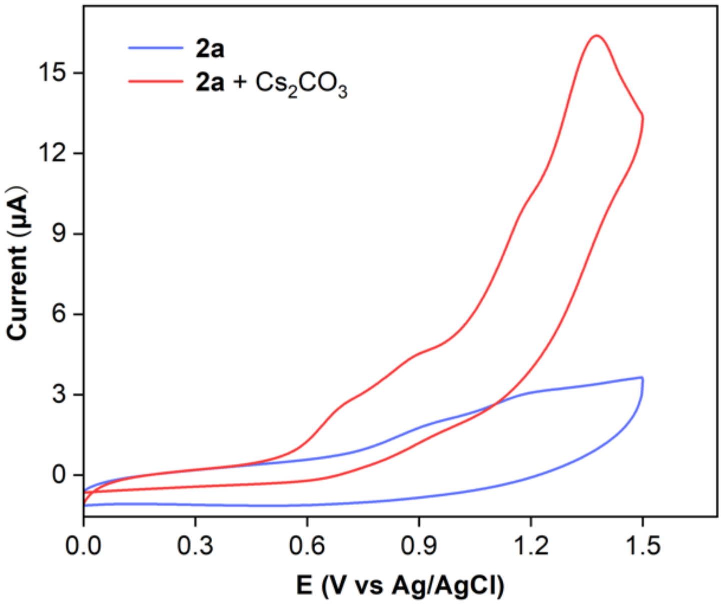

Figure S10. Cyclic voltammogram of **2a** and **2a** + Cs_2CO_3 in CH_3CN.

9 Characterization Date of Products

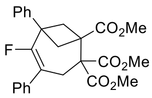

Trimethyl 5-fluoro-4,6-diphenylbicyclo[4.1.1]oct-4-ene-1,2,2-tricarboxylate (**3aa**): White solid (86%, 77.8 mg); m.p.: 104-105 ^{o}C; R$_{f}$ = 0.3 (petroleum ether/EtOAc = 3/1); ^{1}H NMR (400 MHz, CDCl$_3$): δ = 7.49-7.46 (m, 2H), 7.41-7.37 (m, 2H), 7.34-7.29 (m, 3H), 7.25-7.20 (m, 3H), 3.81 (s, 6H), 3.71 (s, 3H), 3.58 (d, *J* = 4.8 Hz, 2H), 3.38-3.34 (m, 2H), 3.09-3.05 (m, 2H); ^{19}F NMR (376 MHz, CDCl$_3$): δ = -98.26 (s); ^{13}C NMR (100 MHz, CDCl$_3$): δ = 173.4, 171.2, 160.1 (d, *J* = 258.8 Hz), 143.3 (d, *J* = 4.8 Hz), 138.2, 128.6 (d, *J* = 3.6 Hz), 128.2, 128.0, 126.9, 126.7, 126.1, 112.9 (d, *J* = 18.0 Hz), 62.7, 52.8, 52.7, 48.6, 45.0 (d, *J* = 27.7 Hz), 41.4, 36.5 (d, *J* = 4.4 Hz) ppm; HRMS (ESI) calcd for $C_{26}H_{26}FO_6$ [M+H]$^+$ 453.1708, found 453.1704.

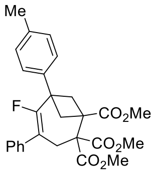

Trimethyl 5-fluoro-4-phenyl-6-(*p*-tolyl)bicyclo[4.1.1]oct-4-ene-1,2,2-tricarboxylate (**3ba**): White solid (95%, 88.5 mg); m.p.: 112-113 ^{o}C; R$_{f}$ = 0.3 (petroleum ether/EtOAc = 3/1); ^{1}H NMR (400 MHz, CDCl$_3$): δ = 7.51-7.48 (m, 2H), 7.35-7.31 (m, 2H), 7.27-7.20 (m, 3H), 7.14-7.10 (m, 2H), 3.82 (s, 6H), 3.72 (s, 3H), 3.58 (d, *J* = 4.4 Hz, 2H), 3.38-3.33 (m, 2H), 3.08-3.04 (m, 2H), 2.38 (s, 3H); ^{19}F NMR (376 MHz, CDCl$_3$): δ = -97.55 (s); ^{13}C NMR (100 MHz, CDCl$_3$): δ = 173.4, 171.2, 160.2 (d, *J* = 258.9 Hz), 140.4 (d, *J* = 5.2 Hz), 138.2, 136.3, 128.8, 128.6 (d, *J* = 3.3 Hz), 127.9, 126.9, 126.0, 112.7 (d, *J* = 17.9 Hz), 62.7, 52.7, 52.6, 44.7 (d, *J* = 27.7 Hz), 44.5, 41.4, 36.5 (d, *J* = 4.5 Hz), 21.0 ppm; HRMS (ESI) calcd for $C_{27}H_{27}NaFO_6$ [M+Na]$^+$ 489.1684, found 489.1673.

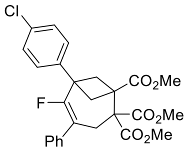

Trimethyl 6-(4-chlorophenyl)-5-fluoro-4-phenylbicyclo[4.1.1]oct-4-ene-1,2,2-tricarboxylate (**3ca**): White solid (86%, 79.5 mg); m.p.: 127-128 ^{o}C; R$_{f}$ = 0.3 (petroleum ether/EtOAc = 3/1); ^{1}H NMR (400 MHz, CDCl$_3$): δ = 7.46-7.43 (m, 2H), 7.36-7.30 (m, 4H), 7.25-7.20 (m, 1H), 7.14-7.11 (m, 2H), 3.80 (s, 6H), 3.72 (s, 3H), 3.55 (d, *J* = 4.8 Hz, 2H), 3.31-3.27 (m, 2H), 3.06-3.02 (m, 2H); ^{19}F NMR (376 MHz,

$CDCl_3$): δ = -98.70 (s); ^{13}C NMR (100 MHz, $CDCl_3$): δ = 173.2, 171.1, 159.6 (d, *J* = 258.7 Hz), 141.8 (d, *J* = 4.8 Hz), 138.0, 132.6, 128.6 (d, *J* = 3.6 Hz), 128.4, 128.0, 127.6, 127.1, 113.2 (d, *J* = 17.3 Hz), 62.6, 52.9, 52.8, 48.5, 44.7 (d, *J* = 27.7 Hz), 41.4, 36.5 (d, *J* = 4.7 Hz) ppm; HRMS (ESI) calcd for $C_{26}H_{25}ClFO_6$ $[M+H]^+$ 487.1318, found 487.1322.

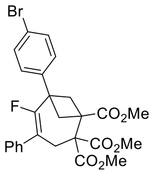

Trimethyl 6-(4-bromophenyl)-5-fluoro-4-phenylbicyclo[4.1.1]oct-4-ene-1,2,2-tricarboxylate (**3da**): White solid (77%, 81.7 mg); m.p.: 147-148 °C; R_f = 0.3 (petroleum ether/EtOAc = 3/1); ^{1}H NMR (400 MHz, $CDCl_3$): δ = 7.51-7.48 (m, 2H), 7.46-7.43 (m, 2H), 7.34-7.30 (m, 2H), 7.25-7.21 (m, 1H), 7.09-7.05 (m, 2H), 3.80 (s, 6H), 3.71 (s, 3H), 3.55 (d, *J* = 4.8 Hz, 2H), 3.30-3.26 (m, 2H), 3.05-3.01 (m, 2H); ^{19}F NMR (376 MHz, $CDCl_3$): δ = -98.68 (s); ^{13}C NMR (100 MHz, $CDCl_3$): δ = 173.2, 171.1, 159.5 (d, *J* = 258.8 Hz), 142.3 (d, *J* = 5.5 Hz), 138.0, 131.3, 128.6 (d, *J* = 3.1 Hz), 128.0, 127.1, 120.7, 113.3 (d, *J* = 17.5 Hz), 62.6, 52.9, 52.8, 48.5, 44.7 (d, *J* = 27.7 Hz), 41.4, 36.6 (d, *J* = 4.6 Hz) ppm; HRMS (ESI) calcd for $C_{26}H_{25}BrFO_6$ $[M+H]^+$ 531.0813, found 531.0832.

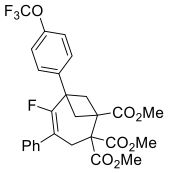

Trimethyl 5-fluoro-4-phenyl-6-(4-(trifluoromethoxy)phenyl)bicyclo[4.1.1]oct-4-ene-1,2,2-tricarboxylate (**3ea**): Colorless oil (97%, 104.1 mg); R_f = 0.3 (petroleum ether/EtOAc = 3/1); ^{1}H NMR (400 MHz, $CDCl_3$): δ = 7.46-7.43 (m, 2H), 7.34-7.30 (m, 2H), 7.25-7.23 (m, 1H), 7.21 (s, 4H), 3.80 (s, 6H), 3.72 (s, 3H), 3.56 (d, *J* = 4.8 Hz, 2H), 3.33-3.29 (m, 2H), 3.08-3.03 (m, 2H); ^{19}F NMR (376 MHz, $CDCl_3$): δ = -57.71 (s), -97.94 (s); ^{13}C NMR (100 MHz, $CDCl_3$): δ = 173.3, 171.1, 159.5 (d, *J* = 258.8 Hz), 147.9, 141.9 (d, *J* = 5.2 Hz), 138.1, 128.6 (d, *J* = 3.1 Hz), 128.1, 127.7, 127.1, 120.8, 120.4 (q, *J* = 255.7 Hz), 113.3 (d, *J* = 17.6 Hz), 62.7, 52.9, 52.8, 48.5, 44.7 (d, *J* = 27.7 Hz), 41.4, 36.6 (d, *J* = 4.5 Hz) ppm; HRMS (ESI) calcd for $C_{27}H_{25}F_4O_7$ $[M+H]^+$ 537.1531, found 537.1539.

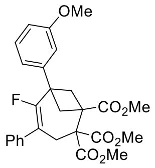

Trimethyl 5-fluoro-6-(3-methoxyphenyl)-4-phenylbicyclo[4.1.1]oct-4-ene-1,2,2-tricarboxylate (**3fa**): Colorless oil (87%, 84.3 mg); R_f = 0.3 (petroleum ether/EtOAc = 3/1); ^{1}H NMR (400 MHz, $CDCl_3$): δ = 7.49-7.45 (m, 2H), 7.34-7.29 (m, 3H), 7.25-7.20 (m, 1H), 6.84-6.73 (m, 3H), 3.82-3.80 (m, 9H), 3.71 (s, 3H), 3.57 (d, *J* = 4.8 Hz, 2H), 3.36-3.33 (m, 2H), 3.05-3.02 (m, 2H); ^{19}F NMR (376 MHz, $CDCl_3$): δ = -98.03 (s); ^{13}C NMR (100 MHz, $CDCl_3$): δ = 173.4, 171.2, 160.0 (d, *J* = 259.1 Hz), 159.4, 144.9 (d, *J* = 4.9 Hz), 138.2, 129.2, 128.7 (d, *J* = 3.0 Hz), 127.9, 126.9, 118.5, 112.9 (d, *J* = 17.4 Hz), 112.2, 111.9, 62.7, 55.1, 52.8, 52.7, 48.4, 45.0 (d, *J* = 27.7 Hz), 41.4, 36.5 (d, *J* = 4.5 Hz) ppm; HRMS (ESI) calcd for $C_{27}H_{28}FO_7$ $[M+H]^+$ 483.1814, found 483.1817.

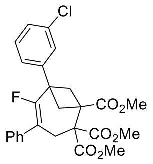

Trimethyl 6-(3-chlorophenyl)-5-fluoro-4-phenylbicyclo[4.1.1]oct-4-ene-1,2,2-tricarboxylate (**3ga**): White solid (87%, 89.5 mg); m.p.: 173-174 °C; R_f = 0.3 (petroleum ether/EtOAc = 3/1); ^{1}H NMR (400 MHz, $CDCl_3$): δ = 7.46-7.43 (m, 2H), 7.34-7.28 (m, 3H), 7.26-7.18 (m, 3H), 7.09-7.06 (m, 1H), 3.80 (s, 6H), 3.72 (s, 3H), 3.55 (d, *J* = 4.8 Hz, 2H), 3.33-3.29 (m, 2H), 3.06-3.02 (m, 2H); ^{19}F NMR (376 MHz, $CDCl_3$): δ = -98.03 (s); ^{13}C NMR (100 MHz, $CDCl_3$): δ = 173.2, 171.1, 159.4 (d, *J* = 258.8 Hz), 145.2 (d, *J* = 4.9 Hz), 138.0, 134.1, 129.5, 128.6 (d, *J* = 3.4 Hz), 128.0, 127.1, 127.0, 126.6, 124.5, 113.3 (d, *J* = 17.7 Hz), 62.6, 52.9, 52.8, 48.5, 44.8 (d, *J* = 27.4 Hz), 41.4, 36.5 (d, *J* = 4.4 Hz) ppm; HRMS (ESI) calcd for $C_{26}H_{24}ClKFO_6$ $[M+K]^+$ 525.0877, found 525.0887.

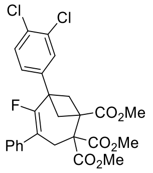

Trimethyl 6-(3,4-dichlorophenyl)-5-fluoro-4-phenylbicyclo[4.1.1]oct-4-ene-1,2,2-tricarboxylate (**3ha**): Colorless oil (78%, 81.3 mg); R_f = 0.3 (petroleum ether/EtOAc = 3/1); ^{1}H NMR (400 MHz, $CDCl_3$): δ = 7.45-7.42 (m, 3H), 7.34-7.30 (m, 2H), 7.28 (d, *J* = 2.0 Hz, 1H), 7.25-7.21 (m, 1H), 7.04-7.01 (m, 1H), 3.79 (s, 6H), 3.72 (s, 3H), 3.54 (d, *J* = 4.8 Hz, 2H), 3.29-3.26 (m, 2H), 3.05-3.01 (m,

2H); ^{19}F NMR (376 MHz, $CDCl_3$): δ = -98.30 (s); ^{13}C NMR (100 MHz, $CDCl_3$): δ = 173.1, 171.1, 159.0 (d, *J* = 258.8 Hz), 143.4 (d, *J* = 4.7 Hz), 137.9, 132.4, 130.9, 130.3, 128.6 (d, *J* = 3.4 Hz), 128.5, 128.1, 127.2, 125.8, 113.6 (d, *J* = 17.4 Hz), 62.9, 52.9, 52.8, 48.4, 44.5 (d, *J* = 27.4 Hz), 41.4, 36.6 (d, *J* = 4.6 Hz) ppm; HRMS (ESI) calcd for $C_{26}H_{24}Cl_2FO_6$ $[M+H]^+$ 521.0929, found 521.0914.

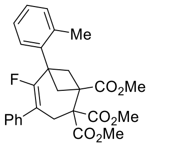

Trimethyl 5-fluoro-4-phenyl-6-(*o*-tolyl)bicyclo[4.1.1]oct-4-ene-1,2,2-tricarboxylate (**3ia**): Colorless oil (53%, 49.5 mg); R_f = 0.3 (petroleum ether/EtOAc = 3/1); ^{1}H NMR (400 MHz, $CDCl_3$): δ = 7.40-7.37 (m, 2H), 7.26-7.22 (m, 2H), 7.18-7.05 (m, 5H), 3.76 (s, 3H), 3.71 (s, 3H), 3.63-3.57 (m, 5H), 3.43-3.37 (m, 2H), 3.01 (dd, *J* = 12.4, 3.6 Hz, 1H), 2.62 (dd, *J* = 12.2, 6.4 Hz, 1H), 2.25 (s, 3H); ^{19}F NMR (376 MHz, $CDCl_3$): δ = -98.40 (s); ^{13}C NMR (100 MHz, $CDCl_3$): δ = 173.5, 172.0, 170.5, 159.8 (d, *J* = 258.2 Hz), 141.0 (d, *J* = 4.7 Hz), 138.4, 136.1, 130.6, 128.6 (d, *J* = 3.3 Hz), 128.0, 127.1, 127.0, 126.4, 125.7, 113.0 (d, *J* = 18.1 Hz), 62.8, 52.82, 52.76, 52.66, 48.4, 45.6 (d, *J* = 28.4 Hz), 42.2, 40.9, 36.5 (d, *J* = 4.7 Hz), 20.3 ppm; HRMS (ESI) calcd for $C_{27}H_{28}FO_6$ $[M+H]^+$ 467.1864, found 467.1869.

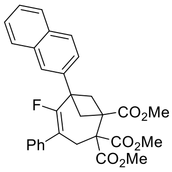

Trimethyl 5-fluoro-6-(naphthalen-2-yl)-4-phenylbicyclo[4.1.1]oct-4-ene-1,2,2-tricarboxylate (**3ja**): Colorless oil (68%, 68.3 mg); R_f = 0.3 (petroleum ether/EtOAc = 3/1); ^{1}H NMR (400 MHz, $CDCl_3$): δ = 7.87-7.83 (m, 3H), 7.66 (s, 1H), 7.51-7.47 (m, 4H), 7.34-7.30 (m, 3H), 7.25-7.21 (m, 1H), 3.83 (s, 6H), 3.70 (s, 3H), 3.61 (d, *J* = 4.8 Hz, 2H), 3.47-3.44 (m, 2H), 3.17-3.12 (m, 2H); ^{19}F NMR (376 MHz, $CDCl_3$): δ = -97.51 (s); ^{13}C NMR (100 MHz, $CDCl_3$): δ = 173.4, 171.2, 160.1 (d, *J* = 258.9 Hz), 140.7 (d, *J* = 3.8 Hz), 138.2, 133.0, 132.2, 128.7 (d, *J* = 3.3 Hz), 128.0, 127.7, 127.6, 127.0, 126.2, 125.8, 124.7, 124.6, 113.2 (d, *J* = 17.4 Hz), 62.8, 52.8, 52.7, 48.6, 45.2 (d, *J* = 27.1 Hz), 41.5, 36.6 (d, *J* = 3.2 Hz) ppm; HRMS (ESI) calcd for $C_{30}H_{28}FO_6$ $[M+H]^+$ 503.1864, found 503.1861.

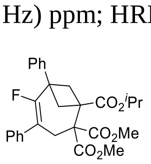

1-Isopropyl 2,2-dimethyl 5-fluoro-4,6-diphenylbicyclo[4.1.1]oct-4-ene-1,2,2-tricarboxylate (**3ka**): White solid (82%, 78.8 mg); m.p.: 131-132 °C; R_f = 0.3 (petroleum ether/EtOAc = 5/1); ^{1}H NMR (400

MHz, $CDCl_3$): δ = 7.49-7.47 (m, 2H), 7.39-7.27 (m, 5H), 7.24-7.19 (m, 3H), 5.08-4.99 (m, 1H), 3.80 (s, 6H), 3.56 (d, *J* = 4.8 Hz, 2H), 3.33-3.29 (m, 2H), 3.05-3.01 (m, 2H), 1.22 (d, *J* = 6.4 Hz, 6H); ^{19}F NMR (376 MHz, $CDCl_3$): δ = -97.53 (s); ^{13}C NMR (100 MHz, $CDCl_3$): δ = 172.6, 171.2, 160.2 (d, *J* = 259.0 Hz), 143.3 (d, *J* = 4.9 Hz), 138.3, 128.7 (d, *J* = 3.6 Hz), 128.1, 127.9, 126.9, 126.6, 126.1, 112.9 (d, *J* = 17.9 Hz), 69.1, 62.8, 52.6, 48.5, 44.9 (d, *J* = 27.5 Hz), 41.4, 36.7 (d, *J* = 4.6 Hz), 21.5 ppm; HRMS (ESI) calcd for $C_{28}H_{30}FO_6$ $[M+H]^+$ 481.2021, found 481.2030.

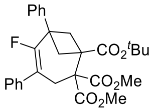

1-(*Tert*-butyl) 2,2-dimethyl 5-fluoro-4,6-diphenylbicyclo[4.1.1]oct-4-ene-1,2,2-tricarboxylate (**3la**): White solid (91%, 90.1 mg); m.p.: 129-130 °C; R_f = 0.5 (petroleum ether/EtOAc = 3/1); ^{1}H NMR (400 MHz, $CDCl_3$): δ = 7.48-7.46 (m, 2H), 7.38-7.26 (m, 5H), 7.23-7.18 (m, 3H), 3.79 (s, 6H), 3.55 (d, *J* = 4.8 Hz, 2H), 3.29-3.25 (m, 2H), 3.03-2.98 (m, 2H), 1.43 (s, 9H); ^{19}F NMR (376 MHz, $CDCl_3$): δ = -97.64 (s); ^{13}C NMR (100 MHz, $CDCl_3$): δ = 172.3, 171.2, 160.4 (d, *J* = 259.0 Hz), 143.4 (d, *J* = 4.9 Hz), 138.4, 128.7 (d, *J* = 3.5 Hz), 128.1, 127.9, 126.9, 126.6, 126.1, 112.9 (d, *J* = 17.9 Hz), 81.7, 62.7, 52.5, 49.0, 44.8 (d, *J* = 27.0 Hz), 41.5, 36.9 (d, *J* = 4.5 Hz), 27.7 ppm; HRMS (ESI) calcd for $C_{29}H_{31}KFO_6$ $[M+K]^+$ 533.1736, found 533.1746.

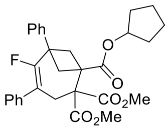

1-Cyclopentyl 2,2-dimethyl 5-fluoro-4,6-diphenylbicyclo[4.1.1]oct-4-ene-1,2,2-tricarboxylate (**3ma**): White solid (87%, 88.2 mg); m.p.: 121-122 °C; R_f = 0.3 (petroleum ether/EtOAc = 3/1); ^{1}H NMR (400 MHz, $CDCl_3$): δ = 7.53-7.50 (m, 2H), 7.43-7.28 (m, 6H), 7.25-7.23 (m, 2H), 5.24-5.21 (m, 1H), 3.84 (s, 6H), 3.60 (d, *J* = 4.8 Hz, 2H), 3.35-3.31 (m, 2H), 3.09-3.04 (m, 2H), 1.91-1.85 (m, 2H), 1.73-1.60 (m, 6H); ^{19}F NMR (376 MHz, $CDCl_3$): δ = -98.21 (s); ^{13}C NMR (100 MHz, $CDCl_3$): δ = 172.9, 171.1, 160.3 (d, *J* = 258.9 Hz), 143.3, (d, *J* = 4.5 Hz), 138.3, 128.7 (d, *J* = 3.1 Hz), 128.2, 128.0, 126.9, 126.6, 126.0, 113.0 (d, *J* = 17.9 Hz), 78.5, 62.7, 52.6, 48.4, 44.9 (d, *J* = 27.5 Hz), 41.4, 36.8 (d, *J* = 4.1 Hz), 32.3, 23.6 ppm; HRMS (ESI) calcd for $C_{30}H_{32}FO_6$ $[M+H]^+$ 507.2177, found 507.2182.

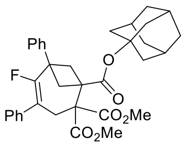

1-(Adamantan-1-yl) 2,2-dimethyl 5-fluoro-4,6-diphenylbicyclo[4.1.1]oct-4-ene-1,2,2-tricarboxylate (**3na**): White solid (84%, 96.2 mg); m.p.: 170-171 °C; R_f = 0.5 (petroleum ether/EtOAc = 3/1); ^{1}H NMR (400 MHz, $CDCl_3$): δ = 7.48-7.45 (m, 2H), 7.37-7.26 (m, 5H), 7.23-7.17 (m, 3H), 3.80 (s, 6H), 3.52 (d, *J* = 5.2 Hz, 2H), 3.29-3.25 (m, 2H), 3.02-2.97 (m, 2H), 2.15 (s, 3H), 2.08 (s, 6H), 1.64 (s, 6H); ^{19}F NMR (376 MHz, $CDCl_3$): δ = -97.66 (s); ^{13}C NMR (100 MHz, $CDCl_3$): δ = 172.0, 171.2, 160.4 (d, *J* = 259.5 Hz), 143.4 (d, *J* = 5.0 Hz), 138.4, 128.7 (d, *J* = 3.6 Hz), 128.1, 127.9, 126.9, 126.5, 126.1, 112.9 (d, *J* = 18.0 Hz), 81.8, 62.7, 52.6, 49.1, 44.8 (d, *J* = 27.0 Hz), 41.6, 40.9, 36.8 (d, *J* = 4.7 Hz), 36.0, 30.7 ppm; HRMS (ESI) calcd for $C_{35}H_{38}FO_6$ $[M+H]^+$ 573.2647, found 573.2651.

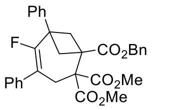

1-Benzyl 2,2-dimethyl 5-fluoro-4,6-diphenylbicyclo[4.1.1]oct-4-ene-1,2,2-tricarboxylate (**3oa**): Colorless oil (94%, 95.5 mg); R_f = 0.3 (petroleum ether/EtOAc = 3/1); ^{1}H NMR (400 MHz, $CDCl_3$): δ = 7.51-7.49 (m, 2H), 7.41-7.29 (m, 10H), 7.26-7.21 (m, 3H), 5.19 (s, 2H), 3.70 (s, 6H), 3.59 (d, *J* = 4.8 Hz, 2H), 3.43-3.39 (m, 2H), 3.11-3.07 (m, 2H); ^{19}F NMR (376 MHz, $CDCl_3$): δ = -98.29 (s); ^{13}C NMR (100 MHz, $CDCl_3$): δ = 172.9, 171.0, 160.1 (d, *J* = 259.6 Hz), 143.2 (d, *J* = 4.8 Hz), 138.2, 135.6, 128.7 (d, *J* = 3.5 Hz), 128.4, 128.2, 128.1, 127.9, 126.9, 126.7, 126.1, 113.0 (d, *J* = 18.1 Hz), 67.1, 62.7, 52.6, 48.5, 44.9 (d, *J* = 27.7 Hz), 41.4, 36.6 (d, *J* = 4.8 Hz) ppm; HRMS (ESI) calcd for $C_{32}H_{30}FO_6$ $[M+H]^+$ 529.2021, found 529.2014.

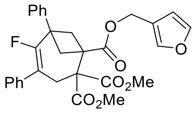

1-(Furan-3-ylmethyl) 2,2-dimethyl 5-fluoro-4,6-diphenylbicyclo[4.1.1]oct-4-ene-1,2,2-tricarboxylate (**3pa**): Colorless oil (65%, 69.5 mg); R_f = 0.3 (petroleum ether/EtOAc = 3/1); ^{1}H NMR (400 MHz, $CDCl_3$): δ = 7.49-7.46 (m, 2H), 7.41-7.28 (m, 6H), 7.24-7.17 (m, 3H), 6.40-6.34 (m, 2H), 5.10 (s, 2H), 3.72 (s, 6H), 3.55 (d, *J* = 4.8 Hz, 2H), 3.36-3.31 (m, 2H), 3.06-3.02 (m, 2H); ^{19}F NMR (376 MHz, $CDCl_3$): δ = -97.55 (s); ^{13}C NMR (100 MHz, $CDCl_3$): δ = 172.8, 171.0, 160.1 (d, *J* = 258.8 Hz), 149.0, 143.2 (d, *J* = 4.8 Hz), 143.1, 138.2, 128.7 (d, *J* = 3.4 Hz), 128.1, 128.0, 126.9, 126.7, 126.1, 112.9 (d, *J* = 18.0 Hz), 110.8, 110.5, 62.8, 59.0, 52.7, 48.4, 44.9 (d, *J* = 27.7 Hz), 41.3, 36.6 (d, *J* = 4.6 Hz) ppm; HRMS (ESI) calcd for $C_{30}H_{27}KFO_7$ $[M+K]^+$ 557.1372, found 557.1375.

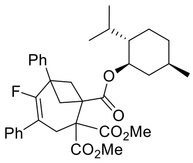

1-(1R,2S,5R)-2-isopropyl-5-methylcyclohexyl) 2,2-dimethyl 5-fluoro-4,6-diphenylbicyclo[4.1.1]oct-4-ene-1,2,2-tricarboxylate (**3qa**): Colorless oil (92%, 105.6 mg); R_f = 0.3 (petroleum ether/EtOAc = 5/1); ^{1}H NMR (400 MHz, $CDCl_3$): δ = 7.50-7.47 (m, 2H), 7.39-7.27 (m, 5H), 7.23-7.18 (m, 3H), 4.71 (td, *J* = 10.8, 4.0 Hz, 1H), 3.80 (s, 3H), 3.77 (s, 3H), 3.55 (d, *J* = 4.8 Hz, 2H), 3.36-3.33 (m, 1H), 3.28-3.24 (m, 1H), 3.05-3.97 (m, 2H), 1.98-1.93 (m, 1H), 1.84-1.77 (m, 1H), 1.69-1.64 (m, 2H), 1.50-1.43 (m, 1H), 1.40-1.33 (m, 1H), 1.06-0.98 (m, 1H), 0.92-0.83 (m, 8H), 0.74 (d, *J* = 7.2 Hz, 3H); ^{19}F NMR (376 MHz, $CDCl_3$): δ = -97.66 (s); ^{13}C NMR (100 MHz, $CDCl_3$): δ = 172.4, 171.24, 171.19, 160.2 (d, *J* = 258.9 Hz), 143.3 (d, *J* = 5.1 Hz), 138.3, 128.7 (d, *J* = 3.6 Hz), 128.2, 128.0, 126.9, 126.6, 126.1, 113.0 (d, *J* = 17.9 Hz), 75.6, 62.8, 52.7, 52.6, 48.6, 46.8, 44.9 (d, *J* = 27.6 Hz), 41.6, 41.1, 40.3, 36.8 (d, *J* = 4.1 Hz), 34.1, 31.3, 26.0, 23.2, 21.9, 20.7, 16.1 ppm; HRMS (ESI) calcd for $C_{35}H_{42}FO_6$ $[M+H]^+$ 577.2960, found 577.2961.

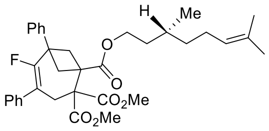

(*R*)-1-(3,7-dimethyloct-6-en-1-yl) 2,2-dimethyl 5-fluoro-4,6-diphenylbicyclo[4.1.1]oct-4-ene-1,2,2-tricarboxylate (**3ra**): Colorless oil (88%, 101.8 mg); R_f = 0.3 (petroleum ether/EtOAc = 5/1); ^{1}H NMR (400 MHz, $CDCl_3$): δ = 7.48-7.45 (m, 2H), 7.39-7.27 (m, 5H), 7.24-7.18 (m, 3H), 5.08-5.04 (m, 1H), 4.16-4.13 (m, 2H), 3.80 (s, 6H), 3.56 (d, *J* = 4.8 Hz, 2H), 3.34-3.30 (m, 2H), 3.05-3.01 (m, 2H), 1.97-1.84 (m, 2H), 1.69-1.59 (m, 7H), 1.50-1.39 (m, 2H), 1.32-1.27 (m, 1H), 1.19-1.10 (m, 1H), 0.88-0.86 (m, 3H); ^{19}F NMR (376 MHz, $CDCl_3$): δ = -97.61 (s); ^{13}C NMR (100 MHz, $CDCl_3$): δ = 173.0, 171.2, 160.2 (d, *J* = 259.5 Hz), 143.3 (d, *J* = 5.4 Hz), 138.3, 131.3, 128.7 (d, *J* = 2.8 Hz), 128.2, 128.0, 127.0, 126.7, 126.1, 124.4, 113.0 (d, *J* = 18.0 Hz), 64.3, 62.7, 52.8, 48.6, 45.0 (d, *J* = 27.5 Hz), 41.5, 36.9, 36.7 (d, *J* = 4.8 Hz), 35.2, 29.5, 25.7, 25.4, 19.3, 17.6 ppm; HRMS (ESI) calcd for $C_{35}H_{42}FO_6$ $[M+H]^+$ 577.2960, found 577.2961.

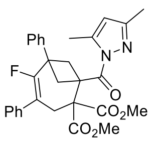

Dimethyl 1-(3,5-dimethyl-1H-pyrazole-1-carbonyl)-5-fluoro-4,6-diphenylbicyclo[4.1.1]oc-

t-4-ene-2,2-dicarboxylate (**3sa**): Colorless oil (58%, 59.9 mg); R_f = 0.2 (petroleum ether/EtOAc = 3/1); ^{1}H NMR (400 MHz, $CDCl_3$): δ = 7.45-7.42 (m, 2H), 7.34-7.30 (m, 4H), 7.24-7.20 (m, 4H), 5.85 (s, 1H), 3.69 (d, *J* = 4.8 Hz, 2H), 3.60 (s, 6H), 3.50-3.46 (m, 2H), 3.39-3.34 (m, 2H), 2.52 (s, 3H), 2.04 (s, 3H); ^{19}F NMR (376 MHz, $CDCl_3$): δ = -98.10 (s); ^{13}C NMR (100 MHz, $CDCl_3$): δ = 173.4, 169.9, 160.4 (d, *J* = 259.9 Hz), 150.9, 144.6, 143.5 (d, *J* = 4.7 Hz), 138.9, 128.7 (d, *J* = 2.6 Hz), 128.01, 127.96, 126.8, 126.5, 126.4, 112.6 (d, *J* = 17.2 Hz), 110.1, 64.1, 52.8, 51.2, 44.8 (d, *J* = 27.5 Hz), 42.7, 35.7 (d, *J* = 4.3 Hz), 14.4, 13.5 ppm; HRMS (ESI) calcd for $C_{30}H_{29}LiN_2FO_5$ $[M+Li]^+$ 523.2215, found 523.2224.

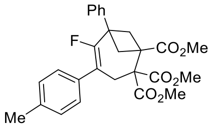

Trimethyl 5-fluoro-6-phenyl-4-(*p*-tolyl)bicyclo[4.1.1]oct-4-ene-1,2,2-tricarboxylate (**3ab**): White solid (92%, 86.0 mg); m.p.: 140-141 °C; R_f = 0.3 (petroleum ether/EtOAc = 3/1); ^{1}H NMR (400 MHz, $CDCl_3$): δ = 7.40-7.35 (m, 4H), 7.31-7.27 (m, 1H), 7.22-7.20 (m, 2H), 7.15-7.12 (m, 2H), 3.81 (s, 6H), 3.71 (s, 3H), 3.56 (d, *J* = 4.8 Hz, 2H), 3.37-3.33 (m, 2H), 3.08-3.03 (m, 2H), 2.33 (s, 3H); ^{19}F NMR (376 MHz, $CDCl_3$): δ = -97.97 (s); ^{13}C NMR (100 MHz, $CDCl_3$): δ = 173.4, 171.2, 159.9 (d, *J* = 258.1 Hz), 143.4 (d, *J* = 4.9 Hz), 136.6, 135.3, 128.7, 128.5 (d, *J* = 3.5 Hz), 128.2, 126.7, 126.2, 112.7 (d, *J* = 17.9 Hz), 62.7, 52.8, 52.7, 48.6, 45.0 (d, *J* = 27.5 Hz), 41.4, 36.6 (d, *J* = 4.7 Hz), 21.1 ppm; HRMS (ESI) calcd for $C_{27}H_{28}FO_6$ $[M+H]^+$ 467.1864, found 467.1867.

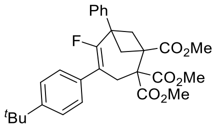

Trimethyl 4-(4-(*tert*-butyl)phenyl)-5-fluoro-6-phenylbicyclo[4.1.1]oct-4-ene-1,2,2-tricarboxylate (**3ac**): Colorless oil (92%, 93.6 mg); R_f = 0.3 (petroleum ether/EtOAc = 3/1); ^{1}H NMR (400 MHz, $CDCl_3$): δ = 7.45-7.35 (m, 6H), 7.31-7.28 (m, 1H), 7.24-7.21 (m, 2H), 3.82 (s, 6H), 3.72 (s, 3H), 3.59 (d, *J* = 4.8 Hz, 2H), 3.38-3.34 (m, 2H), 3.09-3.05 (m, 2H), 1.32 (s, 9H); ^{19}F NMR (376 MHz, $CDCl_3$): δ = -97.88 (s); ^{13}C NMR (100 MHz, $CDCl_3$): δ = 173.4, 171.2, 159.9 (d, *J* = 258.8 Hz), 149.8, 143.4 (d, *J* = 5.0 Hz), 135.1, 128.2 (d, *J* = 3.5 Hz), 128.1, 126.6, 126.1, 124.8, 112.6 (d, *J* = 17.3 Hz), 62.7, 52.8, 52.6, 48.5, 45.0 (d, *J* = 27.8 Hz), 41.4, 36.4 (d, *J* = 4.7 Hz), 34.4, 31.2 ppm; HRMS (ESI) calcd for $C_{30}H_{34}FO_6$ $[M+H]^+$ 509.2334, found 509.2347.

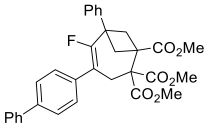

Trimethyl 4-([1,1'-biphenyl]-4-yl)-5-fluoro-6-phenylbicyclo[4.1.1]oct-4-ene-1,2,2-tricarboxylate (**3ad**): Colorless oil (89%, 94.1 mg); R_f = 0.3 (petroleum ether/EtOAc = 3/1); ^{1}H NMR (400 MHz, $CDCl_3$): δ = 7.64-7.59 (m, 6H), 7.48-7.27 (m, 7H), 7.24 (s, 1H), 3.85 (s, 6H), 3.74 (s, 3H), 3.64 (d, *J* = 4.8 Hz, 2H), 3.42-3.38 (m, 2H), 3.13-3.08 (m, 2H); ^{19}F NMR (376 MHz, $CDCl_3$): δ = -97.05 (s); ^{13}C NMR (100 MHz, $CDCl_3$): δ = 173.4, 171.2, 160.3 (d, *J* = 259.3 Hz), 143.3 (d, *J* = 4.7 Hz), 140.7, 139.7, 137.2, 129.1 (d, *J* = 3.6 Hz), 128.6, 128.2, 127.1, 126.9, 126.7, 126.6, 126.1, 112.5 (d, *J* = 17.3 Hz), 62.7, 52.8, 52.7, 48.5, 45.1 (d, *J* = 27.6 Hz), 41.4, 36.4 (d, *J* = 4.8 Hz) ppm; HRMS (ESI) calcd for $C_{32}H_{30}FO_6$ $[M+H]^+$ 529.2021, found 529.2025.

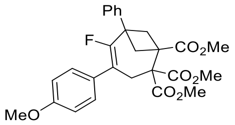

Trimethyl 5-fluoro-4-(4-methoxyphenyl)-6-phenylbicyclo[4.1.1]oct-4-ene-1,2,2-tricarboxylate (**3ae**): Colorless oil (56%, 54.0 mg); R_f = 0.3 (petroleum ether/EtOAc = 3/1); ^{1}H NMR (400 MHz, $CDCl_3$): δ = 7.42-7.36 (m, 4H), 7.30-7.27 (m, 1H), 7.21-7.19 (m, 2H), 6.87-6.83 (m, 2H), 3.80 (s, 6H), 3.79 (s, 3H), 3.70 (s, 3H), 3.54 (d, *J* = 4.8 Hz, 2H), 3.35-3.31 (m, 2H), 3.06-3.01 (m, 2H); ^{19}F NMR (376 MHz, $CDCl_3$): δ = -99.29 (s); ^{13}C NMR (100 MHz, $CDCl_3$): δ = 173.5, 171.3, 159.7 (d, *J* = 257.1 Hz), 158.4, 143.5 (d, *J* = 4.8Hz), 130.5, 129.9 (d, *J* = 3.4 Hz), 128.2, 126.7, 126.2, 113.4, 112.4 (d, *J* = 18.0 Hz), 62.8, 55.2, 52.8, 52.7, 48.6, 45.0 (d, *J* = 27.7 Hz), 41.4, 36.7 (d, *J* = 4.6 Hz) ppm; HRMS (ESI) calcd for $C_{27}H_{27}KFO_7$ $[M+K]^+$ 521.1372, found 521.1361.

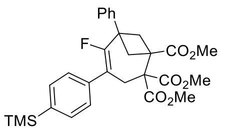

Trimethyl 5-fluoro-6-phenyl-4-(4-(trimethylsilyl)phenyl)bicyclo[4.1.1]oct-4-ene-1,2,2-tri-carboxylate (**3af**): Colorless oil (81%, 85.0 mg); R_f = 0.3 (petroleum ether/EtOAc = 3/1); ^{1}H NMR (400 MHz, $CDCl_3$): δ = 7.49-7.43 (m, 4H), 7.39-7.36 (m, 2H), 7.30-7.26 (m, 1H), 7.21-7.19 (m, 2H), 3.81 (s, 6H), 3.71 (s, 3H), 3.57 (d, *J* = 4.8 Hz, 2H), 3.36-3.32 (m, 2H), 3.07-3.03 (m, 2H), 0.25 (s, 9H); ^{19}F NMR (376 MHz, $CDCl_3$): δ = -97.29 (s); ^{13}C NMR (100 MHz, $CDCl_3$): δ = 173.5, 171.2, 160.3 (d, *J* = 259.3 Hz), 143.3 (d, *J* = 4.9 Hz), 139.1, 138.7, 133.0, 128.2, 127.9 (d, *J* = 3.1 Hz), 126.7, 126.2,

112.8 (d, J = 18.0 Hz), 62.7, 52.8, 52.7, 48.6, 45.0 (d, J = 27.7 Hz), 41.4, 36.4 (d, J = 4.7 Hz), -1.20 ppm; HRMS (ESI) calcd for $C_{29}H_{33}SiKFO_6$ $[M+K]^+$ 563.1662, found 563.1666.

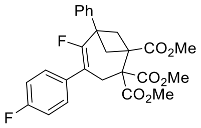

Trimethyl 5-fluoro-4-(4-fluorophenyl)-6-phenylbicyclo[4.1.1]oct-4-ene-1,2,2-tricarboxylate (**3ag**): White solid (94%, 88.3 mg); m.p.: 125-126 °C; R_f = 0.3 (petroleum ether/EtOAc = 3/1); ^{1}H NMR (400 MHz, $CDCl_3$): δ = 7.47-7.43 (m, 2H), 7.40-7.36 (m, 2H), 7.31-7.27 (m, 1H), 7.20-7.18 (m, 2H), 7.02-6.97 (m, 2H), 3.80 (s, 6H), 3.71 (s, 3H), 3.52 (d, J = 4.8 Hz, 2H), 3.36-3.32 (m, 2H), 3.05-3.01 (m, 2H); ^{19}F NMR (376 MHz, $CDCl_3$): δ = -97.47 (s), -115.11 (s); ^{13}C NMR (100 MHz, $CDCl_3$): δ = 173.3, 171.2, 161.6 (d, J = 244.5 Hz), 160.2 (d, J = 259.0 Hz), 143.2 (d, J = 5.0 Hz), 134.1 (d, J = 3.7 Hz), 130.5 (dd, J = 7.8, 3.7 Hz), 128.2, 126.8, 126.1, 114.8 (d, J = 21.2 Hz), 112.1 (d, J = 17.8 Hz), 62.8, 52.9, 52.7, 48.5, 45.0 (d, J = 27.6 Hz), 41.4, 36.6 (d, J = 4.0 Hz) ppm; HRMS (ESI) calcd for $C_{26}H_{24}KF_2O_6$ $[M+K]^+$ 509.1173, found 509.1199.

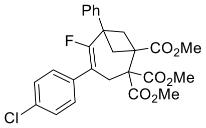

Trimethyl 4-(4-chlorophenyl)-5-fluoro-6-phenylbicyclo[4.1.1]oct-4-ene-1,2,2-tricarboxylate (**3ah**): White solid (90%, 87.8 mg); m.p.: 137-138 °C; R_f = 0.3 (petroleum ether/EtOAc = 3/1); ^{1}H NMR (400 MHz, $CDCl_3$): δ = 7.39-7.32 (m, 4H), 7.24-7.21 (m, 3H), 7.16-7.13 (m, 2H), 3.76 (s, 6H), 3.66 (s, 3H), 3.47 (d, J = 4.8 Hz, 2H), 3.32-3.28 (m, 2H), 3.01-2.96 (m, 2H); ^{19}F NMR (376 MHz, $CDCl_3$): δ = -97.41 (s); ^{13}C NMR (100 MHz, $CDCl_3$): δ = 173.3, 171.2, 160.5 (d, J = 260.4 Hz), 143.1 (d, J = 4.8 Hz), 136.7, 132.6, 130.1 (d, J = 3.4 Hz), 128.2, 128.1, 126.8, 126.1, 112.0 (d, J = 17.9 Hz), 62.7, 52.9, 52.7, 49.5, 45.0 (d, J = 27.1 Hz), 41.3, 36.4 (d, J = 4.3 Hz) ppm; HRMS (ESI) calcd for $C_{26}H_{25}ClFO_6$ $[M+H]^+$ 487.1318, found 487.1322.

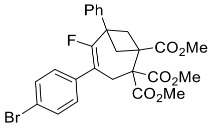

Trimethyl 4-(4-bromophenyl)-5-fluoro-6-phenylbicyclo[4.1.1]oct-4-ene-1,2,2-tricarboxylate (**3ai**): Colorless oil (97%, 94.0 mg); R_f = 0.3 (petroleum ether/EtOAc = 3/1); ^{1}H NMR (400 MHz, $CDCl_3$): δ = 7.45-7.42 (m, 3H), 7.38-7.36 (m, 3H), 7.30-7.28 (m, 1H), 7.20-7.18 (m, 2H), 3.80 (s, 6H), 3.71 (s,

3H), 3.52 (d, *J* = 4.8 Hz, 2H), 3.36-3.32 (m, 2H), 3.05-3.01 (m, 2H); ^{19}F NMR (376 MHz, $CDCl_3$): δ = -96.55 (s); ^{13}C NMR (100 MHz, $CDCl_3$): δ = 173.2, 171.1, 160.5 (d, *J* = 260.3 Hz), 143.0 (d, *J* = 4.8 Hz), 137.2, 131.1, 130.5 (d, *J* = 3.6 Hz), 128.2, 126.8, 126.1, 120.9, 112.0 (d, *J* = 17.3 Hz), 62.7, 52.9, 52.7, 48.5, 45.0 (d, *J* = 27.6 Hz), 41.3, 36.3 (d, *J* = 4.5 Hz) ppm; HRMS (ESI) calcd for $C_{26}H_{25}BrFO_6$ $[M+H]^+$ 531.0813, found 531.0824.

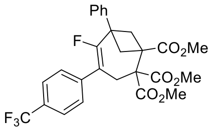

Trimethyl 5-fluoro-6-phenyl-4-(4-(trifluoromethyl)phenyl)bicyclo[4.1.1]oct-4-ene-1,2,2-tri-carboxylate (**3aj**): Colorless oil (69%, 71.8 mg); R_f = 0.3 (petroleum ether/EtOAc = 3/1); ^{1}H NMR (400 MHz, $CDCl_3$): δ = 7.61-7.55 (m, 4H), 7.40-7.37 (m, 2H), 7.31-7.29 (m, 1H), 7.21-7.19 (m, 2H), 3.81 (s, 6H), 3.71 (s, 3H), 3.55 (d, *J* = 4.8 Hz, 2H), 3.39-3.35 (m, 2H), 3.07-3.03 (m, 2H); ^{19}F NMR (376 MHz, $CDCl_3$): δ = -62.41 (s, 3F), -95.78 (s, 1F); ^{13}C NMR (100 MHz, $CDCl_3$): δ = 173.2, 171.1, 161.2 (d, *J* = 261.6 Hz), 142.9 (d, *J* = 4.9 Hz), 142.0, 129.1 (d, *J* = 3.7 Hz), 128.9 (q, *J* = 33.2 Hz), 128.3, 126.9, 126.2, 124.9 (q, *J* = 3.0 Hz), 124.1 (q, *J* = 270.5 Hz), 112.0 (d, *J* = 17.3 Hz), 62.8, 52.9, 52.8, 48.5, 45.1 (d, *J* = 27.0 Hz), 41.4, 36.3 (d, *J* = 4.5 Hz) ppm; HRMS (ESI) calcd for $C_{27}H_{24}LiF_4O_6$ $[M+Li]^+$ 527.1664, found 527.1640.

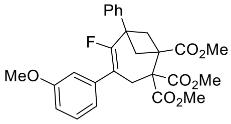

Trimethyl 5-fluoro-4-(3-methoxyphenyl)-6-phenylbicyclo[4.1.1]oct-4-ene-1,2,2-tricarboxylate (**3ak**): White solid (86%, 83.2 mg); m.p.: 139-140 ^{o}C; R_f = 0.3 (petroleum ether/EtOAc = 3/1); ^{1}H NMR (400 MHz, $CDCl_3$): δ = 7.40-7.36 (m, 2H), 7.30-7.28 (m, 1H), 7.23-7.19 (m, 3H), 7.05-7.03 (m, 2H), 6.80-6.77 (m, 1H), 3.81 (s, 6H), 3.80 (s, 3H), 3.71 (s, 3H), 3.56 (d, *J* = 4.8 Hz, 2H), 3.36-3.32 (m, 2H), 3.08-3.03 (m, 2H); ^{19}F NMR (376 MHz, $CDCl_3$): δ = -96.77 (s); ^{13}C NMR (100 MHz, $CDCl_3$): δ = 173.4, 171.2, 160.2 (d, *J* = 259.6 Hz), 159.2, 143.3 (d, *J* = 4.8 Hz), 139.6, 128.9, 128.2, 126.7, 126.2, 121.2 (d, *J* = 3.1 Hz), 114.2 (d, *J* = 3.2 Hz), 112.9, 112.7, 62.7, 55.1, 52.8, 52.7, 48.6, 45.0 (d, *J* = 27.1 Hz), 41.4, 36.5 (d, *J* = 4.7 Hz) ppm; HRMS (ESI) calcd for $C_{27}H_{27}KFO_7$ $[M+K]^+$ 521.1372, found 521.1382.

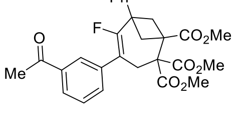

Trimethyl 4-(3-acetylphenyl)-5-fluoro-6-phenylbicyclo[4.1.1]oct-4-ene-1,2,2-tricarboxylate (3al): White solid (89%, 88.5 mg); m.p.: 117-118 °C; R_f = 0.2 (petroleum ether/EtOAc = 3/1); ^{1}H NMR (400 MHz, $CDCl_3$): δ = 8.08 (s, 1H), 7.82-7.80 (m, 1H), 7.67-7.64 (m, 1H), 7.42-7.35 (m, 3H), 7.30-7.27 (m, 1H), 7.20-7.18 (m, 2H), 3.82 (s, 6H), 3.70 (s, 3H), 3.56 (d, *J* = 4.8 Hz, 2H), 3.37-3.33 (m, 2H), 3.08-3.03 (m, 2H), 2.60 (s, 3H); ^{19}F NMR (376 MHz, $CDCl_3$): δ = -97.21 (s); ^{13}C NMR (100 MHz, $CDCl_3$): δ = 198.1, 173.3, 171.1, 160.9 (d, *J* = 260.3 Hz), 143.0 (d, *J* = 4.9 Hz), 138.8, 137.0, 133.6, 133.56, 128.6 (d, *J* = 2.9 Hz), 128.2, 126.9, 126.8, 126.2, 112.2 (d, *J* = 17.4 Hz), 62.8, 52.9, 52.7, 48.6, 45.0 (d, *J* = 27.2 Hz), 41.4, 36.4 (d, *J* = 4.4 Hz), 26.6 ppm; HRMS (ESI) calcd for $C_{28}H_{27}NaFO_7$ $[M+Na]^+$ 517.1633, found 517.1639

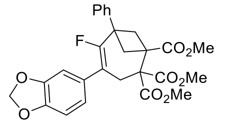

Trimethyl 4-(benzo[*d*][1,3]dioxol-5-yl)-5-fluoro-6-phenylbicyclo[4.1.1]oct-4-ene-1,2,2-tri-carboxylate (3am): White solid (59%, 58.6 mg); m.p.: 142-143 °C; R_f = 0.3 (petroleum ether/EtOAc = 3/1); ^{1}H NMR (400 MHz, $CDCl_3$): δ = 7.39-7.35 (m, 2H), 7.30-7.27 (m, 1H), 7.19-7.17 (m, 2H), 6.98-6.92 (m, 2H), 6.76 (d, *J* = 8.0 Hz, 1H), 5.91 (s, 2H), 3.80 (s, 6H), 3.70 (s, 3H), 3.51 (d, *J* = 4.8 Hz, 2H), 3.34-3.30 (m, 2H), 3.04-2.99 (m, 2H); ^{19}F NMR (376 MHz, $CDCl_3$): δ = -97.55 (s); ^{13}C NMR (100 MHz, $CDCl_3$): δ = 173.4, 171.2, 159.9 (d, *J* = 258.3 Hz), 147.2, 146.3, 143.3 (d, *J* = 5.2 Hz), 132.0, 128.2, 126.7, 126.2, 122.2 (d, *J* = 3.4 Hz), 112.6 (d, *J* = 17.8 Hz), 109.4 (d, *J* = 3.8 Hz), 107.8, 100.86, 62.7, 52.8, 52.7, 48.5, 45.0 (d, *J* = 27.8 Hz), 41.4, 36.8 (d, *J* = 4.3 Hz) ppm; HRMS (ESI) calcd for $C_{27}H_{25}KFO_8$ $[M+K]^+$ 535.1165, found 535.1171.

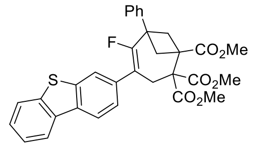

Trimethyl 4-(dibenzo[*b*,*d*]thiophen-3-yl)-5-fluoro-6-phenylbicyclo[4.1.1]oct-4-ene-1,2,2-tri-carboxylate (3an): Colorless oil (63%, 74.2 mg); R_f = 0.3 (petroleum ether/EtOAc = 3/1); ^{1}H NMR (400 MHz, $CDCl_3$): δ = 8.31 (s, 1H), 8.19-8.17 (m, 1H), 7.85-7.79 (m, 2H), 7.62-7.59 (m, 1H), 7.46-7.40 (m, 4H), 7.34-7.27 (m, 2H), 7.25-7.24 (m, 1H), 3.86 (s, 6H), 3.74 (s, 3H), 3.70 (d, *J* = 4.8 Hz, 2H), 3.43-3.39 (m, 2H), 3.15-3.11 (m, 2H); ^{19}F NMR (376 MHz, $CDCl_3$): δ = -97.30 (s); ^{13}C NMR (100 MHz, $CDCl_3$): δ = 173.4, 171.3, 160.4 (d, *J* = 259.6 Hz), 143.3 (d, *J* = 4.8 Hz), 139.6, 138.0, 135.4 (d, *J* = 4.5 Hz), 134.7, 128.2, 127.6, 126.8, 126.6, 126.2, 124.2, 122.7, 122.2, 121.8, 121.6, 112.8

(d, J = 17.4 Hz), 62.9, 52.9, 52.7, 48.6, 45.1 (d, J = 27.5 Hz), 41.5, 36.9 (d, J = 4.5 Hz) ppm; HRMS (ESI) calcd for $C_{32}H_{28}FO_6S$ $[M+H]^+$ 559.1585, found 559.1588.

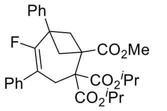

2,2-Diisopropyl 1-methyl 5-fluoro-4,6-diphenylbicyclo[4.1.1]oct-4-ene-1,2,2-tricarboxylate (**3ao**): White solid (38%, 38.7 mg); m.p.: 106-107 ^{o}C; R_f = 0.4 (petroleum ether/EtOAc = 3/1); ^{1}H NMR (400 MHz, $CDCl_3$): δ = 7.52-7.49 (m, 2H), 7.39-7.27 (m, 5H), 7.24-7.20 (m, 3H), 5.16-5.06 (m, 2H), 3.68 (s, 3H), 3.54 (d, J = 4.8 Hz, 2H), 3.35-3.31 (m, 2H), 3.09-3.04 (m, 2H), 1.28 (d, J = 6.0 Hz, 6H), 1.25 (d, J = 6.0 Hz, 6H); ^{19}F NMR (376 MHz, $CDCl_3$): δ = -97.81 (s); ^{13}C NMR (100 MHz, $CDCl_3$): δ = 173.5, 170.1, 160.3 (d, J = 258.9 Hz), 143.5 (d, J = 4.9 Hz), 138.5, 128.8 (d, J = 3.5 Hz), 128.2, 127.9, 126.9, 126.6, 126.2, 112.9 (d, J = 17.7 Hz), 69.4, 62.7, 52.4, 48.3, 44.9 (d, J = 27.7 Hz), 41.5, 36.5 (d, J = 4.4 Hz), 21.6, 21.5 ppm; HRMS (ESI) calcd for $C_{30}H_{34}FO_6$ $[M+H]^+$ 509.2334, found 509.2343.

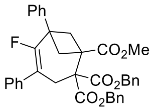

2,2-Dibenzyl 1-methyl 5-fluoro-4,6-diphenylbicyclo[4.1.1]oct-4-ene-1,2,2-tricarboxylate (**3ap**): Colorless oil (85%, 102.7 mg); R_f = 0.3 (petroleum ether/EtOAc = 3/1); ^{1}H NMR (400 MHz, $CDCl_3$): δ = 7.45-7.43 (m, 2H), 7.41-7.38 (m, 2H), 7.37-7.35 (m, 6H), 7.33-7.28 (m, 6H), 7.25-7.20 (m, 4H), 5.24 (s, 4H), 3.65 (d, J = 4.8 Hz, 2H), 3.54 (s, 3H), 3.37-3.34 (m, 2H), 3.12-3.07 (m, 2H); ^{19}F NMR (376 MHz, $CDCl_3$): δ = -97.25 (s); ^{13}C NMR (100 MHz, $CDCl_3$): δ = 173.4, 170.4, 160.3 (d, J = 259.3 Hz), 143.2 (d, J = 4.9 Hz), 138.1, 135.1, 128.7 (d, J = 3.2 Hz), 128.4, 128.2, 128.22, 128.1, 127.9, 126.9, 126.7, 126.2, 112.7 (d, J = 17.3 Hz), 67.7, 62.9, 52.4, 48.6, 44.9 (d, J = 27.7 Hz), 41.4, 36.4 (d, J = 4.3 Hz) ppm; HRMS (ESI) calcd for $C_{38}H_{34}FO_6$ $[M+H]^+$ 605.2334, found 605.2344.

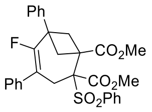

Dimethyl 5-fluoro-4,6-diphenyl-2-(phenylsulfonyl)bicyclo[4.1.1]oct-4-ene-1,2-dicarboxylate (**3aq**): White solid (79%, 84.5 mg); m.p.: 92-93 ^{o}C; R_f = 0.3 (petroleum ether/EtOAc = 3/1); ^{1}H NMR (400 MHz, $CDCl_3$): δ = 7.92 (d, J = 7.6 Hz, 2H), 7.68-7.64 (m, 1H), 7.55-7.51 (m, 2H), 7.37-7.33 (m, 4H), 7.30-7.27 (m, 2H), 7.26-7.19 (m, 2H), 7.16-7.13 (m, 2H), 3.83 (s, 3H), 3.77 (dd, J = 16.8, 4.8 Hz, 1H), 3.71-3.67 (m, 4H), 3.39 (dd, J = 17.2, 1.0 Hz, 1H), 3.26 (dd, J = 12.8, 0.9 Hz, 1H), 3.17 (d, J = 12.0 Hz, 1H), 3.09 (dd, J = 12.0, 5.6 Hz, 1H); ^{19}F NMR (376 MHz, $CDCl_3$): δ = -97.70 (s); ^{13}C NMR (100 MHz,

$CDCl_3$): δ = 173.5, 167.4, 159.0 (d, *J* = 258.9 Hz), 142.8 (d, *J* = 5.0 Hz), 138.1, 137.4, 134.4, 130.6, 128.8, 128.5 (d, *J* = 3.1 Hz), 128.2, 128.0, 127.1, 126.8, 126.1, 111.9 (d, *J* = 19.1 Hz), 80.2, 53.2, 52.7, 47.4, 45.0, 44.2 (d, *J* = 27.7 Hz), 39.0, 35.2 (d, *J* = 4.7 Hz) ppm; HRMS (ESI) calcd for $C_{30}H_{28}FO_6S$ $[M+H]^+$ 535.1585, found 535.1580.

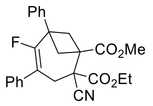

2-Ethyl 1-methyl 2-cyano-5-fluoro-4,6-diphenylbicyclo[4.1.1]oct-4-ene-1,2-dicarboxylate (**3ar**): Colorless oil (72%, 62.4 mg); R_f = 0.2 (petroleum ether/EtOAc = 1/1); ^1H NMR (400 MHz, $CDCl_3$): δ = 7.40-7.34 (m, 6H), 7.32-7.26 (m, 2H), 7.19 (d, *J* = 7.2 Hz, 2H), 4.29 (q, *J* = 7.2 Hz, 2H), 3.74-3.69 (m, 4H), 3.41 (d, *J* = 3.6 Hz, 2H), 3.16-3.07 (m, 3H), 1.33 (t, *J* = 7.2 Hz, 3H); ^{19}F NMR (376 MHz, $CDCl_3$): δ = -94.53 (s); ^{13}C NMR (100 MHz, $CDCl_3$): δ = 172.6, 168.9, 160.7 (d, *J* = 261.6 Hz), 142.3 (d, *J* = 4.8 Hz), 137.3, 128.4 (d, *J* = 2.9 Hz), 128.3, 127.4, 127.1, 126.1, 118.2, 111.4 (d, *J* = 20.4 Hz), 63.4, 52.8, 52.5, 48.1, 45.7 (d, *J* = 27.7 Hz), 44.9 (d, *J* = 4.0 Hz), 39.0 (d, *J* = 4.7 Hz), 34.7 (d, *J* = 3.0 Hz), 13.9 ppm; HRMS (ESI) calcd for $C_{26}H_{25}FNO_4$ $[M+H]^+$ 434.1762, found 434.1765.

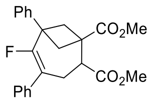

Dimethyl 5-fluoro-4,6-diphenylbicyclo[4.1.1]oct-4-ene-1,2-dicarboxylate (**4aa**): Colorless oil (76%, 60.0 mg); R_f = 0.4 (petroleum ether/EtOAc = 5/1); ^1H NMR (400 MHz, $CDCl_3$): δ = 7.37-7.35 (m, 2H), 7.27-7.21 (m, 4H), 7.17-7.09 (m, 4H), 3.81 (t, *J* = 5.2 Hz, 1H), 3.64 (d, *J* = 3.2 Hz, 6H), 3.34 (d, *J* = 11.6 Hz, 1H), 3.18-3.07 (m, 3H), 2.65 (dd, *J* = 11.6, 4.2 Hz, 1H), 2.53 (dd, *J* = 11.6, 6.0 Hz, 1H); ^{19}F NMR (376 MHz, $CDCl_3$): δ = -97.74 (s); ^{13}C NMR (100 MHz, $CDCl_3$): δ = 175.1, 173.5, 159.5 (d, *J* = 257.3 Hz), 143.6 (d, *J* = 5.2 Hz), 138.9, 128.6 (d, *J* = 3.3 Hz), 128.1, 128.0, 126.8, 126.6, 126.2, 114.1 (d, *J* = 17.1 Hz), 52.4, 52.0, 47.7, 45.2 (d, *J* = 28.1 Hz), 45.0, 41.4, 39.2 (d, *J* = 2.3 Hz), 30.2 (d, *J* = 4.5 Hz) ppm; HRMS (ESI) calcd for $C_{24}H_{24}FO_4$ $[M+H]^+$ 395.1653, found 395.1657.

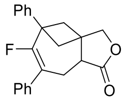

6-Fluoro-5,7-diphenyl-4,5,8,8a-tetrahydro-1*H*,3*H*-3a,5-methanocyclohepta[*c*]furan-1-one (**5aa**): White solid (45%, 45.1 mg); m.p.: 180-181 °C; R_f = 0.4 (petroleum ether/EtOAc = 10/1); ^1H NMR (400 MHz, $CDCl_3$): δ = 7.41-7.28 (m, 8H), 7.25-7.21 (m, 2H), 4.25 (d, *J* = 9.6 Hz, 1H), 4.18 (d, *J* = 10.0 Hz, 1H), 3.44 (dd, *J* = 11.2, 6.8 Hz, 1H), 3.31-3.22 (m, 1H), 3.13-3.04 (m, 2H), 2.85 (d, *J* = 10.0

Hz, 1H), 2.68-2.62 (m, 1H), 2.37 (dd, J = 11.2, 4.8 Hz, 1H); ^{19}F NMR (376 MHz, $CDCl_3$): δ = -95.94 (s); ^{13}C NMR (100 MHz, $CDCl_3$): δ = 177.2, 159.9 (d, J = 257.3 Hz), 143.1 (d, J = 5.7 Hz), 138.5, 128.3, 128.2, 128.1, 127.0, 126.8, 126.2, 113.6 (d, J = 17.7 Hz), 74.9, 46.64, 46.6 (d, J =28.4 Hz), 43.9 (d, J = 3.6 Hz), 42.6, 39.1 (d, J = 2.6 Hz), 28.9 (d, J = 4.8 Hz) ppm; HRMS (ESI) calcd for $C_{22}H_{20}FO_2$ $[M+H]^+$ 335.1442, found 335.1445.

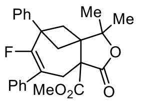

Methyl 6-fluoro-3,3-dimethyl-1-oxo-5,7-diphenyl-5,8-dihydro-1*H*,3*H*-3a,5-methanocyclohepta[c]furan-8a(4*H*)-carboxylatee (**6ka**): Colorless oil (55%, 46.2 mg); R_f = 0.3 (petroleum ether/EtOAc = 5/1); ^{1}H NMR (400 MHz, $CDCl_3$): δ = 7.51 (d, J = 8.0 Hz, 2H), 7.36-7.32 (m, 4H), 7.27-7.22 (m, 2H), 7.18-7.15 (m, 2H), 3.80 (s, 3H), 3.37-3.28 (m, 3H), 3.05 (dd, J = 16.0, 3.6 Hz, 1H), 2.79 (dd, J = 11.2, 4.8 Hz, 1H), 2.27 (dd, J = 11.2, 7.2 Hz, 1H). 1.47 (s, 3H), 1.40 (s, 3H); ^{19}F NMR (376 MHz, $CDCl_3$): δ = -96.41 (s); ^{13}C NMR (100 MHz, $CDCl_3$): δ = 176.4, 172.1, 158.8 (d, J = 256.2 Hz), 142.2 (d, J = 5.5 Hz), 138.7, 128.8 (d, J = 3.4 Hz), 128.2, 128.0, 127.1, 126.9, 126.0, 114.2 (d, J = 18.8 Hz), 85.0, 61.9, 52.2, 48.1, 46.2 (d, J =28.9 Hz), 41.6 (d, J = 3.8 Hz), 39.6 (d, J = 1.7 Hz), 30.9 (d, J = 4.4 Hz), 26.5, 24.5 ppm; HRMS (ESI) calcd for $C_{26}H_{26}FO_4$ $[M+H]^+$ 421.1810, found 421.1817.

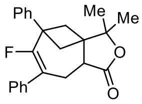

6-Fluoro-3,3-dimethyl-5,7-diphenyl-4,5,8,8a-tetrahydro-1*H*,3*H*-3a,5-methanocyclohepta[c]furan-1-one (**6ka'**): White solid (34%, 24.7 mg); m.p.: 206-207 °C; R_f = 0.4 (petroleum ether/EtOAc = 5/1); ^{1}H NMR (400 MHz, $CDCl_3$): δ = 7.38-7.34 (m, 2H), 7.32-7.30 (m, 4H), 7.27-7.25 (m, 1H), 7.23-7.20 (m, 3H), 3.32-3.28 (m, 1H), 3.26-3.14 (m, 2H), 3.07-3.02 (m, 1H), 2.70 (d, J = 10.0 Hz, 1H), 2.60-2.56 (m, 1H), 2.45-2.41 (m, 1H), 1.36 (s, 3H), 1.34 (s, 3H); ^{19}F NMR (376 MHz, $CDCl_3$): δ = -96.32 (s); ^{13}C NMR (100 MHz, $CDCl_3$): δ = 176.2, 159.6 (d, J = 257.4 Hz), 143.6 (d, J = 5.7 Hz), 138.3, 128.3 (d, J = 3.8 Hz), 128.0, 127.0, 126.8, 126.3, 113.5 (d, J = 16.7 Hz), 85.3, 48.3, 47.2, 44.7 (d, J = 28.0 Hz), 43.3 (d, J = 3.6 Hz), 34.6, 28.9 (d, J = 4.9 Hz), 23.6, 23.2 ppm; HRMS (ESI) calcd for $C_{24}H_{24}FO_2$ $[M+H]^+$ 363.1755, found 363.1763.

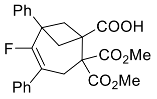

5-Fluoro-2,2-bis(methoxycarbonyl)-4,6-diphenylbicyclo[4.1.1]oct-4-ene-1-carboxylic acid (**7la**): White solid (88%, 87.7 mg); m.p.: 166-167 °C; ^{1}H NMR (400 MHz, DMSO-d6): δ = 12.92 (s, 1H), 7.39-7.31 (m, 6H), 7.27-7.21 (m, 4H), 3.66 (s, 6H), 3.40 (d, *J* = 4.8 Hz, 2H), 3.17-3.13 (m, 2H), 3.01-2.96 (m, 2H); ^{19}F NMR (376 MHz, DMSO-d6): δ = -98.43 (s); ^{13}C NMR (100 MHz, DMSO-d6): δ = 174.7, 171.3, 160.5 (d, *J* = 257.6 Hz), 144.1 (d, *J* = 4.9 Hz), 139.1, 129.0, 128.7, 128.6, 127.5, 127.1, 126.7, 113.2 (d, *J* = 17.7 Hz), 62.8, 53.0, 48.5, 45.1 (d, *J* = 27.5 Hz), 42.0, 37.0 (d, *J* = 3.6 Hz) ppm; HRMS (ESI) calcd for $C_{25}H_{24}FO_6$ $[M+H]^+$ 439.1551 found 439.1542.

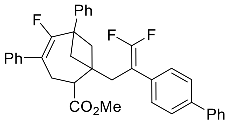

Methyl 1-(2-([1,1'-biphenyl]-4-yl)-3,3-difluoroallyl)-5-fluoro-4,6-diphenylbicyclo[4.1.1]oct-4-ene-2-carboxylate (**8la**): Colorless oil (75%, 42.3 mg); R_f = 0.3 (petroleum ether/EtOAc = 10/1); ^{1}H NMR (400 MHz, $CDCl_3$): δ = 7.57-7.50 (m, 4H), 7.42-7.38 (m, 2H), 7.33-7.28 (m, 3H), 7.25-7.09 (m, 8H), 6.99-6.96 (m, 2H), 3.71 (s, 3H), 3.12-3.07 (m, 2H), 3.01 (dt, *J* = 16.8, 5.2 Hz, 1H), 2.84 (dt, *J* = 16.8, 4.0 Hz, 1H), 2.69-2.63 (m, 2H), 2.58-2.54 (m, 1H), 2.42 (d, *J* = 11.2 Hz, 1H), 2.08 (dd, *J* = 12.0, 4.4 Hz, 1H); ^{19}F NMR (376 MHz, $CDCl_3$): δ = -89.23 (d, *J* = 39.1 Hz), -89.91 (d, *J* = 38.7 Hz), -99.17 (s); ^{13}C NMR (100 MHz, $CDCl_3$): δ = 174.1, 159.4 (d, *J* = 256.7 Hz), 154.5, (t, *J* = 288.3 Hz), 144.6 (d, *J* = 5.4 Hz), 140.4, 140.3, 139.3, 132.8 (dd, *J* = 4.4, 2.3 Hz), 129.0 (t, *J* = 2.7 Hz), 128.8, 128.6 (d, *J* =3.3 Hz), 128.0, 127.9, 127.5, 127.1, 127.0, 126.6, 126.3, 126.25, 114.5 (d, *J* = 17.1 Hz), 89.9 (dd, *J* = 21.0, 14.6 Hz), 51.6, 47.9, 45.9 (d, *J* = 27.0 Hz), 43.3 (d, *J* = 3.2 Hz), 42.1, 40.9, 39.8, 31.1 (d, *J* = 4.8 Hz) ppm; HRMS (ESI) calcd for $C_{37}H_{32}F_3O_2$ $[M+H]^+$ 565.2349, found 565.2333.

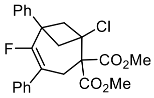

Dimethyl 1-chloro-5-fluoro-4,6-diphenylbicyclo[4.1.1]oct-4-ene-2,2-dicarboxylate (**9la**): Colorless oil (52%, 22.3 mg); R_f = 0.3 (petroleum ether/EtOAc = 10/1); ^{1}H NMR (400 MHz, $CDCl_3$): δ = 7.34 (d, *J* = 8.4 Hz, 2H), 7.30-7.24 (m, 3H), 7.21-7.18 (m, 2H), 7.16-7.12 (m, 1H), 7.07 (d, *J* = 7.2 Hz, 2H), 3.78 (s, 6H), 3.53-3.51 (m, 3H), 3.49-3.46 (m, 1H), 3.20-3.16 (m, 2H); ^{19}F NMR (376 MHz, $CDCl_3$): δ = -98.45 (s); ^{13}C NMR (100 MHz, $CDCl_3$): δ = 169.7, 159.1 (d, *J* = 258.9 Hz), 142.7 (d, *J* = 4.7 Hz), 137.9, 128.6 (d, *J* = 3.6 Hz), 128.4, 128.1, 127.1, 127.0, 126.3, 113.1 (d, *J* = 17.6 Hz), 67.4, 63.7, 52.9, 49.9, 44.7 (d, *J* = 28.6 Hz), 36.3 (d, *J* = 4.2 Hz) ppm; HRMS (ESI) calcd for $C_{24}H_{22}ClNaFO_4$ $[M+Na]^+$ 451.1083, found 451.1087.

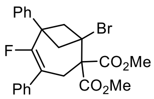

Dimethyl 1-bromo-5-fluoro-4,6-diphenylbicyclo[4.1.1]oct-4-ene-2,2-dicarboxylate (**10la**): Colorless oil (55%, 26.1 mg); R_f = 0.3 (petroleum ether/EtOAc = 10/1); ^{1}H NMR (400 MHz, $CDCl_3$): δ = 7.34 (d, *J* = 8.4 Hz, 2H), 7.30-7.23 (m, 3H), 7.22-7.20 (m, 2H), 7.15-7.12 (m, 1H), 7.06 (d, *J* = 6.8 Hz, 2H), 3.78 (s, 6H), 3.62-3.57 (m, 2H), 3.53 (d, *J* = 4.4 Hz, 2H), 3.44-3.40 (m, 2H); ^{19}F NMR (376 MHz, $CDCl_3$): δ = -98.78 (s); ^{13}C NMR (100 MHz, $CDCl_3$): δ = 169.8, 159.2 (d, *J* = 258.5 Hz), 142.5 (d, *J* = 4.9 Hz), 137.9, 128.6 (d, *J* = 3.6 Hz), 128.4, 128.1, 127.1, 127.0, 126.2, 113.2 (d, *J* = 18.0 Hz), 67.9, 54.7, 52.9, 51.1, 47.0 (d, *J* = 28.2 Hz), 36.2 (d, *J* = 4.1 Hz) ppm; HRMS (ESI) calcd for $C_{24}H_{22}BrNaFO_4$ $[M+Na]^+$ 495.0578, found 495.0582.

10 Single-crystal X-ray diffraction data for 3aa and 5aa

Preparation of the single crystals of **3aa**: 30.0 mg of pure **3aa** was dissolved in the combined solvents of MeOH and CH_2Cl_2 (2.0 mL, v/v = 1:1) at room temperature. The bottle was sealed by a piece of plastic film with several tiny holes, thus allowing the slow solvent evaporation at 0 ^oC. After about two days, several small crystals were observed at the bottom of the bottle. The crystals were collected and subjected to the single crystal X-ray diffraction analysis for the determination of the structure of **3aa**.

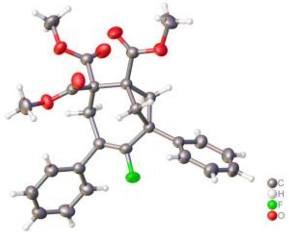

X-ray Structure of **3aa** (CCDC 2382103)
50% probability ellipsoids

Empirical formula	$C_{26}H_{25}FO_6$
Formula weight	452.46
Temperature/K	300.00
Crystal system	triclinic
Space group	P-1
a/Å	7.28910(10)
b/Å	9.7884(2)
c/Å	15.6447(3)
α/°	88.0640(10)
β/°	84.5890(10)
γ/°	80.1740(10)
Volume/Å^3	1094.77(3)
Z	2
ρ_{calc}g/cm^3	1.373
μ/mm^{-1}	0.854
F(000)	476.0
Crystal size/mm^3	0.2 × 0.2 × 0.2
Radiation	CuKα (λ = 1.54184)
2Θ range for data collection/°	5.676 to 133.248
Index ranges	$-8 \le h \le 8$, $-11 \le k \le 11$, $-18 \le l \le 18$
Reflections collected	19597
Independent reflections	3861 [R_{int} = 0.0462, R_{sigma} = 0.0430]
Data/restraints/parameters	3861/0/302
Goodness-of-fit on F^2	1.030
Final R indexes [I>=2σ (I)]	R_1 = 0.0474, wR_2 = 0.1264

Final R indexes [all data]	R_1 = 0.0491, wR_2 = 0.1282
Largest diff. peak/hole / e Å^{-3}	0.32/-0.25

Preparation of the single crystals of **5aa**: 20.0 mg of pure **5aa** was dissolved in the combined solvents of MeOH and CH_2Cl_2 (2.0 mL, v/v = 1:1) at room temperature. The bottle was sealed by a piece of plastic film with several tiny holes, thus allowing the slow solvent evaporation at 0 ^{o}C. After about two days, several small crystals were observed at the bottom of the bottle. The crystals were collected and subjected to the single crystal X-ray diffraction analysis for the determination of the structure of **5aa**.

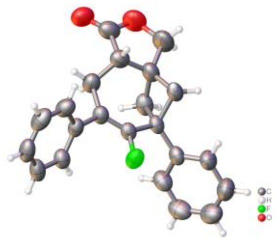

X-ray Structure of **5aa** (CCDC 2382195)
50% probability ellipsoids

Empirical formula	$C_{22}H_{19}FO_2$
Formula weight	334.37
Temperature/K	301.00
Crystal system	orthorhombic
Space group	$Pna2_1$
a/Å	11.4103(2)
b/Å	16.6264(3)
c/Å	9.23370(10)
α/°	90
β/°	90
γ/°	90
Volume/Å^3	1751.75(5)
Z	4
ρ_{calc}g/cm^3	1.268
μ/mm^{-1}	0.707
F(000)	704.0
Crystal size/mm^3	0.2 × 0.2 × 0.2
Radiation	CuKα (λ = 1.54178)
2Θ range for data collection/°	9.4 to 136.506
Index ranges	$-13 \leq h \leq 13$, $-19 \leq k \leq 20$, $-11 \leq l \leq 10$
Reflections collected	12016
Independent reflections	3049 [R_{int} = 0.0336, R_{sigma} = 0.0316]
Data/restraints/parameters	3049/73/226

Goodness-of-fit on F^2	1.027
Final R indexes [I>=2σ (I)]	R_1 = 0.0355, wR_2 = 0.0949
Final R indexes [all data]	R_1 = 0.0376, wR_2 = 0.0972
Largest diff. peak/hole / e $Å^{-3}$	0.15/-0.15

11 References

1 (a) K. Livingstone, K. Siebold, S. Meyer, V. M. Heras, C. G. Daniliuc and R. Gilmour, *ACS Catal.*, 2022, **12**, 14507–14516; (b) R. Guo, Y.-C. Chang, L. Herter, C. Salome, S. E. Braley, T. C. Fessard and M. K. Brown, *J. Am. Chem. Soc*., 2022, **144**, 7988–7994; (c) H.-M. Wang, H.-L. Shao, A. Das, S. Dutta, H. T. Chan, C. Daniliuc, K. N. Houk and F. Glorius, *Science*, 2023, **381**, 75–81; (d) K. Zhang, S. Tian, W. Li, X. Yang, X.-H. Duan, L.-N. Guo and P. Li, *Org. Lett.*, 2024, **26**, 5482−5487.

2 (a) M. Xu, Z. Wang, Z. Sun, Y. Ouyang, Z. Ding, T. Yu, L. Xu and P. Li, *Angew. Chem.*, *Int. Ed.*, 2022, **61**, e20221450; (b) K. Dhake, K. J. Woelk, J. Becica, A. Un, S. E. Jenny and D. C. Leitch, *Angew. Chem.*, *Int. Ed*., 2022, **61**, e202204719.

3 G.-X. Zhang, D.-S. Li, F. Xue, A.-F. Chen and H.-P. Deng, *Adv. Synth. Catal*., 2023, **365**, 4603–4608.

4 J. Tian, L. Zhou and *Chem. Sci.*, 2023, **14**, 6045–6051.

5 F. Chen, X. Xu, Y. He, G. Huang and S. Zhu, *Angew. Chem.*, *Int. Ed.*, 2020, **59**, 5398 –5402.

6 D. Wu, W. Kong , Y. Bao, D Zhao, Y. Li and G. Yin, *Nat. Catal*., 2023, **31**, 1030-1041.

7 M. Chen, Z.-J. Wu, J. Song and H.-C. Xu, *Angew. Chem.*, *Int. Ed*., 2022, **61**, e202115954.

8 C. S. Gravatt, L. Melecio-Zambrano and T. P. Yoon, *Angew. Chem.*, *Int. Ed*., 2021, **60**, 3989−3993.

9 L. Tang, Y. Xiao, F. Wu, J.-L. Zhou, T.-T. Xu and J.-J. Feng, *Angew. Chem.*, *Int. Ed*., 2023, **62**, e202310066.

10 Z. Ding, Z. Liu, Z. Wang, T. Yu, M. Xu, J. Wen, K. Yang, H. Zhang, L. Xu and P. Li, *J. Am. Chem. Soc*., 2022, **144**, 8870−8882.

11 J.-H. Zhang, H.-J. Miao, J.-Y. Li, W. Li, P. Ma, X.-H. Duan and L.-N. Guo, *Chem. Commun*., 2024, **60**, 8095–8098.

12 J. Qian, Y. Zhang, W. Zhao and P. Hu, *Chem. Commun*., 2024, **60**, 2764–2767.

12 ^{1}H NMR, ^{19}F NMR and ^{13}C NMR Spectra of Products

^{1}H NMR (400MHz, $CDCl_3$), ^{13}C NMR (100 MHz, $CDCl_3$) spectra of product **1n**

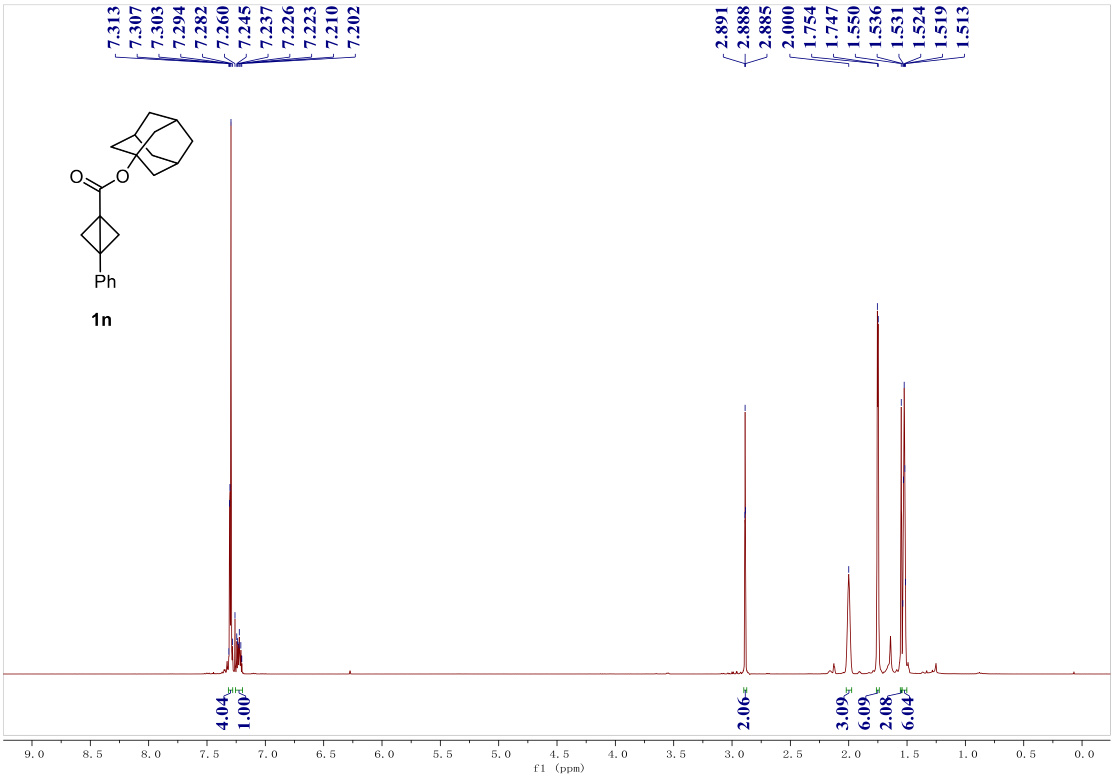

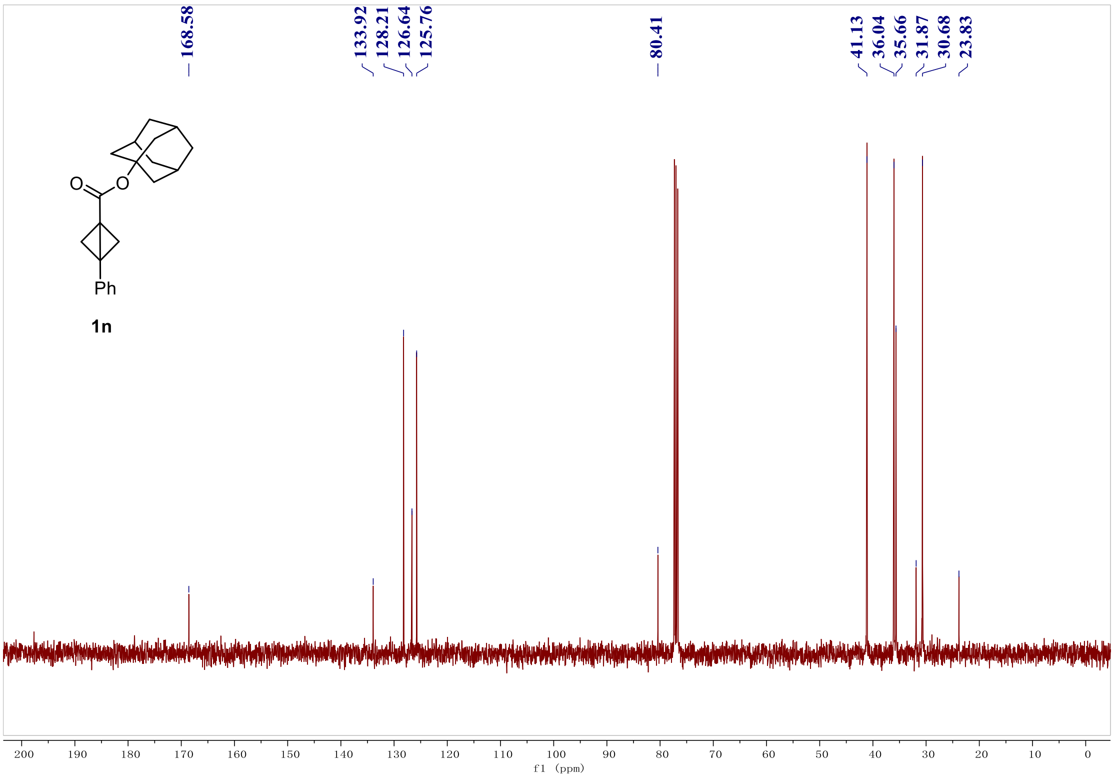

^{1}H NMR (400MHz, $CDCl_3$), ^{13}C NMR (100 MHz, $CDCl_3$) spectra of product **1r**

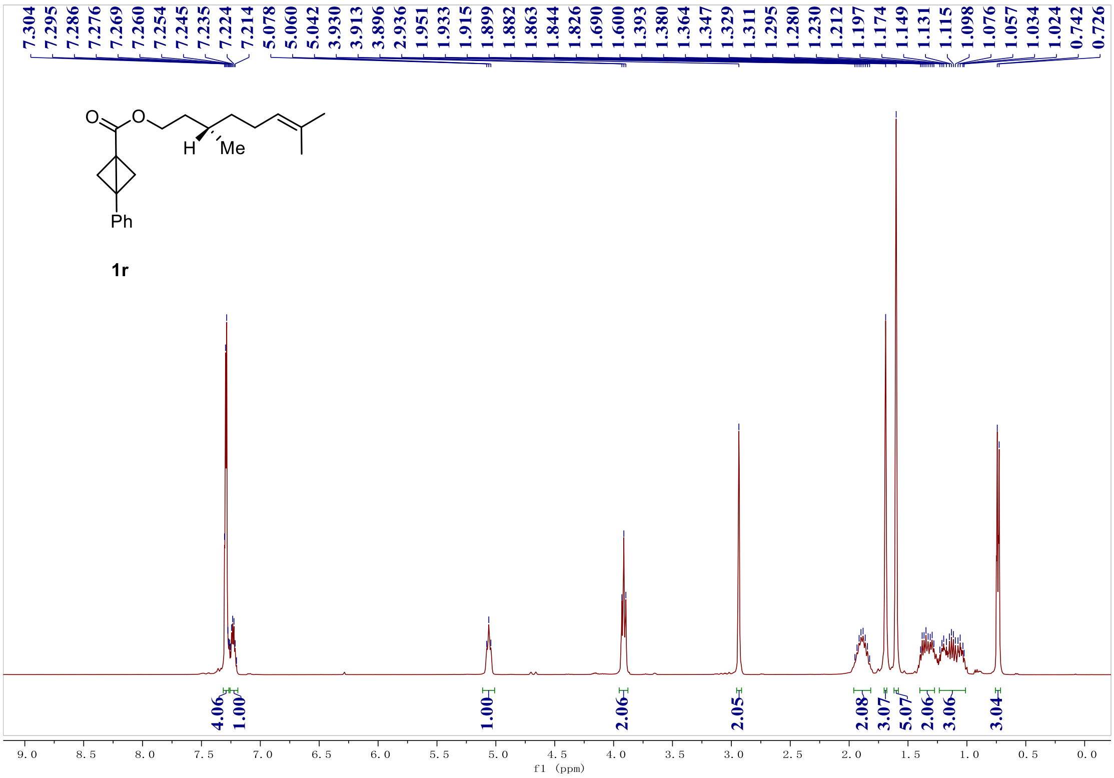

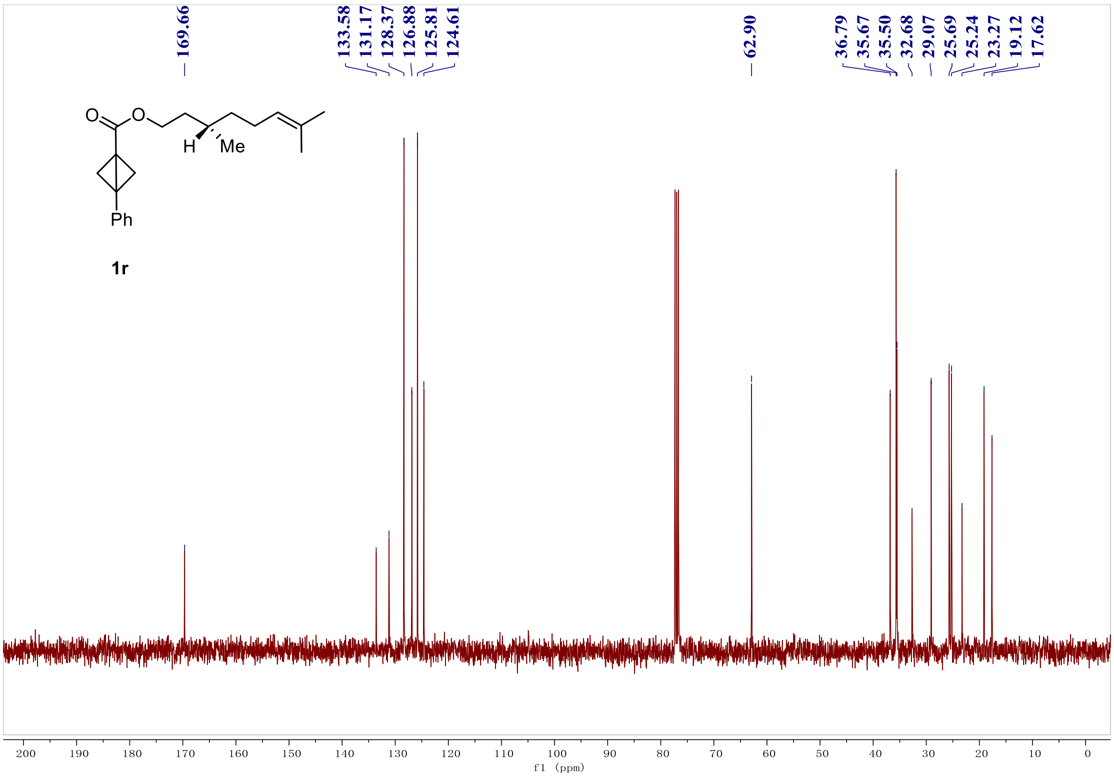

^{1}H NMR (400MHz, $CDCl_3$), ^{19}F NMR (376 MHz, $CDCl_3$) and ^{13}C NMR (100 MHz, $CDCl_3$) spectra of product **2b**

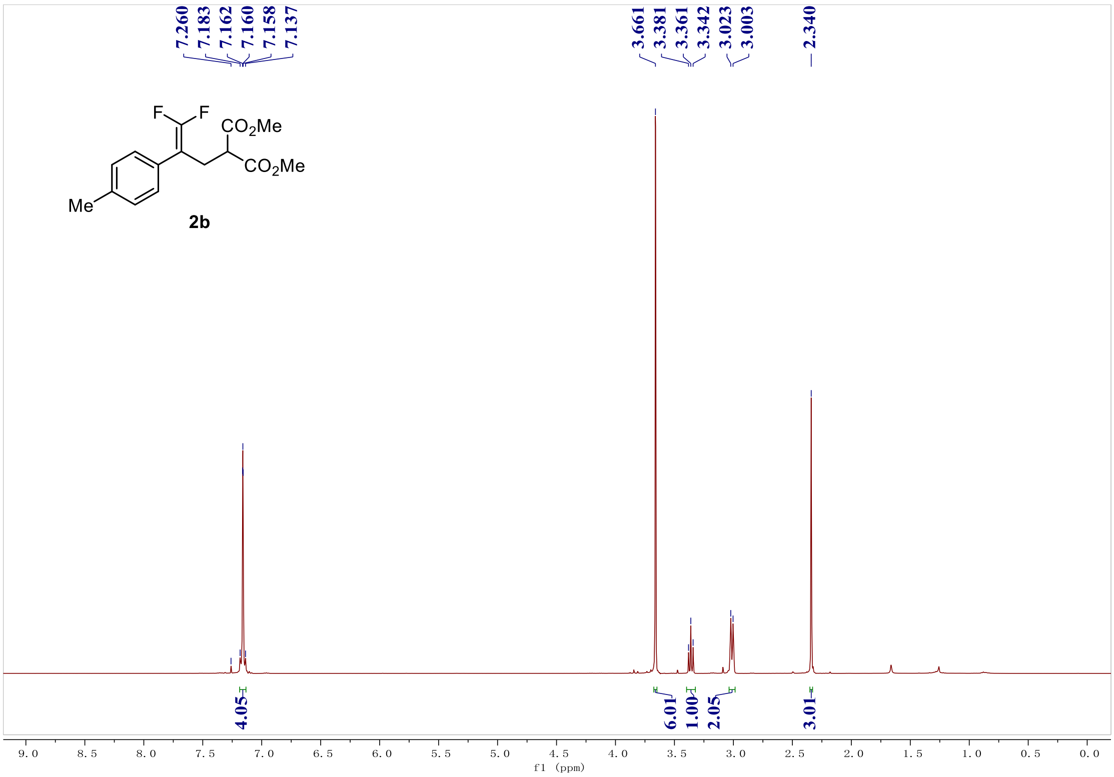

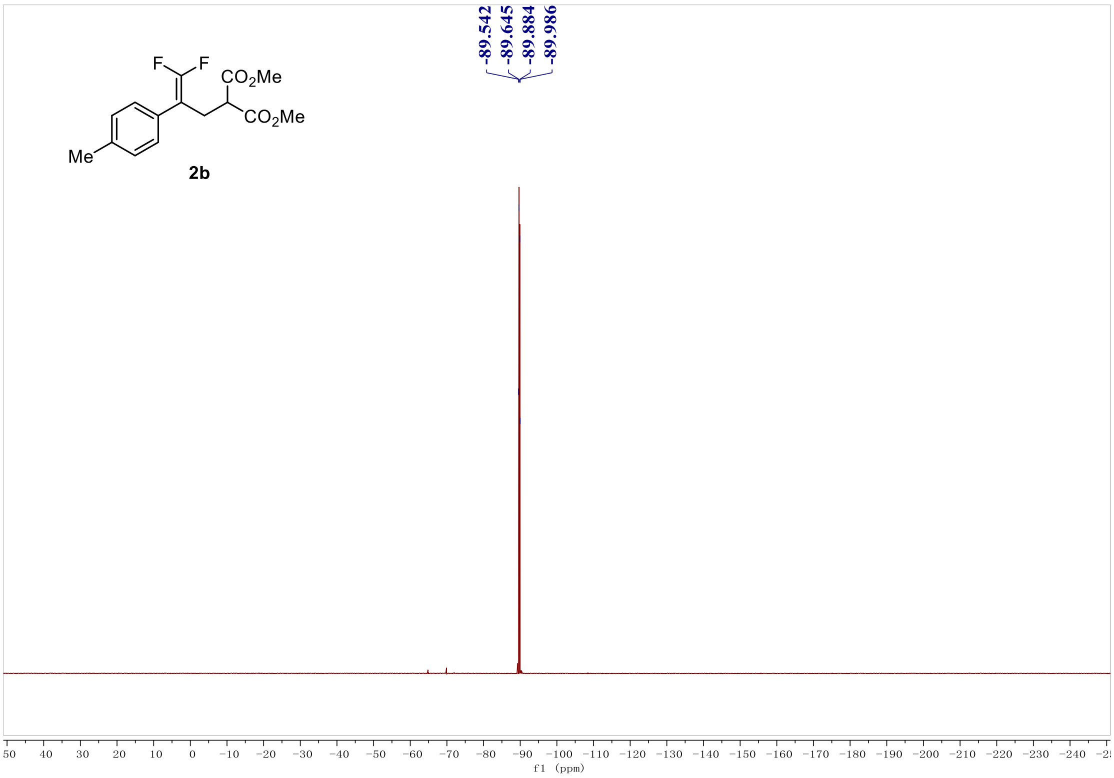

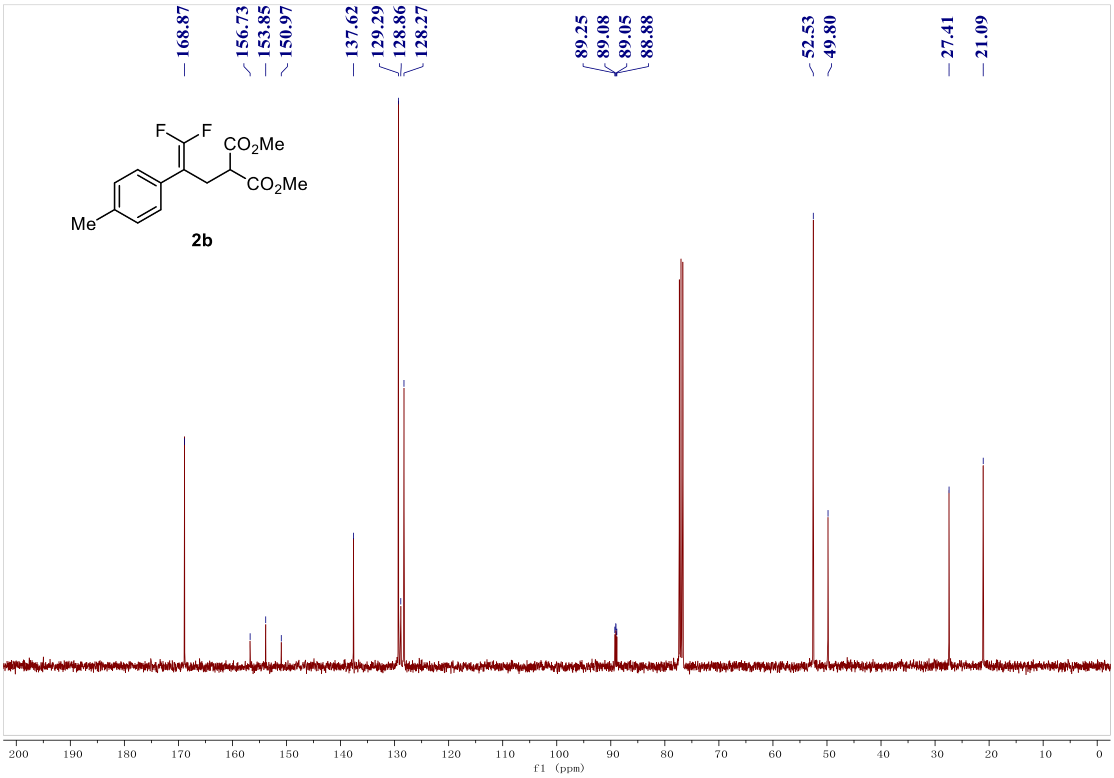

^{1}H NMR (400MHz, $CDCl_3$), ^{19}F NMR (376 MHz, $CDCl_3$) and ^{13}C NMR (100 MHz, $CDCl_3$) spectra of product **2c**

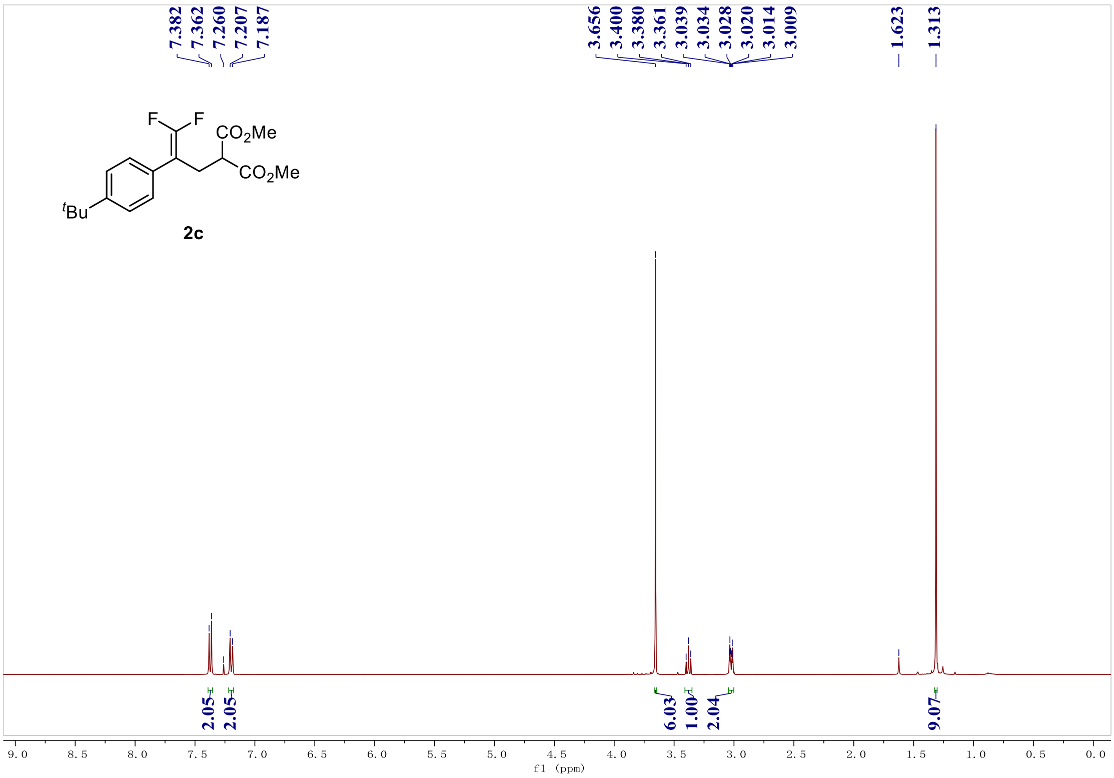

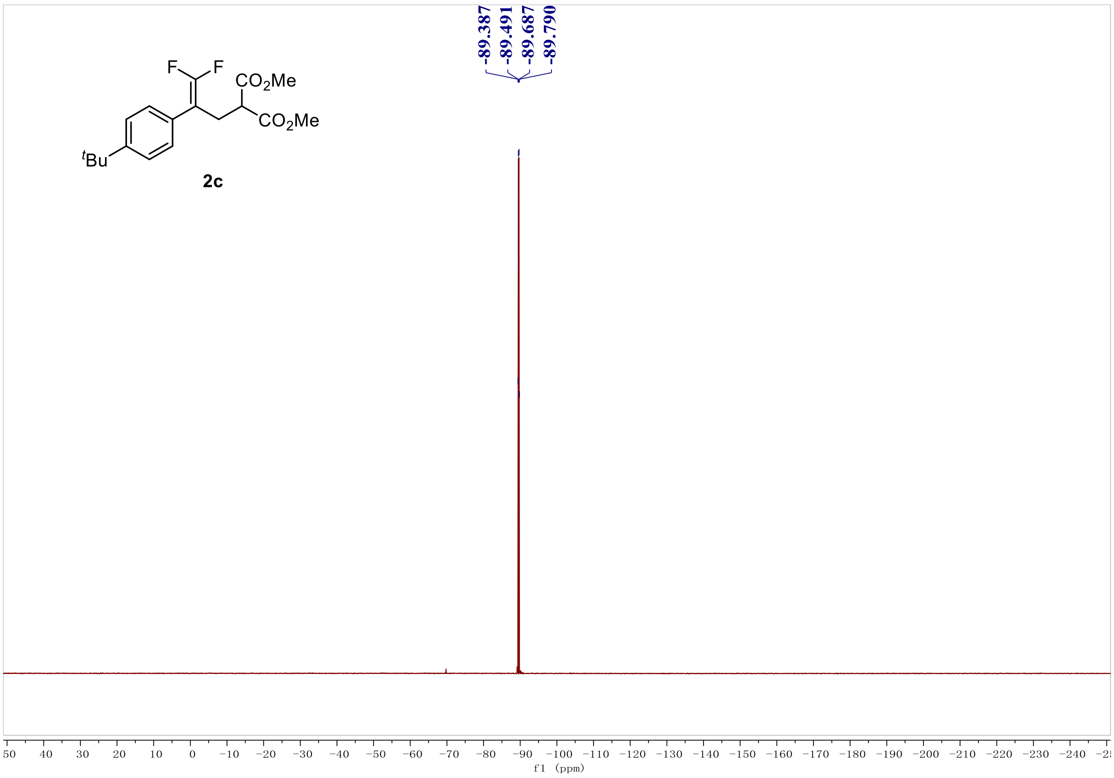
-89.387
-89.491
-89.687
-89.790
F
F
CO2Me
CO2Me
tBu
2c
50 40 30 20 10 0 −10 −20 −30 −40 −50 −60 −70 −80 −90 −100 −110 −120 −130 −140 −150 −160 −170 −180 −190 −200 −210 −220 −230 −240
f1 (ppm)

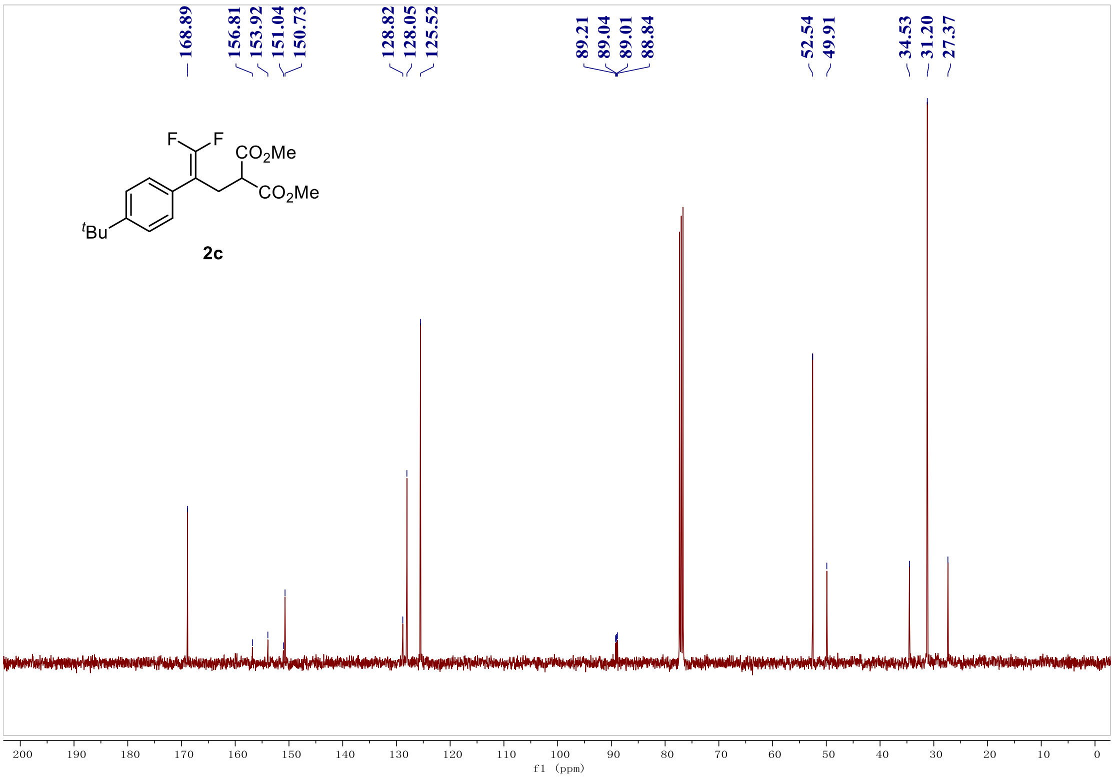
168.89
156.81
153.92
151.04
150.73
128.82
128.05
125.52
89.21
89.04
89.01
88.84
52.54
49.91
34.53
31.20
27.37
F
F
CO2Me
CO2Me
tBu
2c
200 190 180 170 160 150 140 130 120 110 100 90 80 70 60 50 40 30 20 10 0
f1 (ppm)

^{1}H NMR (400MHz, $CDCl_3$), ^{19}F NMR (376 MHz, $CDCl_3$) and ^{13}C NMR (100 MHz, $CDCl_3$) spectra of product **2f**

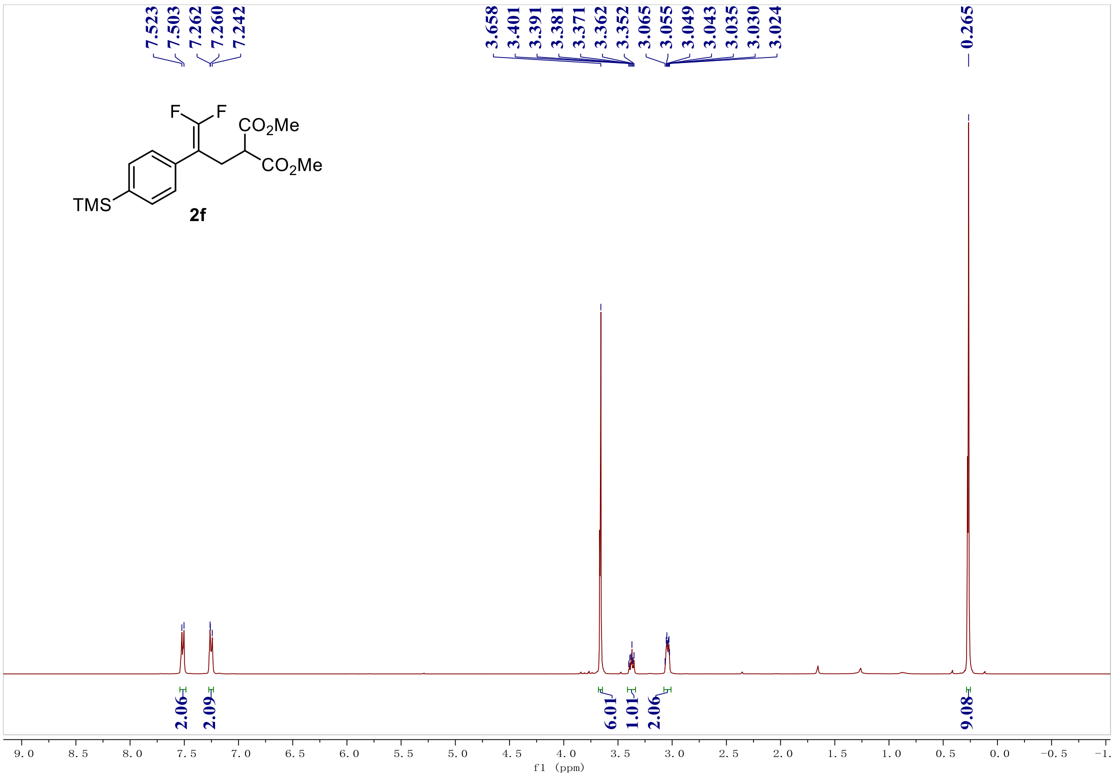

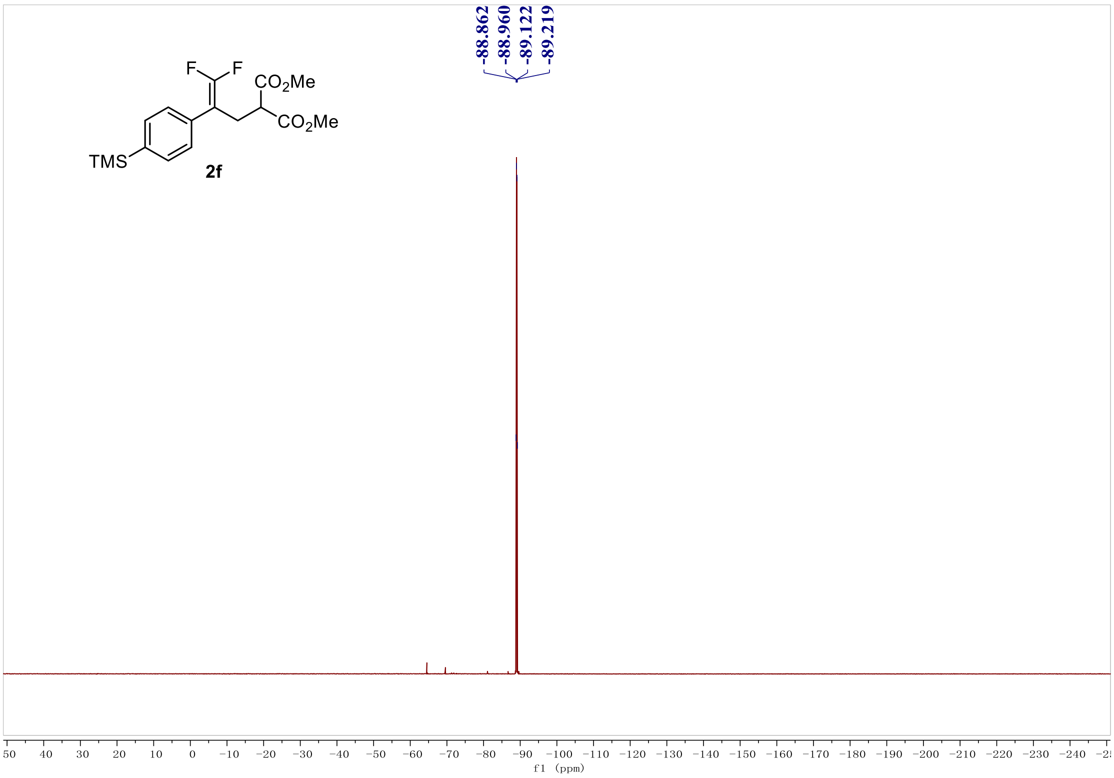

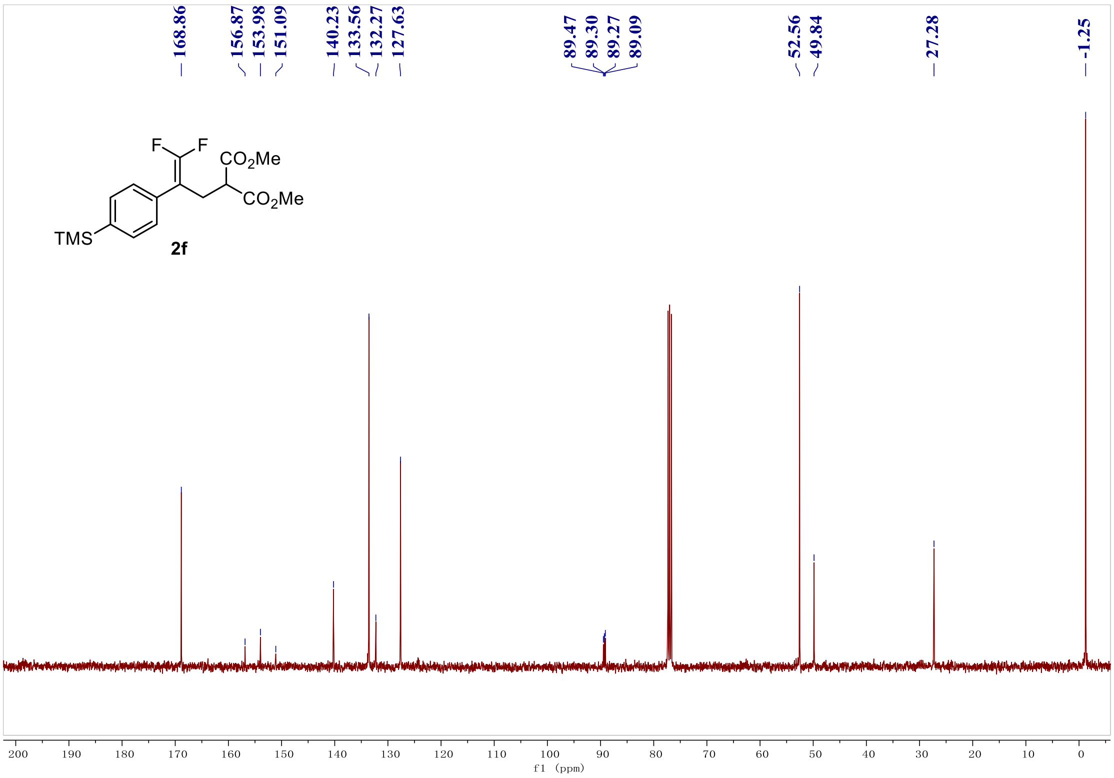

^{1}H NMR (400MHz, $CDCl_3$), ^{19}F NMR (376 MHz, $CDCl_3$) and ^{13}C NMR (100 MHz, $CDCl_3$) spectra of product **2g**

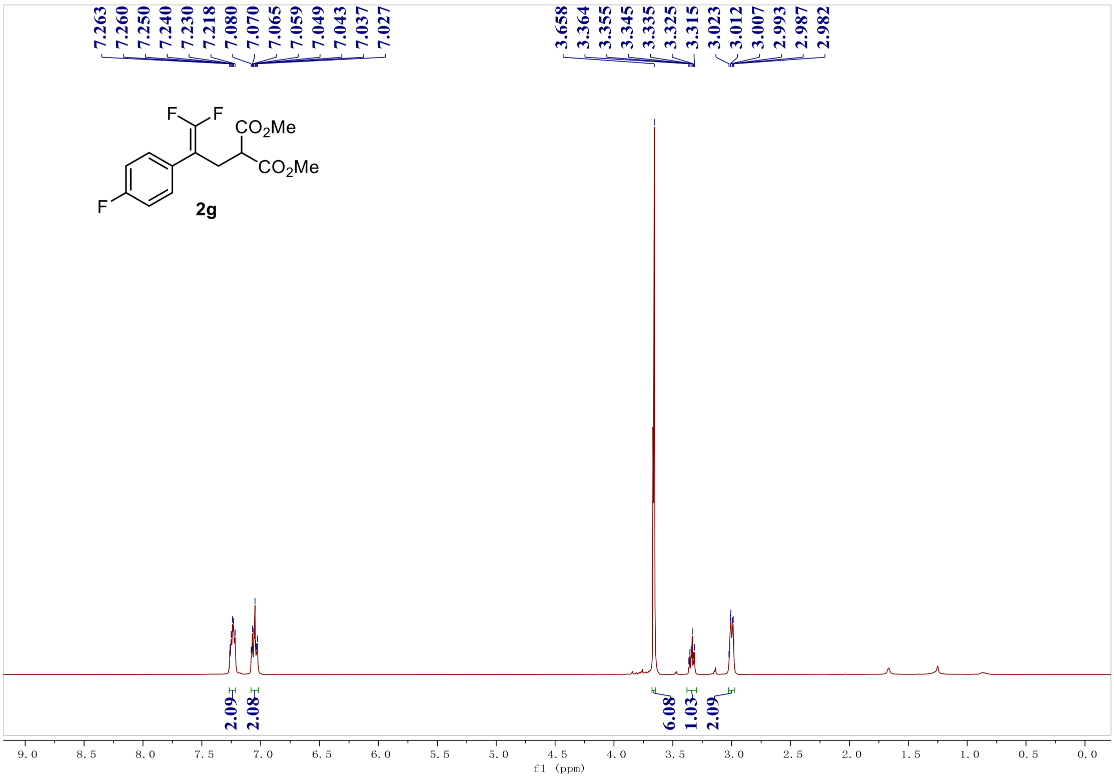

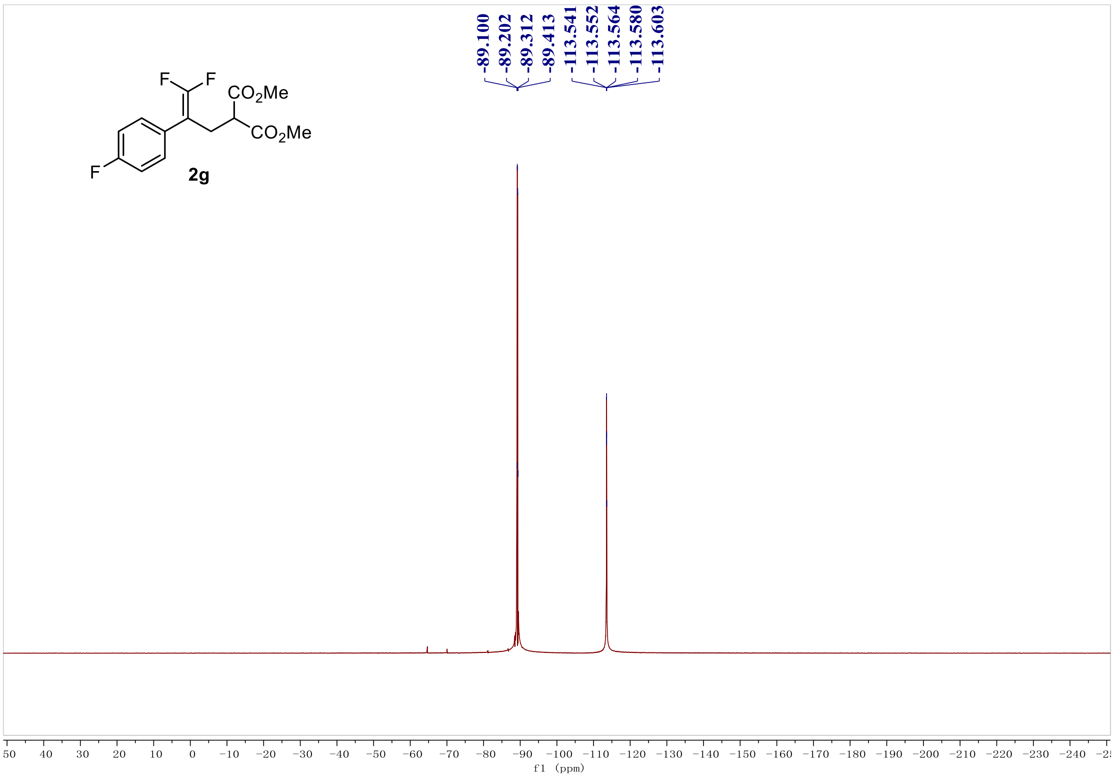
-89.100
-89.202
-89.312
-89.413
-113.541
-113.552
-113.564
-113.580
-113.603
F
F
CO2Me
CO2Me
F
2g
f1 (ppm)

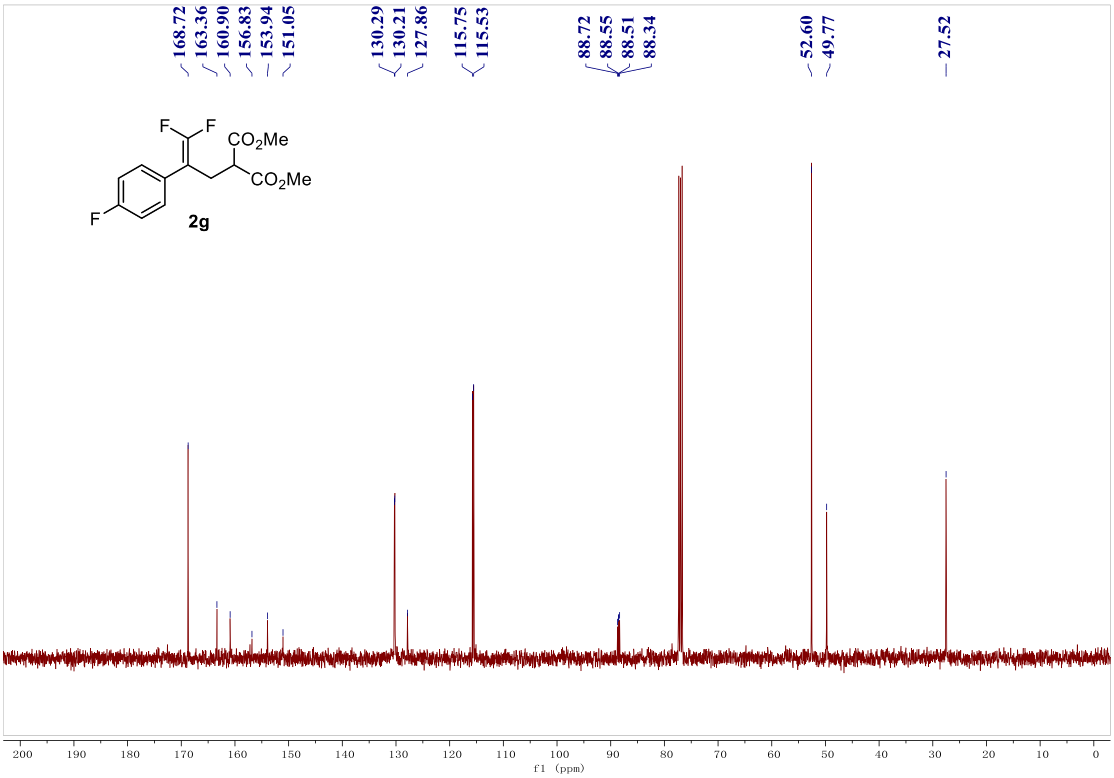
168.72
163.36
160.90
156.83
153.94
151.05
130.29
130.21
127.86
115.75
115.53
88.72
88.55
88.51
88.34
52.60
49.77
27.52
F
F
CO2Me
CO2Me
F
2g
f1 (ppm)

^{1}H NMR (400MHz, $CDCl_3$), ^{19}F NMR (376 MHz, $CDCl_3$) and ^{13}C NMR (100 MHz, $CDCl_3$) spectra of product **2k**

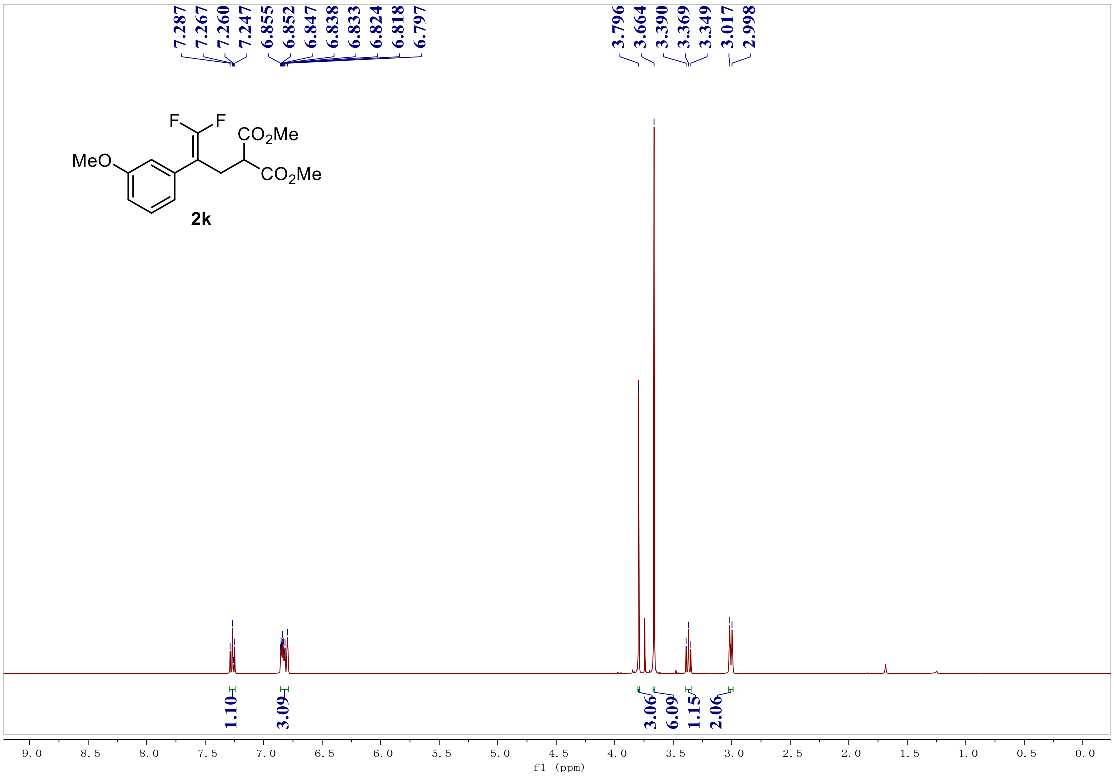

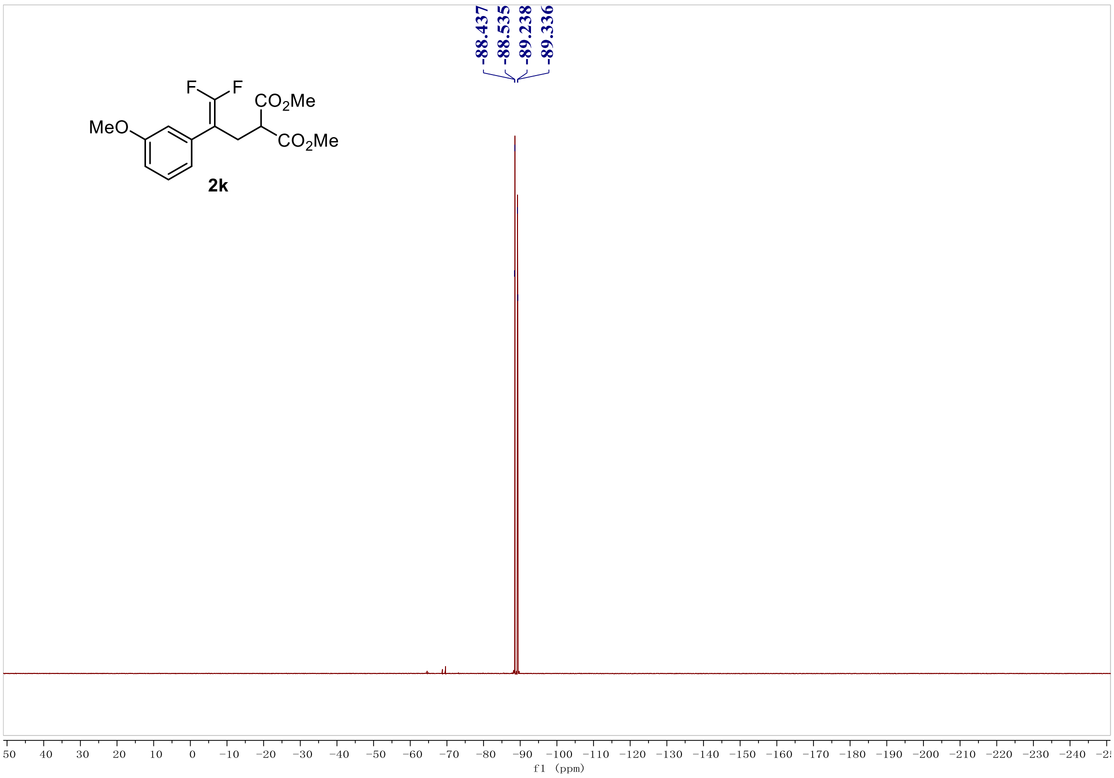

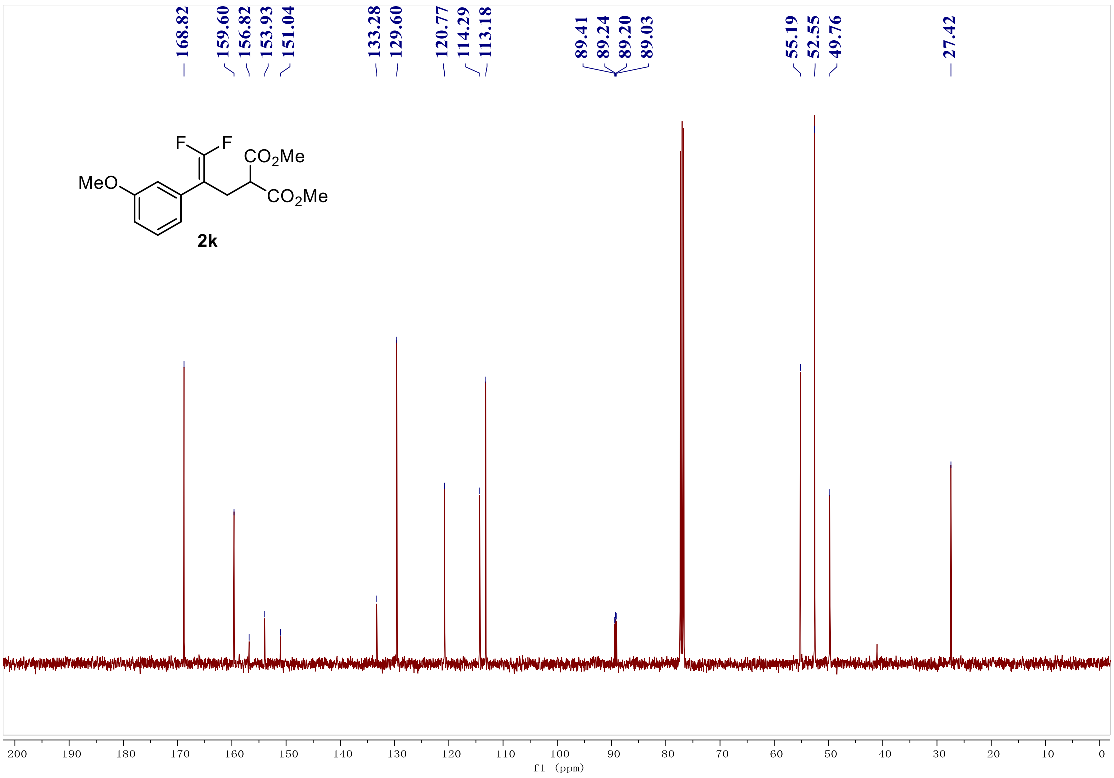

^{1}H NMR (400MHz, $CDCl_3$), ^{19}F NMR (376 MHz, $CDCl_3$) and ^{13}C NMR (100 MHz, $CDCl_3$) spectra of product **2l**

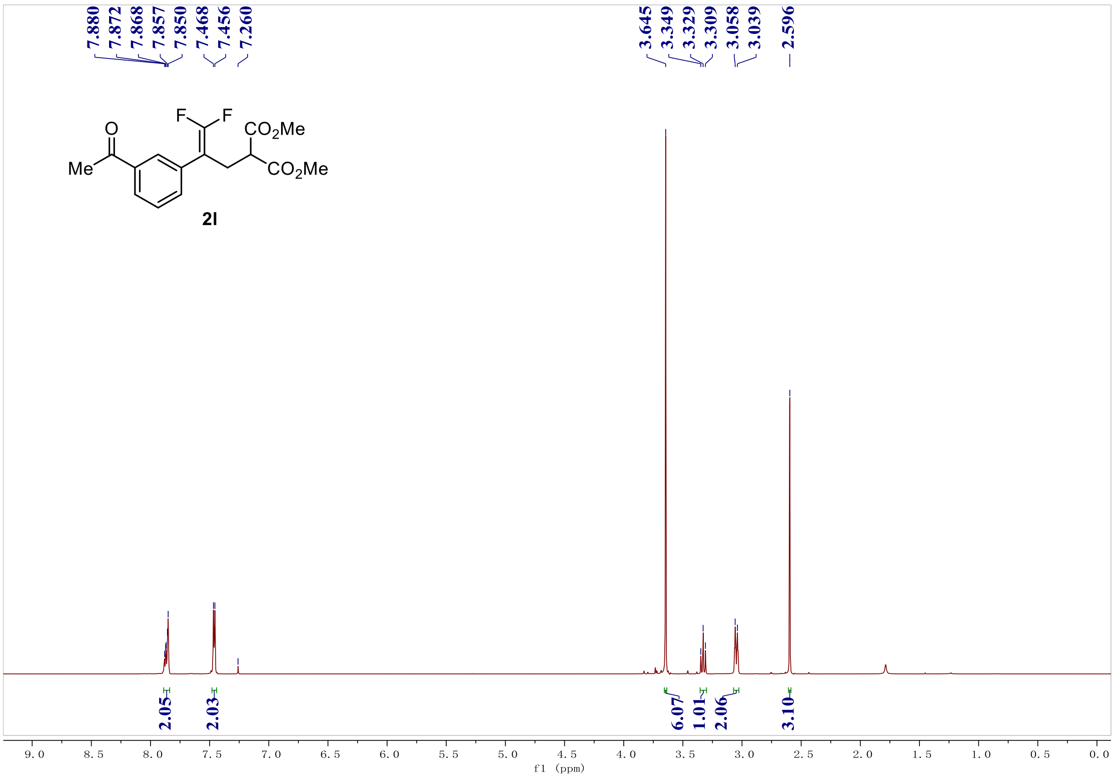

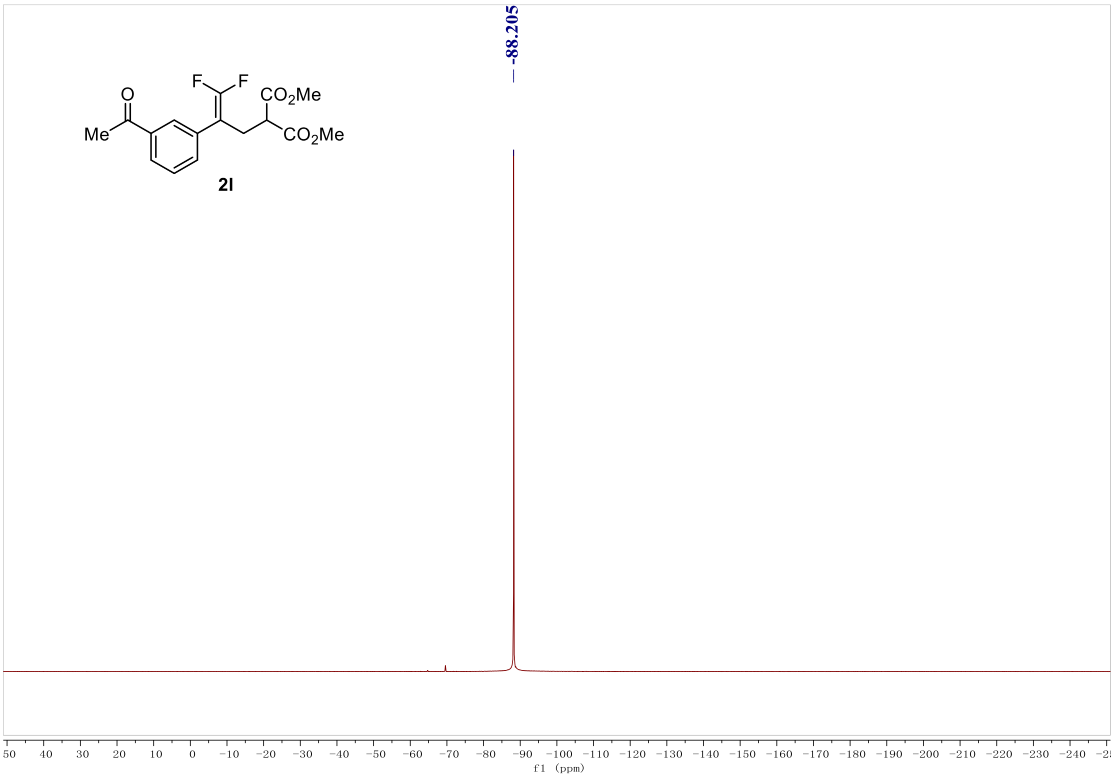

-88.205
O
Me
F
F
CO2Me
CO2Me
2l
f1 (ppm)

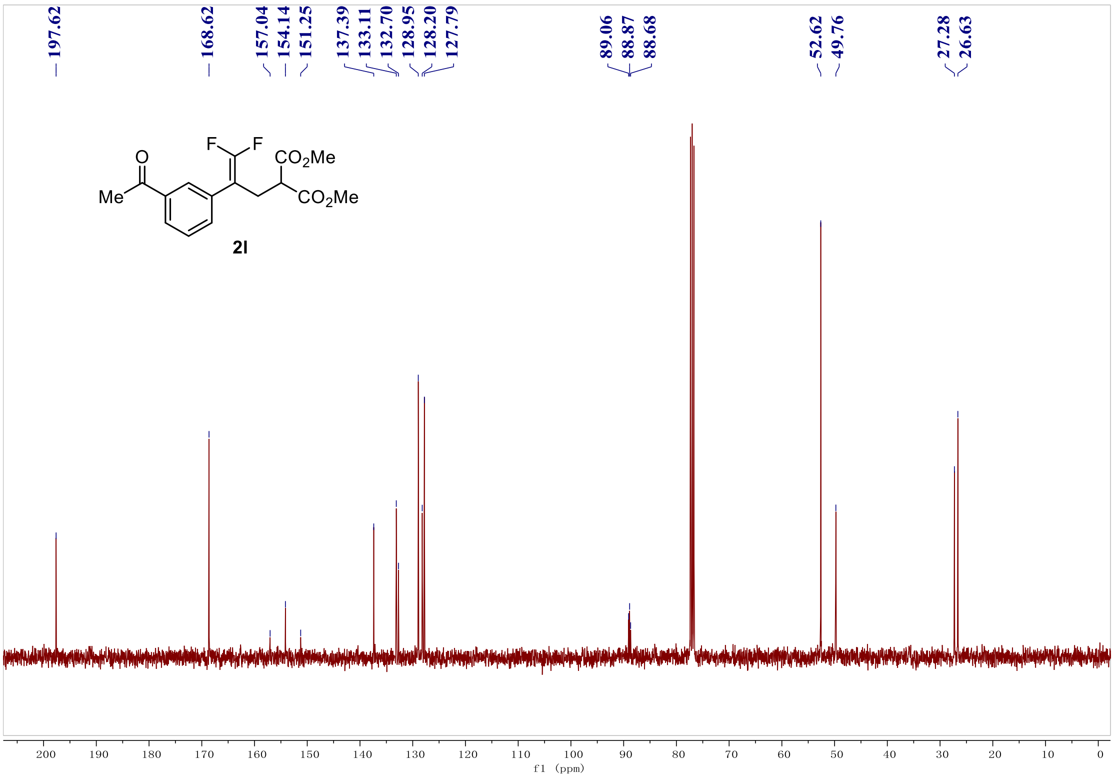

197.62
168.62
157.04
154.14
151.25
137.39
133.11
132.70
128.95
128.20
127.79
89.06
88.87
88.68
52.62
49.76
27.28
26.63
O
Me
F
F
CO2Me
CO2Me
2l
f1 (ppm)

^{1}H NMR (400MHz, $CDCl_3$), ^{19}F NMR (376 MHz, $CDCl_3$) and ^{13}C NMR (100 MHz, $CDCl_3$) spectra of product **2m**

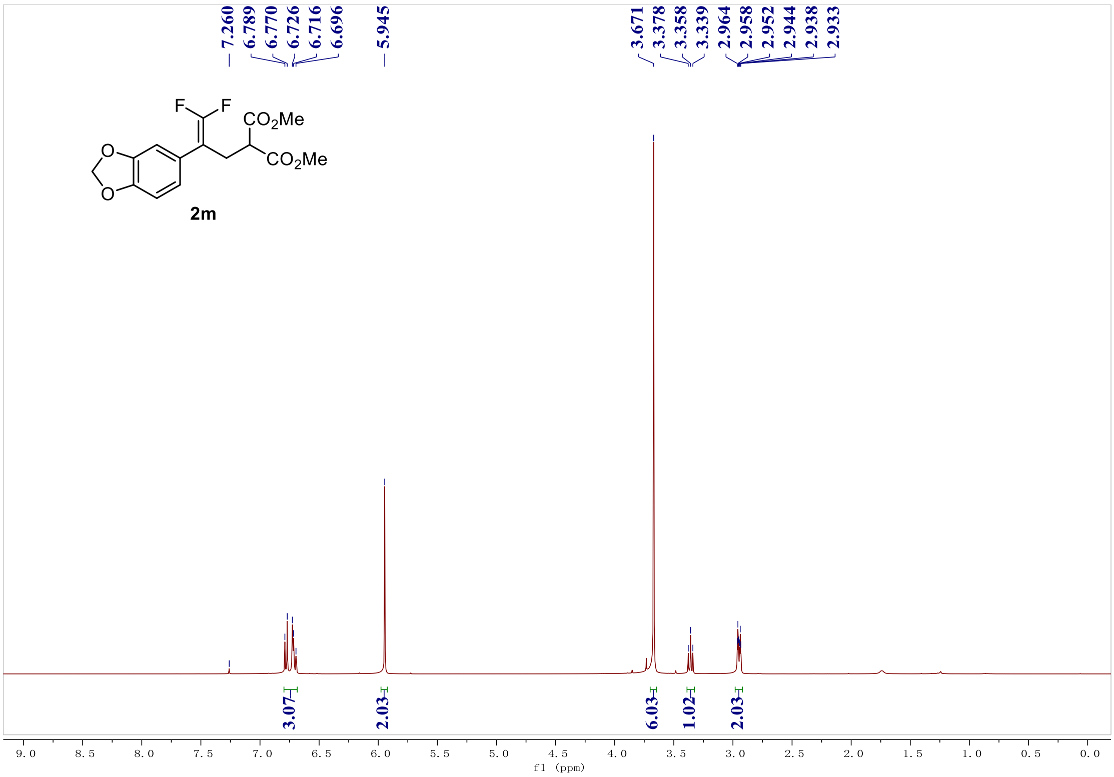

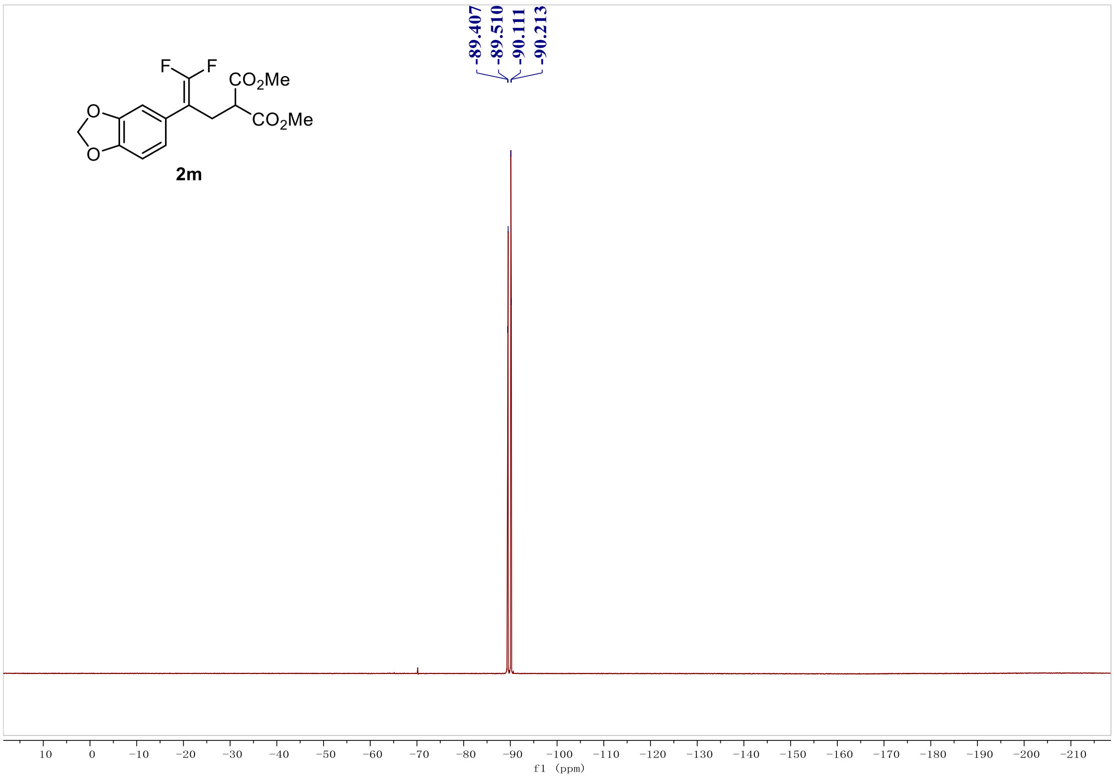

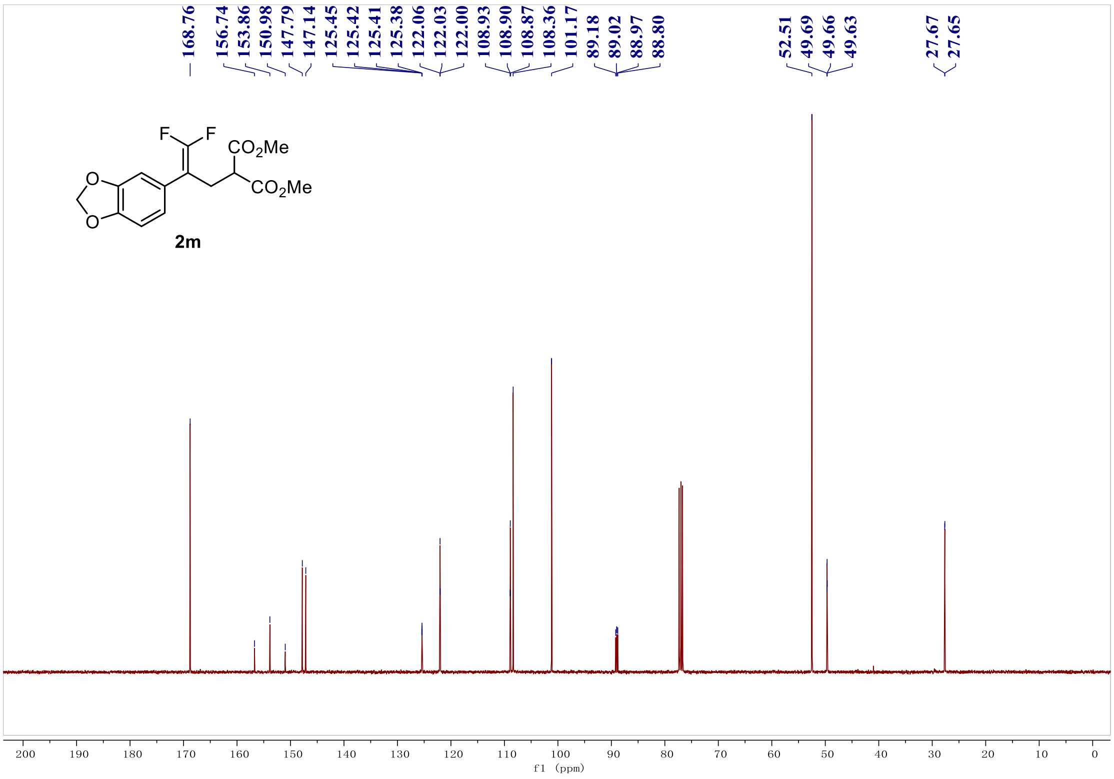

^{1}H NMR (400MHz, $CDCl_3$), ^{19}F NMR (376 MHz, $CDCl_3$) and ^{13}C NMR (100 MHz, $CDCl_3$) spectra of product **2o**

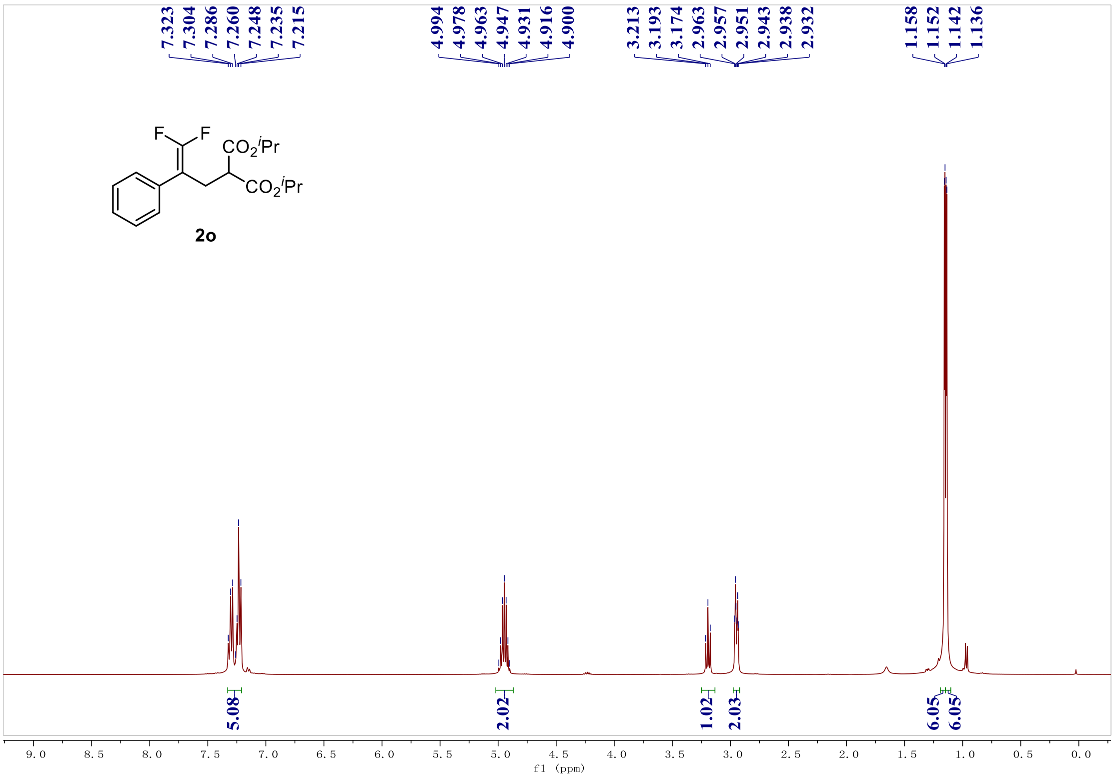

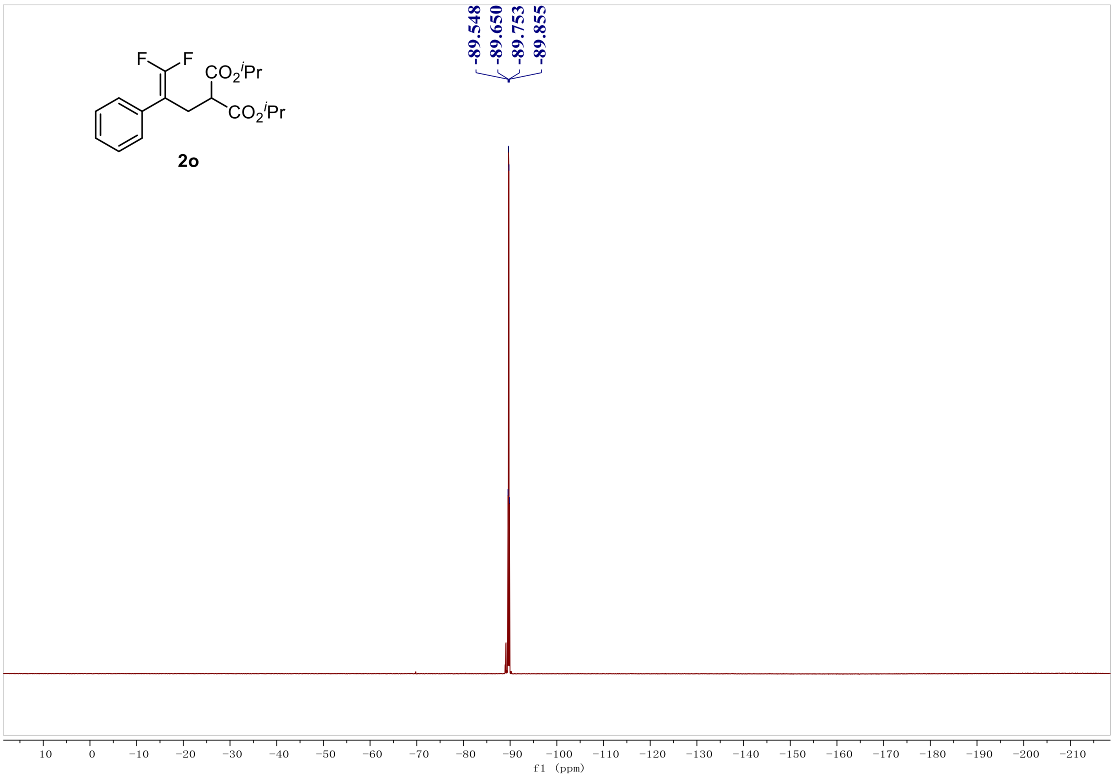
-89.548
-89.650
-89.753
-89.855
F
F
CO2iPr
CO2iPr
2o
10
0
−10
−20
−30
−40
−50
−60
−70
−80
−90
−100
−110
−120
−130
−140
−150
−160
−170
−180
−190
−200
−210
f1 (ppm)

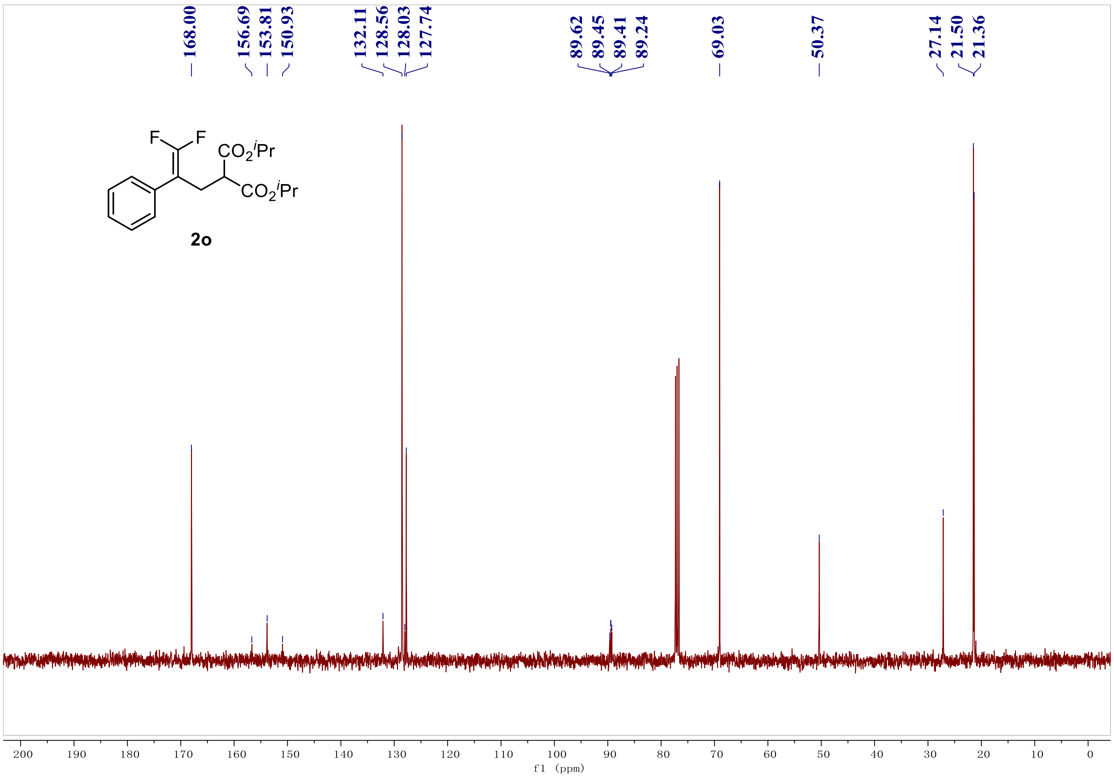
168.00
156.69
153.81
150.93
132.11
128.56
128.03
127.74
89.62
89.45
89.41
89.24
69.03
50.37
27.14
21.50
21.36
F
F
CO2iPr
CO2iPr
2o
200
190
180
170
160
150
140
130
120
110
100
90
80
70
60
50
40
30
20
10
0
f1 (ppm)

^{1}H NMR (400MHz, $CDCl_3$), ^{19}F NMR (376 MHz, $CDCl_3$) and ^{13}C NMR (100 MHz, $CDCl_3$) spectra of product **3aa**

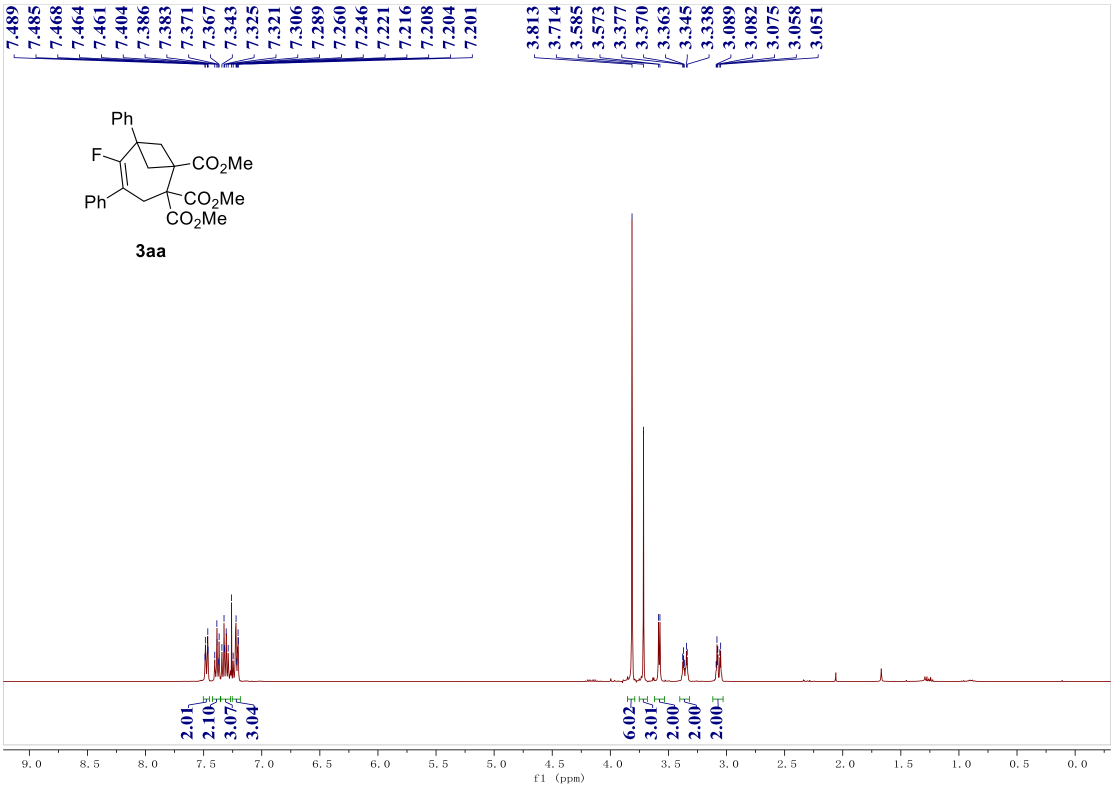

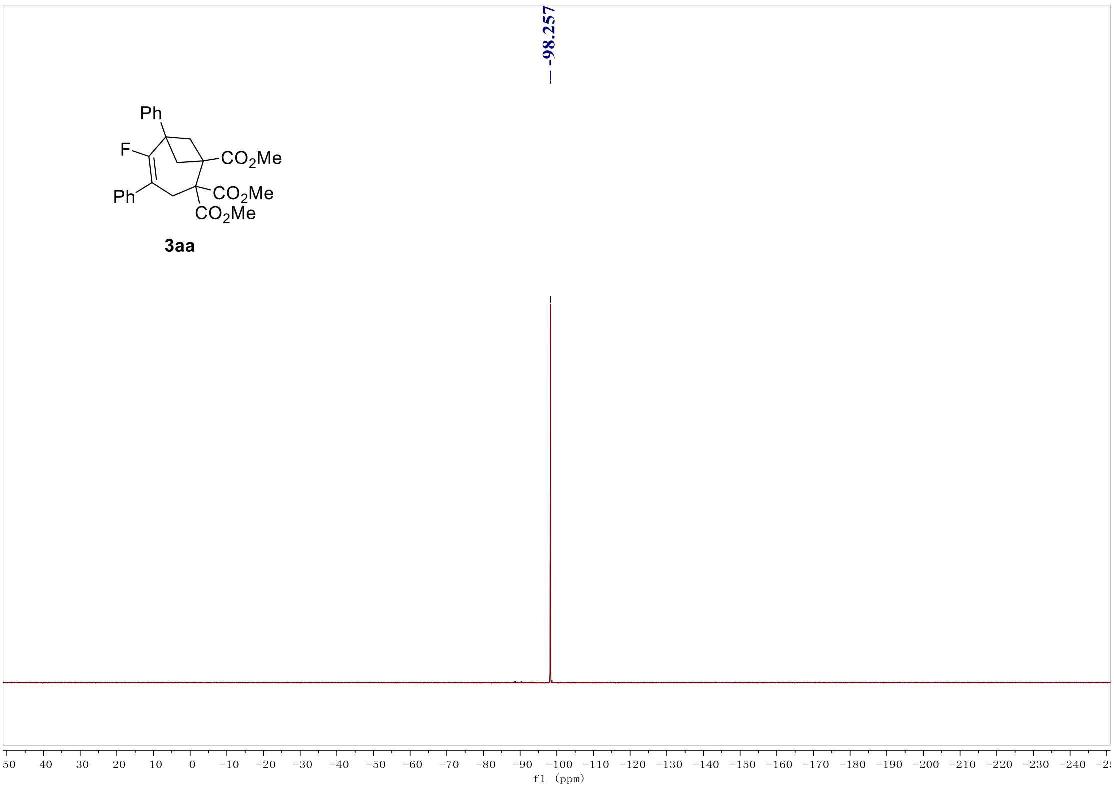

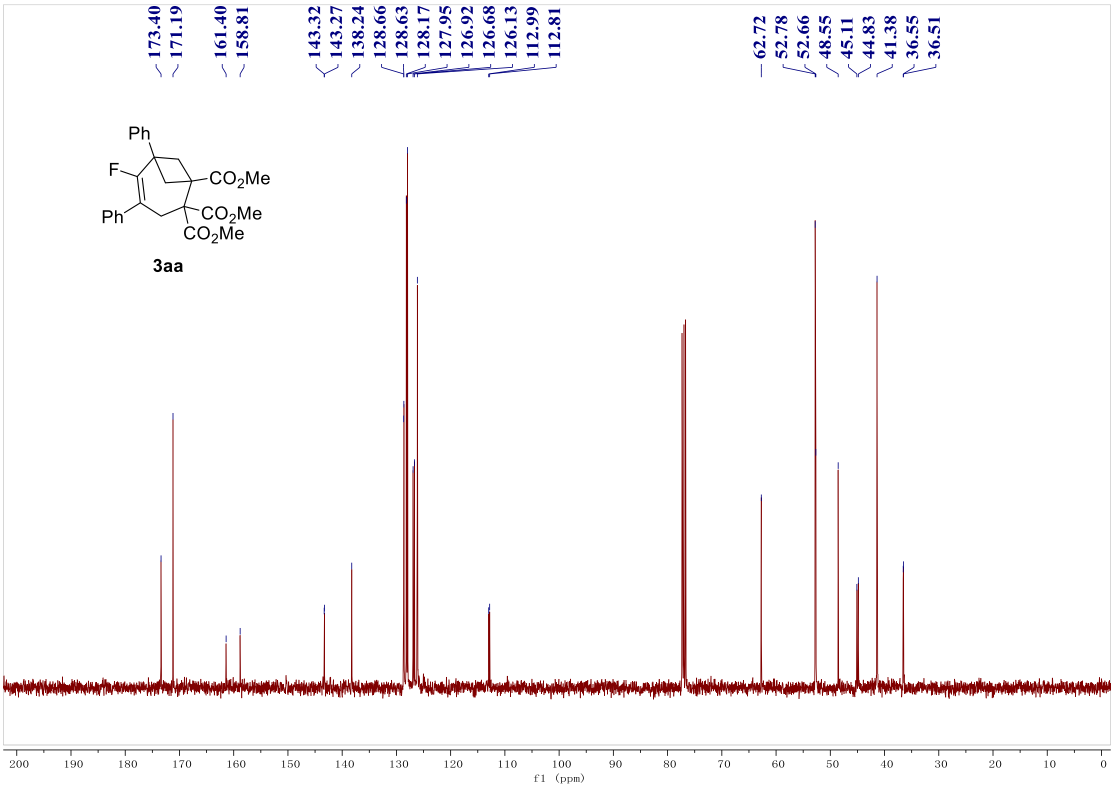

^{1}H NMR (400MHz, $CDCl_3$), ^{19}F NMR (376 MHz, $CDCl_3$) and ^{13}C NMR (100 MHz, $CDCl_3$) spectra of product **3ba**

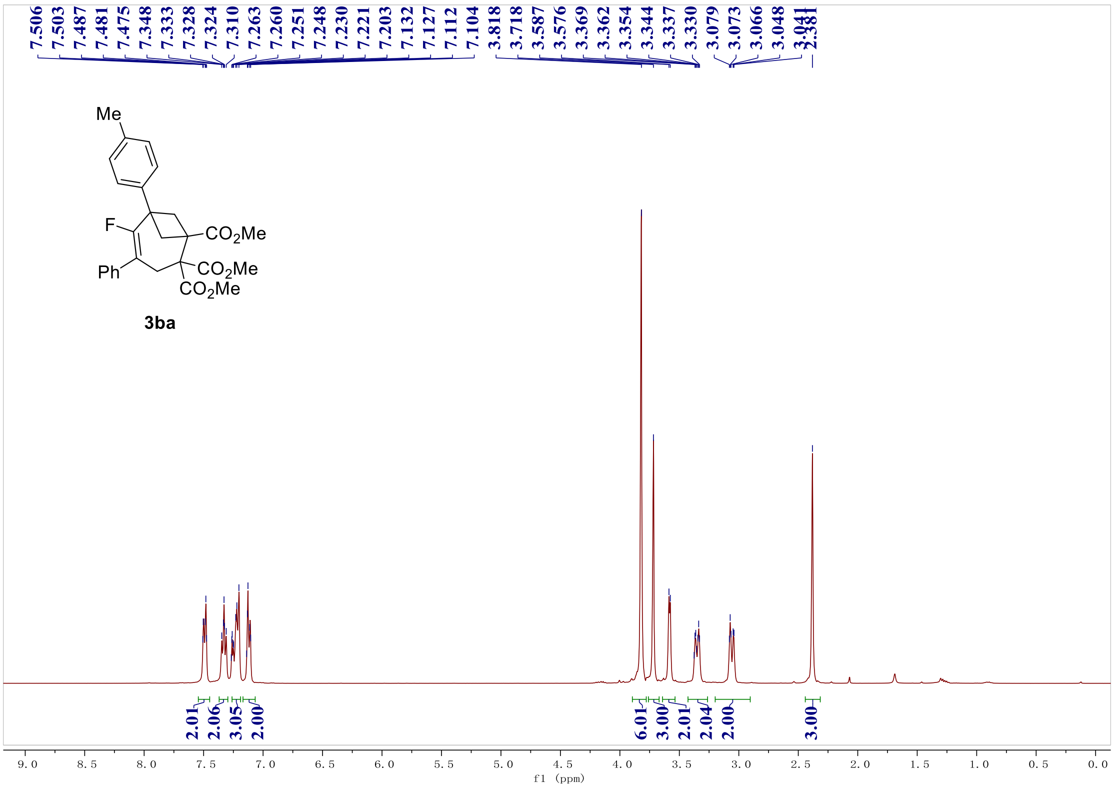

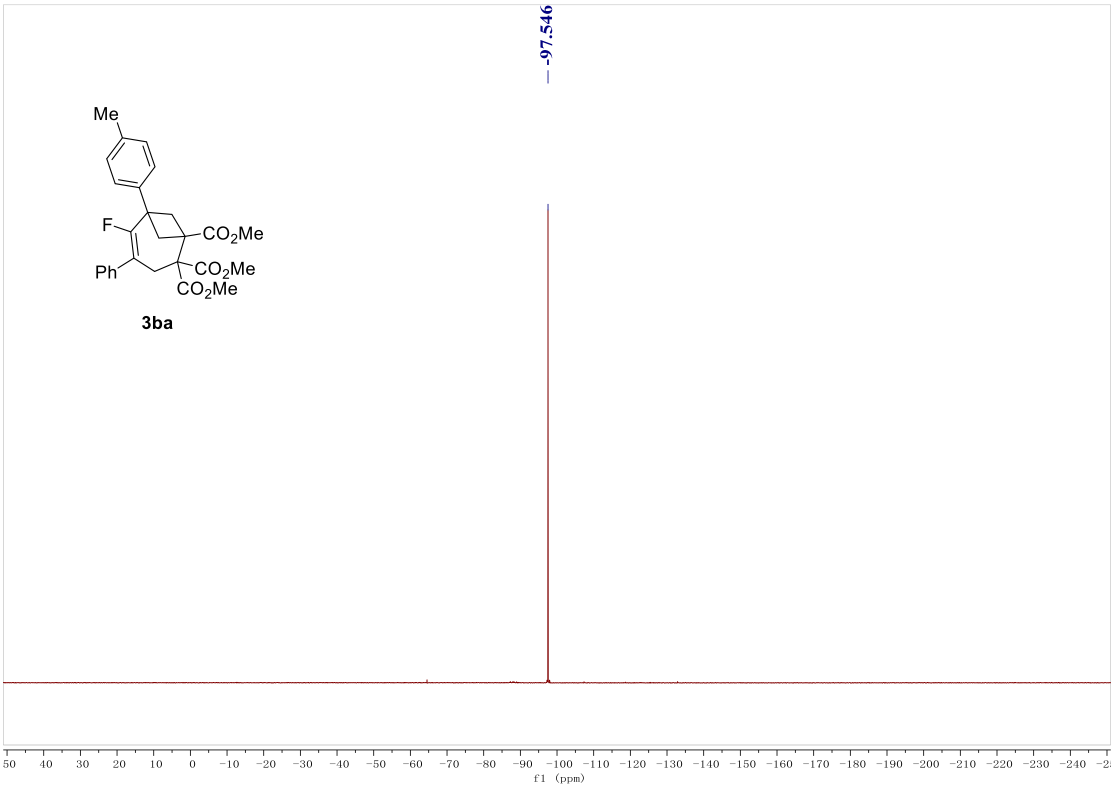

-97.546
Me
F
CO2Me
CO2Me
Ph
CO2Me
3ba
f1 (ppm)

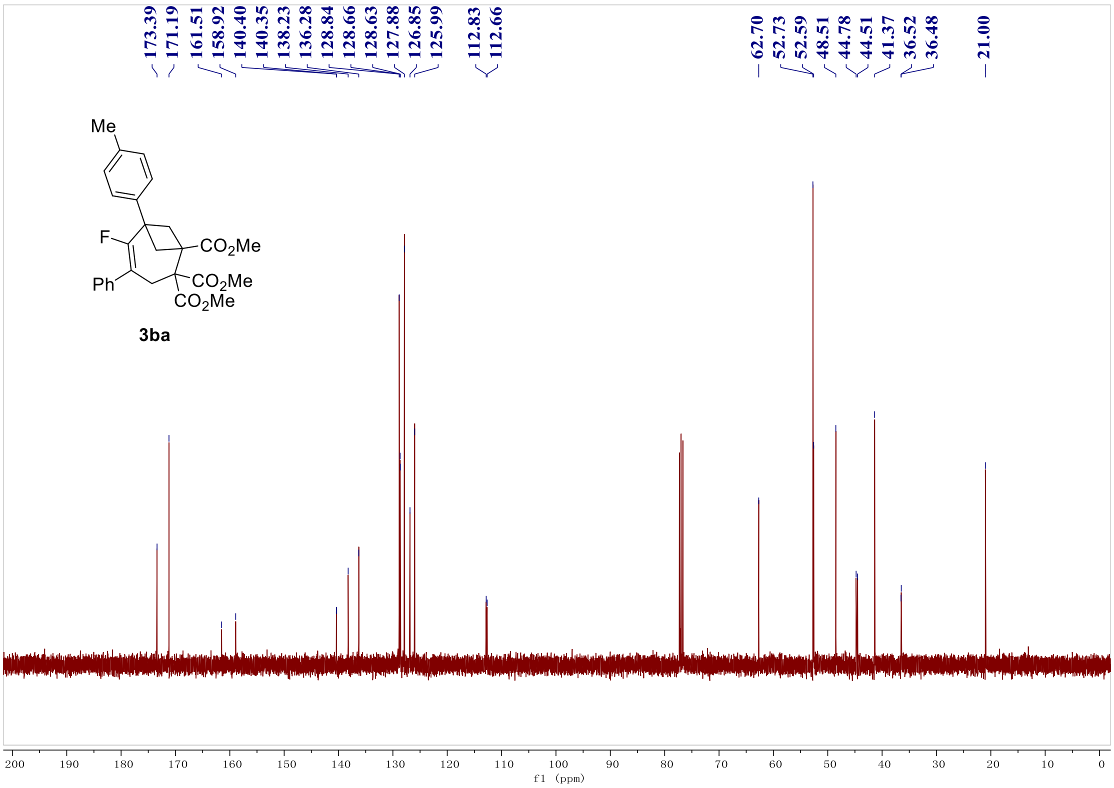

173.39
171.19
161.51
158.92
140.40
140.35
138.23
136.28
128.84
128.66
128.63
127.88
126.85
125.99
112.83
112.66
62.70
52.73
52.59
48.51
44.78
44.51
41.37
36.52
36.48
21.00
Me
F
CO2Me
CO2Me
Ph
CO2Me
3ba
f1 (ppm)

^{1}H NMR (400MHz, $CDCl_3$), ^{19}F NMR (376 MHz, $CDCl_3$) and ^{13}C NMR (100 MHz, $CDCl_3$) spectra of product **3ca**

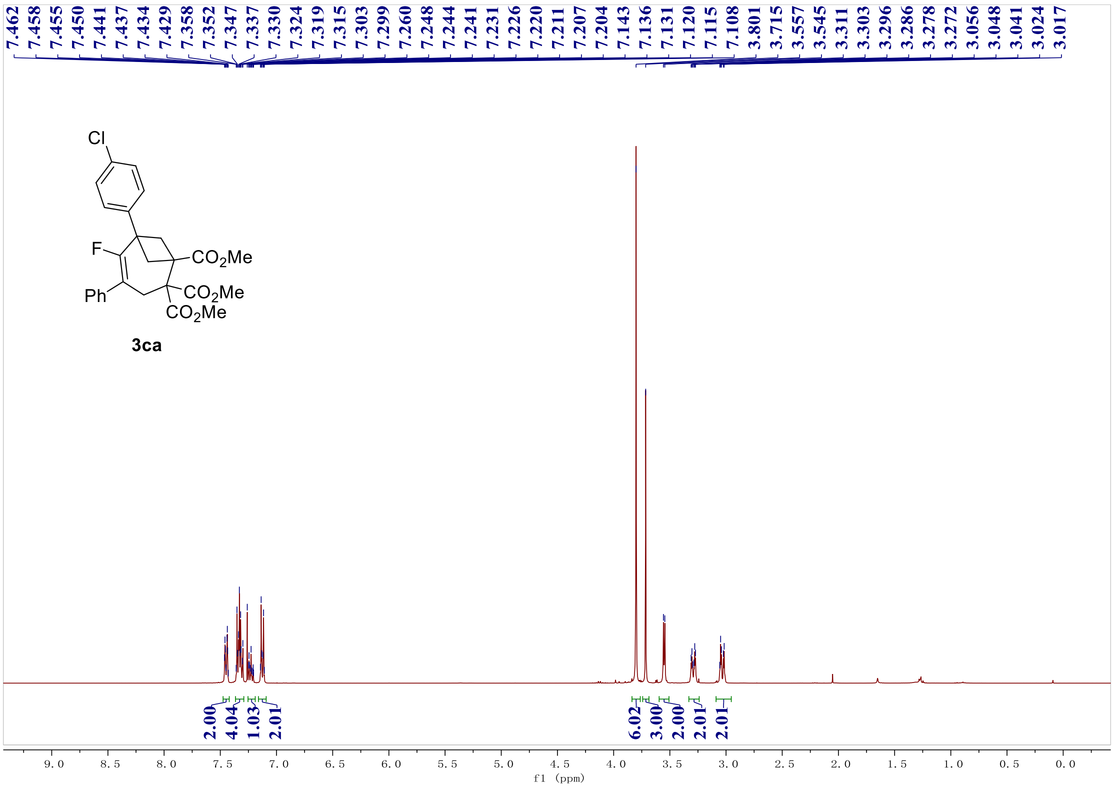

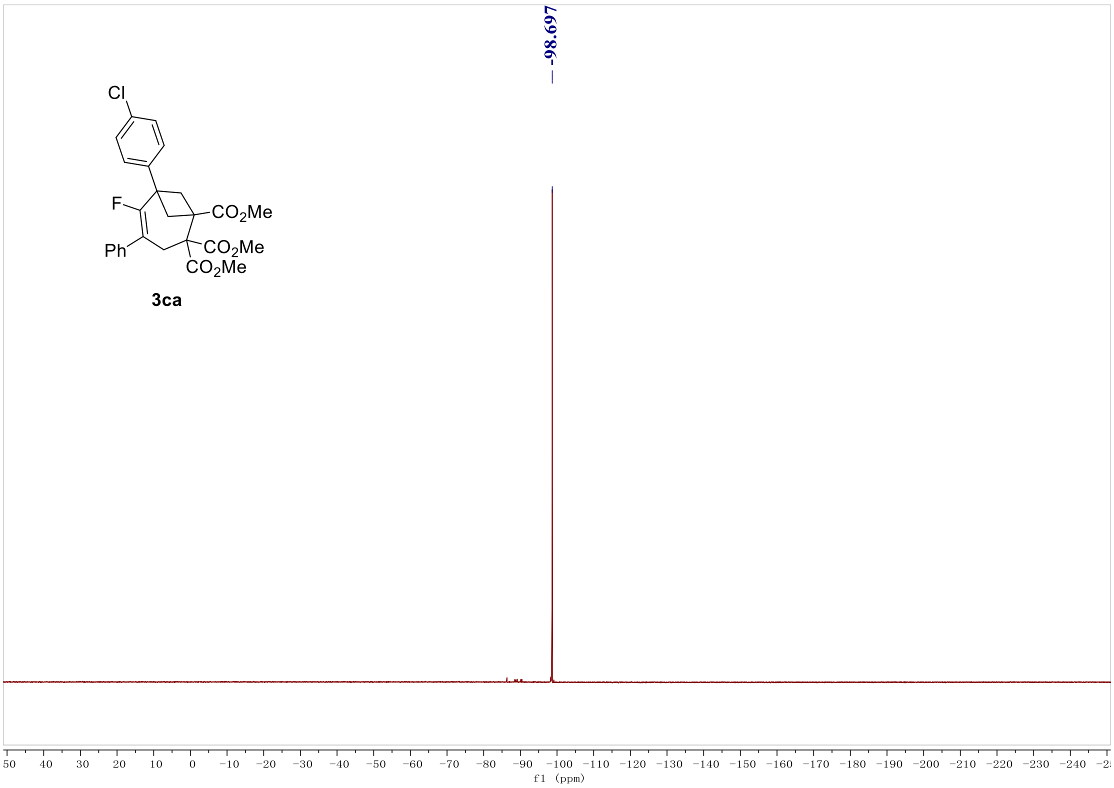

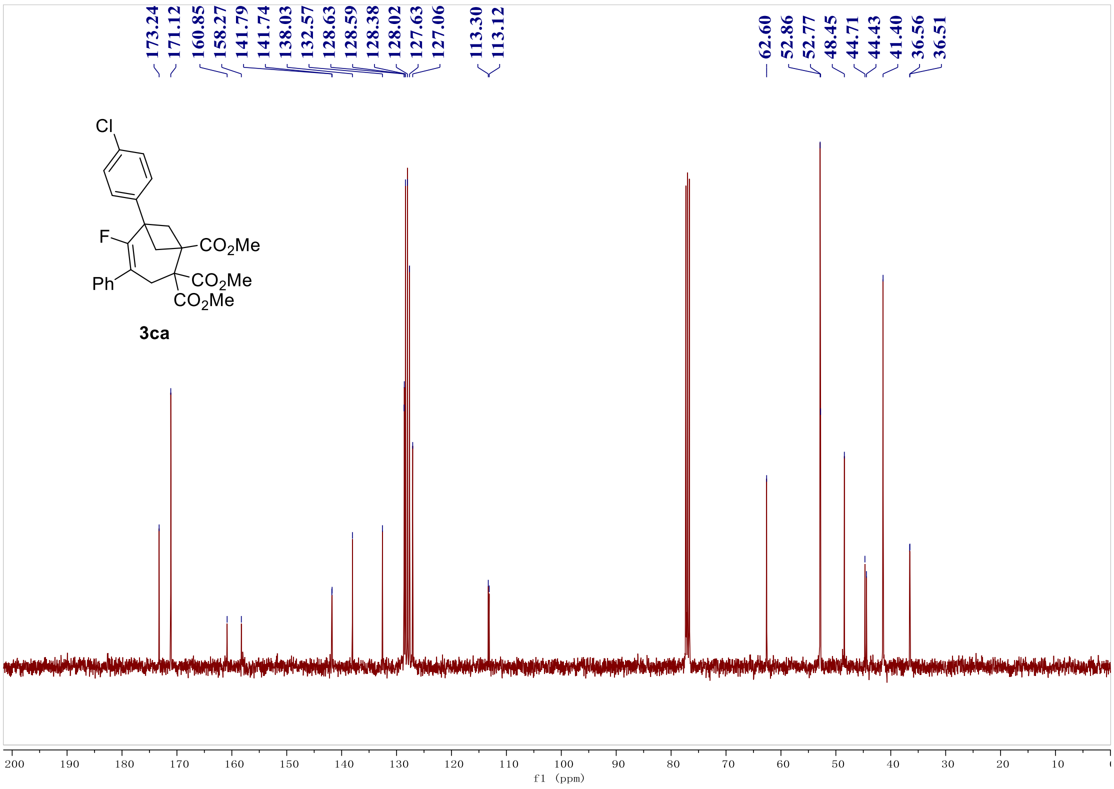

^{1}H NMR (400MHz, $CDCl_3$), ^{19}F NMR (376 MHz, $CDCl_3$) and ^{13}C NMR (100 MHz, $CDCl_3$) spectra of product **3da**

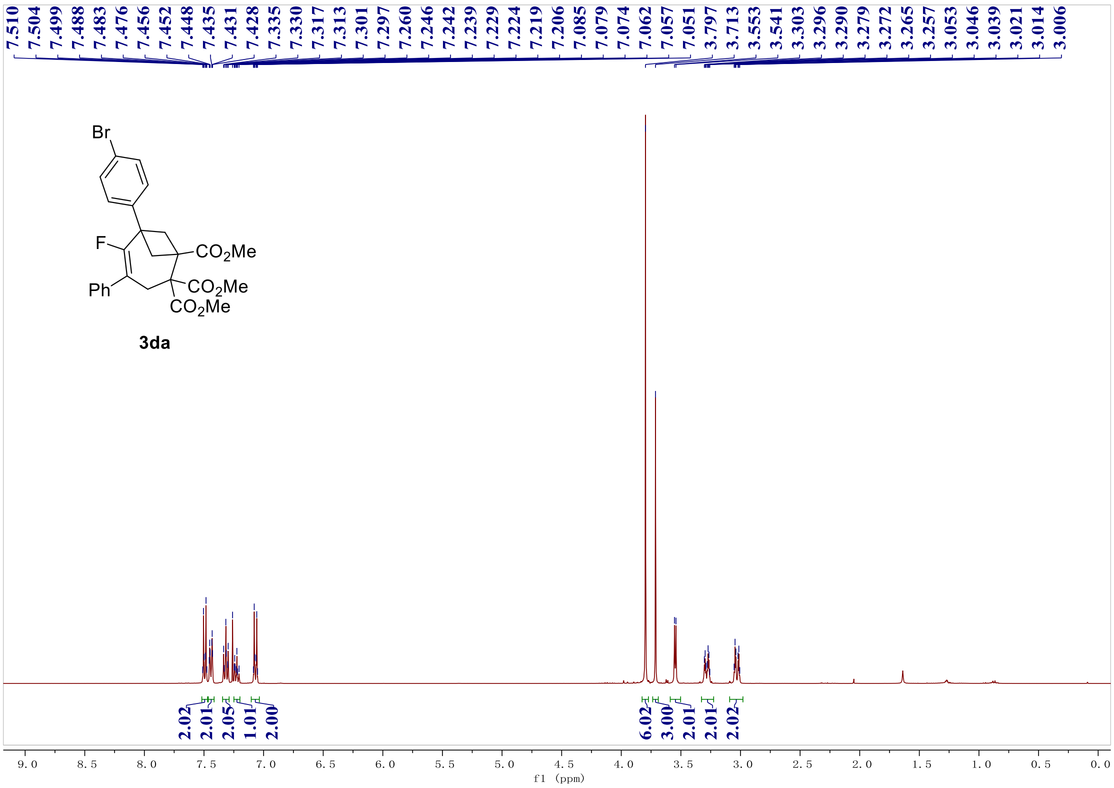

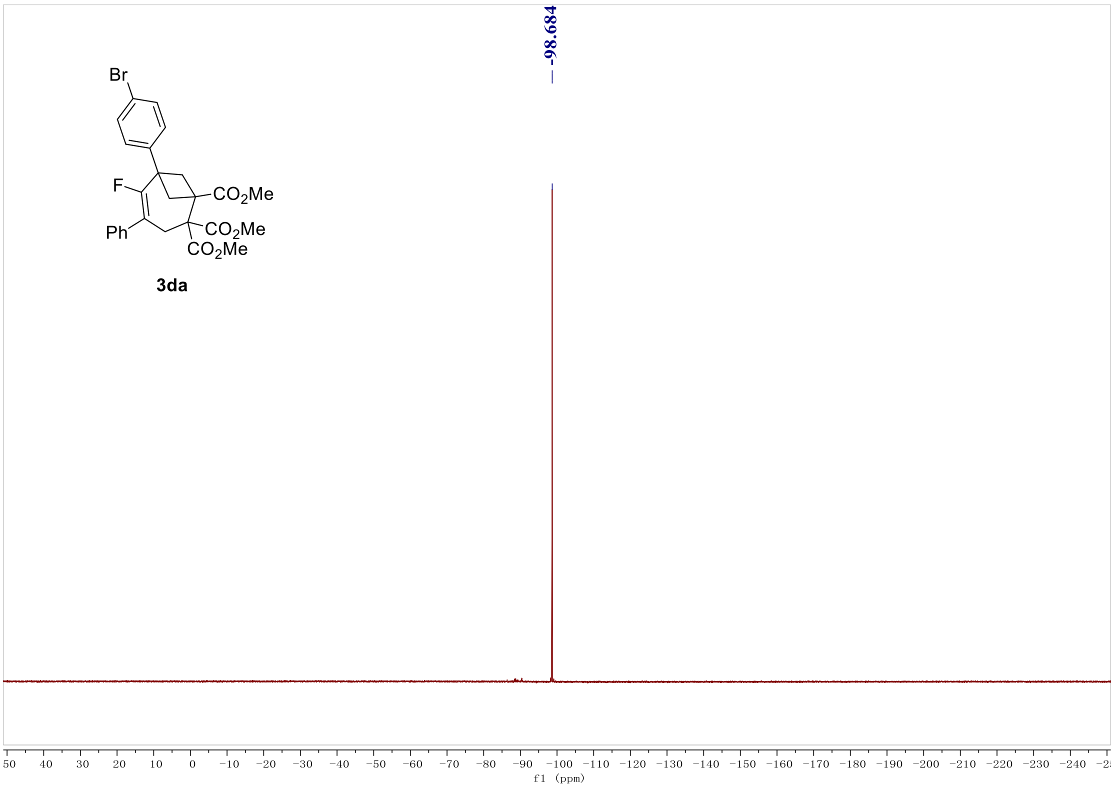
-98.684
Br
F
Ph
CO2Me
CO2Me
CO2Me
3da
f1 (ppm)

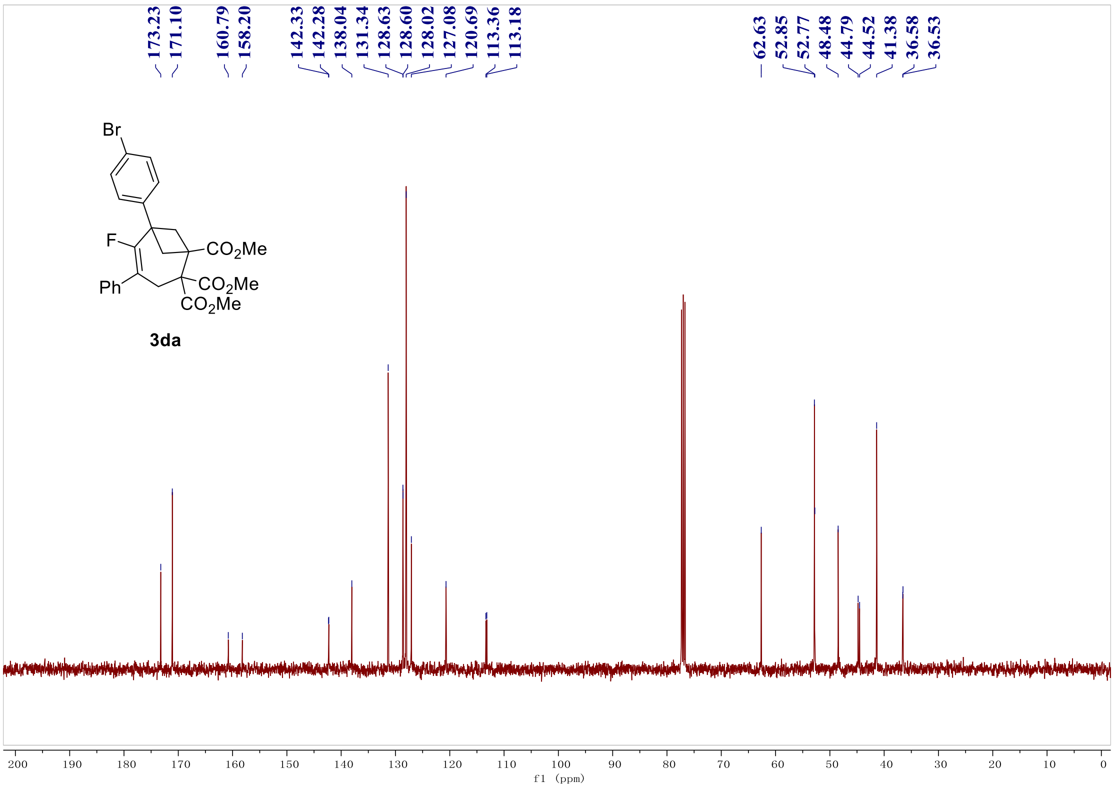
173.23
171.10
160.79
158.20
142.33
142.28
138.04
131.34
128.63
128.60
128.02
127.08
120.69
113.36
113.18
62.63
52.85
52.77
48.48
44.79
44.52
41.38
36.58
36.53
Br
F
Ph
CO2Me
CO2Me
CO2Me
3da
f1 (ppm)

^1H NMR (400MHz, $CDCl_3$), ^{19}F NMR (376 MHz, $CDCl_3$) and ^{13}C NMR (100 MHz, $CDCl_3$) spectra of product **3ea**

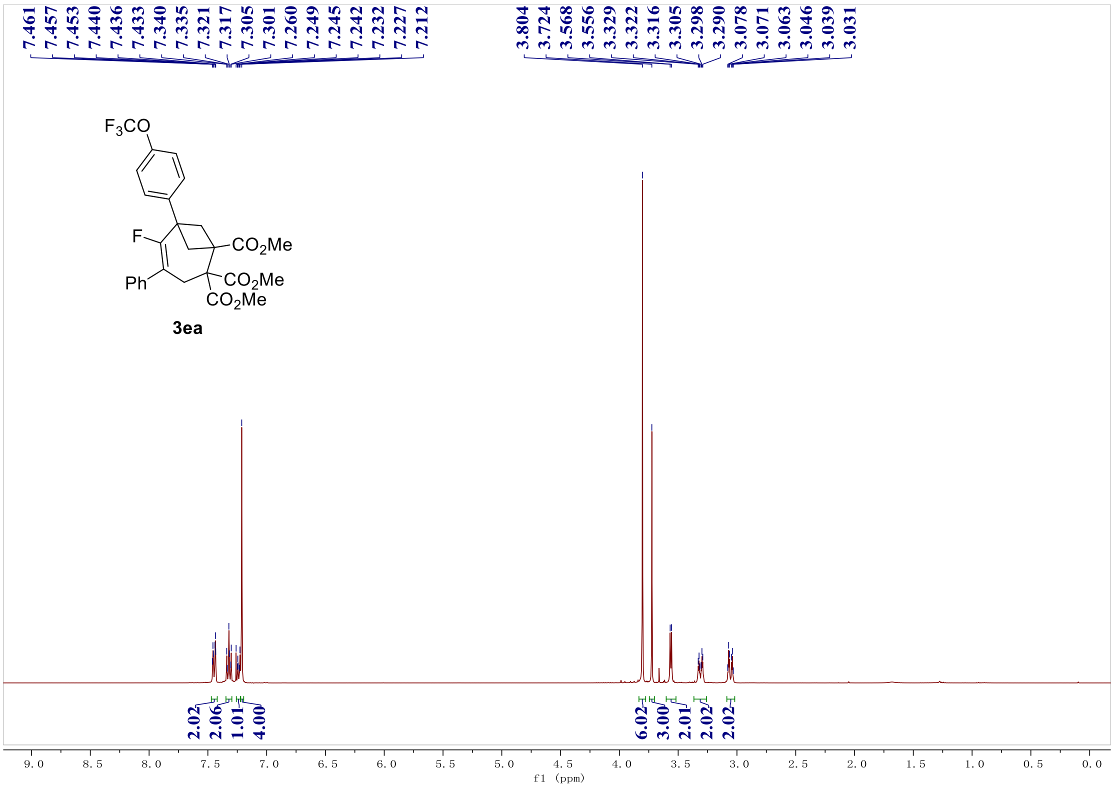

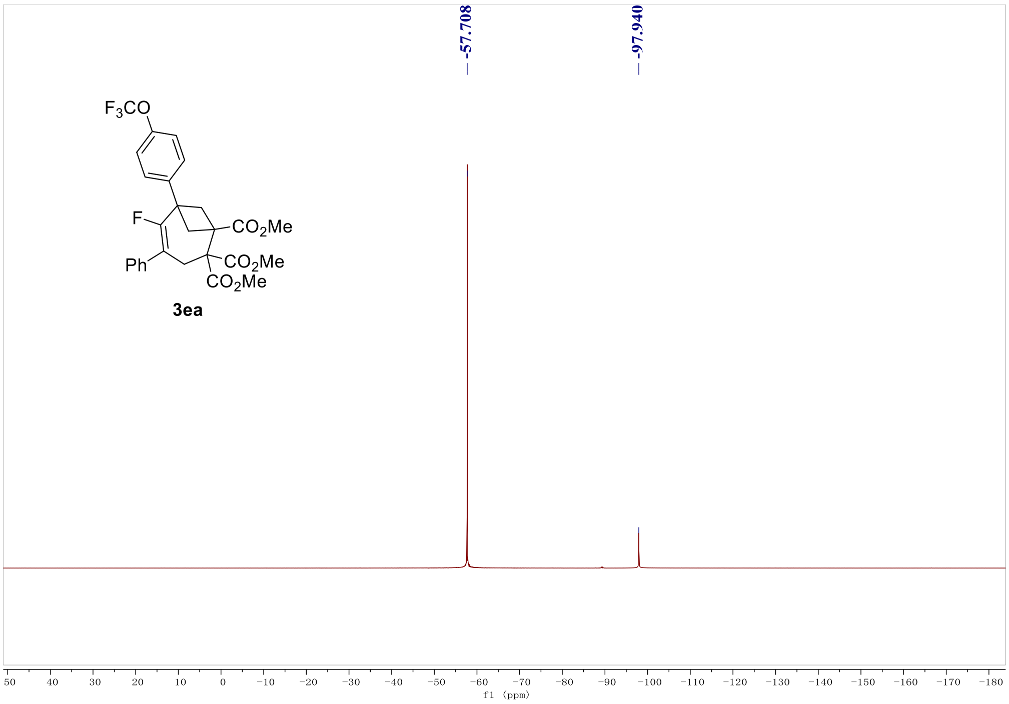

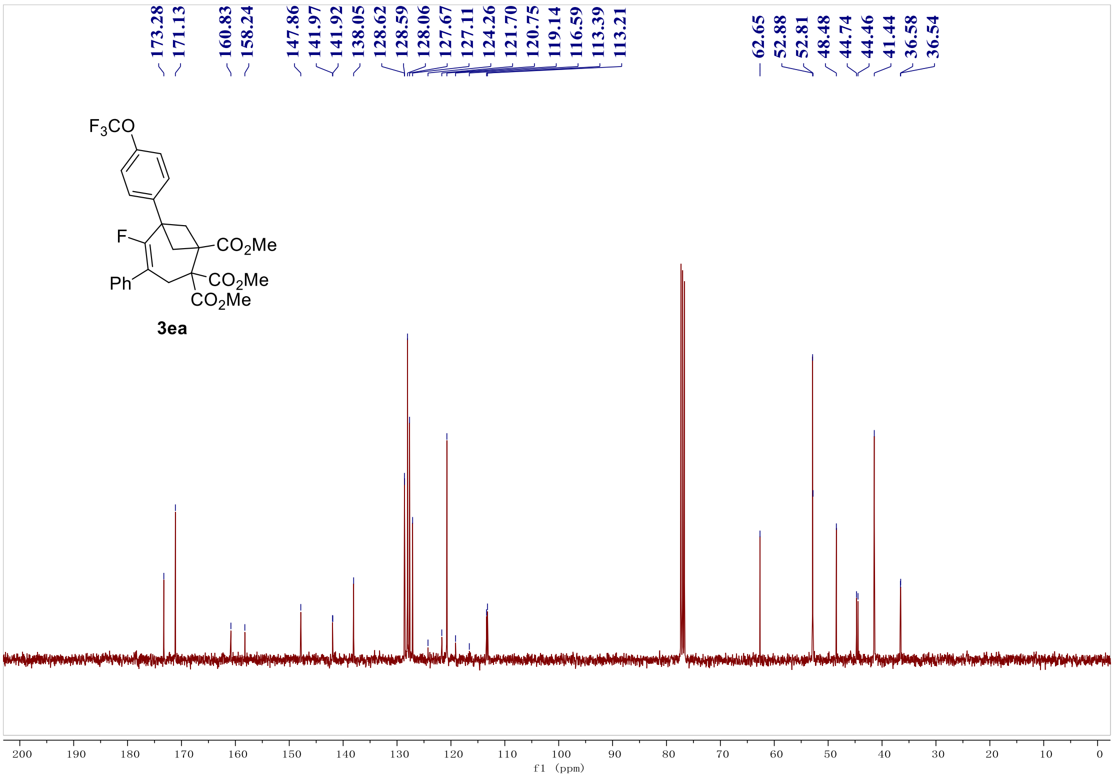

^1H NMR (400MHz, $CDCl_3$), ^{19}F NMR (376 MHz, $CDCl_3$) and ^{13}C NMR (100 MHz, $CDCl_3$) spectra of product **3fa**

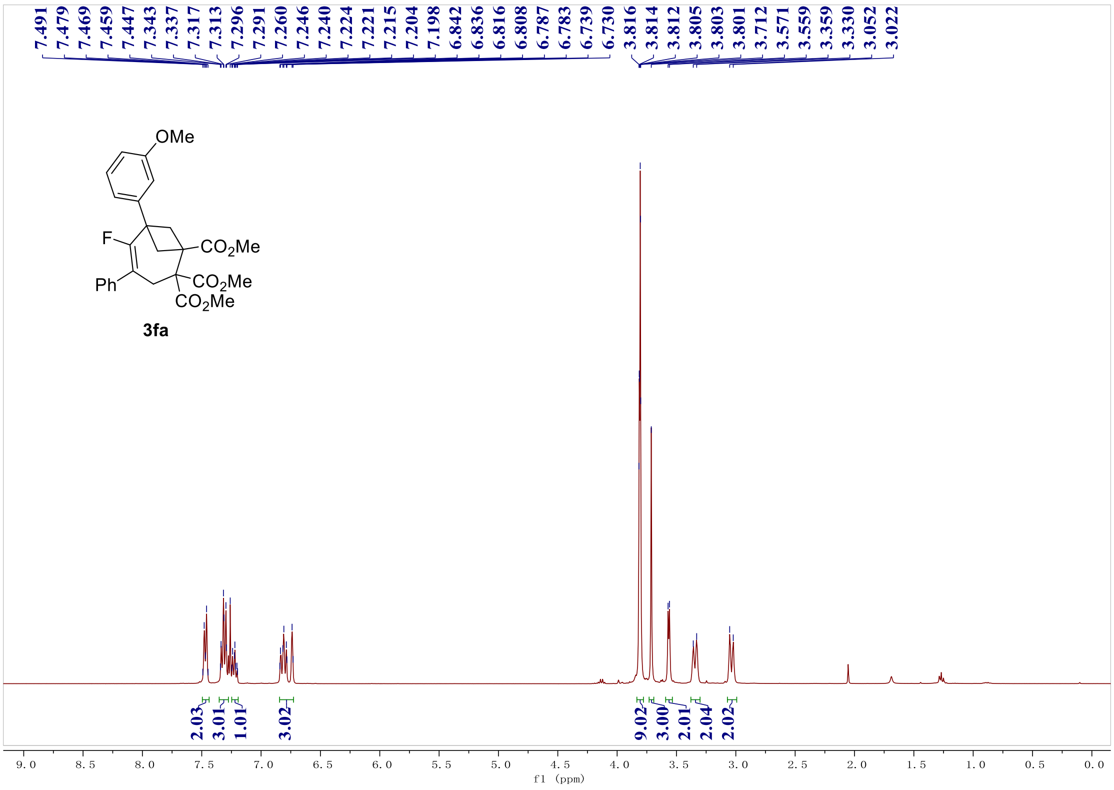

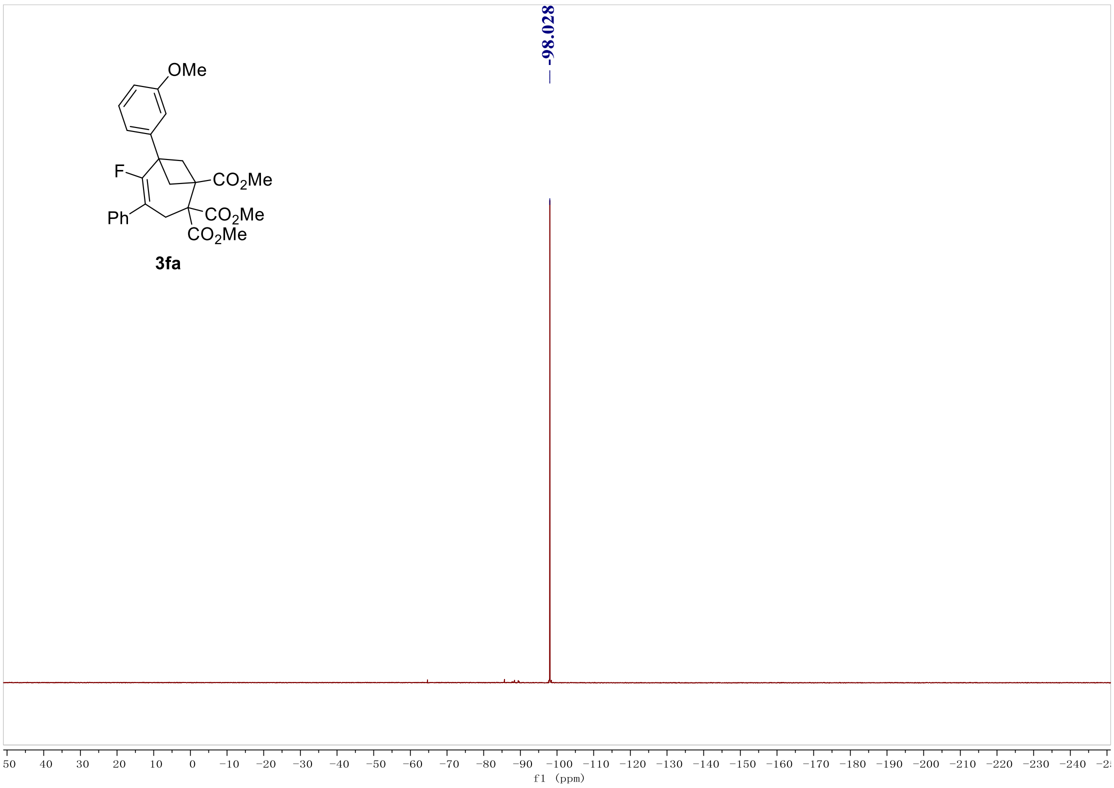
-98.028
OMe
F
CO2Me
Ph
CO2Me
CO2Me
3fa
f1 (ppm)

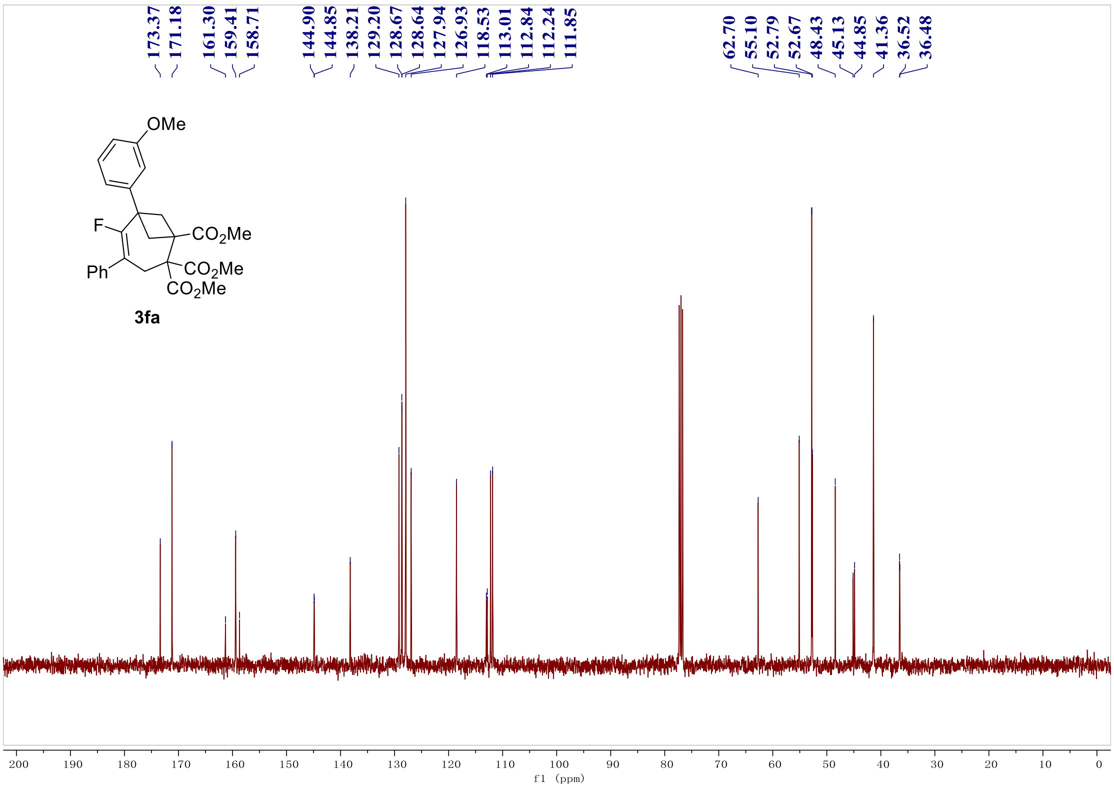
173.37
171.18
161.30
159.41
158.71
144.90
144.85
138.21
129.20
128.67
128.64
127.94
126.93
118.53
113.01
112.84
112.24
111.85
62.70
55.10
52.79
52.67
48.43
45.13
44.85
41.36
36.52
36.48
OMe
F
CO2Me
Ph
CO2Me
CO2Me
3fa
f1 (ppm)

^{1}H NMR (400MHz, $CDCl_3$), ^{19}F NMR (376 MHz, $CDCl_3$) and ^{13}C NMR (100 MHz, $CDCl_3$) spectra of product **3ga**

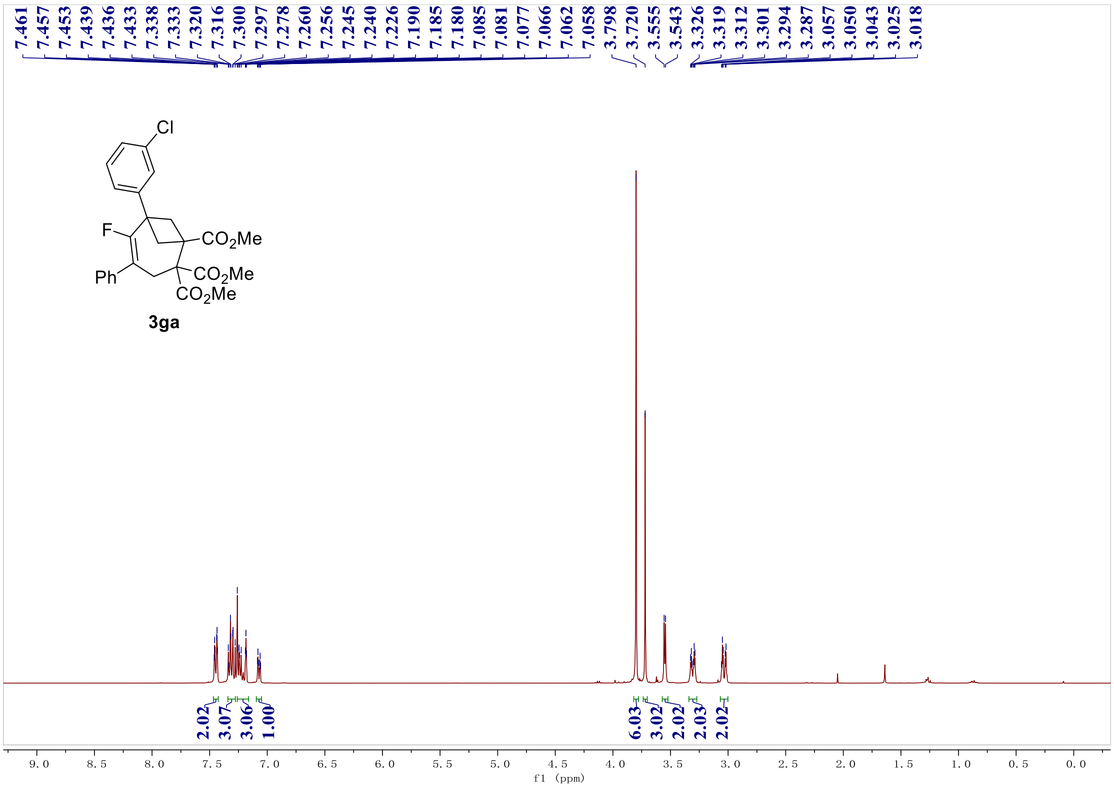

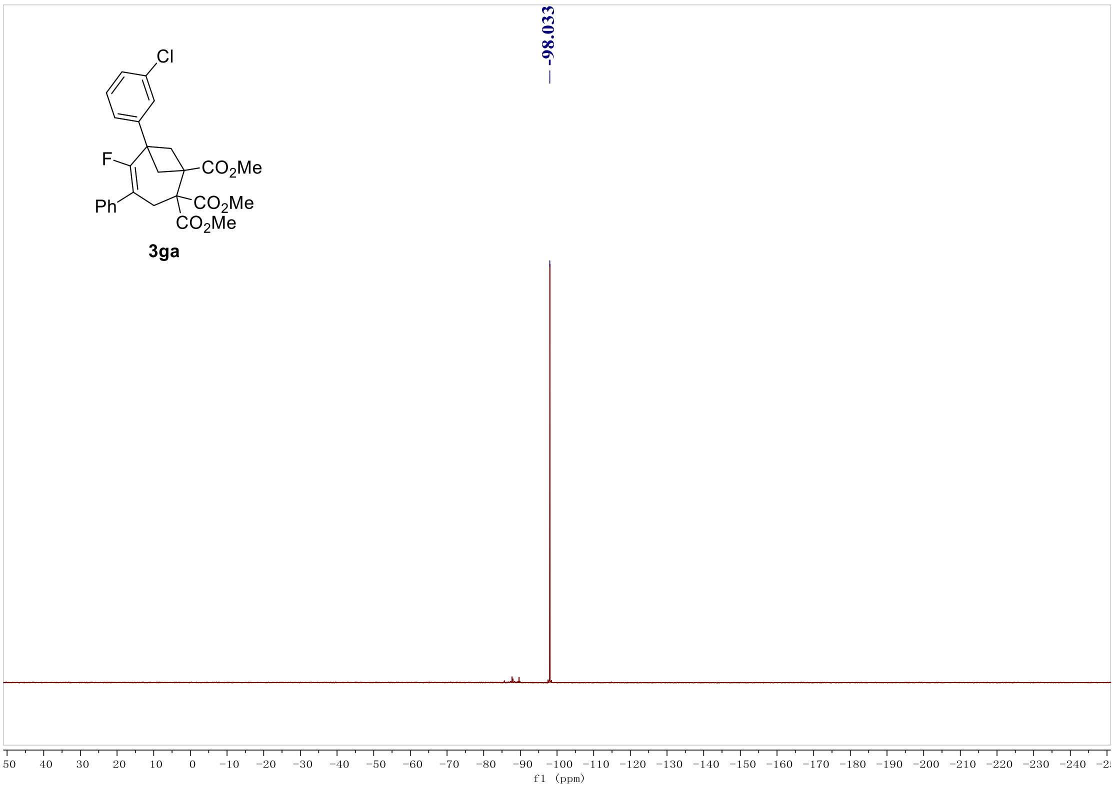

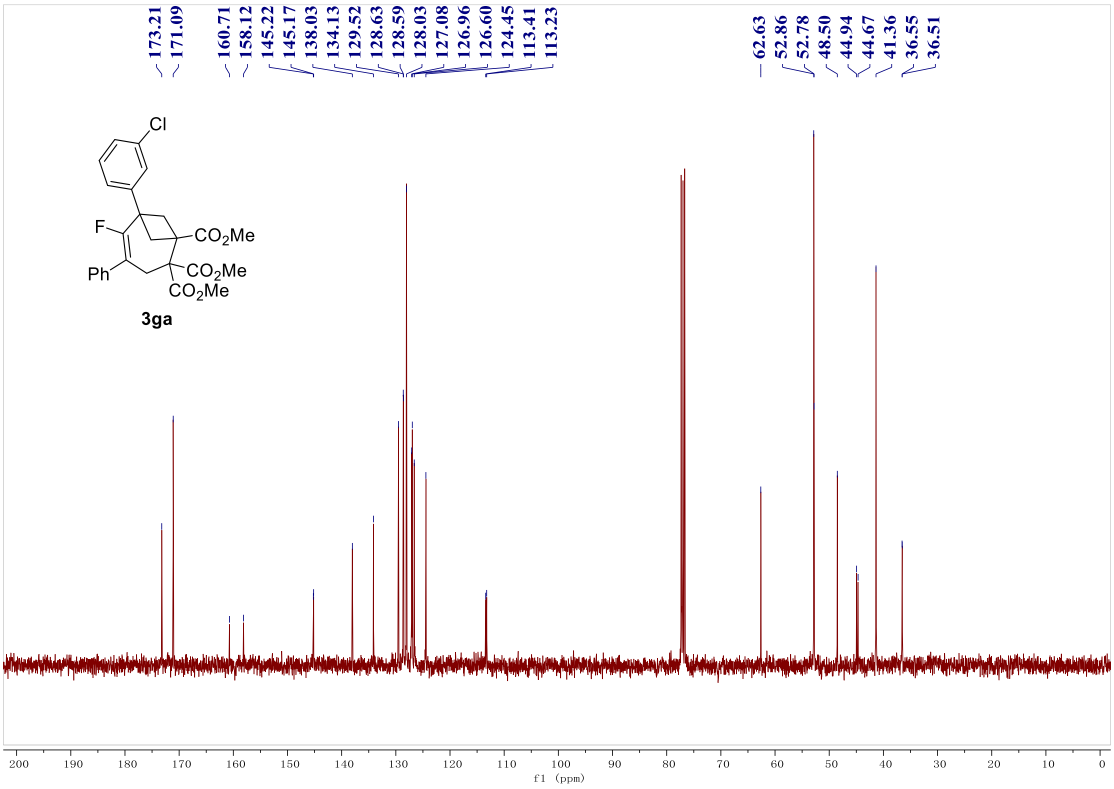

^{1}H NMR (400MHz, $CDCl_3$), ^{19}F NMR (376 MHz, $CDCl_3$) and ^{13}C NMR (100 MHz, $CDCl_3$) spectra of product **3ha**

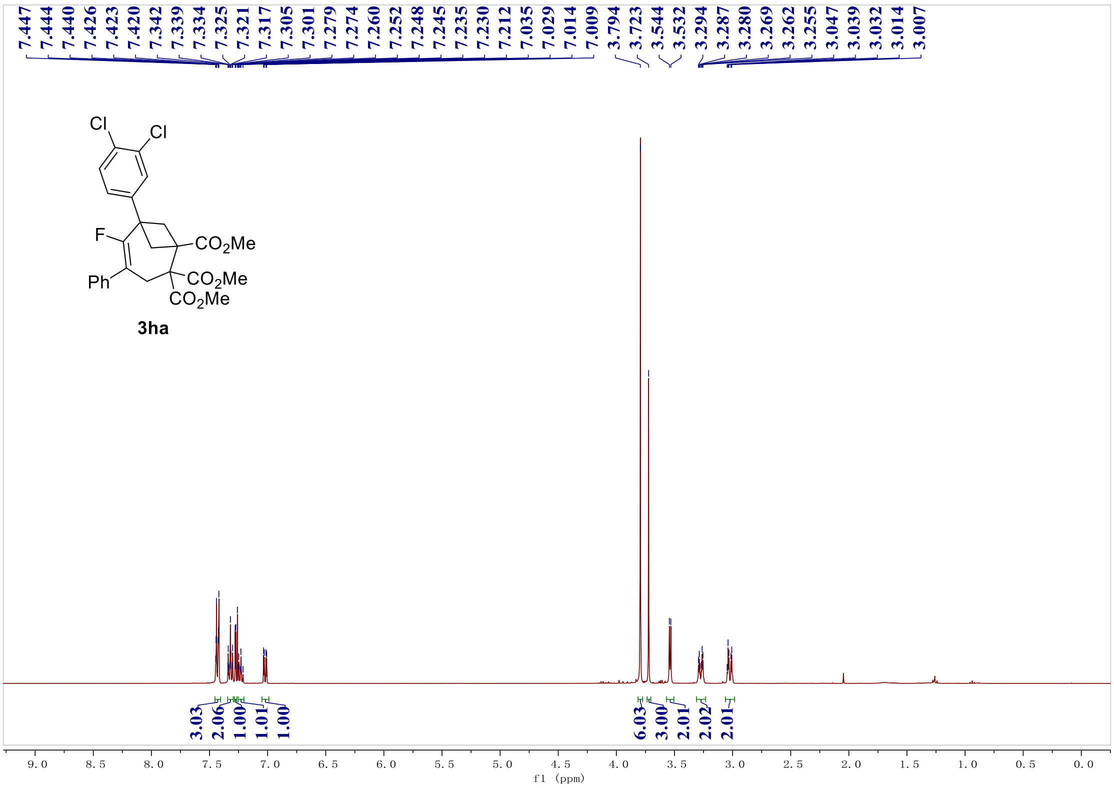

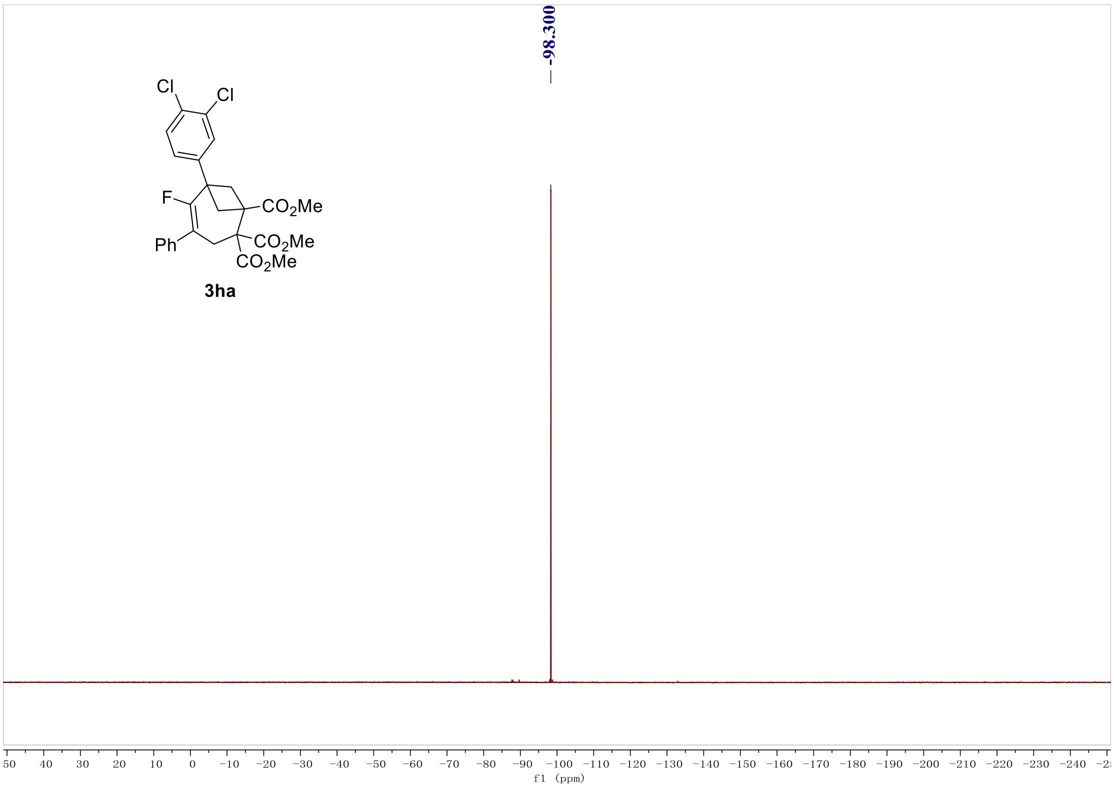
-98.300
Cl
Cl
F
CO2Me
Ph
CO2Me
CO2Me
3ha
50 40 30 20 10 0 −10 −20 −30 −40 −50 −60 −70 −80 −90 −100 −110 −120 −130 −140 −150 −160 −170 −180 −190 −200 −210 −220 −230 −240
f1 (ppm)

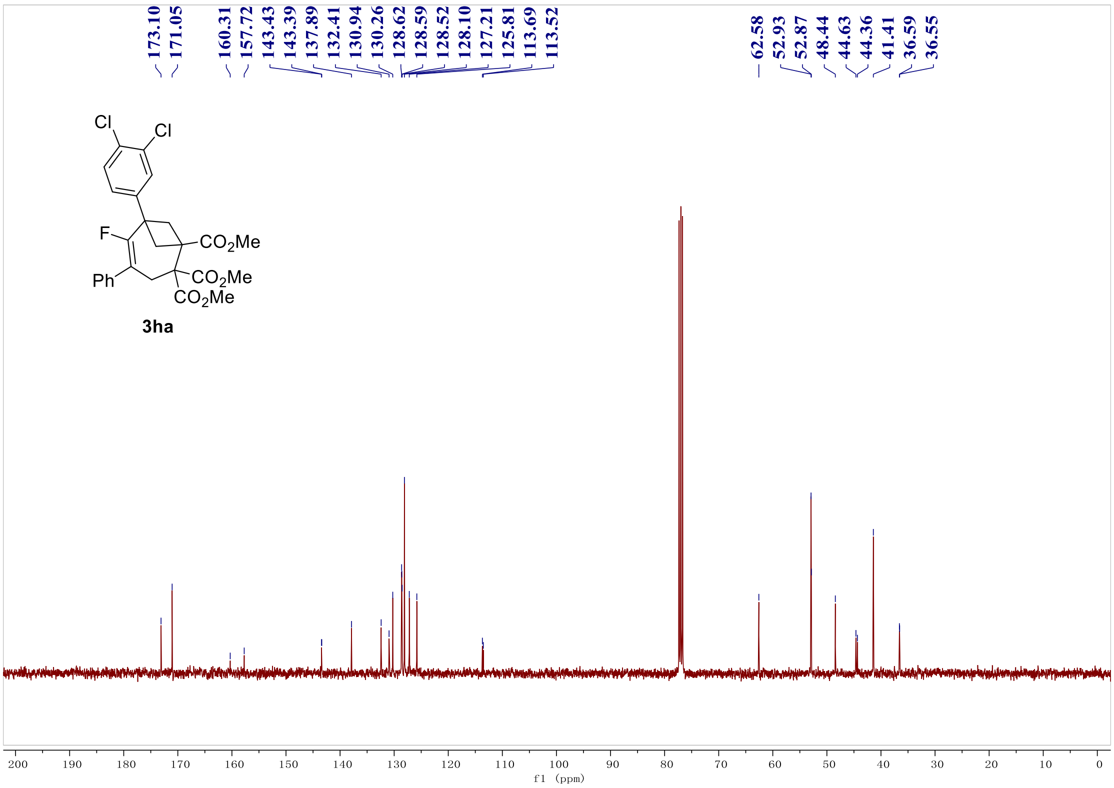
173.10
171.05
160.31
157.72
143.43
143.39
137.89
132.41
130.94
130.26
128.62
128.59
128.52
128.10
127.21
125.81
113.69
113.52
62.58
52.93
52.87
48.44
44.63
44.36
41.41
36.59
36.55
Cl
Cl
F
CO2Me
Ph
CO2Me
CO2Me
3ha
200 190 180 170 160 150 140 130 120 110 100 90 80 70 60 50 40 30 20 10 0
f1 (ppm)

^{1}H NMR (400MHz, $CDCl_3$), ^{19}F NMR (376 MHz, $CDCl_3$) and ^{13}C NMR (100 MHz, $CDCl_3$) spectra of product **3ia**

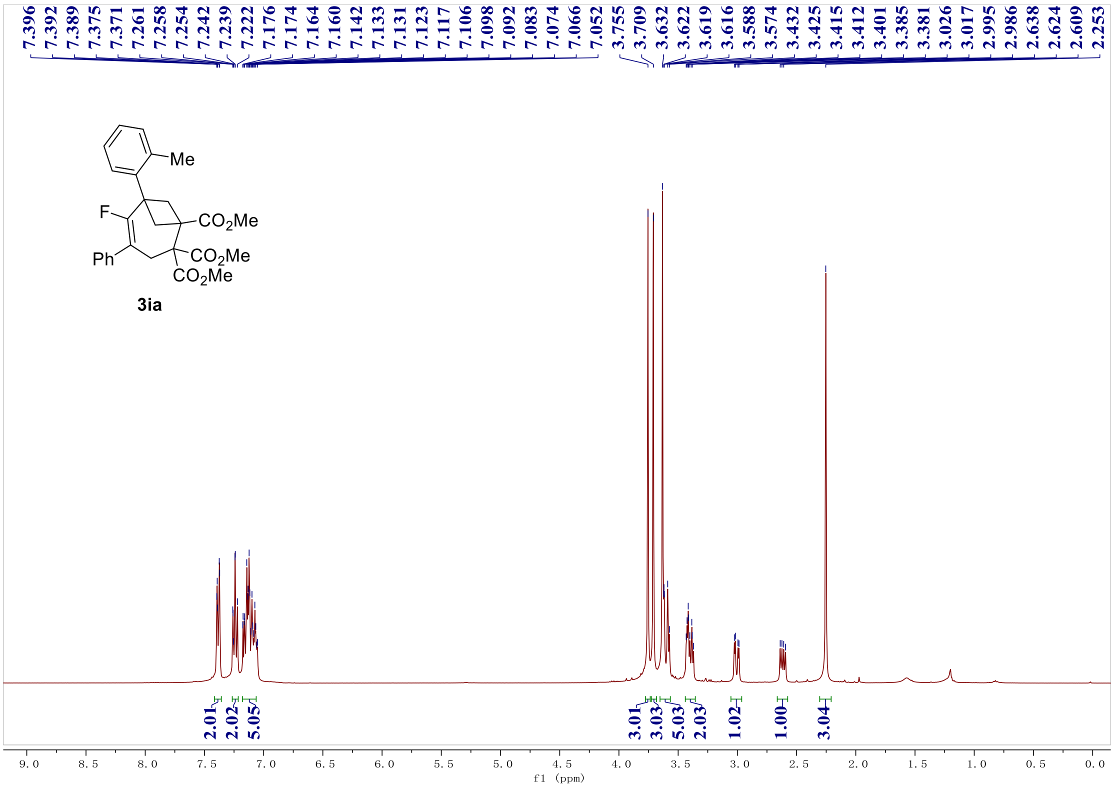

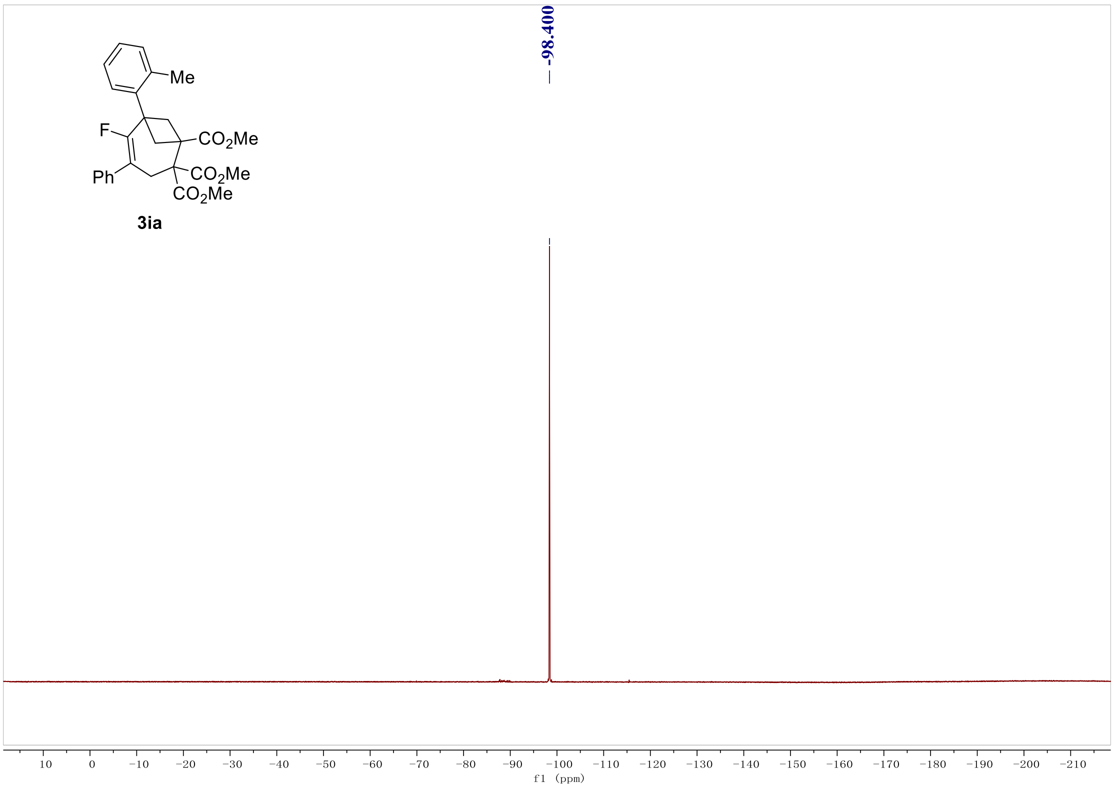

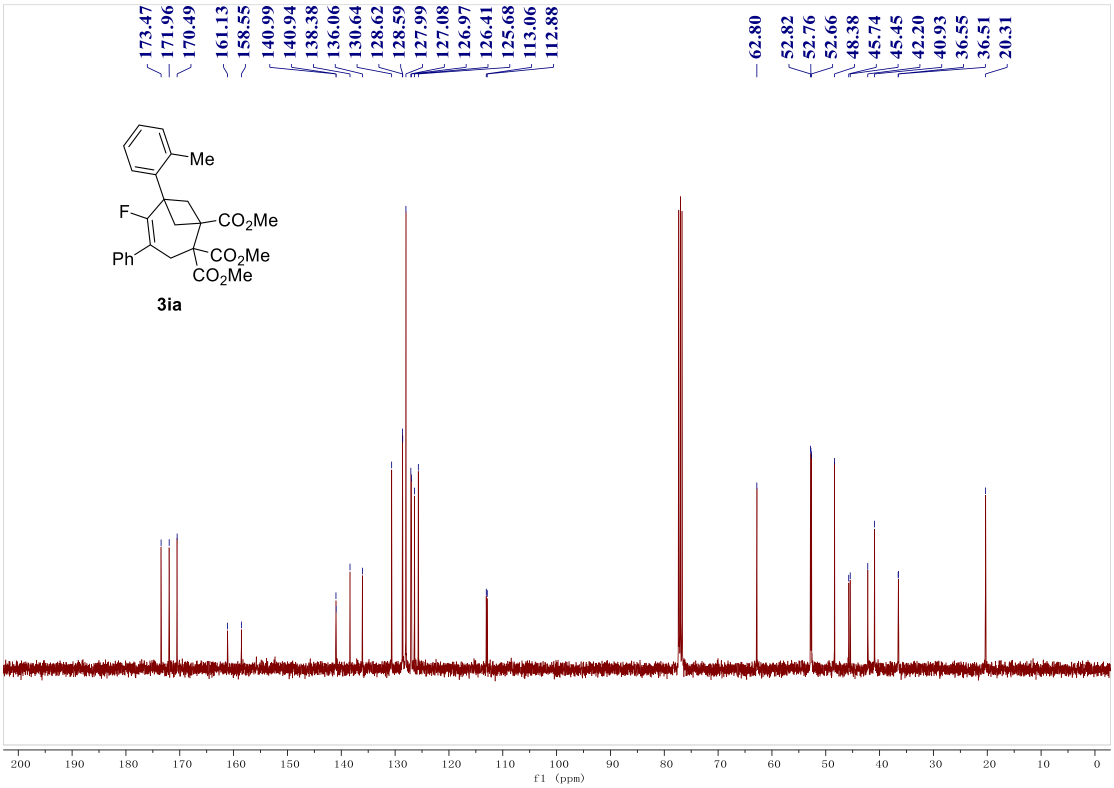

^{1}H NMR (400MHz, $CDCl_3$), ^{19}F NMR (376 MHz, $CDCl_3$) and ^{13}C NMR (100 MHz, $CDCl_3$) spectra of product **3ja**

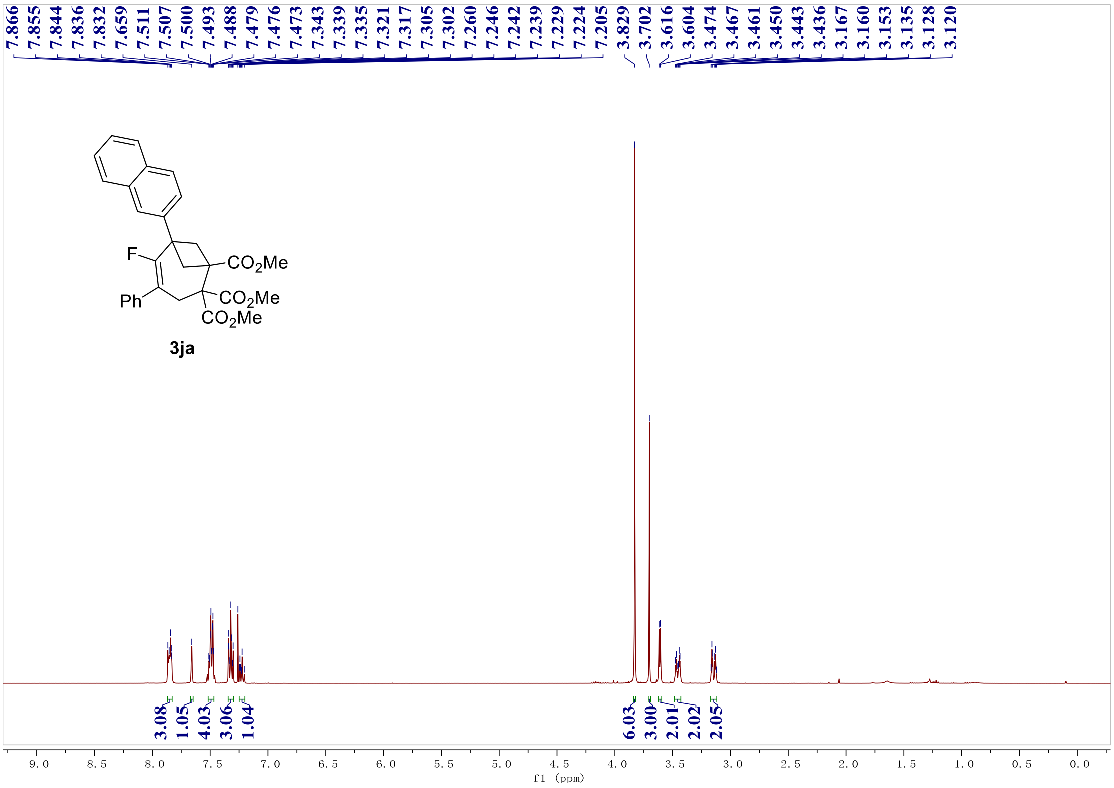

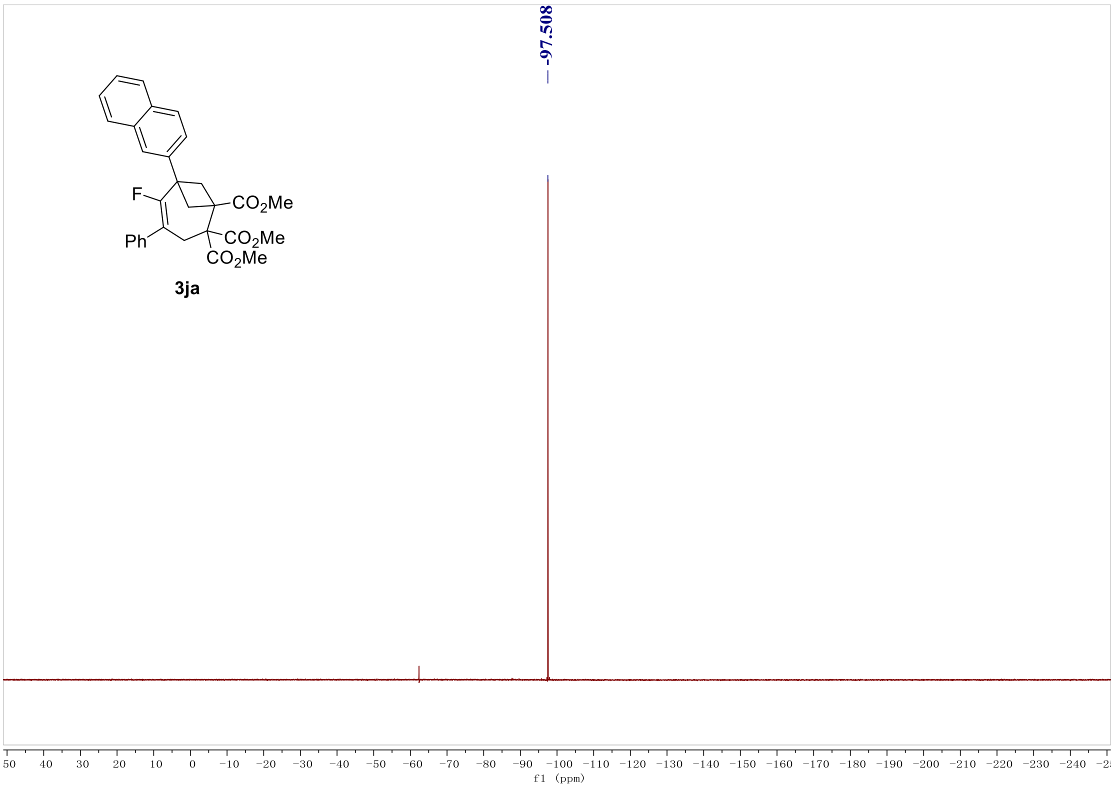
-97.508
F
CO2Me
Ph
CO2Me
CO2Me
3ja
50 40 30 20 10 0 −10 −20 −30 −40 −50 −60 −70 −80 −90 −100 −110 −120 −130 −140 −150 −160 −170 −180 −190 −200 −210 −220 −230 −240
f1 (ppm)

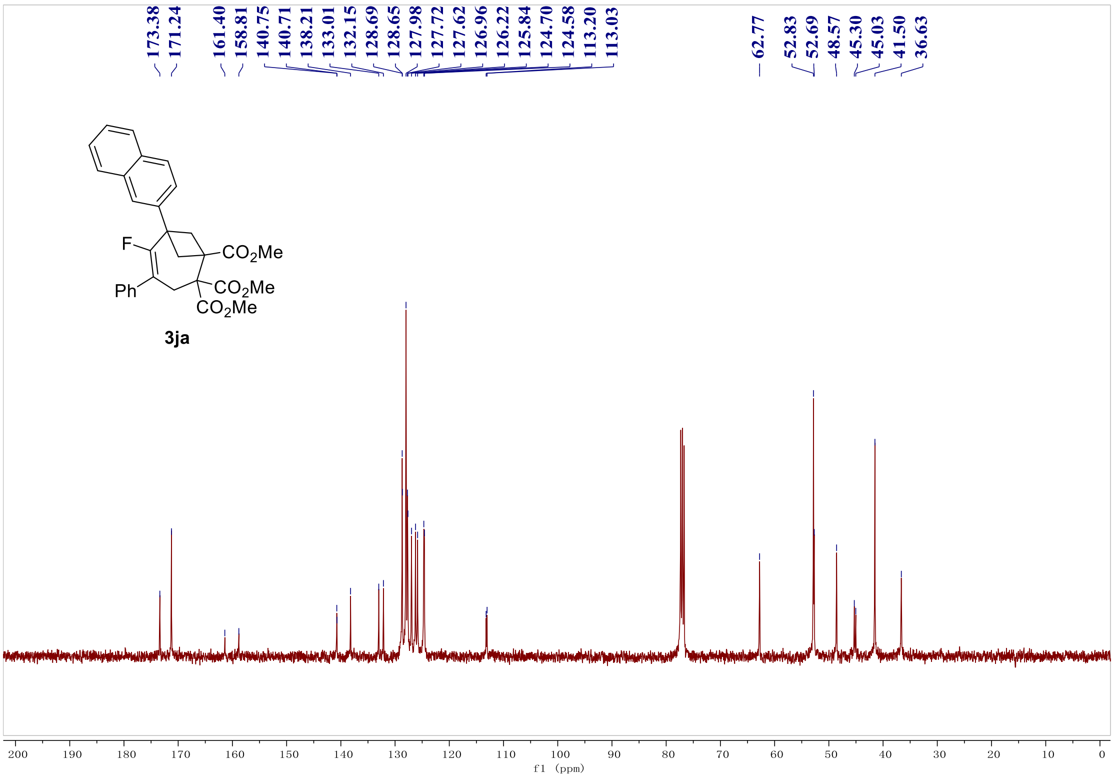
173.38
171.24
161.40
158.81
140.75
140.71
138.21
133.01
132.15
128.69
128.65
127.98
127.72
127.62
126.96
126.22
125.84
124.70
124.58
113.20
113.03
62.77
52.83
52.69
48.57
45.30
45.03
41.50
36.63
F
CO2Me
Ph
CO2Me
CO2Me
3ja
200 190 180 170 160 150 140 130 120 110 100 90 80 70 60 50 40 30 20 10 0
f1 (ppm)

^{1}H NMR (400MHz, $CDCl_3$), ^{19}F NMR (376 MHz, $CDCl_3$) and ^{13}C NMR (100 MHz, $CDCl_3$) spectra of product **3ka**

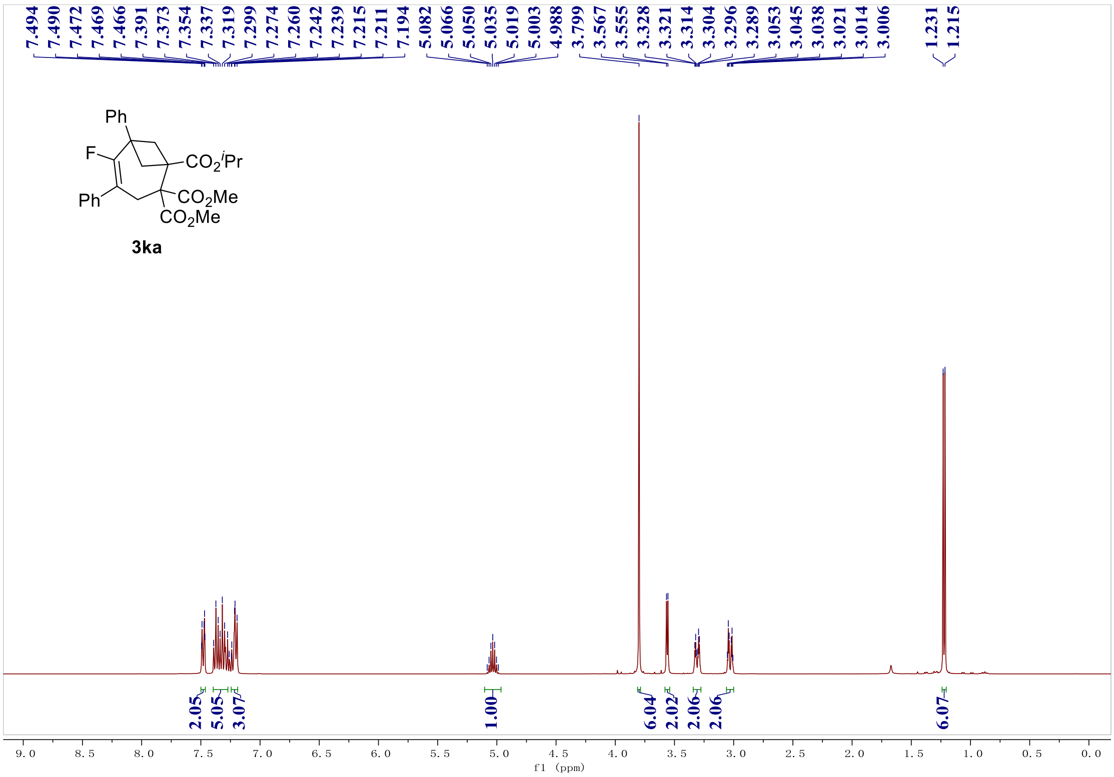

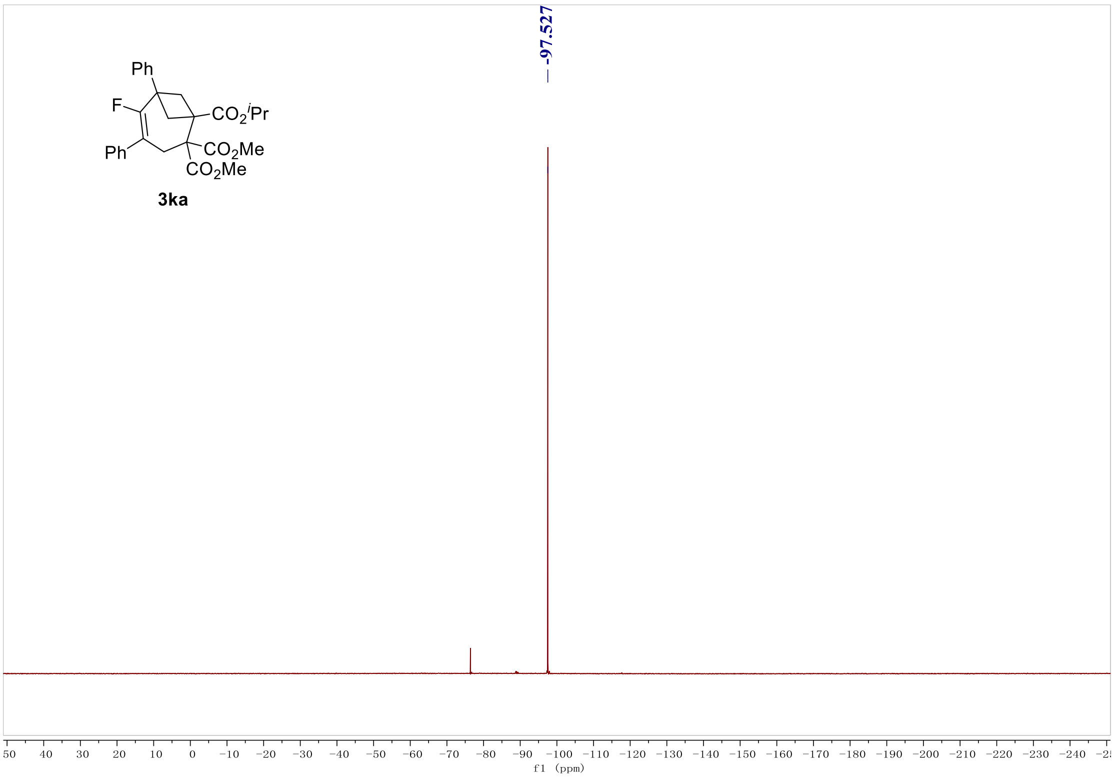

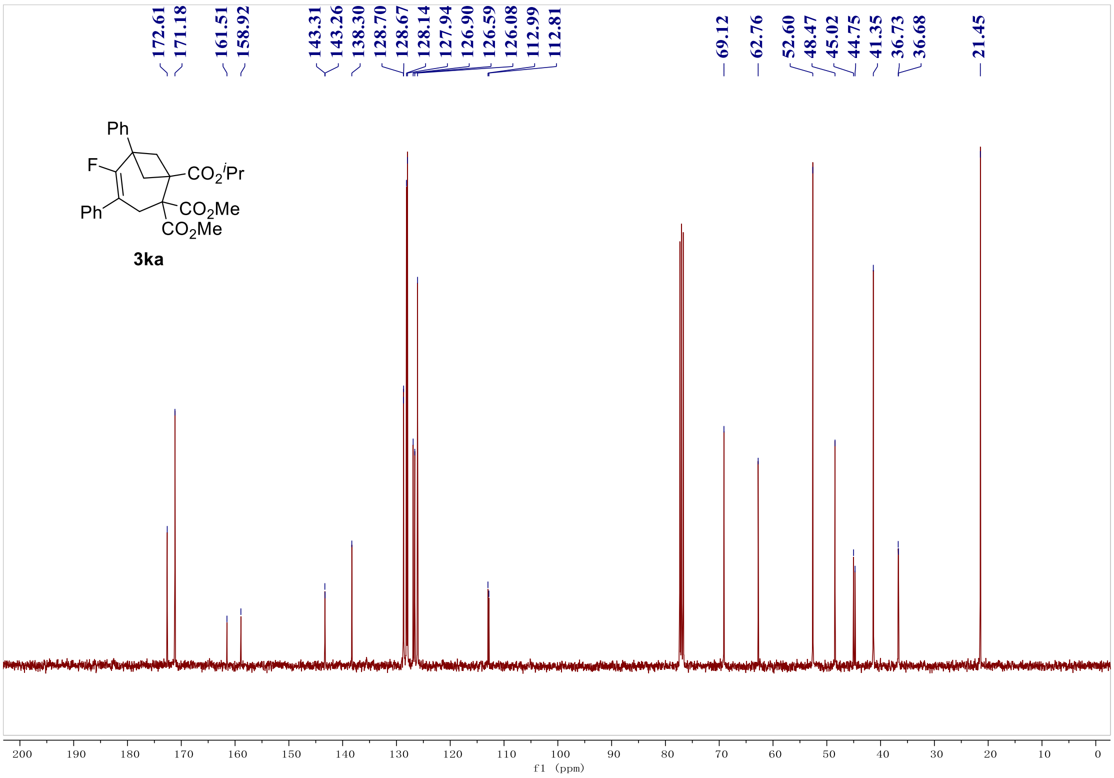

^{1}H NMR (400MHz, $CDCl_3$), ^{19}F NMR (376 MHz, $CDCl_3$) and ^{13}C NMR (100 MHz, $CDCl_3$) spectra of product **3la**

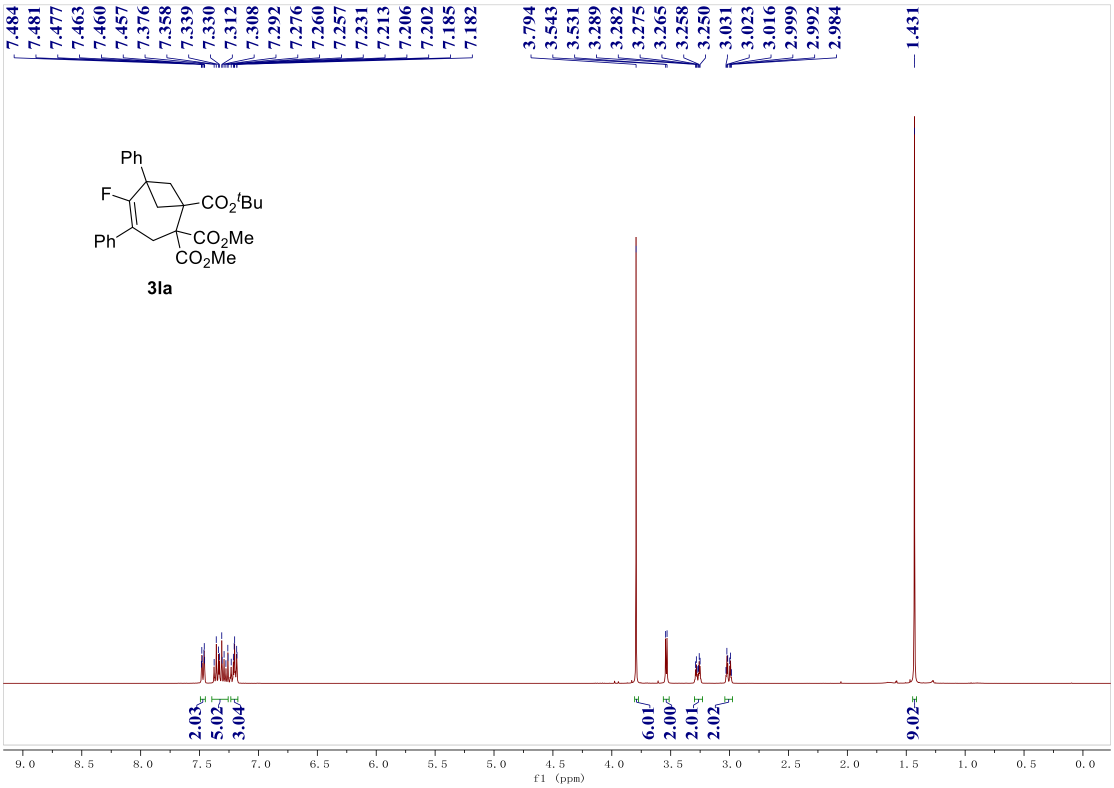

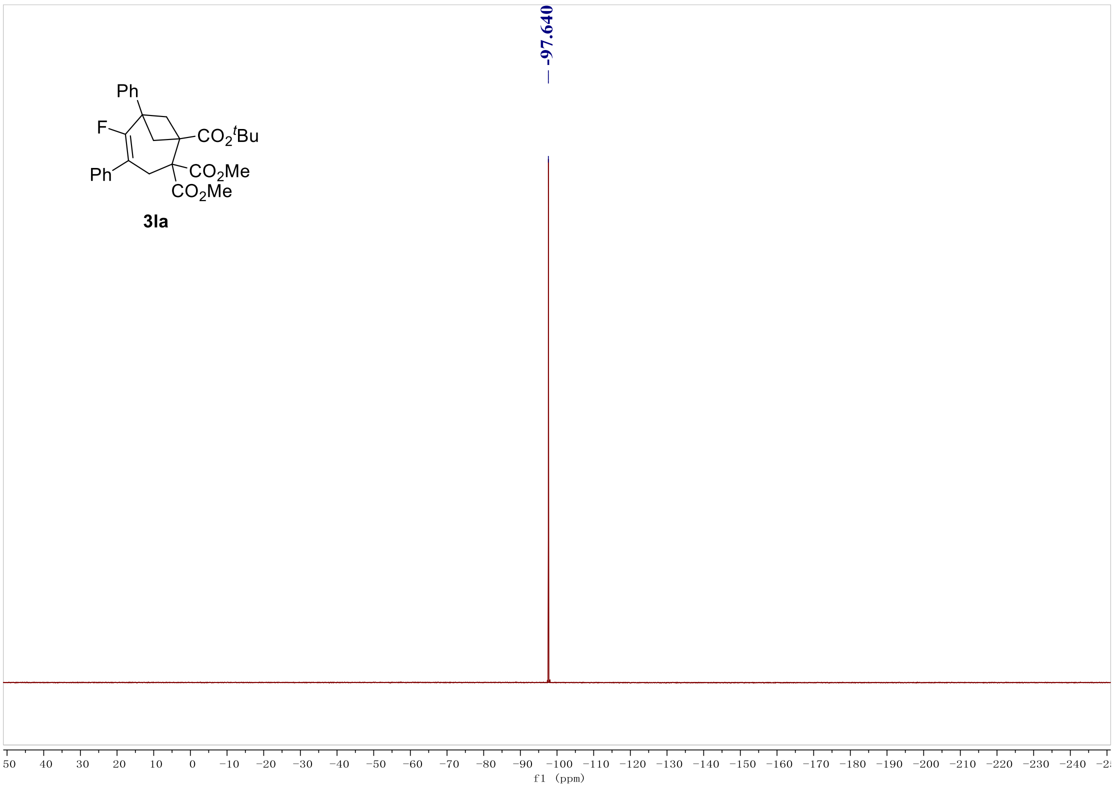
-97.640
Ph
F
CO2tBu
Ph
CO2Me
CO2Me
3la
f1 (ppm)

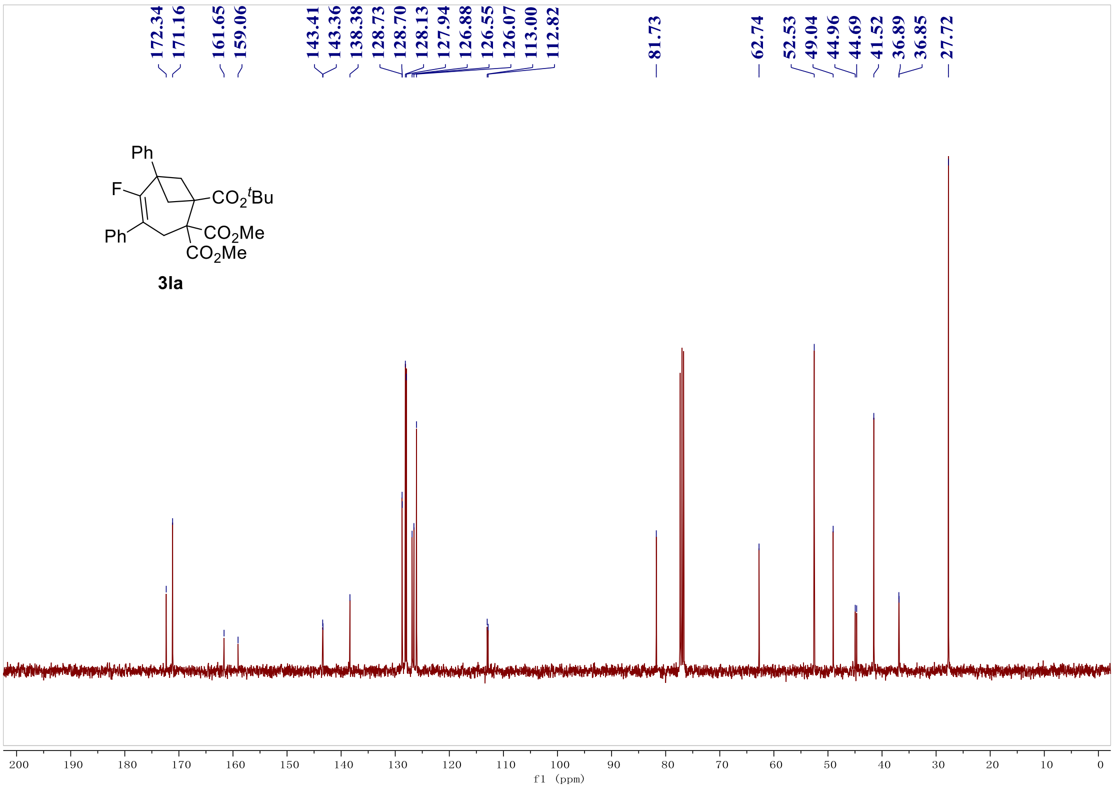
172.34
171.16
161.65
159.06
143.41
143.36
138.38
128.73
128.70
128.13
127.94
126.88
126.55
126.07
113.00
112.82
81.73
62.74
52.53
49.04
44.96
44.69
41.52
36.89
36.85
27.72
Ph
F
CO2tBu
Ph
CO2Me
CO2Me
3la
f1 (ppm)

^{1}H NMR (400MHz, $CDCl_3$), ^{19}F NMR (376 MHz, $CDCl_3$) and ^{13}C NMR (100 MHz, $CDCl_3$) spectra of product **3ma**

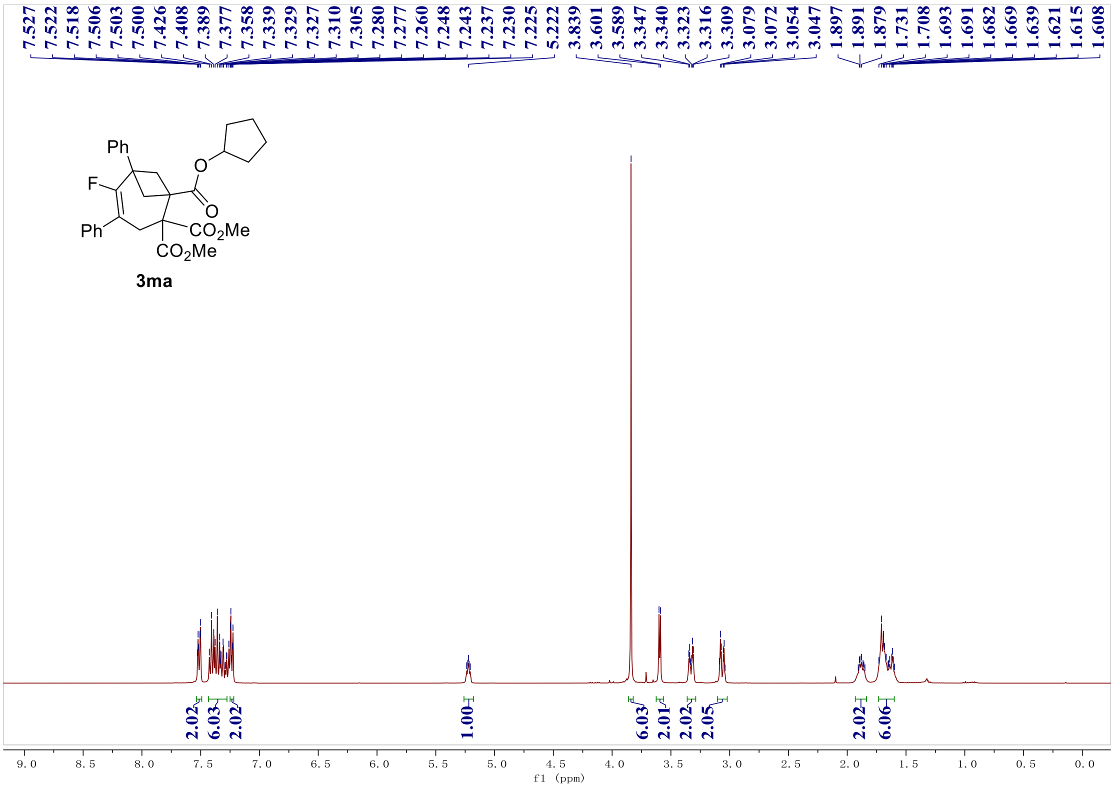

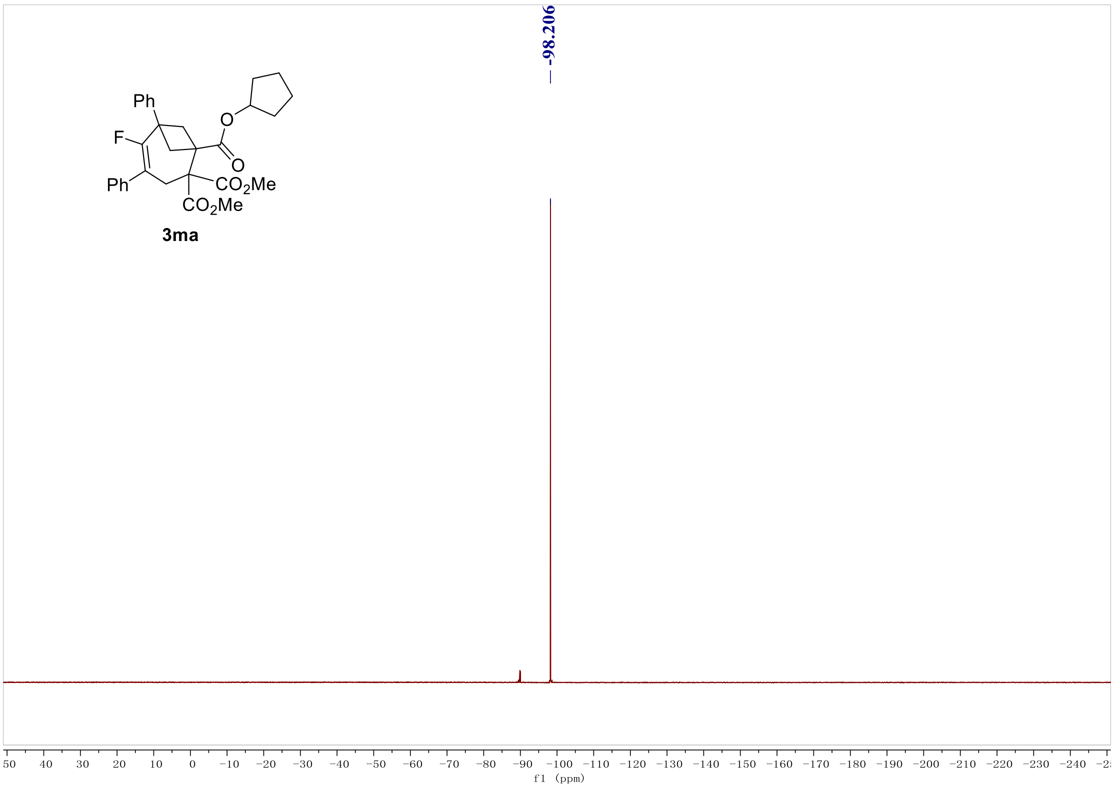

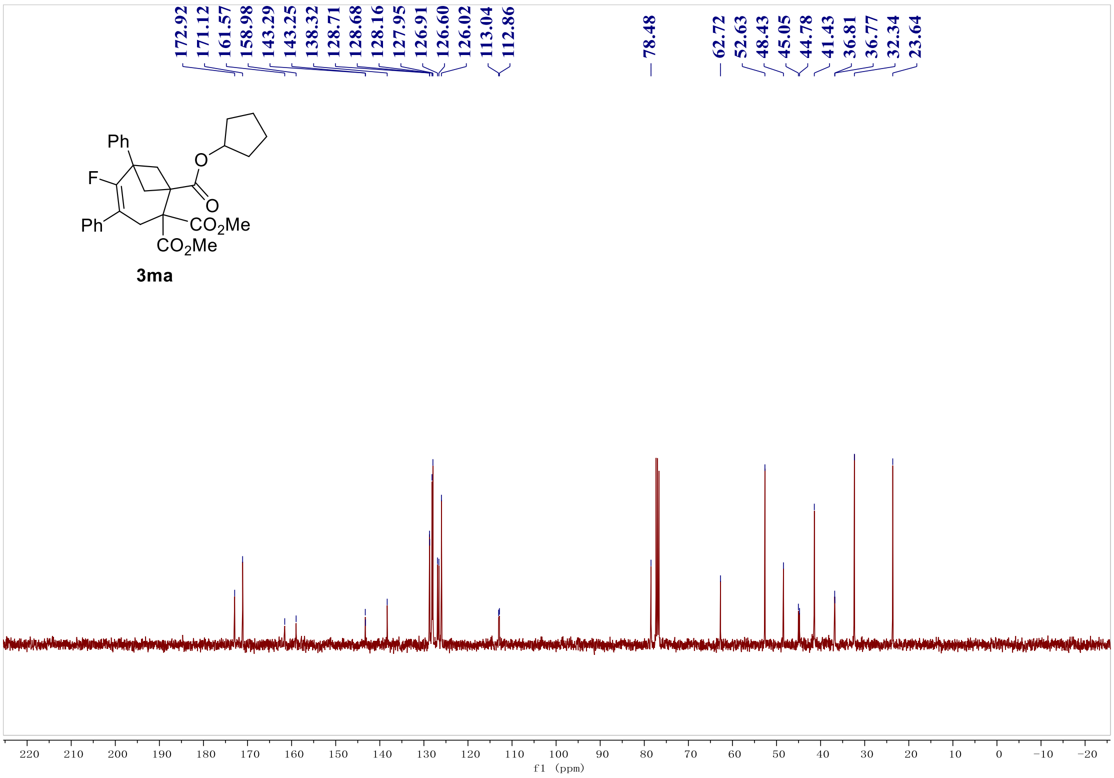

^{1}H NMR (400MHz, $CDCl_3$), ^{19}F NMR (376 MHz, $CDCl_3$) and ^{13}C NMR (100 MHz, $CDCl_3$) spectra of product **3na**

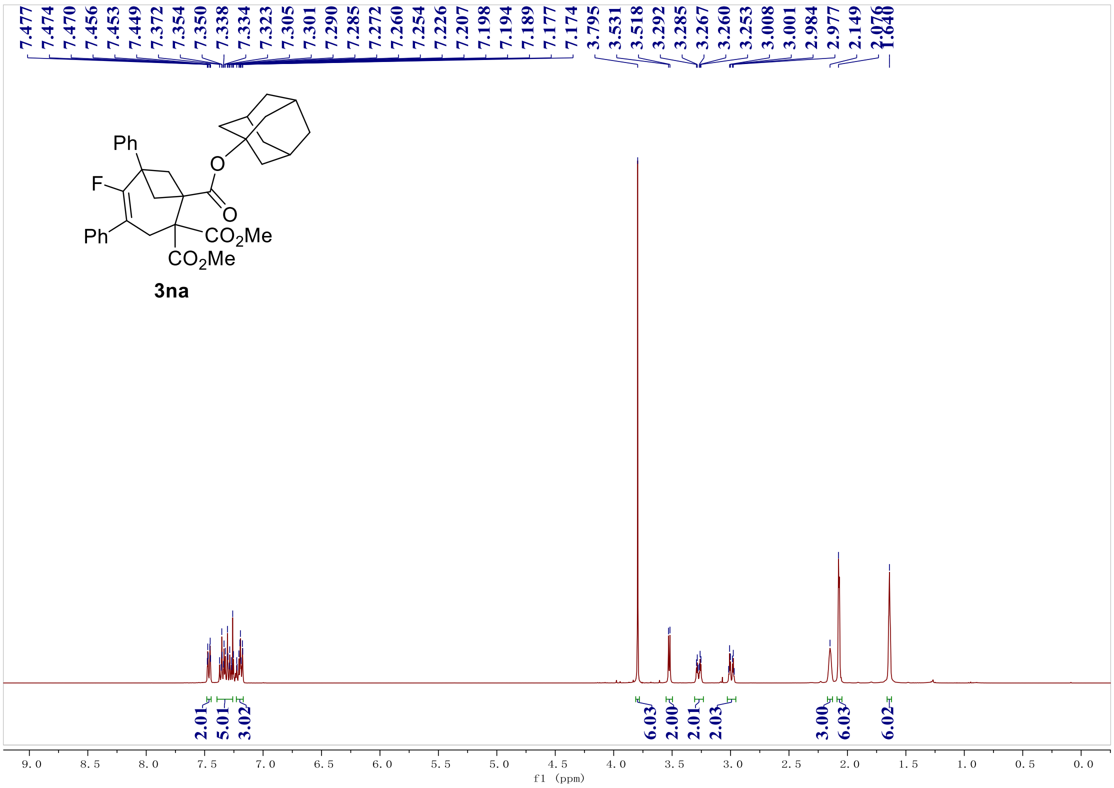

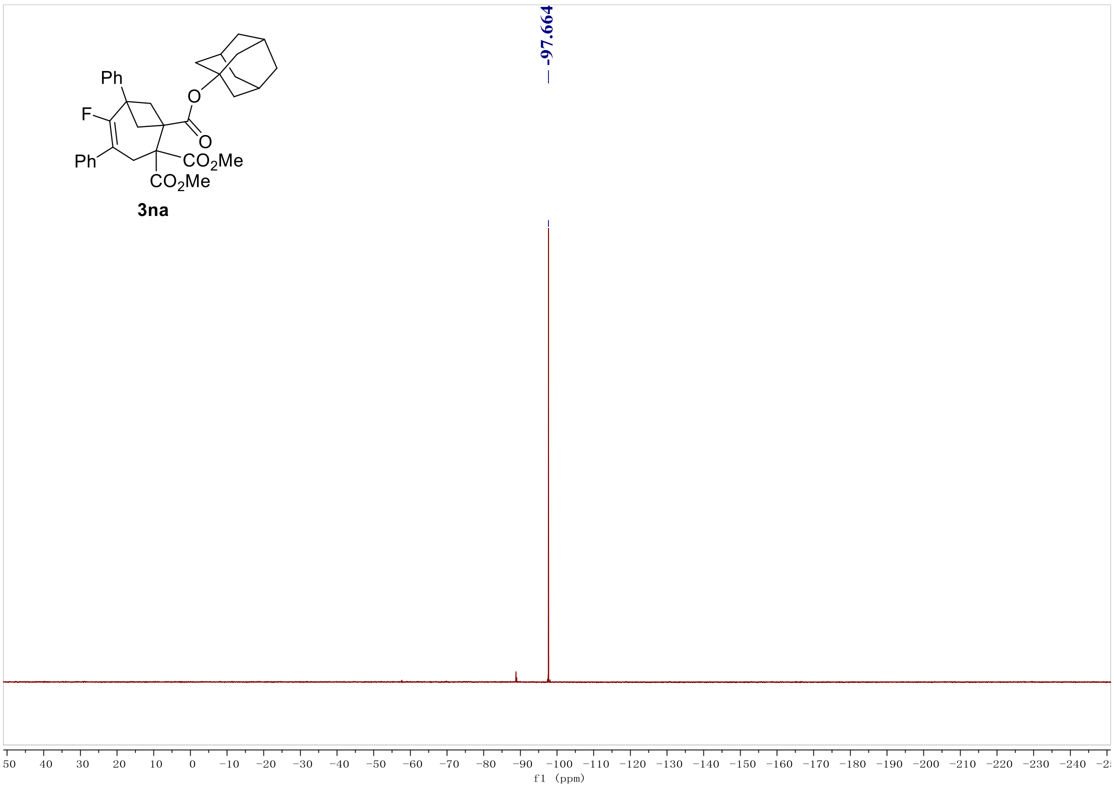
-97.664
Ph
F
Ph
O
O
CO2Me
CO2Me
3na
50 40 30 20 10 0 -10 -20 -30 -40 -50 -60 -70 -80 -90 -100 -110 -120 -130 -140 -150 -160 -170 -180 -190 -200 -210 -220 -230 -240
f1 (ppm)

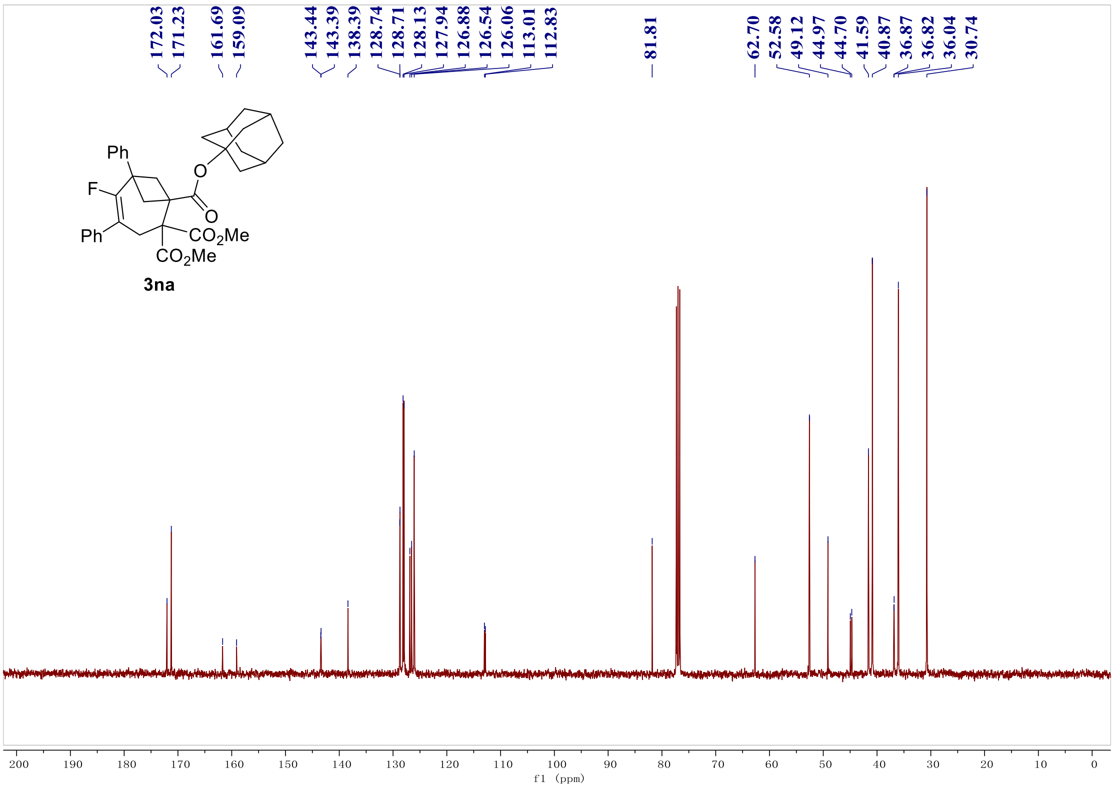
172.03
171.23
161.69
159.09
143.44
143.39
138.39
128.74
128.71
128.13
127.94
126.88
126.54
126.06
113.01
112.83
81.81
62.70
52.58
49.12
44.97
44.70
41.59
40.87
36.87
36.82
36.04
30.74
Ph
F
Ph
O
O
CO2Me
CO2Me
3na
200 190 180 170 160 150 140 130 120 110 100 90 80 70 60 50 40 30 20 10 0
f1 (ppm)

^{1}H NMR (400MHz, $CDCl_3$), ^{19}F NMR (376 MHz, $CDCl_3$) and ^{13}C NMR (100 MHz, $CDCl_3$) spectra of product **3oa**

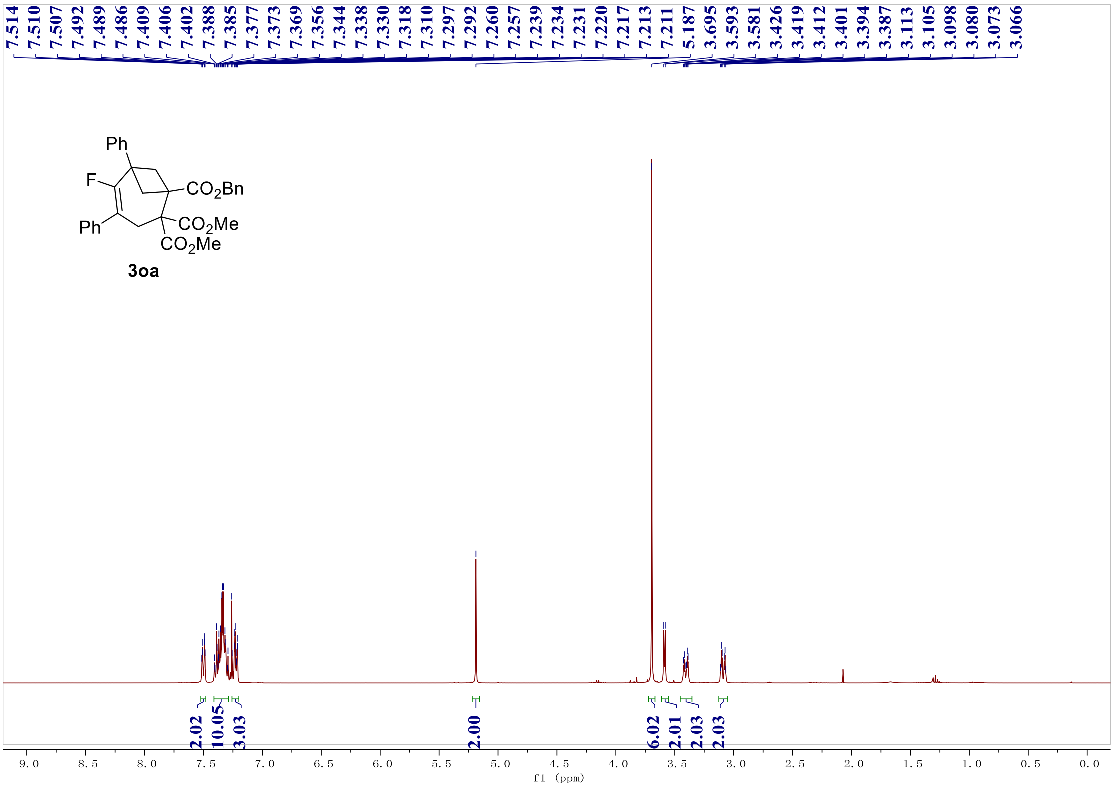

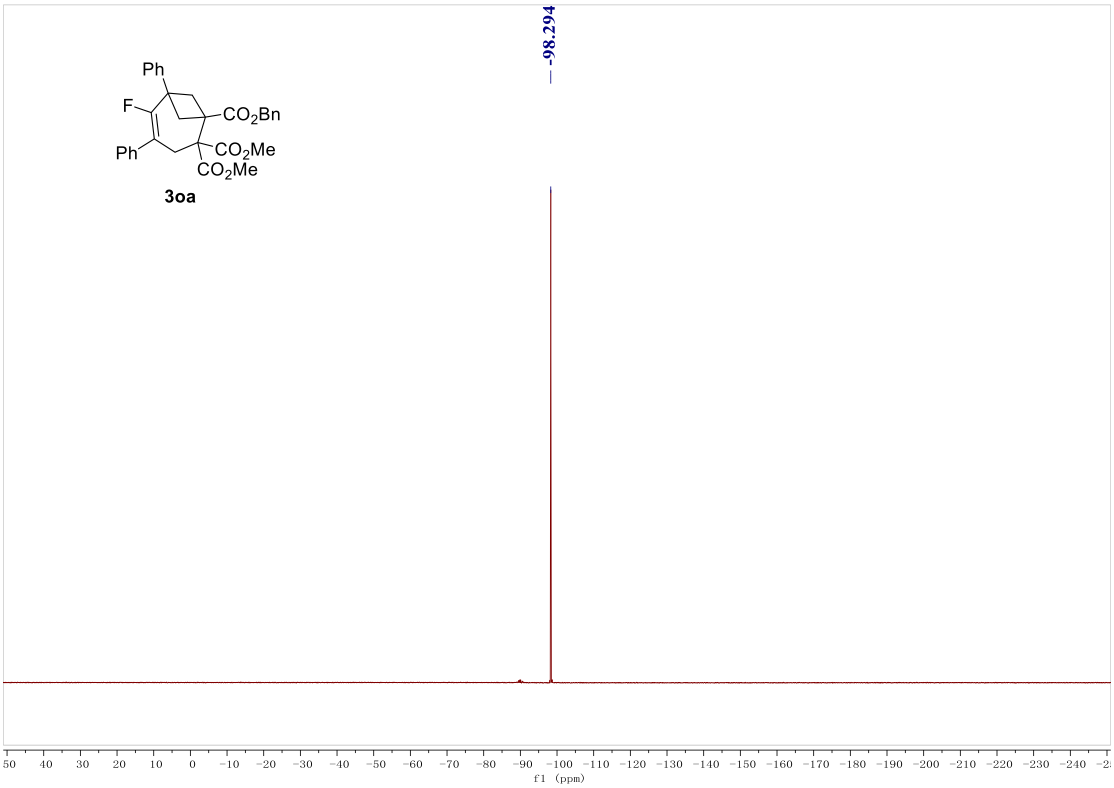

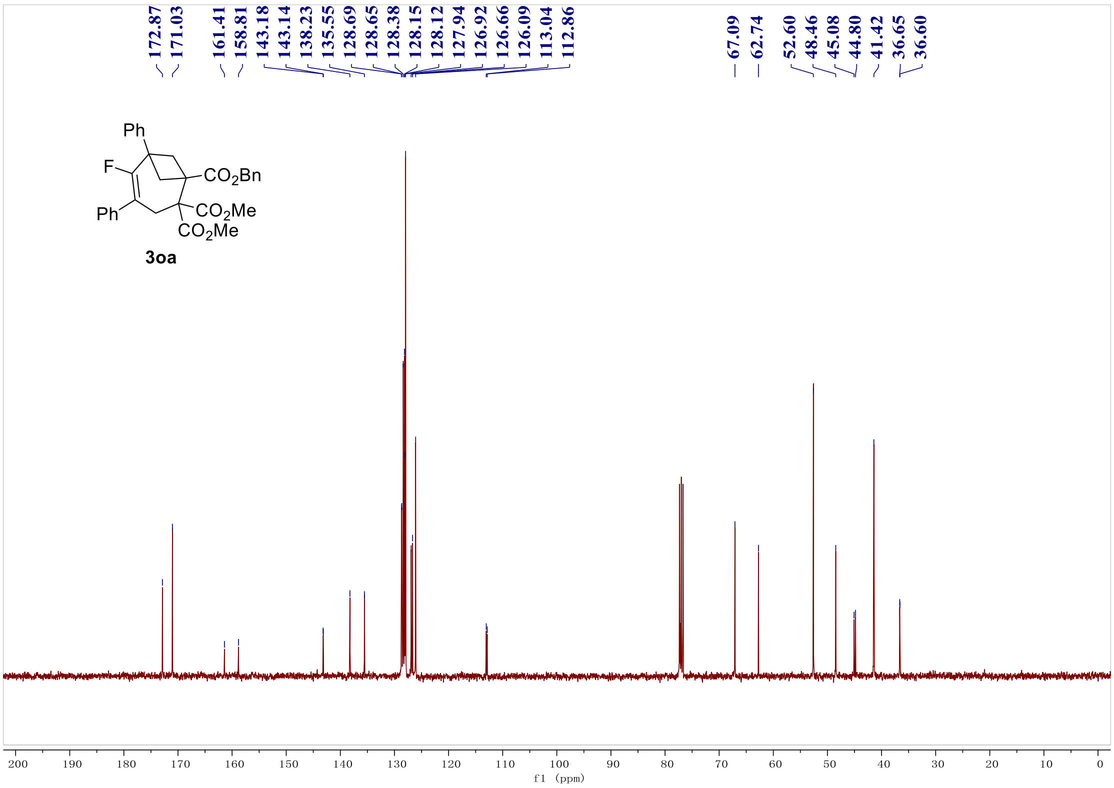

^{1}H NMR (400MHz, $CDCl_3$), ^{19}F NMR (376 MHz, $CDCl_3$) and ^{13}C NMR (100 MHz, $CDCl_3$) spectra of product **3pa**

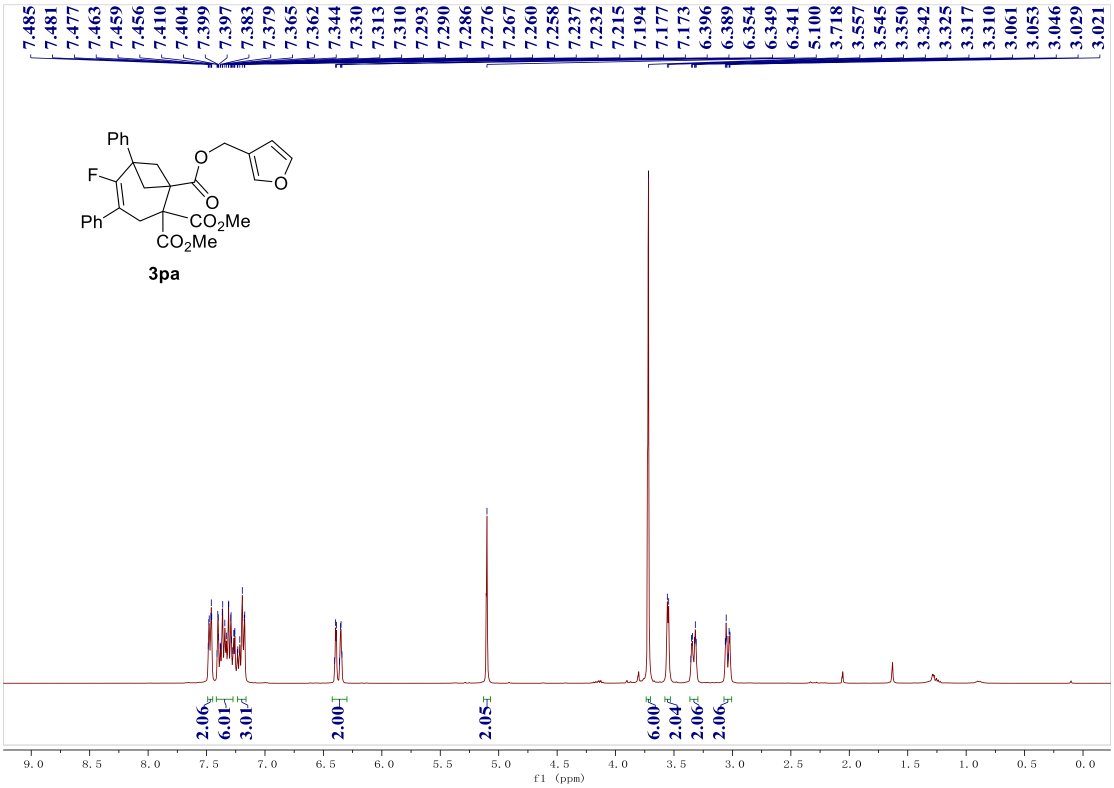

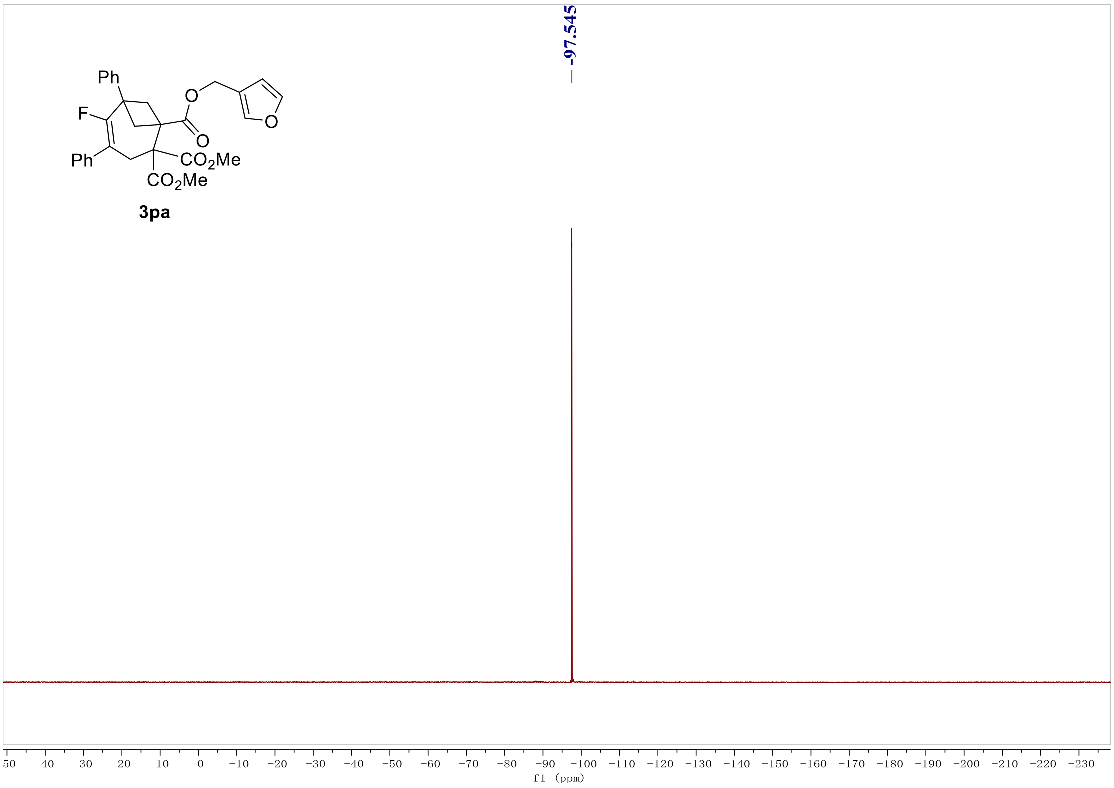
-97.545
Ph
F
Ph
O
O
CO2Me
CO2Me
O
3pa
f1 (ppm)

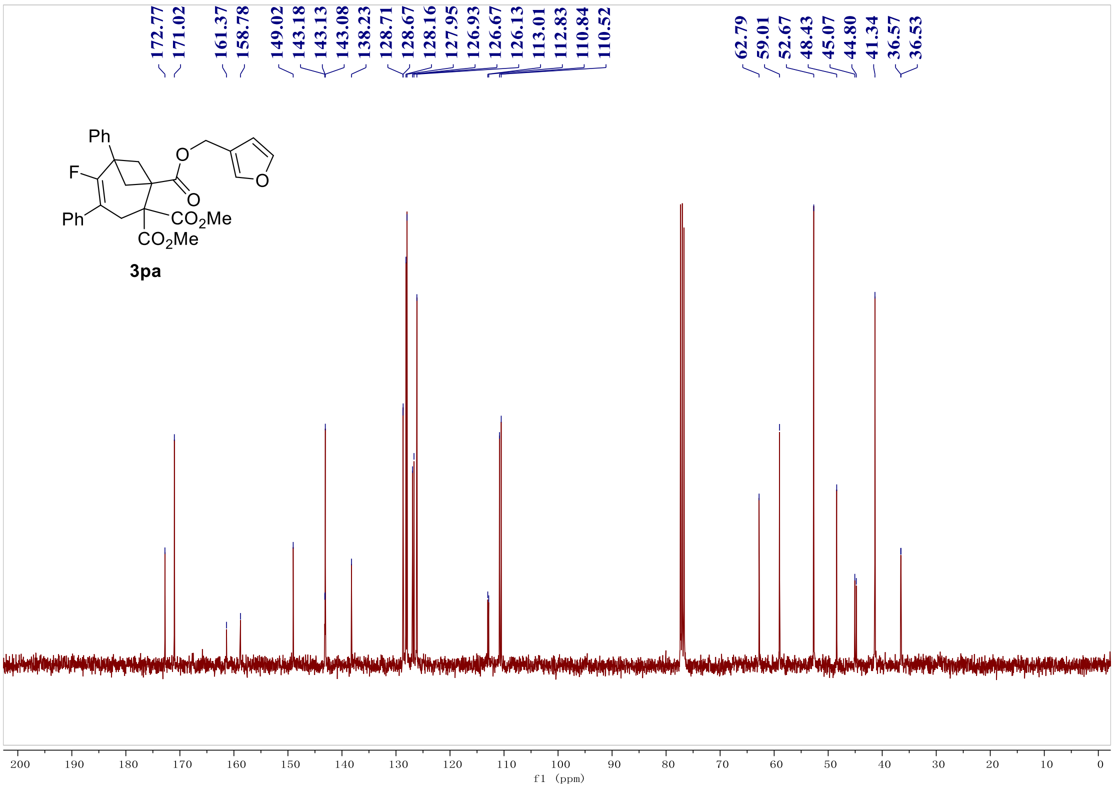
172.77
171.02
161.37
158.78
149.02
143.18
143.13
143.08
138.23
128.71
128.67
128.16
127.95
126.93
126.67
126.13
113.01
112.83
110.84
110.52
62.79
59.01
52.67
48.43
45.07
44.80
41.34
36.57
36.53
Ph
F
Ph
O
O
CO2Me
CO2Me
O
3pa
f1 (ppm)

^{1}H NMR (400MHz, $CDCl_3$), ^{19}F NMR (376 MHz, $CDCl_3$) and ^{13}C NMR (100 MHz, $CDCl_3$) spectra of product **3qa**

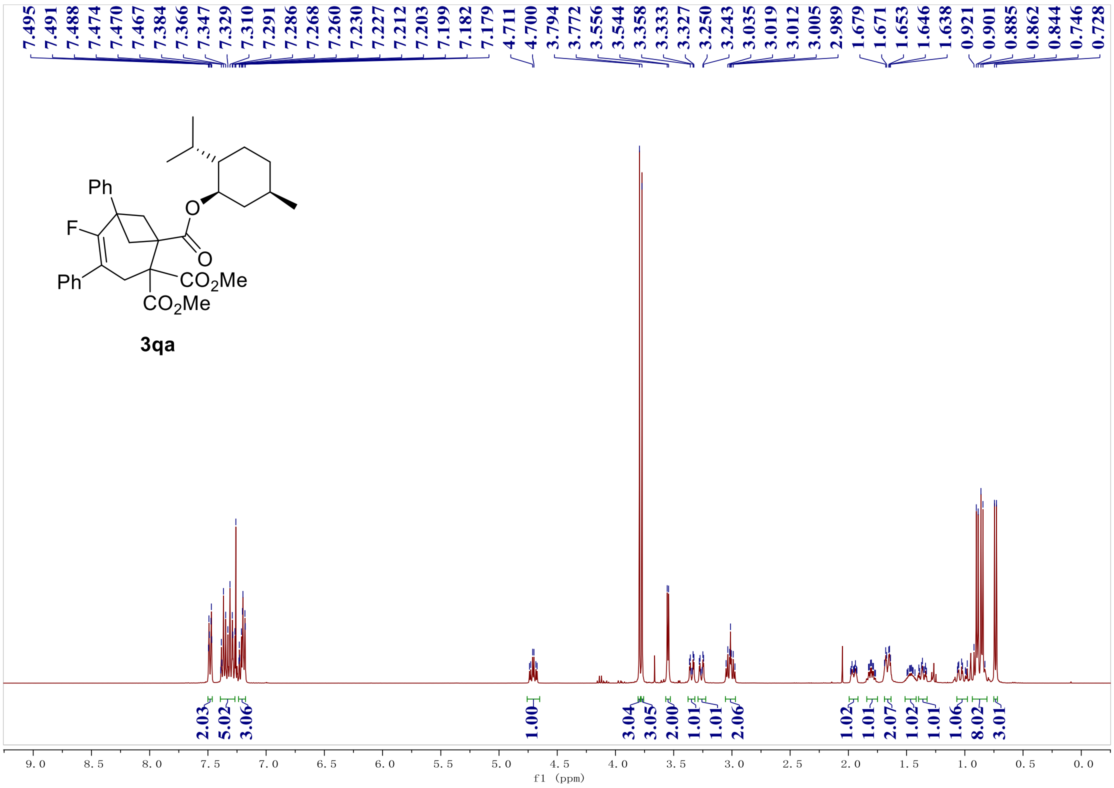

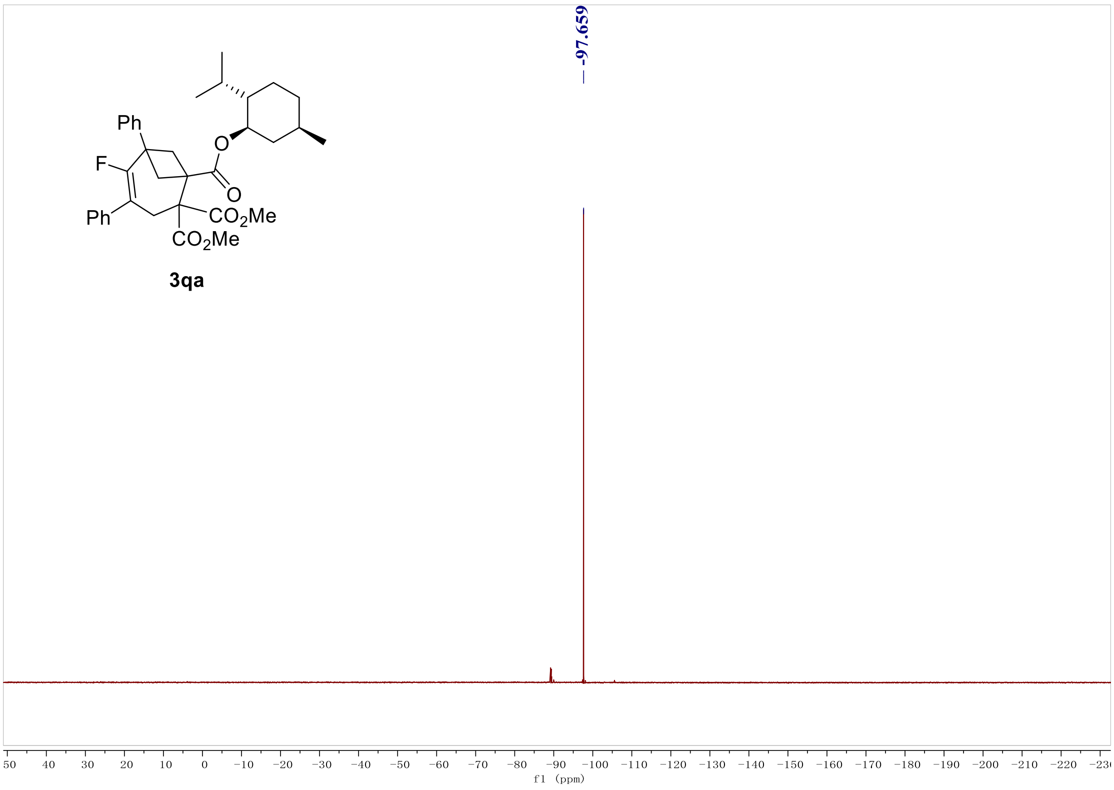

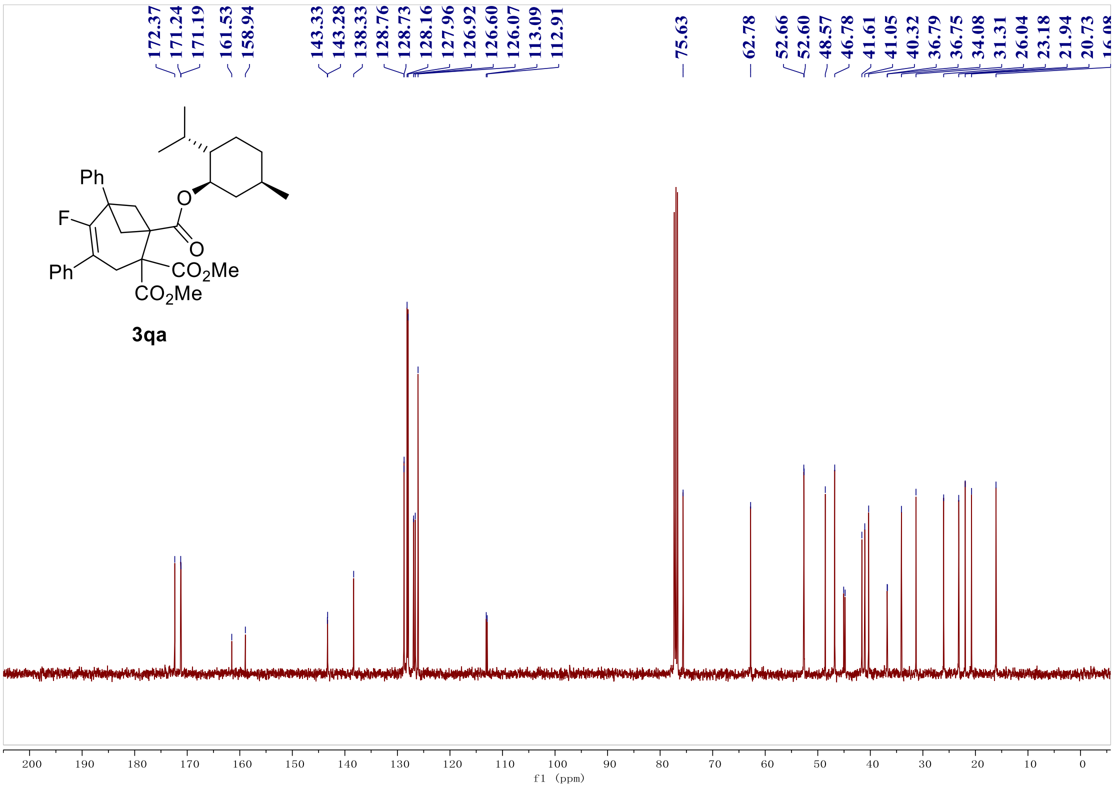

^{1}H NMR (400MHz, $CDCl_3$), ^{19}F NMR (376 MHz, $CDCl_3$) and ^{13}C NMR (100 MHz, $CDCl_3$) spectra of product **3ra**

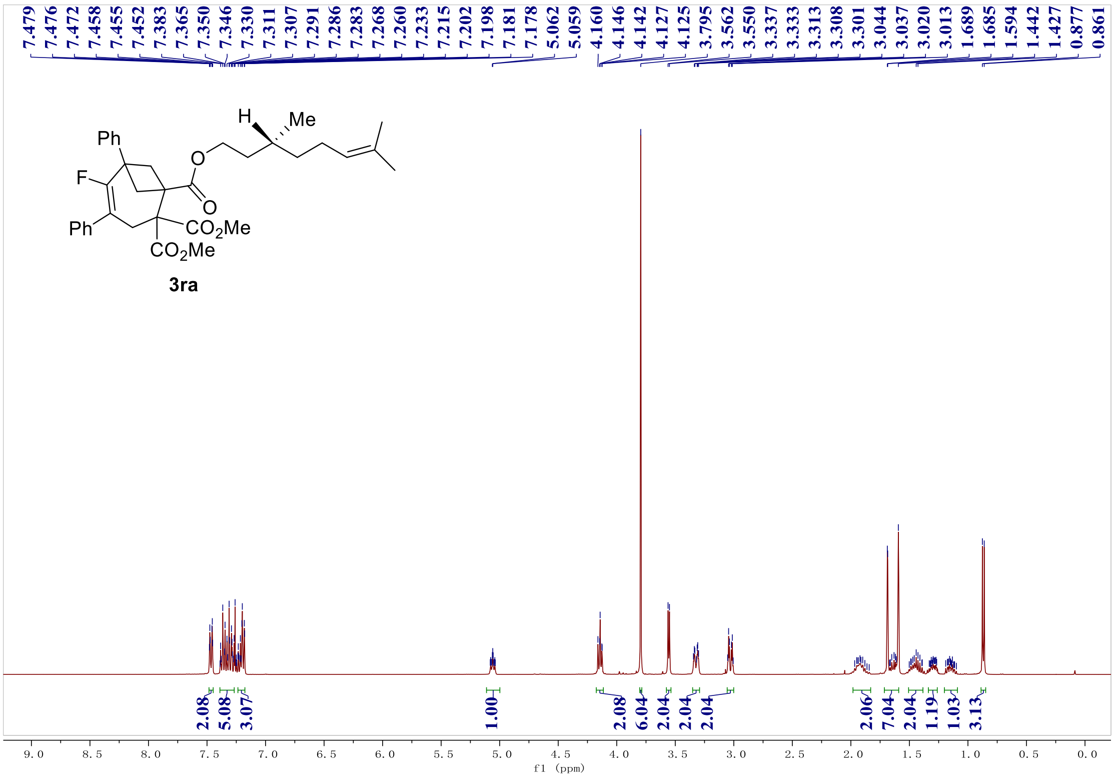

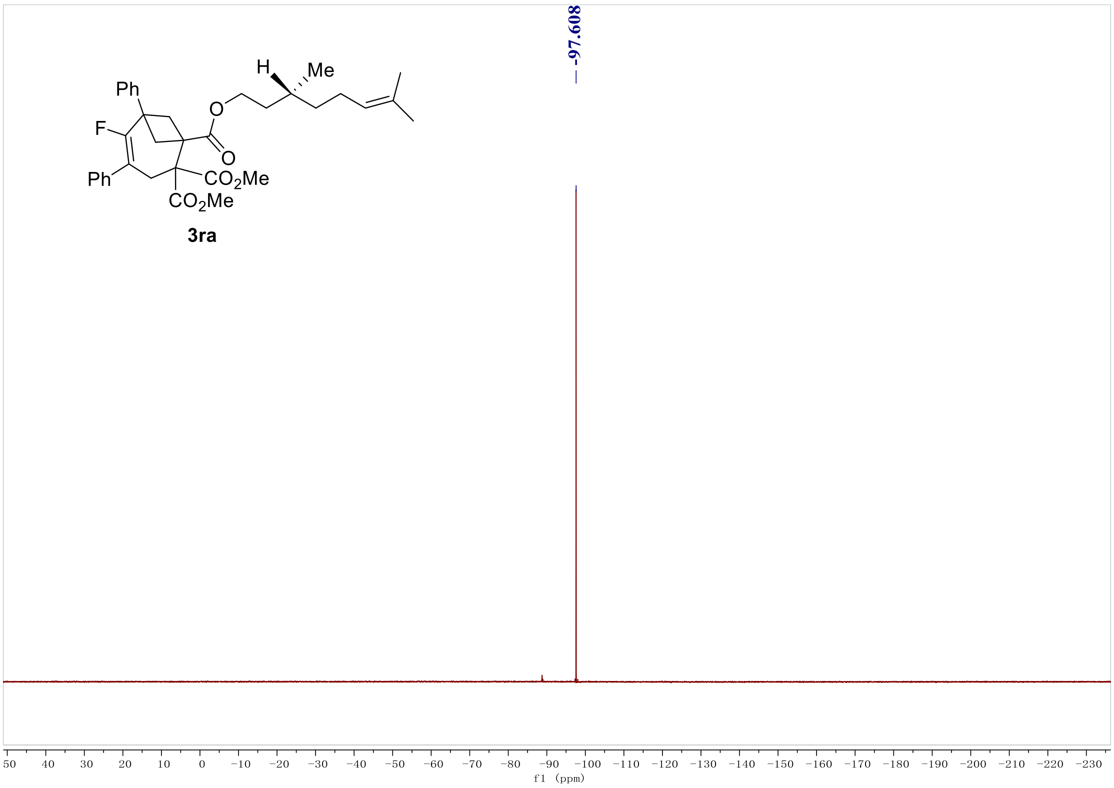

3ra
-97.608
f1 (ppm)

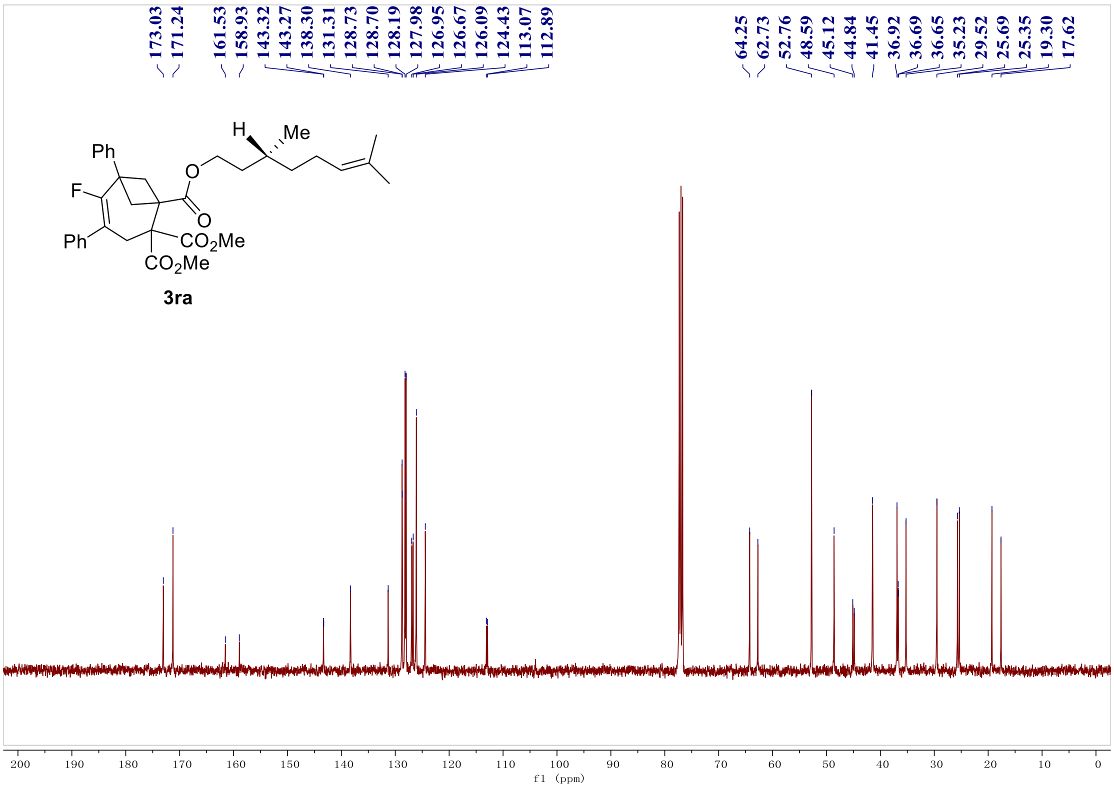

3ra
173.03
171.24
161.53
158.93
143.32
143.27
138.30
131.31
128.73
128.70
128.19
127.98
126.95
126.67
126.09
124.43
113.07
112.89
64.25
62.73
52.76
48.59
45.12
44.84
41.45
36.92
36.69
36.65
35.23
29.52
25.69
25.35
19.30
17.62
f1 (ppm)

^{1}H NMR (400MHz, $CDCl_3$), ^{19}F NMR (376 MHz, $CDCl_3$) and ^{13}C NMR (100 MHz, $CDCl_3$) spectra of product **3sa**

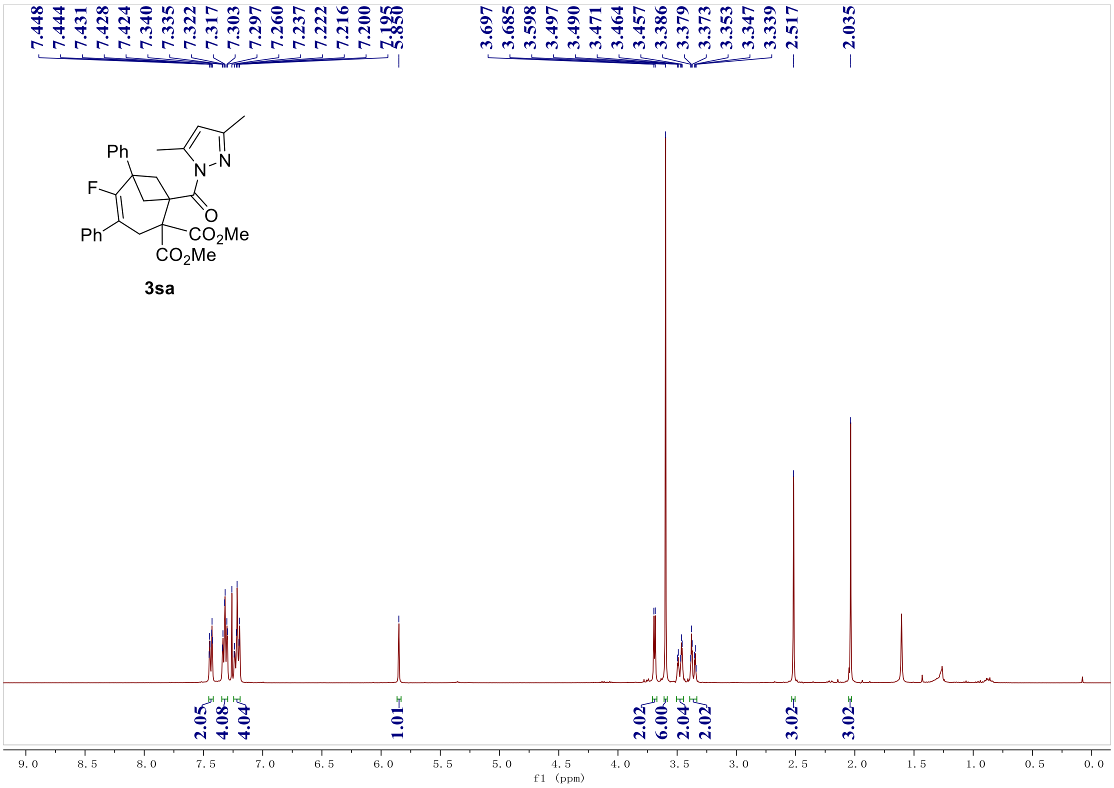

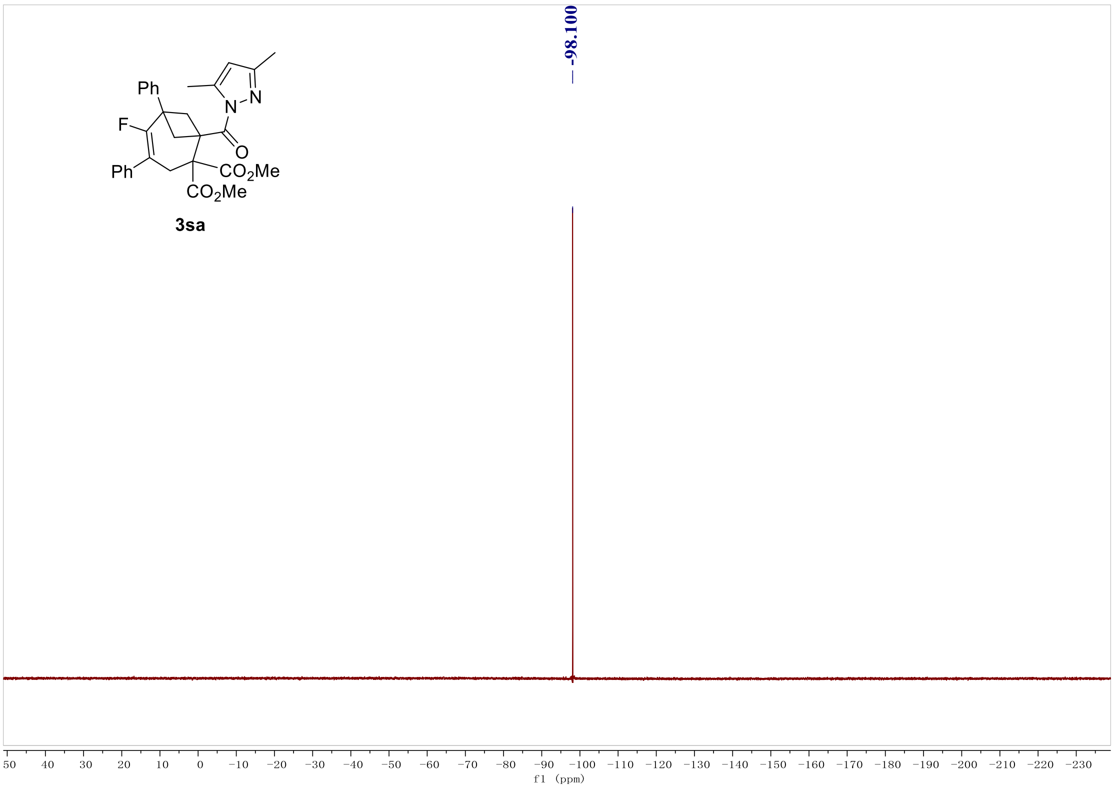

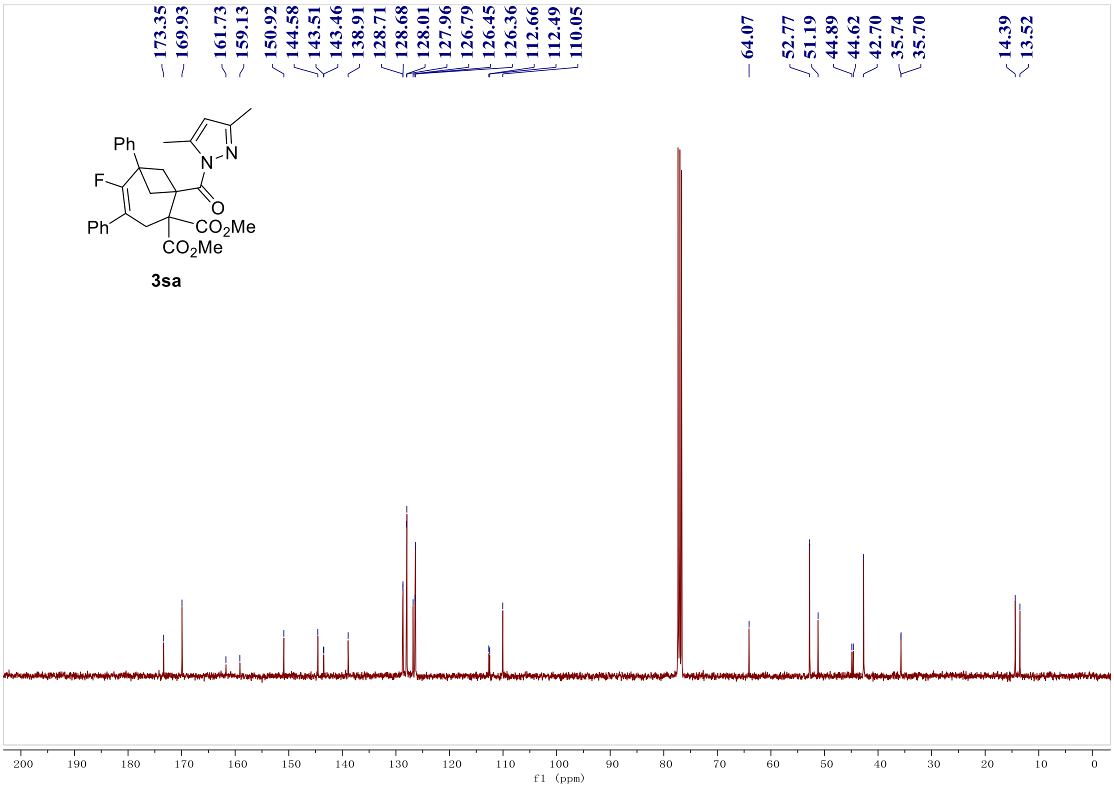

^{1}H NMR (400MHz, $CDCl_3$), ^{19}F NMR (376 MHz, $CDCl_3$) and ^{13}C NMR (100 MHz, $CDCl_3$) spectra of product **3ab**

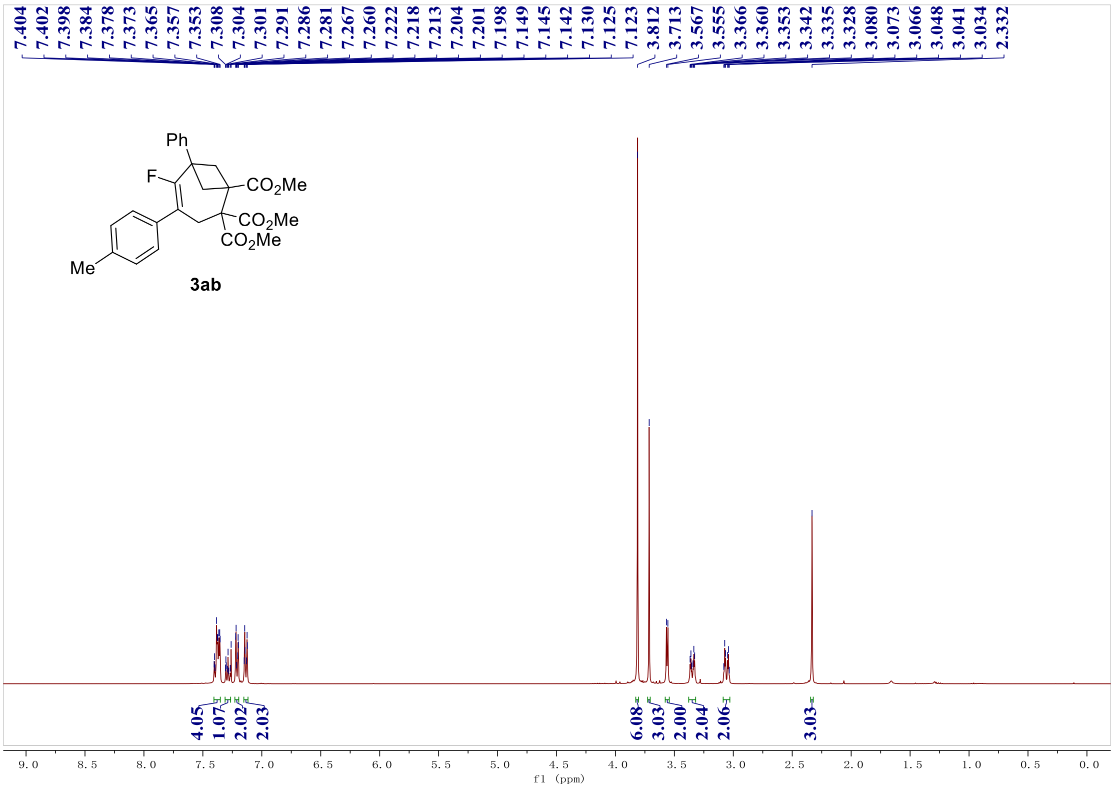

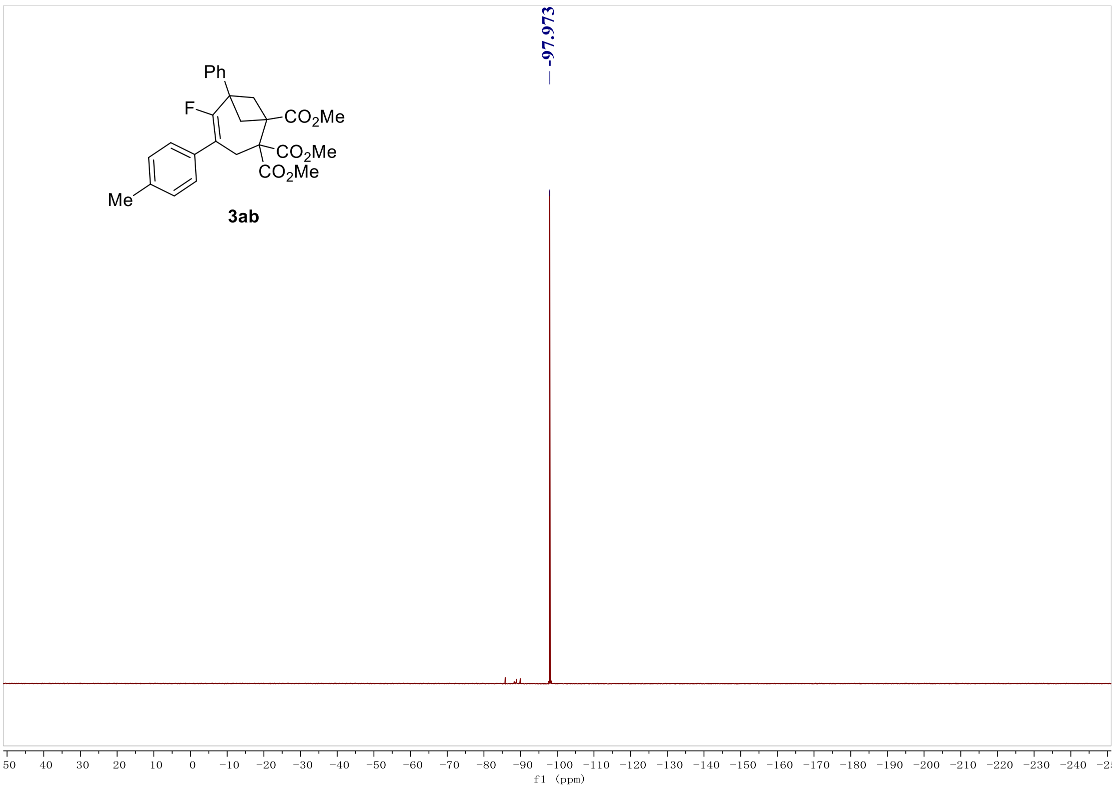
-97.973
Ph
F
CO_2Me
CO_2Me
CO_2Me
Me
3ab
f1 (ppm)

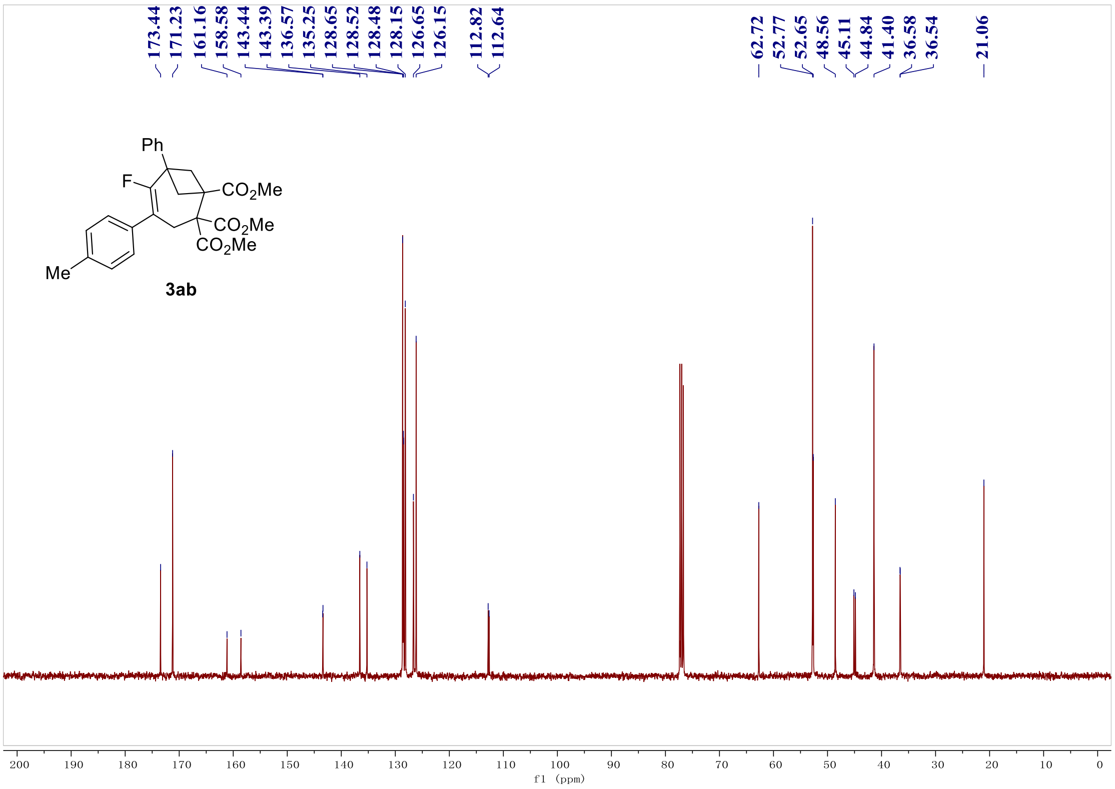
173.44
171.23
161.16
158.58
143.44
143.39
136.57
135.25
128.65
128.52
128.48
128.15
126.65
126.15
112.82
112.64
62.72
52.77
52.65
48.56
45.11
44.84
41.40
36.58
36.54
21.06
Ph
F
CO_2Me
CO_2Me
CO_2Me
Me
3ab
f1 (ppm)

^{1}H NMR (400MHz, $CDCl_3$), ^{19}F NMR (376 MHz, $CDCl_3$) and ^{13}C NMR (100 MHz, $CDCl_3$) spectra of product **3ac**

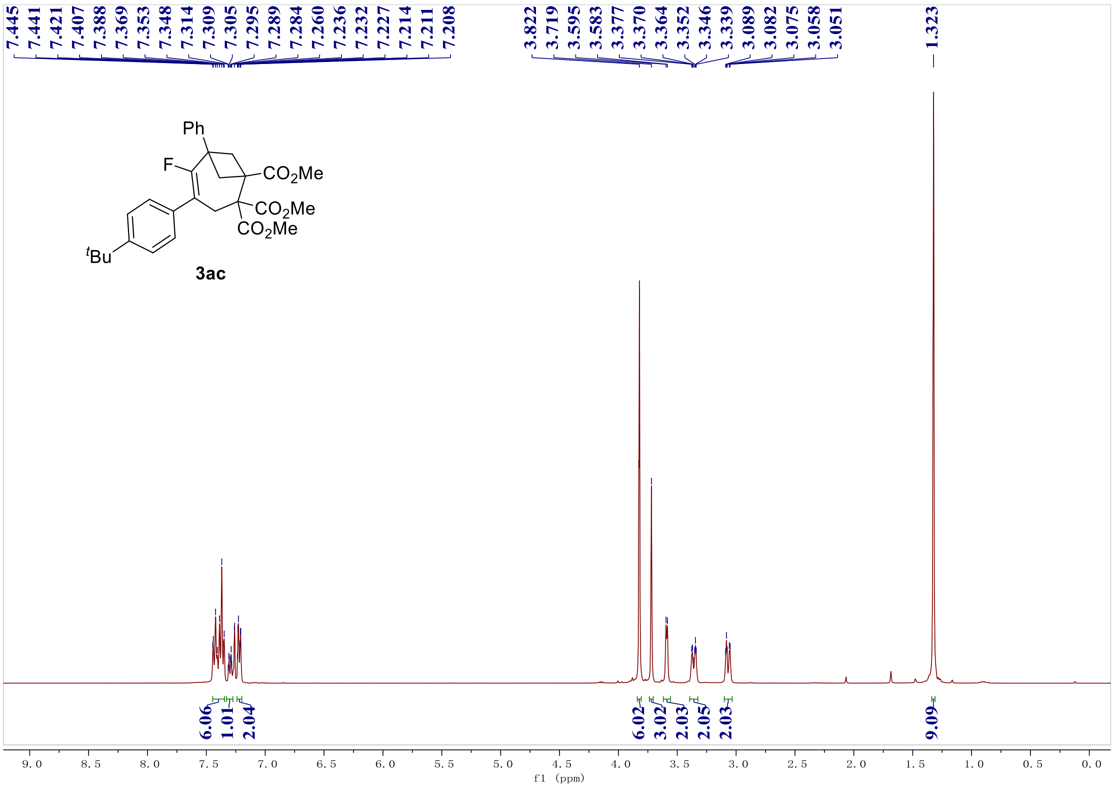

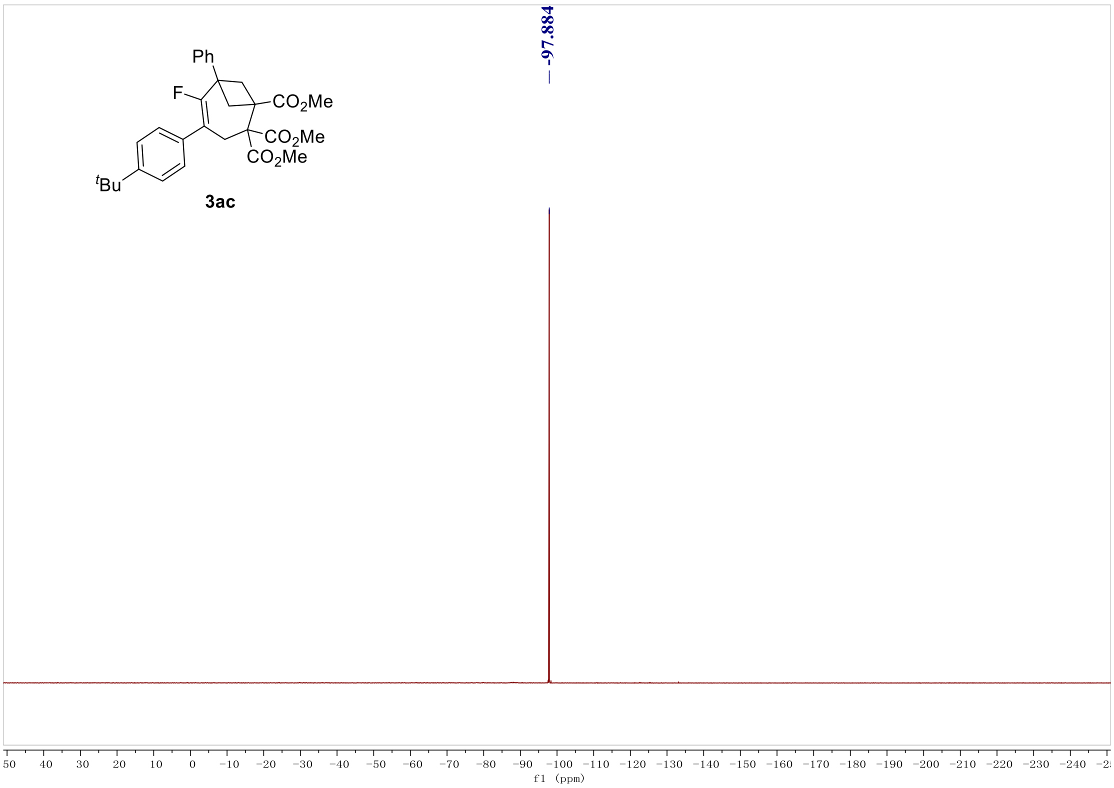

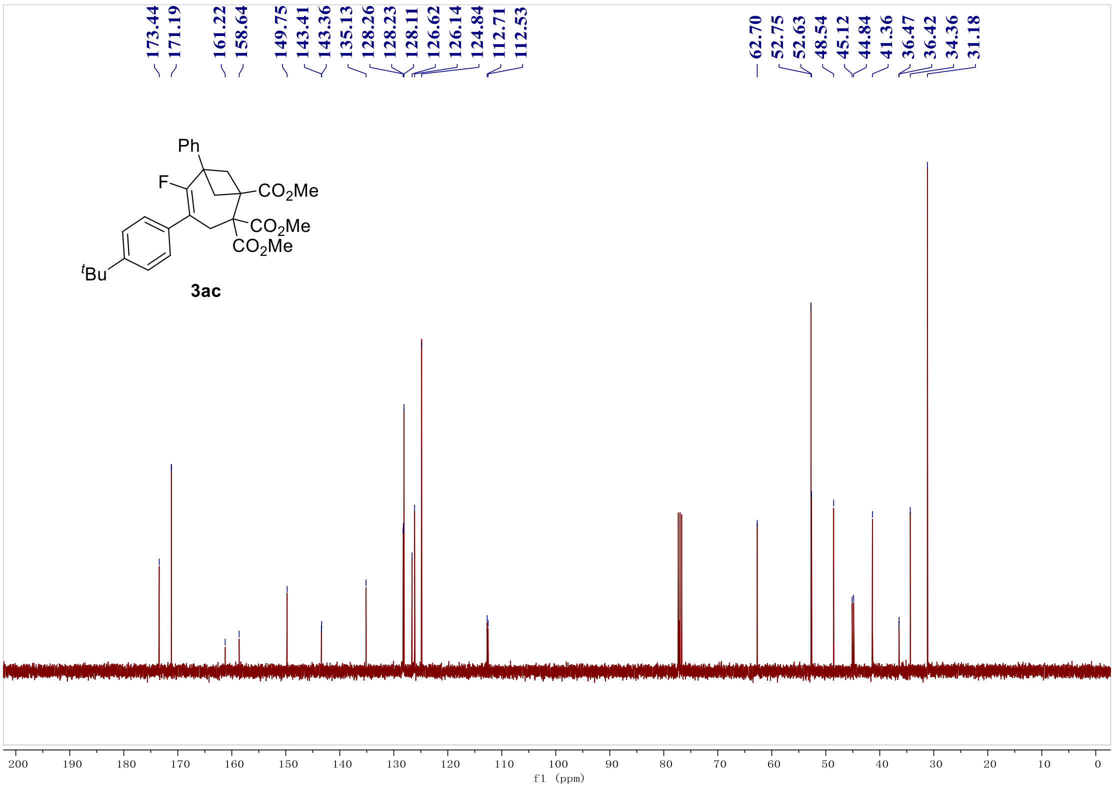

^{1}H NMR (400MHz, $CDCl_3$), ^{19}F NMR (376 MHz, $CDCl_3$) and ^{13}C NMR (100 MHz, $CDCl_3$) spectra of product **3ad**

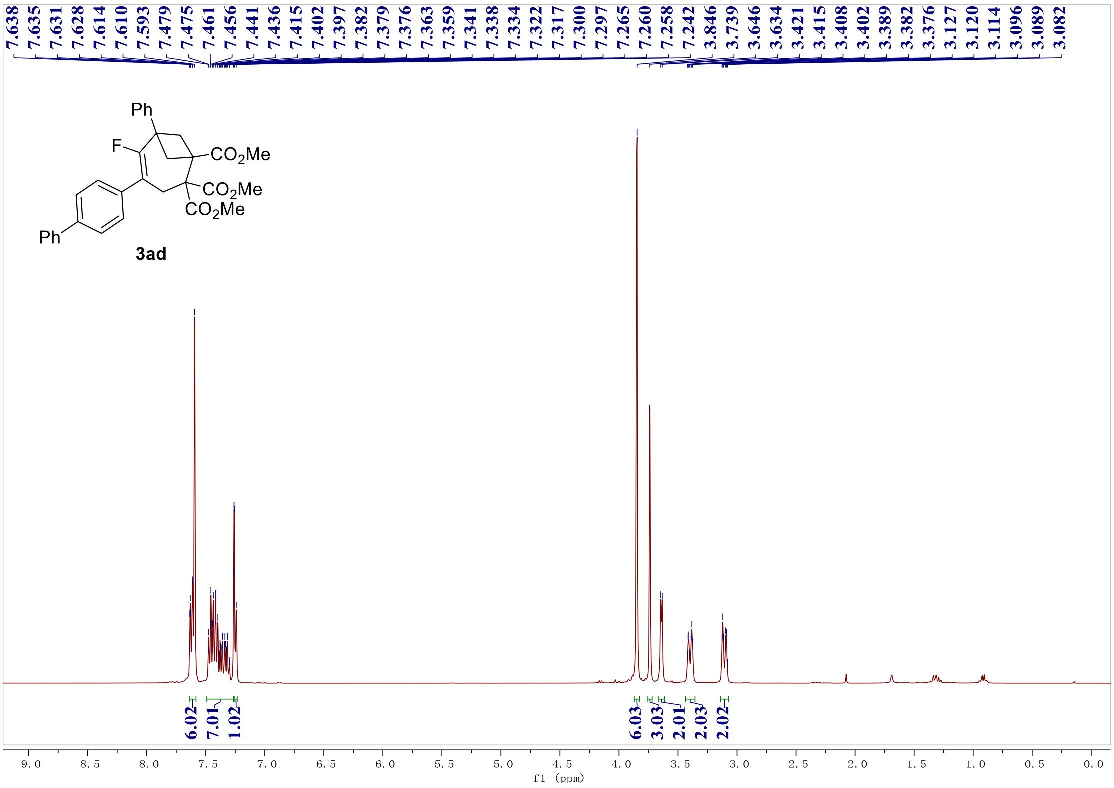

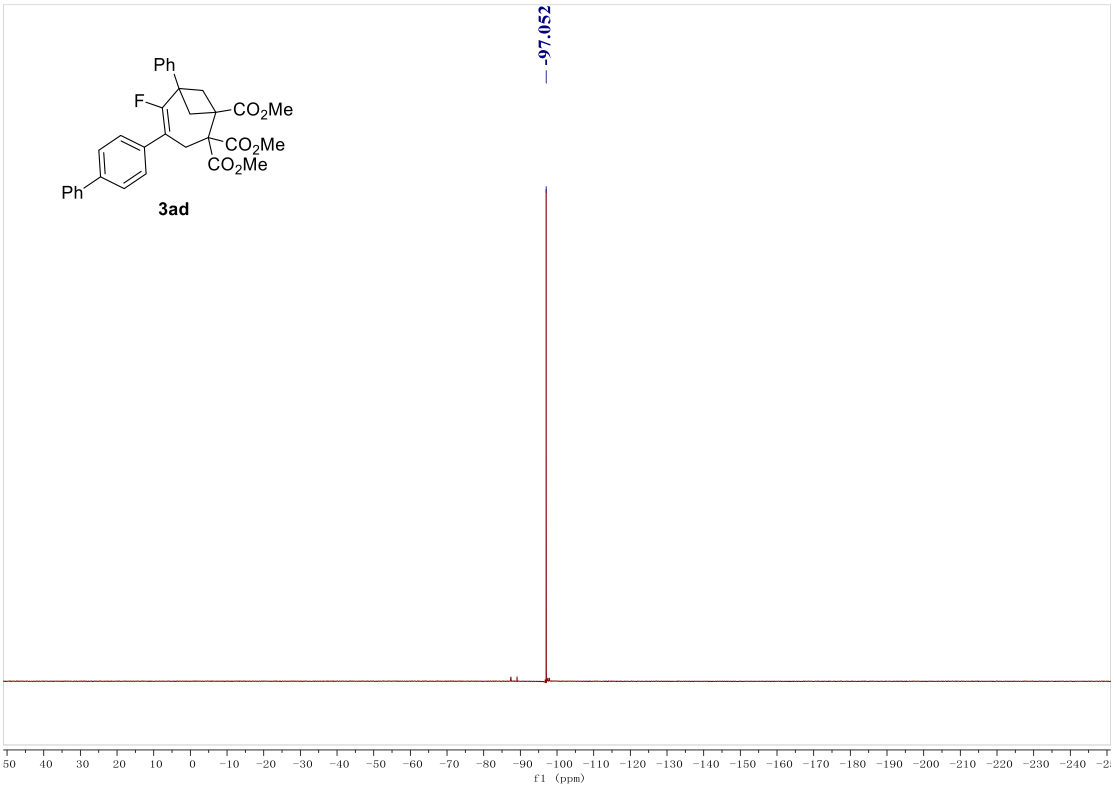
-97.052
Ph
F
CO2Me
CO2Me
CO2Me
Ph
3ad
f1 (ppm)

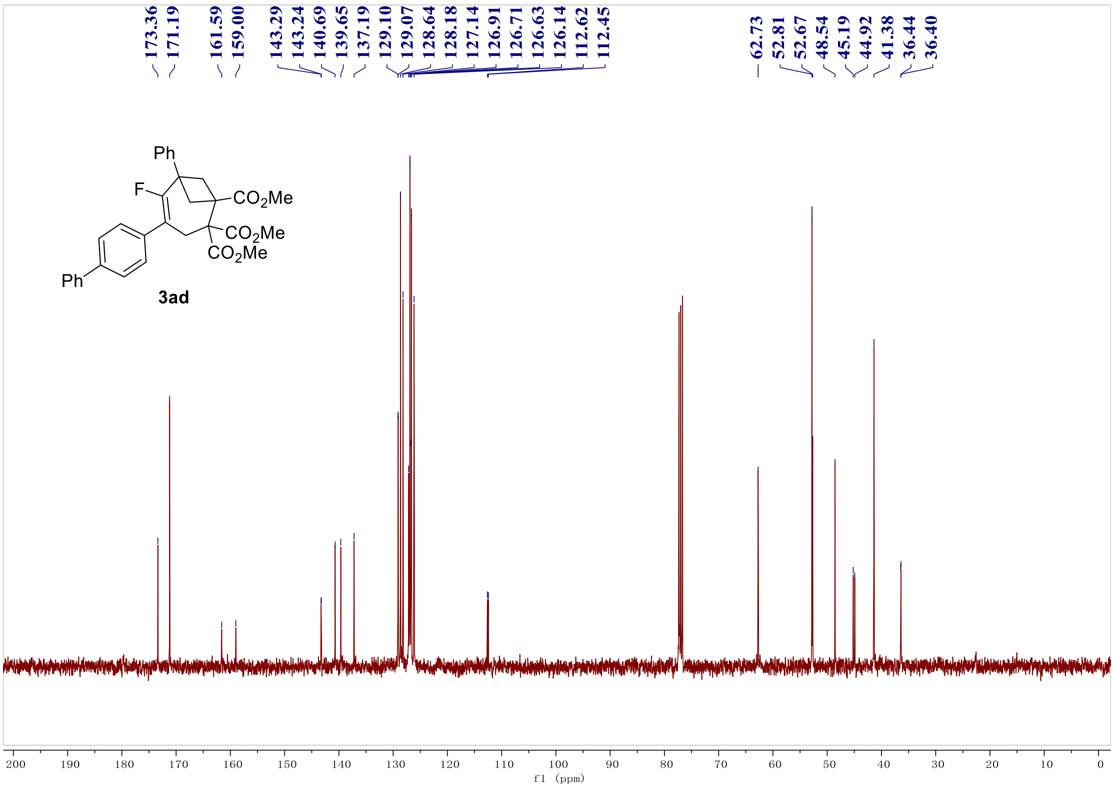
173.36
171.19
161.59
159.00
143.29
143.24
140.69
139.65
137.19
129.10
129.07
128.64
128.18
127.14
126.91
126.71
126.63
126.14
112.62
112.45
62.73
52.81
52.67
48.54
45.19
44.92
41.38
36.44
36.40
Ph
F
CO2Me
CO2Me
CO2Me
Ph
3ad
f1 (ppm)

^{1}H NMR (400MHz, $CDCl_3$), ^{19}F NMR (376 MHz, $CDCl_3$) and ^{13}C NMR (100 MHz, $CDCl_3$) spectra of product **3ae**

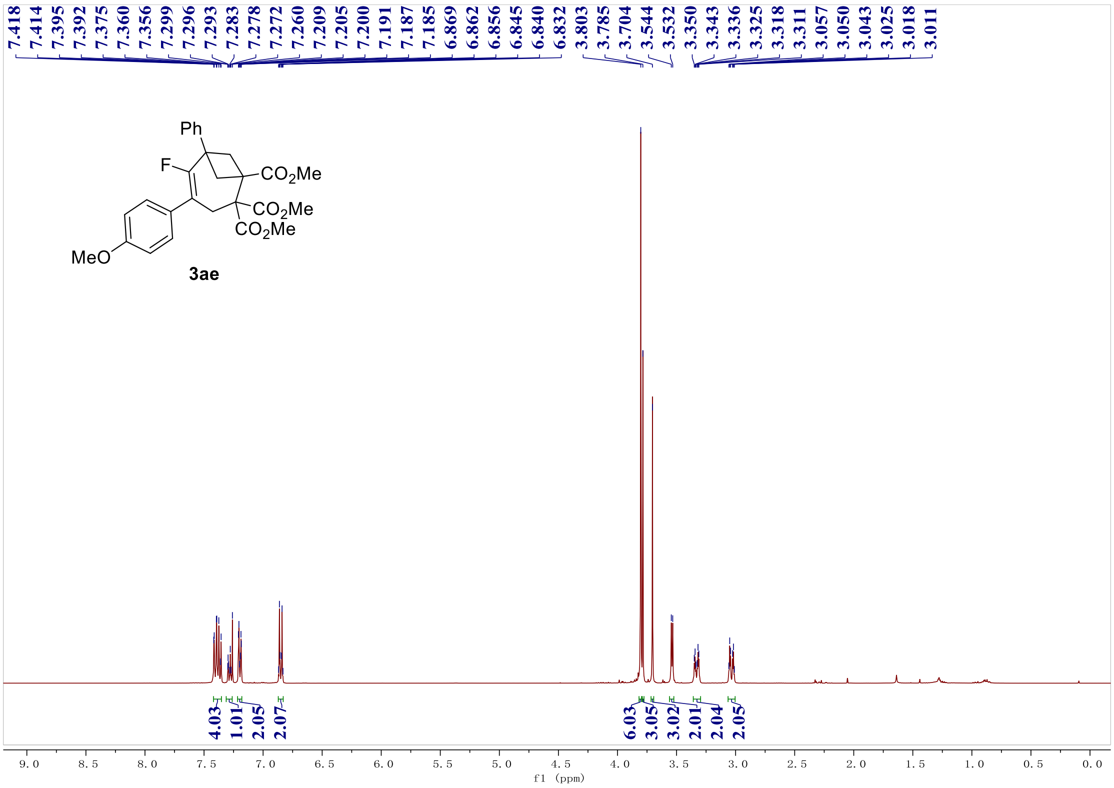

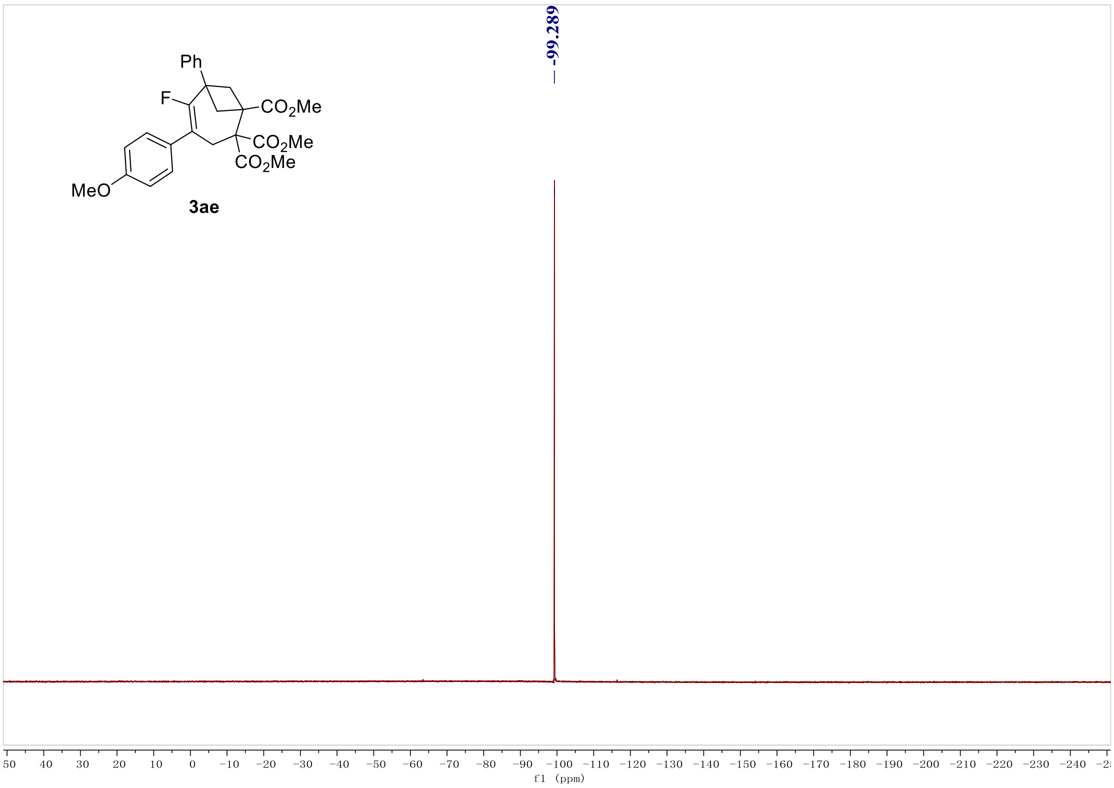

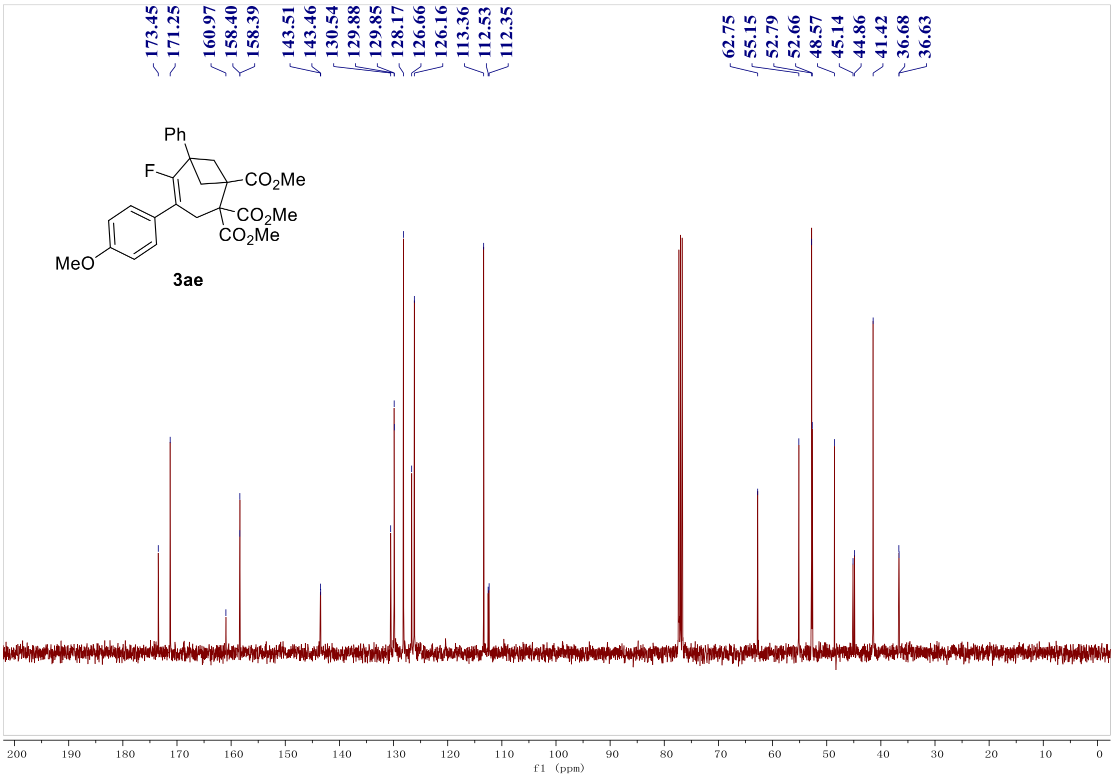

^{1}H NMR (400MHz, $CDCl_3$), ^{19}F NMR (376 MHz, $CDCl_3$) and ^{13}C NMR (100 MHz, $CDCl_3$) spectra of product **3af**

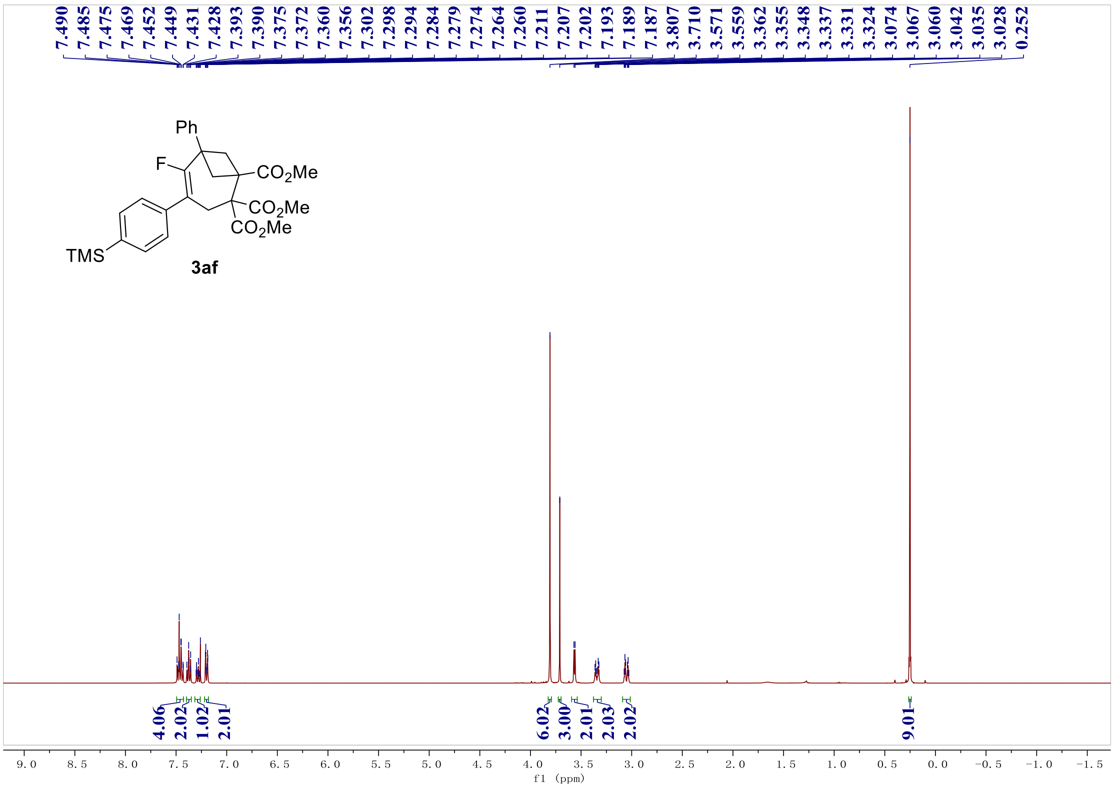

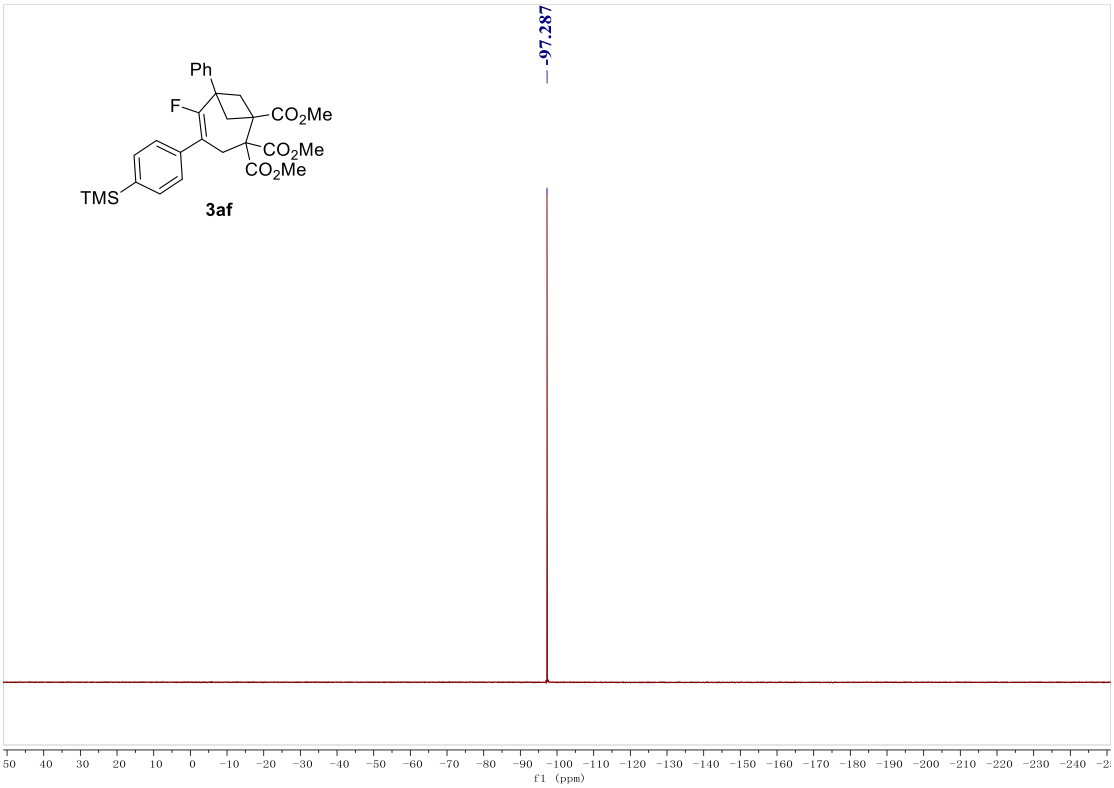

-97.287
Ph
F
CO2Me
CO2Me
CO2Me
TMS
3af
f1 (ppm)

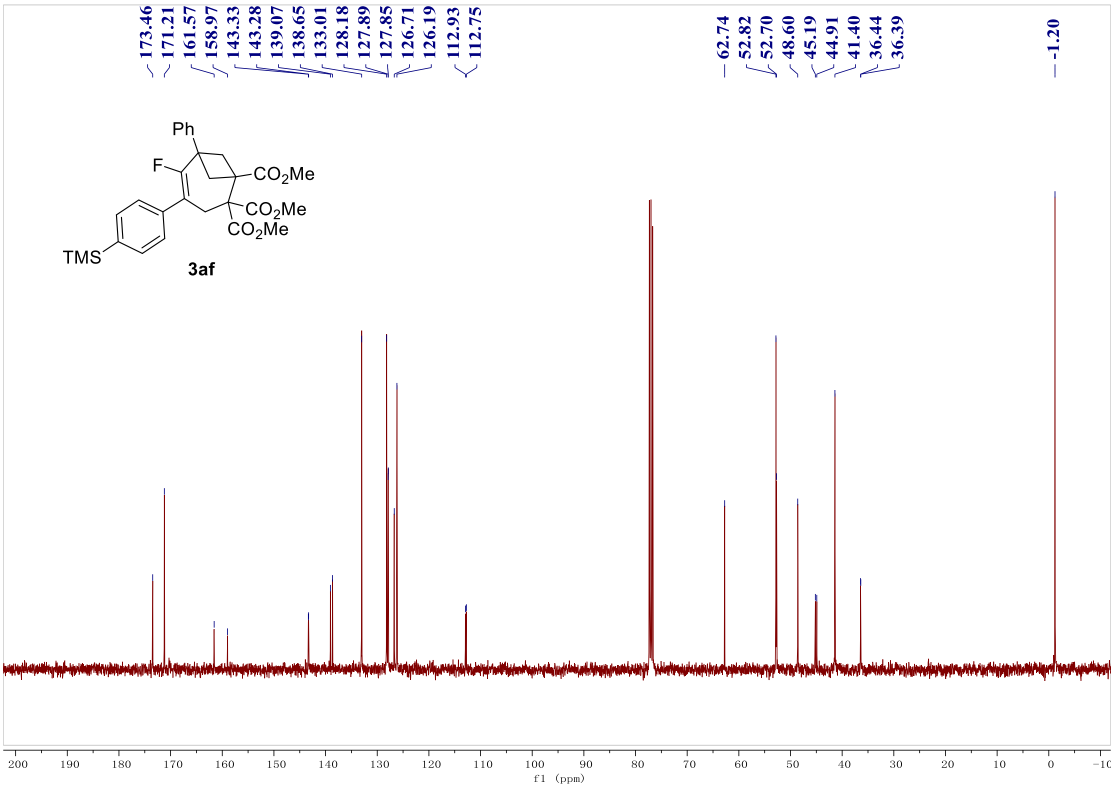

173.46
171.21
161.57
158.97
143.33
143.28
139.07
138.65
133.01
128.18
127.89
127.85
126.71
126.19
112.93
112.75
62.74
52.82
52.70
48.60
45.19
44.91
41.40
36.44
36.39
-1.20
Ph
F
CO2Me
CO2Me
CO2Me
TMS
3af
f1 (ppm)

^{1}H NMR (400MHz, $CDCl_3$), ^{19}F NMR (376 MHz, $CDCl_3$) and ^{13}C NMR (100 MHz, $CDCl_3$) spectra of product **3ag**

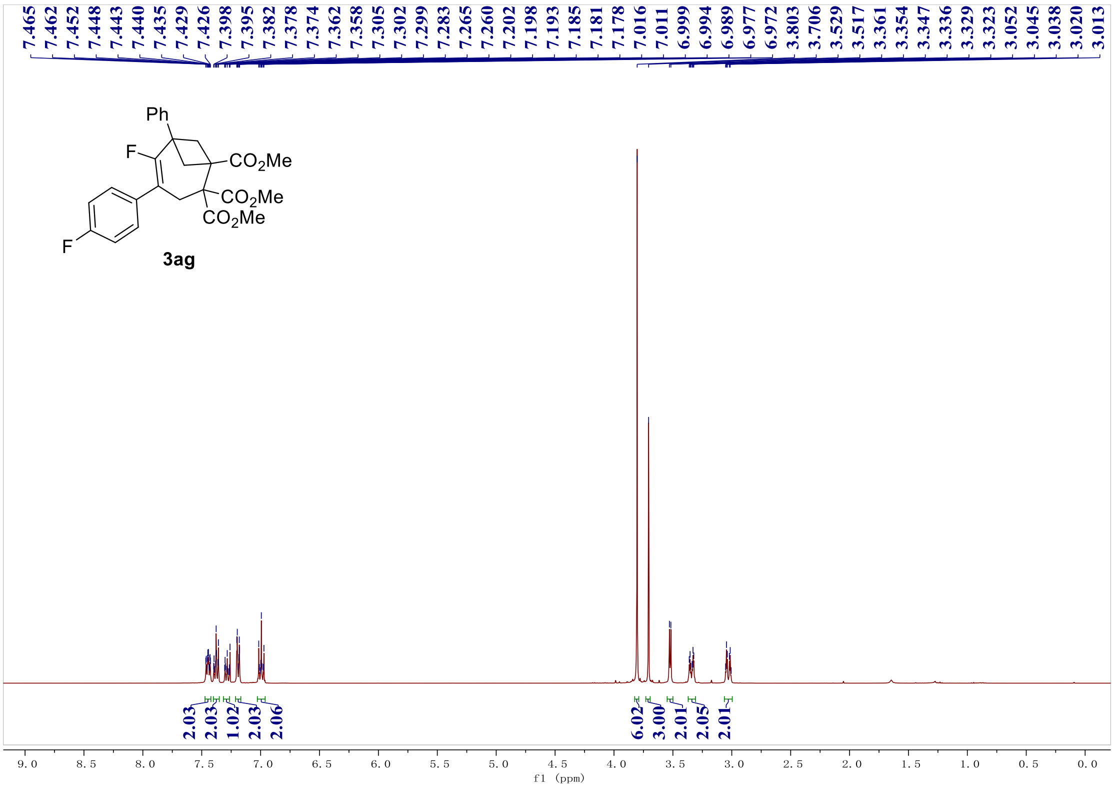

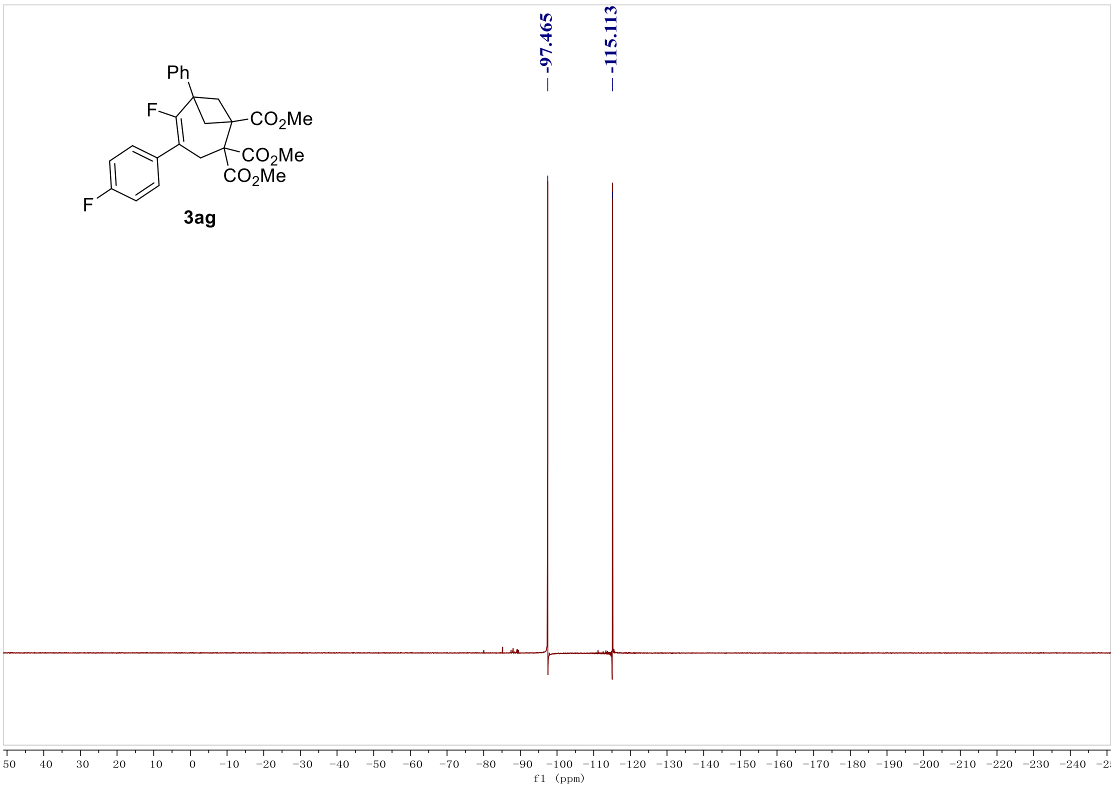

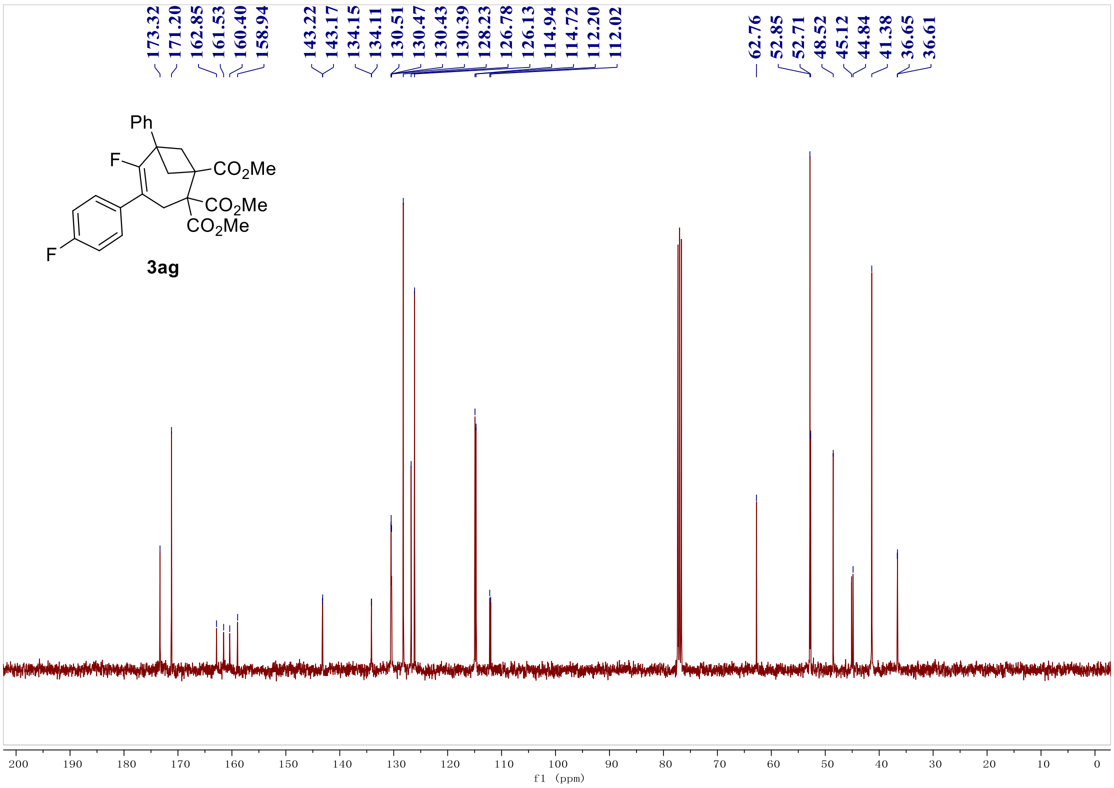

^{1}H NMR (400MHz, $CDCl_3$), ^{19}F NMR (376 MHz, $CDCl_3$) and ^{13}C NMR (100 MHz, $CDCl_3$) spectra of product **3ah**

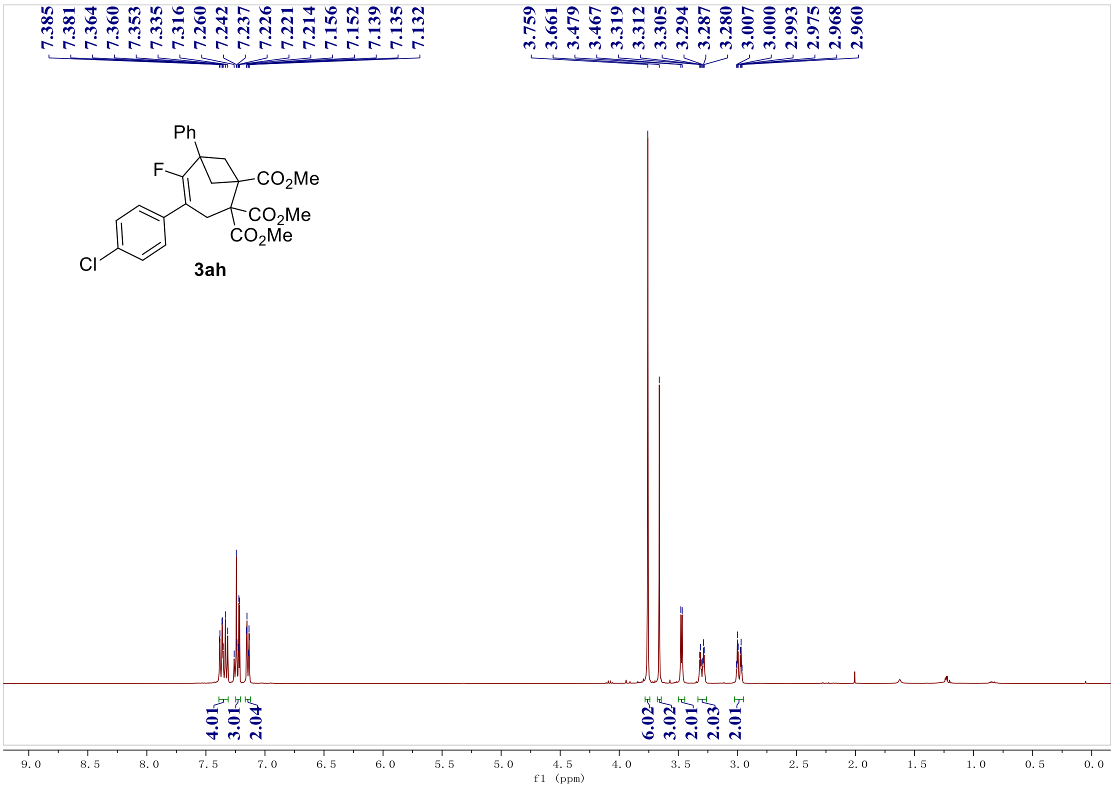

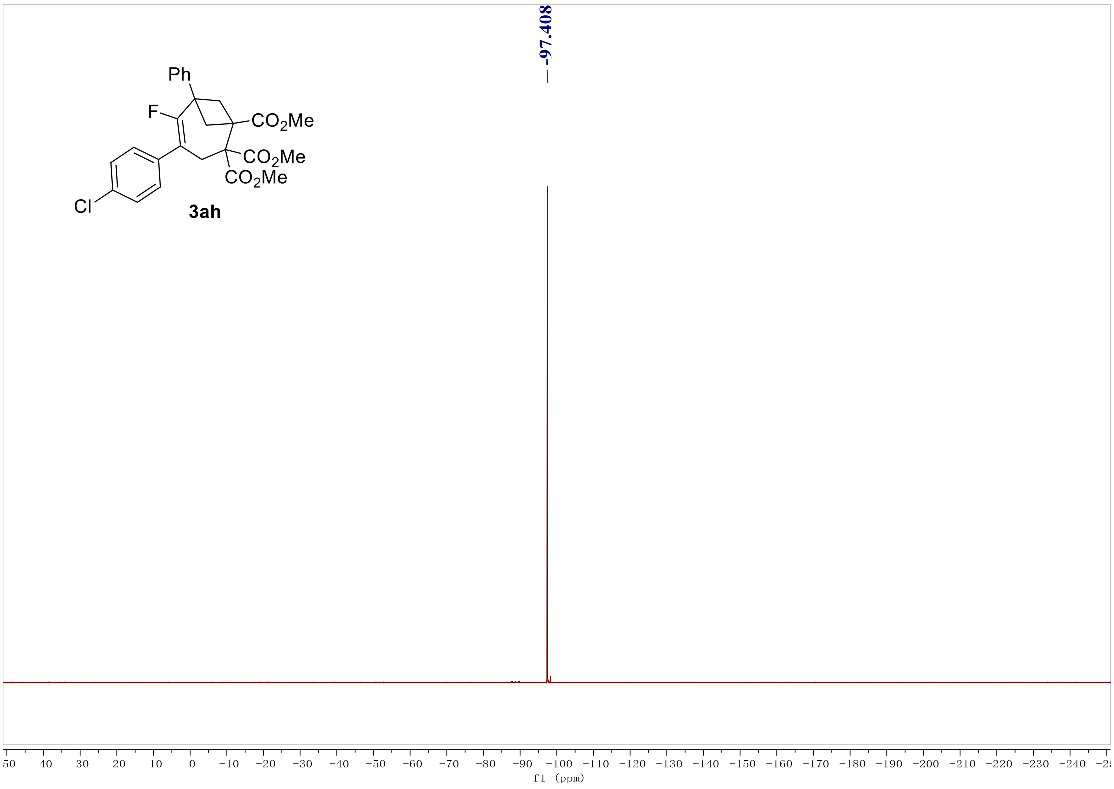
-97.408
Ph
F
CO_2Me
CO_2Me
CO_2Me
Cl
3ah
f1 (ppm)

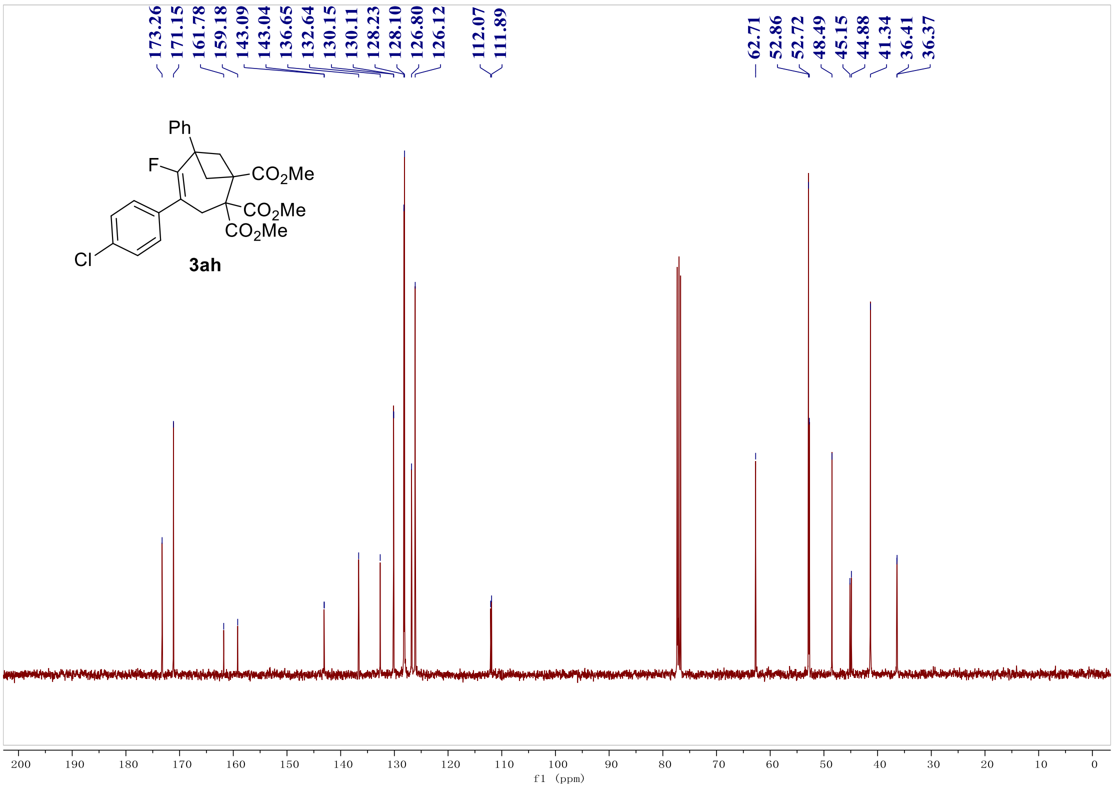
173.26
171.15
161.78
159.18
143.09
143.04
136.65
132.64
130.15
130.11
128.23
128.10
126.80
126.12
112.07
111.89
62.71
52.86
52.72
48.49
45.15
44.88
41.34
36.41
36.37
Ph
F
CO_2Me
CO_2Me
CO_2Me
Cl
3ah
f1 (ppm)

^{1}H NMR (400MHz, $CDCl_3$), ^{19}F NMR (376 MHz, $CDCl_3$) and ^{13}C NMR (100 MHz, $CDCl_3$) spectra of product **3ai**

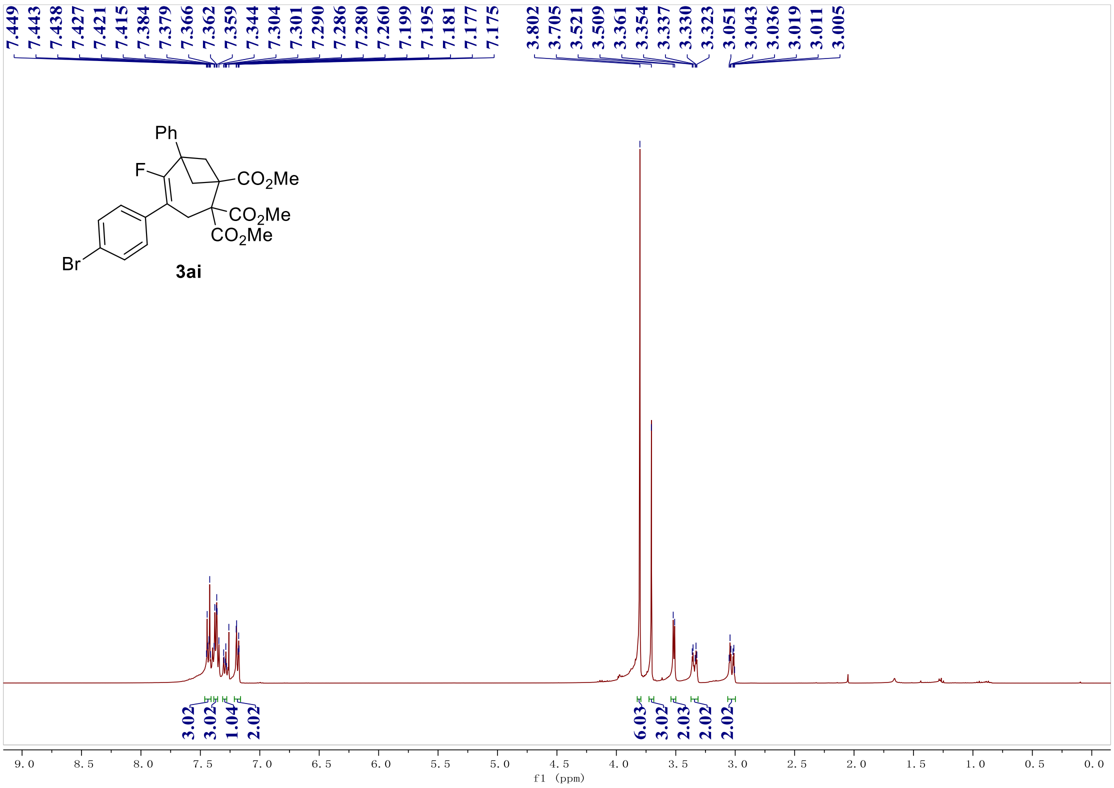

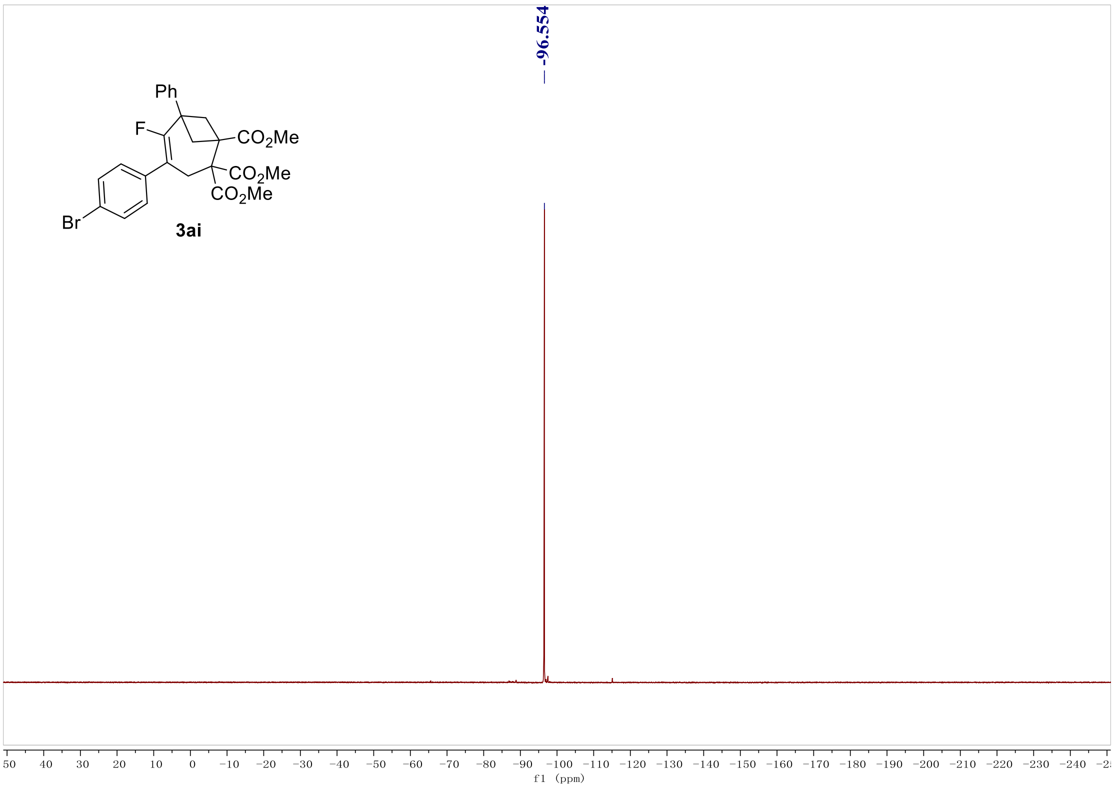

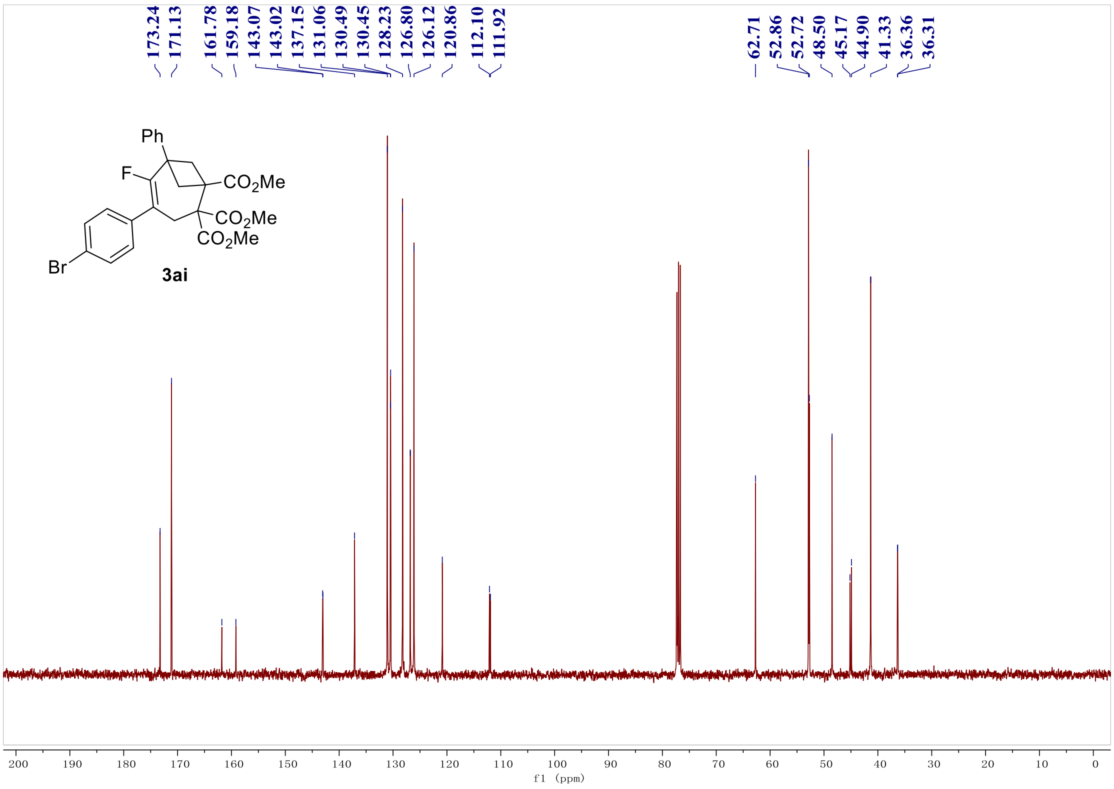

^{1}H NMR (400MHz, $CDCl_3$), ^{19}F NMR (376 MHz, $CDCl_3$) and ^{13}C NMR (100 MHz, $CDCl_3$) spectra of product **3aj**

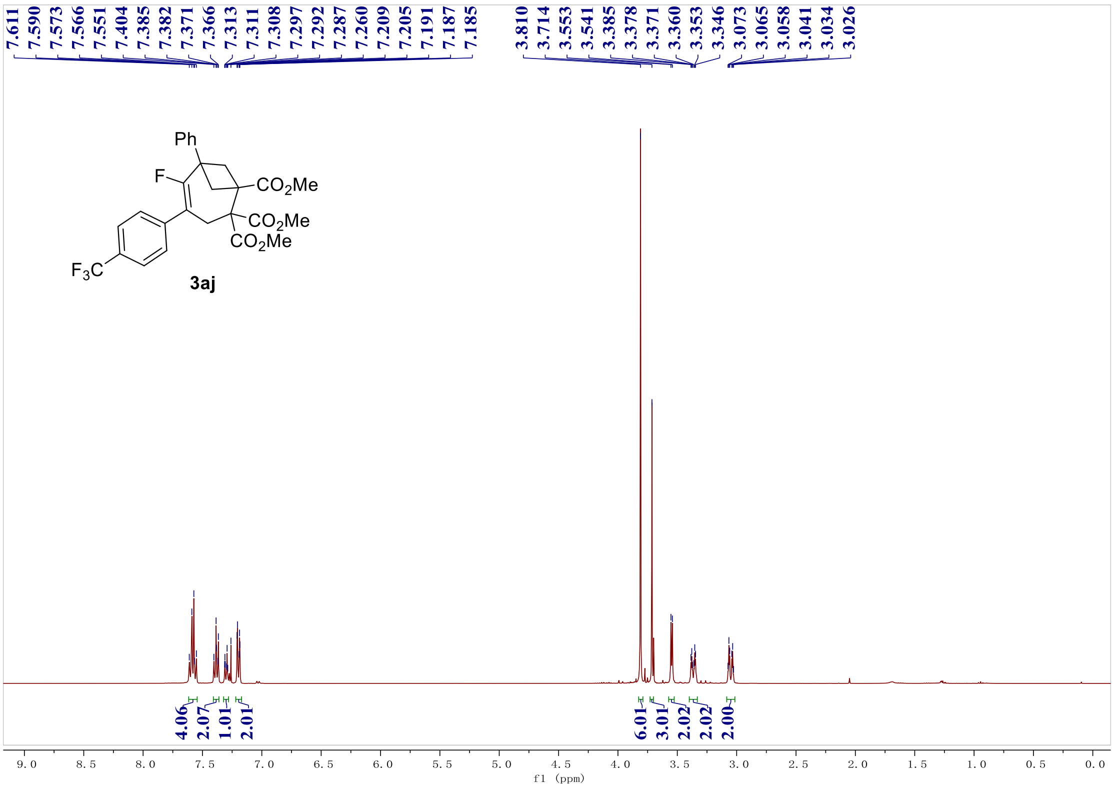

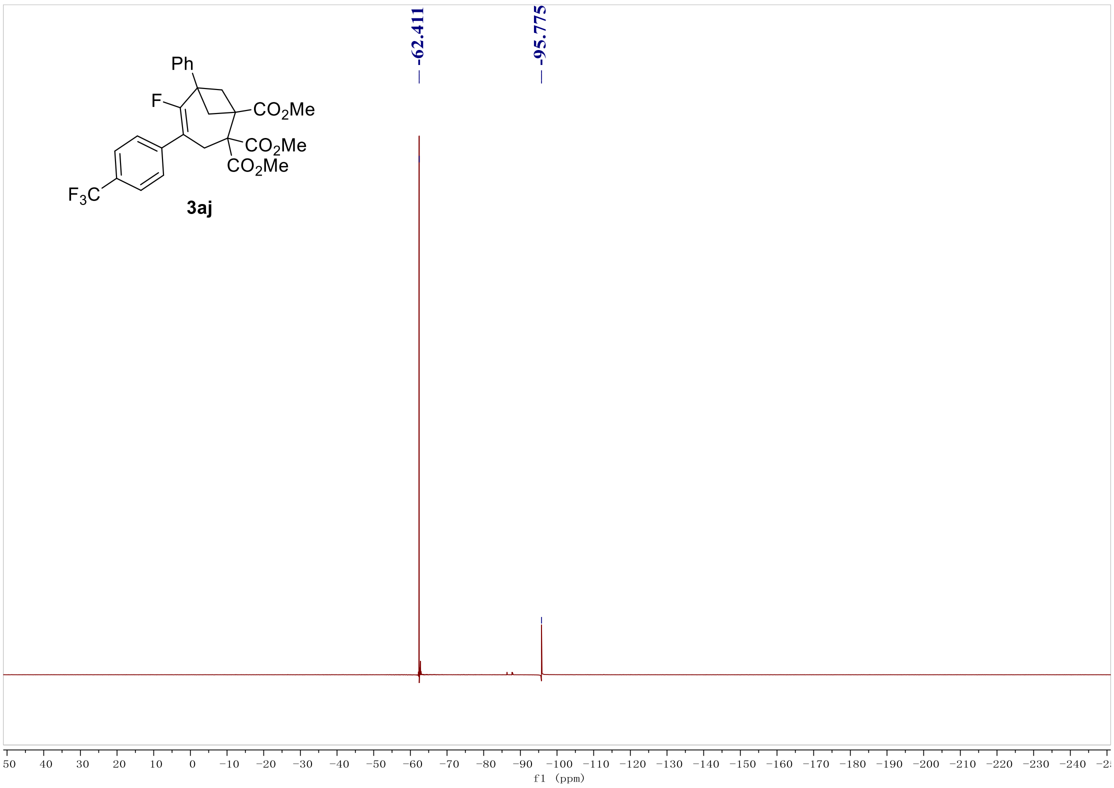
-62.411
-95.775
Ph
F
CO2Me
CO2Me
CO2Me
F3C
3aj
50 40 30 20 10 0 -10 -20 -30 -40 -50 -60 -70 -80 -90 -100 -110 -120 -130 -140 -150 -160 -170 -180 -190 -200 -210 -220 -230 -240
f1 (ppm)

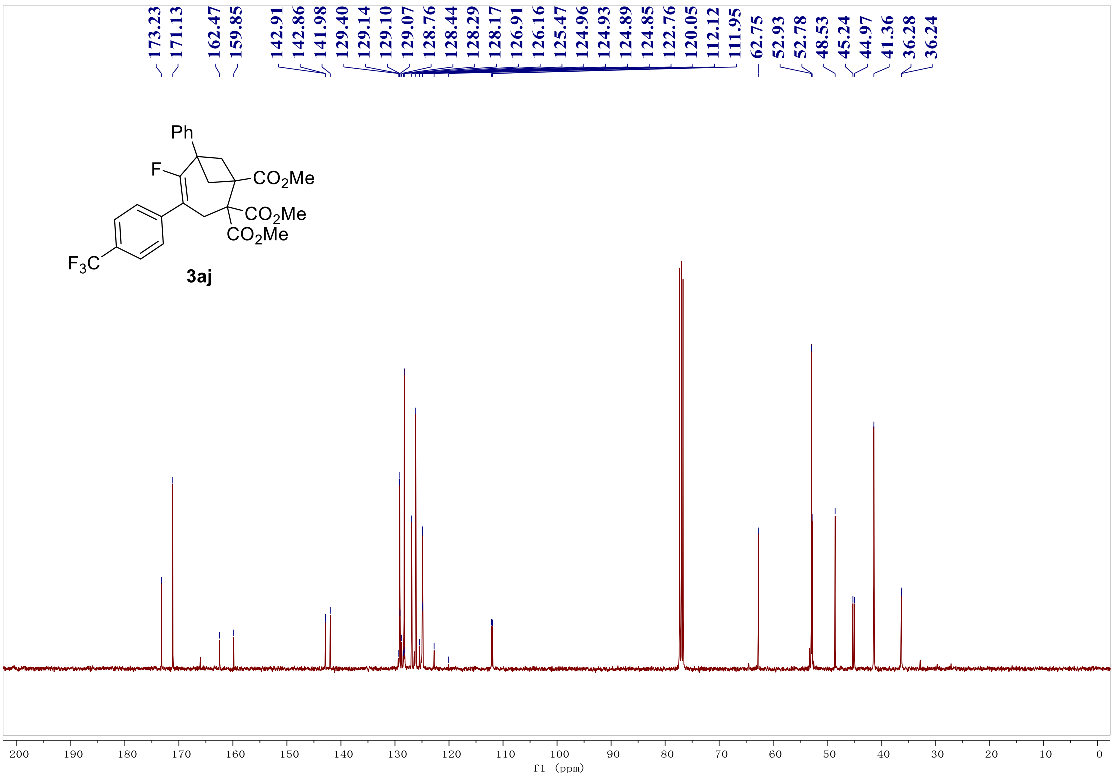
173.23
171.13
162.47
159.85
142.91
142.86
141.98
129.40
129.14
129.10
129.07
128.76
128.44
128.29
128.17
126.91
126.16
125.47
124.96
124.93
124.89
124.85
122.76
120.05
112.12
111.95
62.75
52.93
52.78
48.53
45.24
44.97
41.36
36.28
36.24
Ph
F
CO2Me
CO2Me
CO2Me
F3C
3aj
200 190 180 170 160 150 140 130 120 110 100 90 80 70 60 50 40 30 20 10 0
f1 (ppm)

^{1}H NMR (400MHz, $CDCl_3$), ^{19}F NMR (376 MHz, $CDCl_3$) and ^{13}C NMR (100 MHz, $CDCl_3$) spectra of product **3ak**

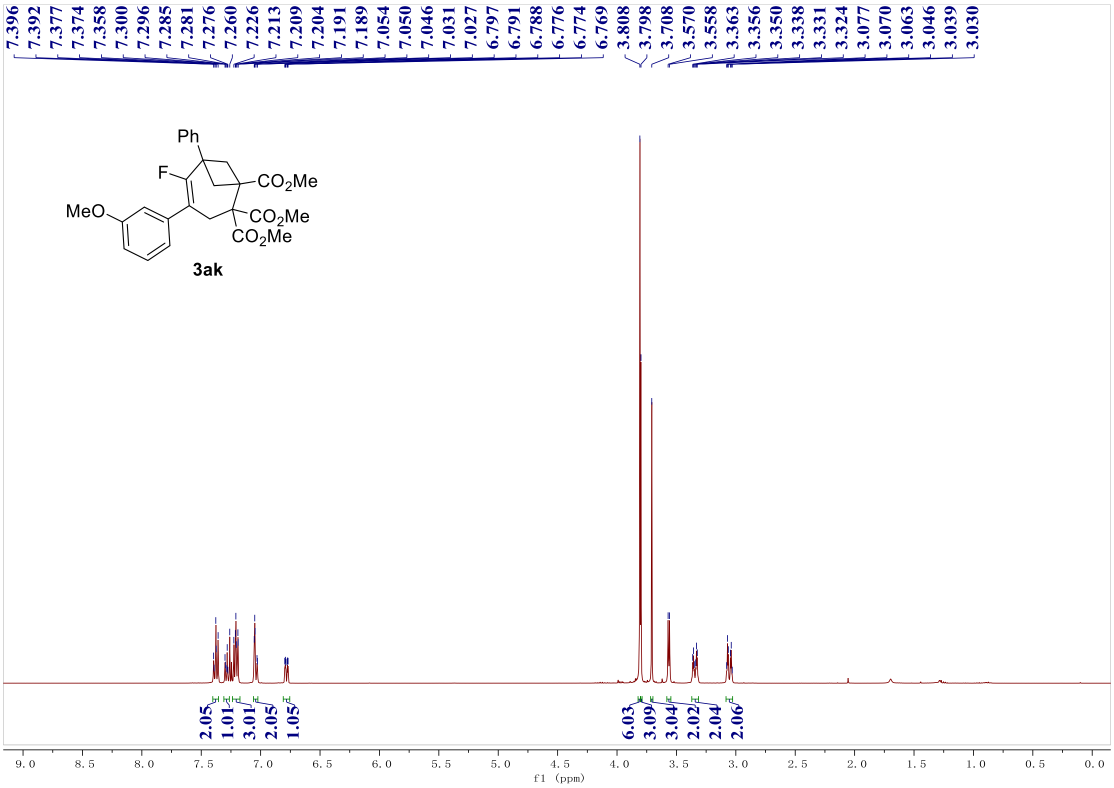

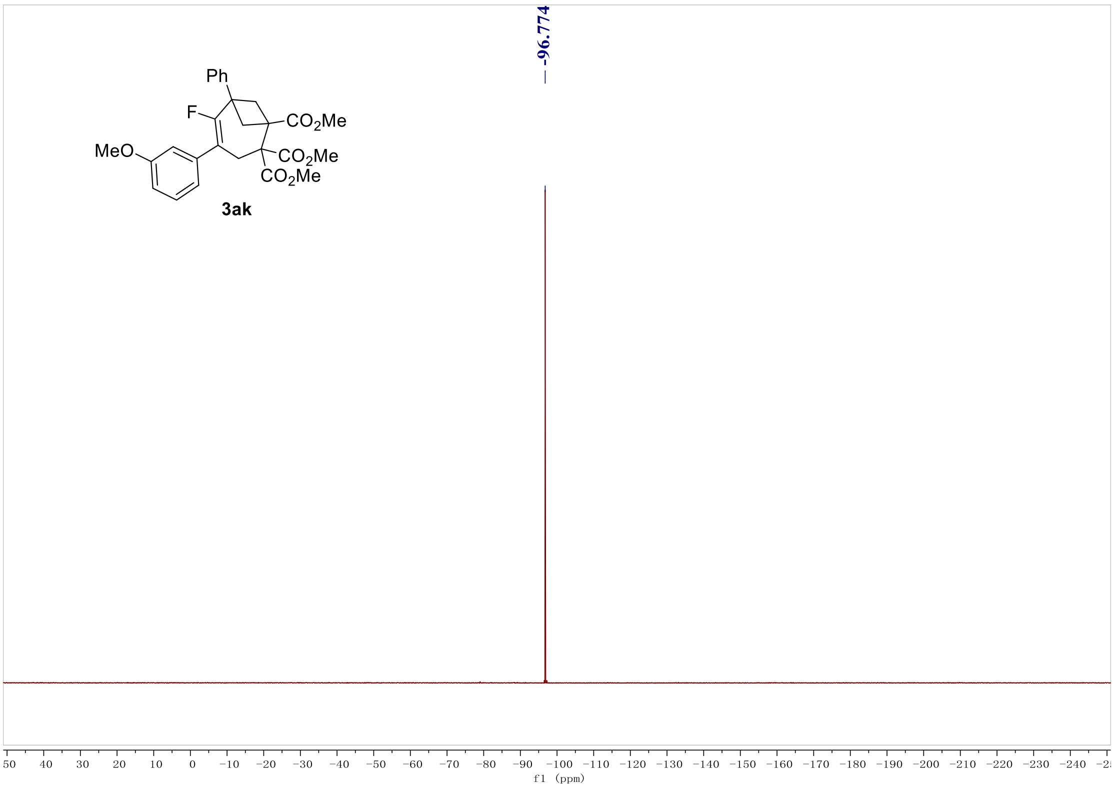

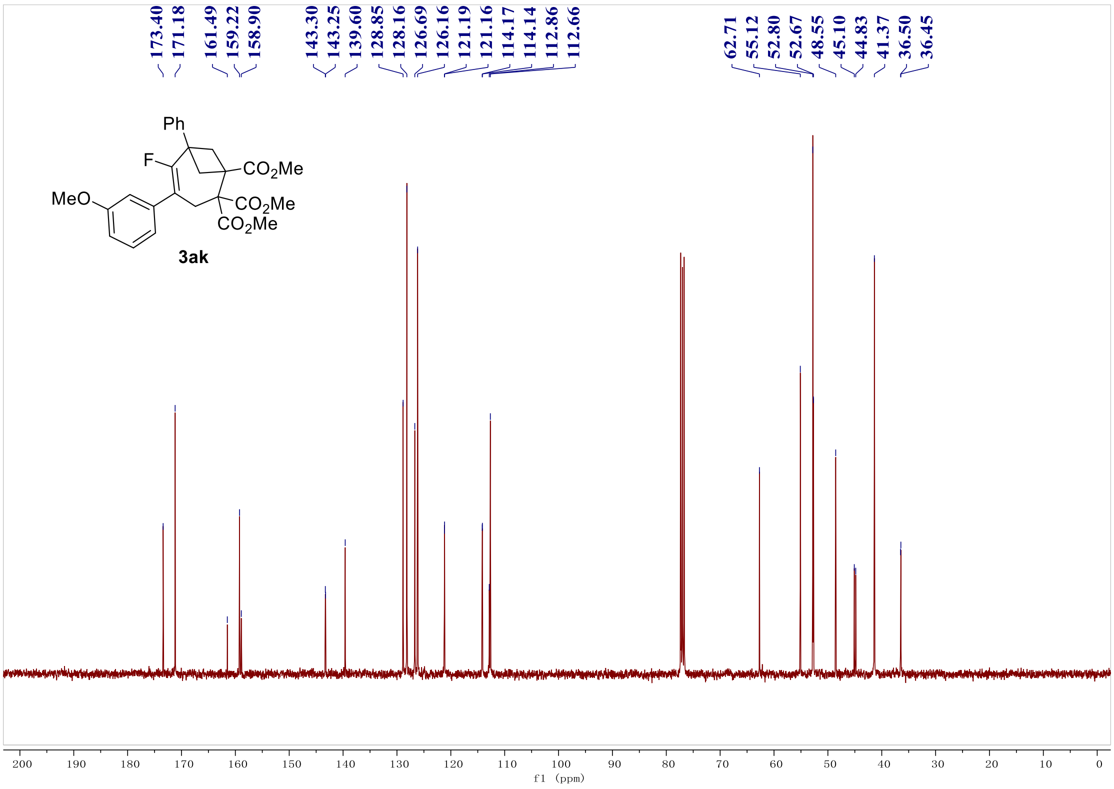

^{1}H NMR (400MHz, $CDCl_3$), ^{19}F NMR (376 MHz, $CDCl_3$) and ^{13}C NMR (100 MHz, $CDCl_3$) spectra of product **3al**

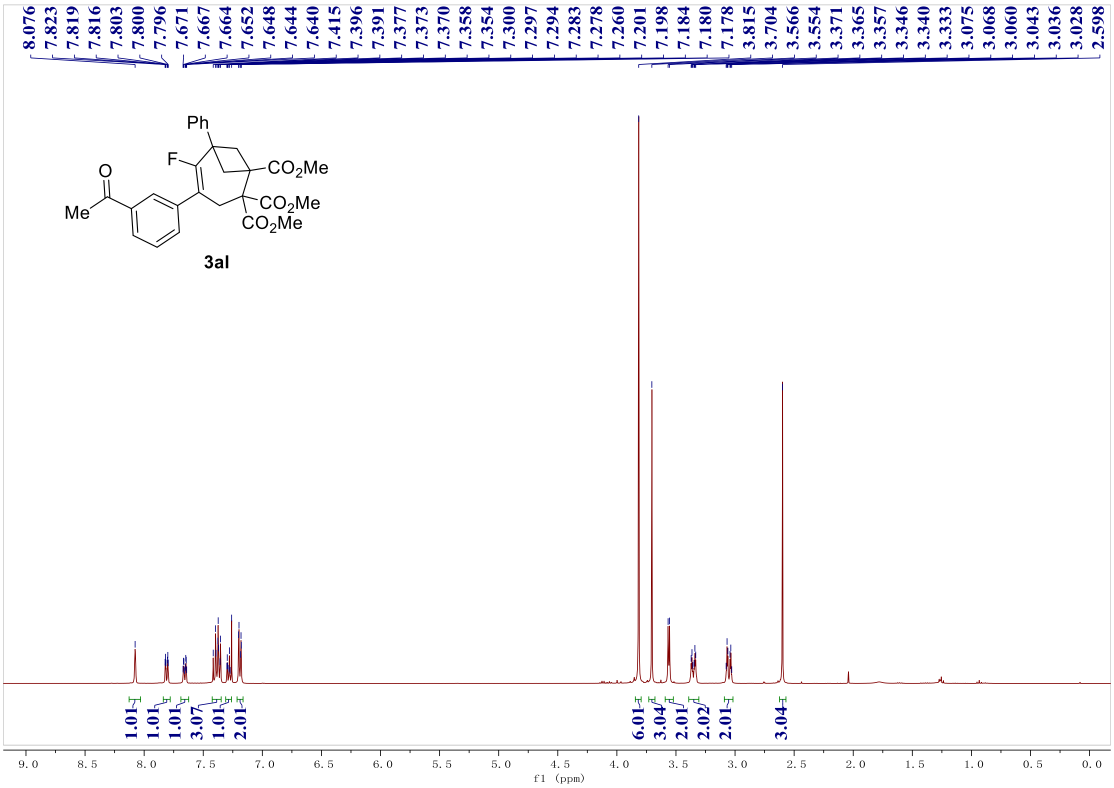

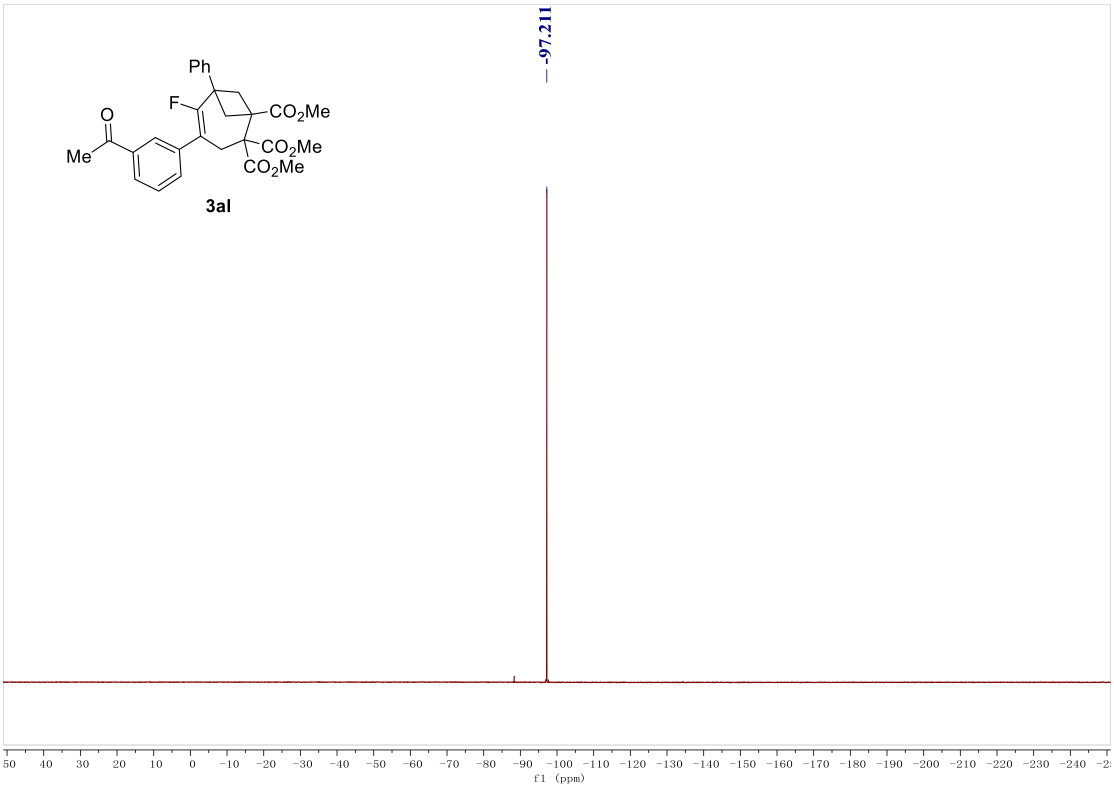
-97.211
Ph
F
O
Me
CO2Me
CO2Me
CO2Me
3al
50 40 30 20 10 0 -10 -20 -30 -40 -50 -60 -70 -80 -90 -100 -110 -120 -130 -140 -150 -160 -170 -180 -190 -200 -210 -220 -230 -240
f1 (ppm)

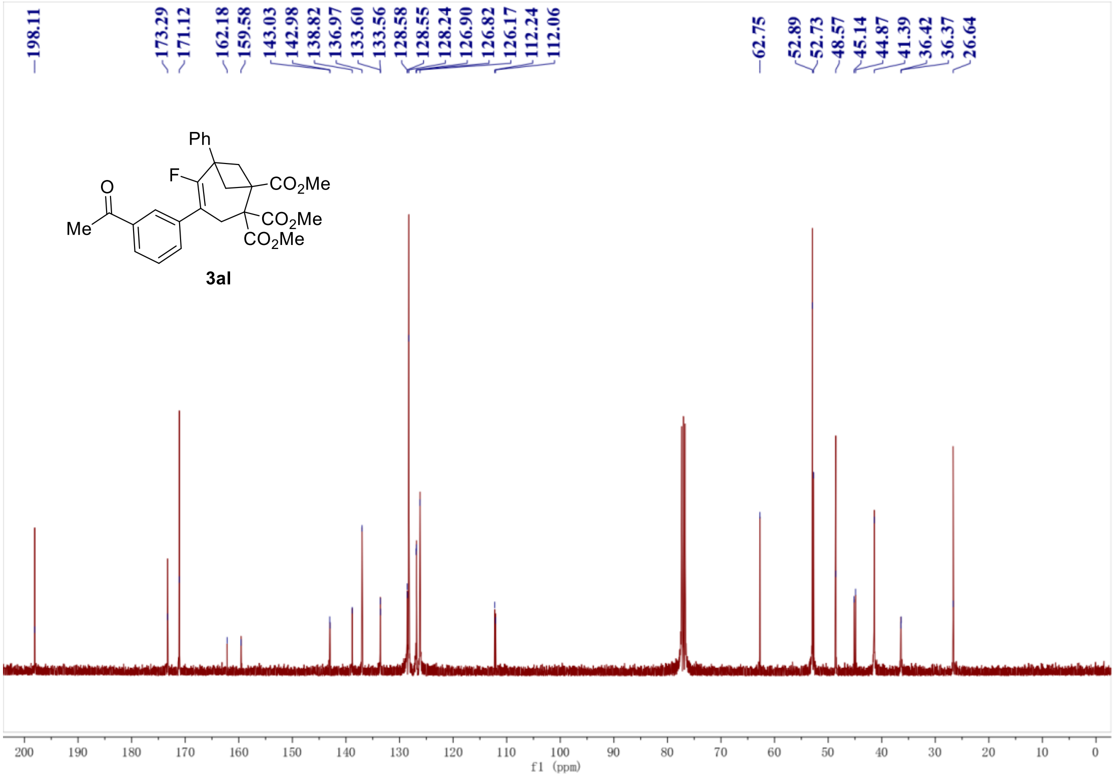
198.11
173.29
171.12
162.18
159.58
143.03
142.98
138.82
136.97
133.60
133.56
128.58
128.55
128.24
126.90
126.82
126.17
112.24
112.06
62.75
52.89
52.73
48.57
45.14
44.87
41.39
36.42
36.37
26.64
Ph
F
O
Me
CO2Me
CO2Me
CO2Me
3al
200 190 180 170 160 150 140 130 120 110 100 90 80 70 60 50 40 30 20 10 0
f1 (ppm)

^{1}H NMR (400MHz, $CDCl_3$), ^{19}F NMR (376 MHz, $CDCl_3$) and ^{13}C NMR (100 MHz, $CDCl_3$) spectra of product **3am**

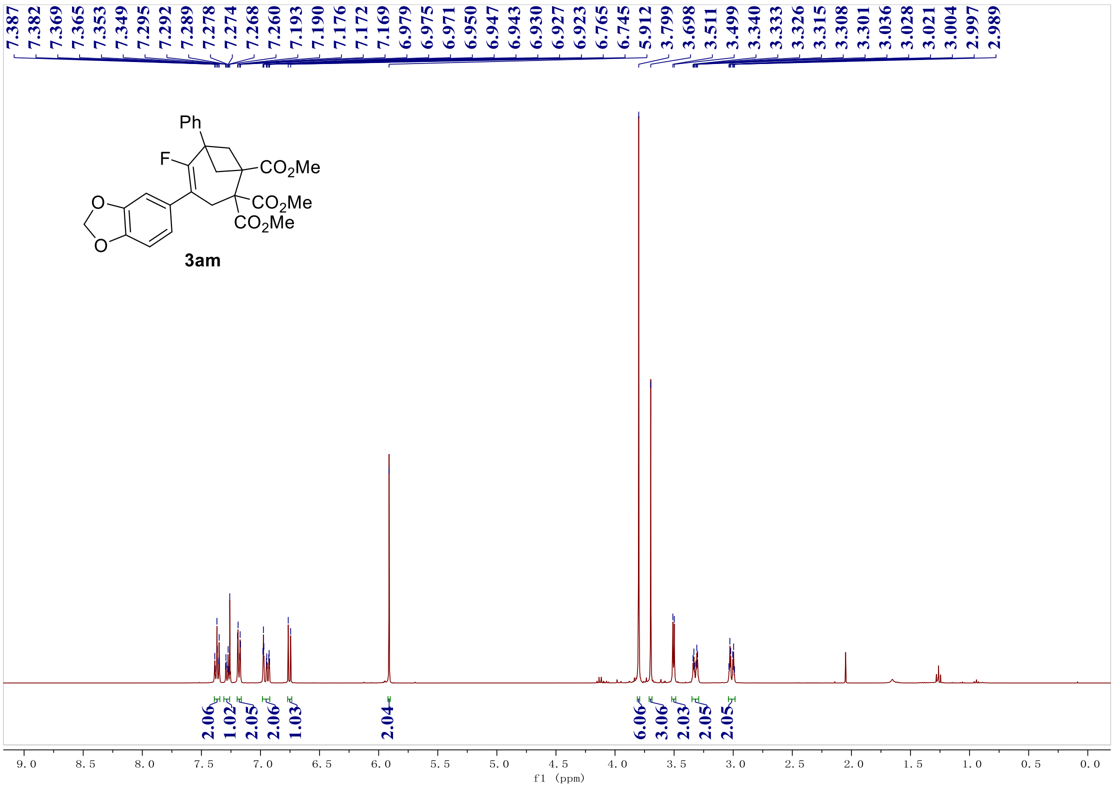

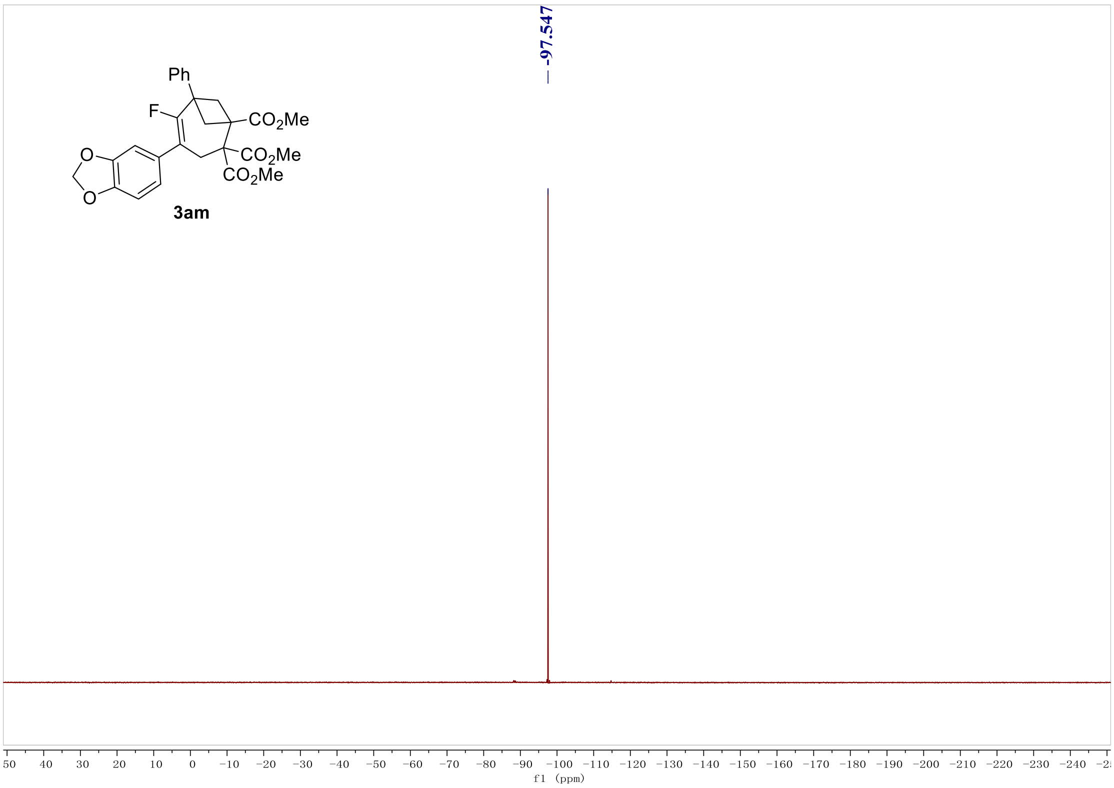

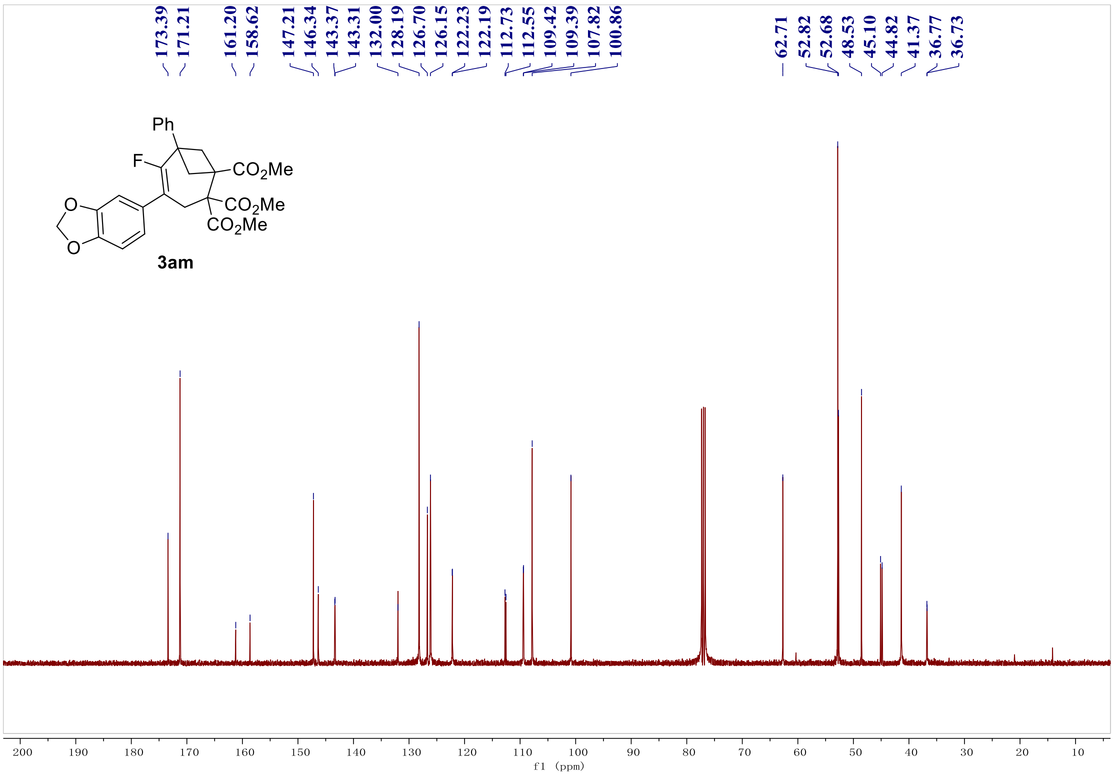

^{1}H NMR (400MHz, $CDCl_3$), ^{19}F NMR (376 MHz, $CDCl_3$) and ^{13}C NMR (100 MHz, $CDCl_3$) spectra of product **3an**

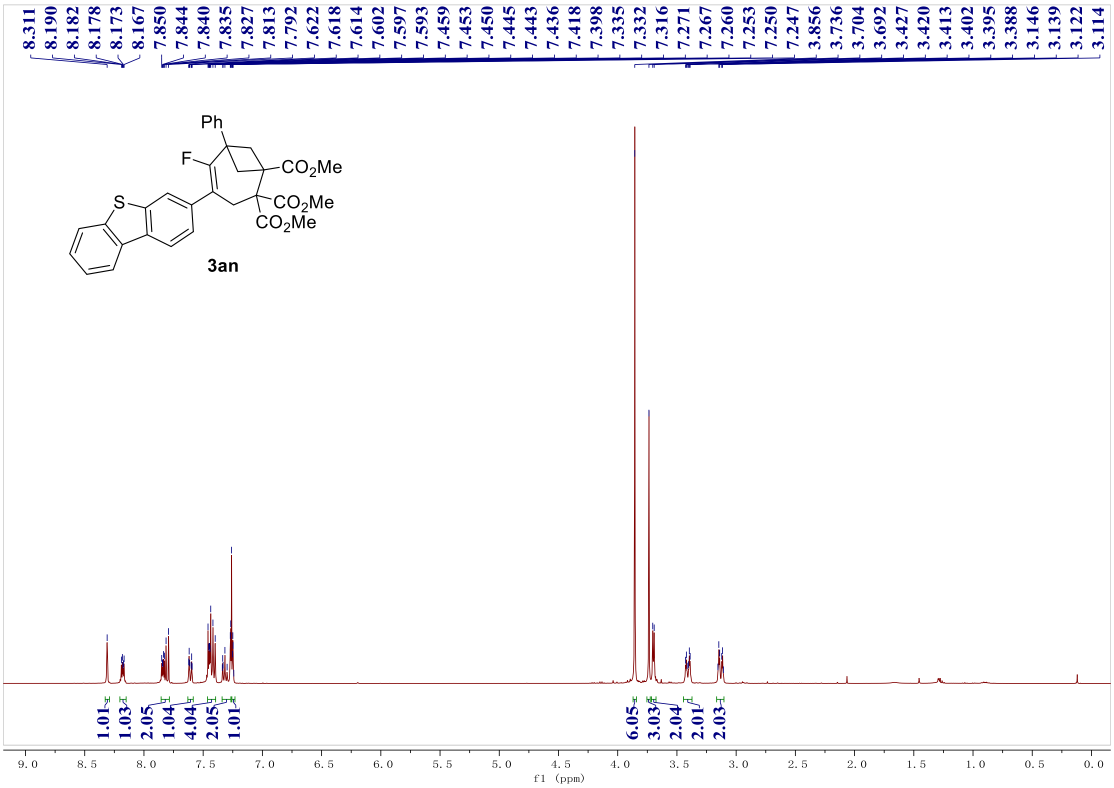

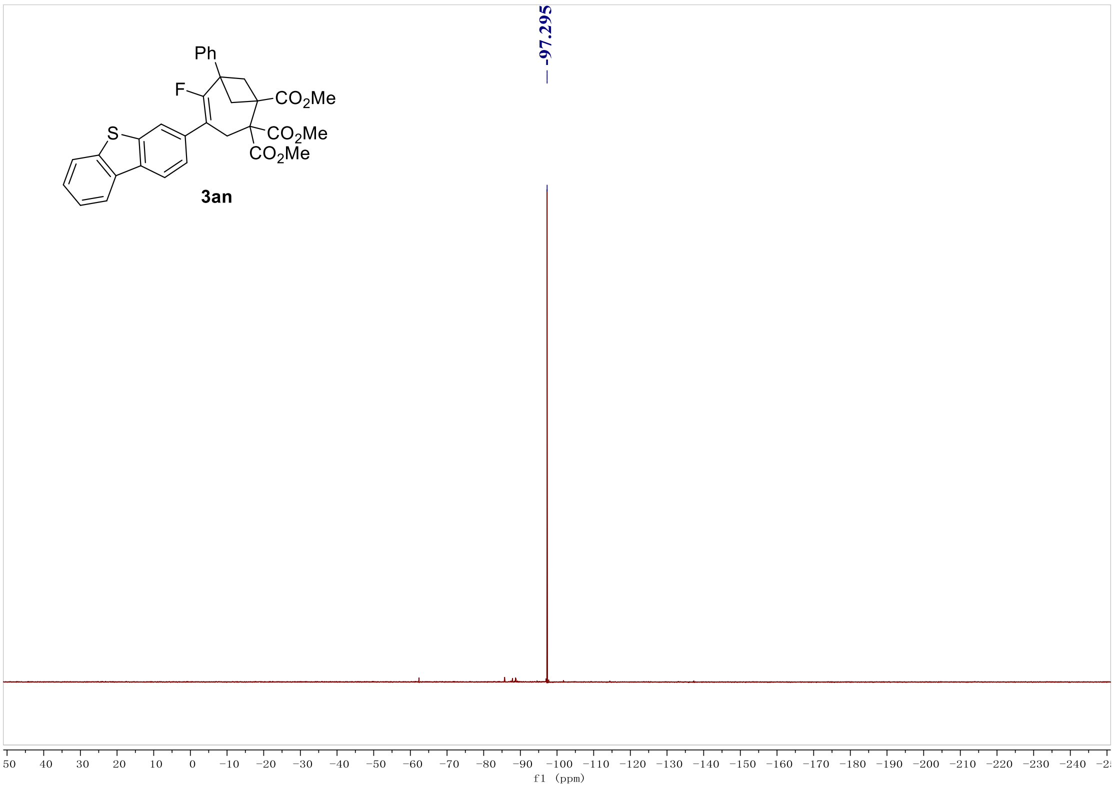
-97.295
Ph
F
CO2Me
CO2Me
CO2Me
S
3an
f1 (ppm)

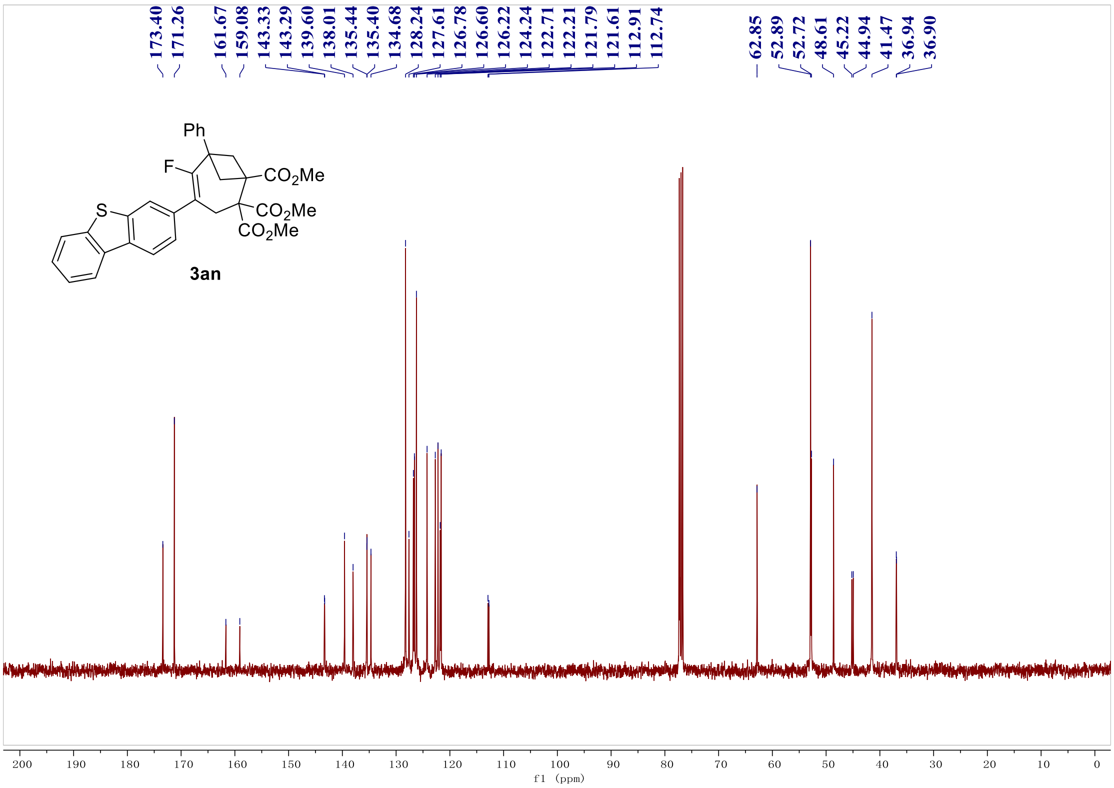
173.40
171.26
161.67
159.08
143.33
143.29
139.60
138.01
135.44
135.40
134.68
128.24
127.61
126.78
126.60
126.22
124.24
122.71
122.21
121.79
121.61
112.91
112.74
62.85
52.89
52.72
48.61
45.22
44.94
41.47
36.94
36.90
Ph
F
CO2Me
CO2Me
CO2Me
S
3an
f1 (ppm)

^1H NMR (400MHz, $CDCl_3$), ^{19}F NMR (376 MHz, $CDCl_3$) and ^{13}C NMR (100 MHz, $CDCl_3$) spectra of product **3ao**

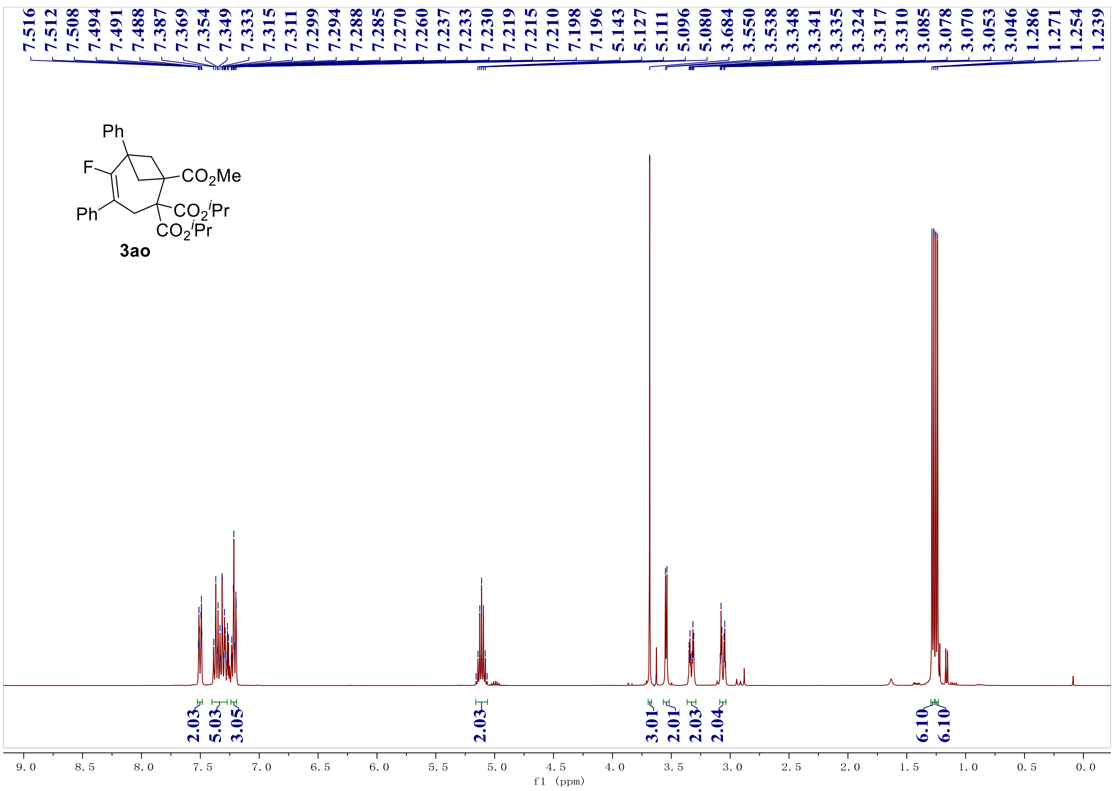

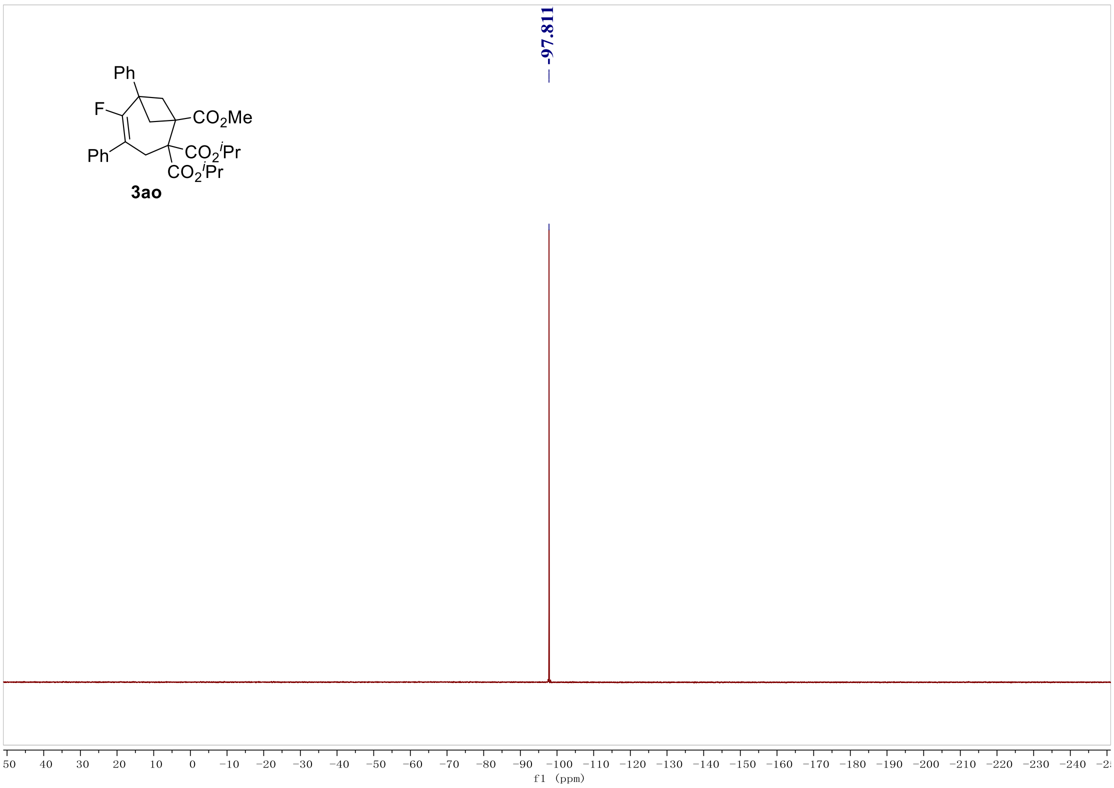

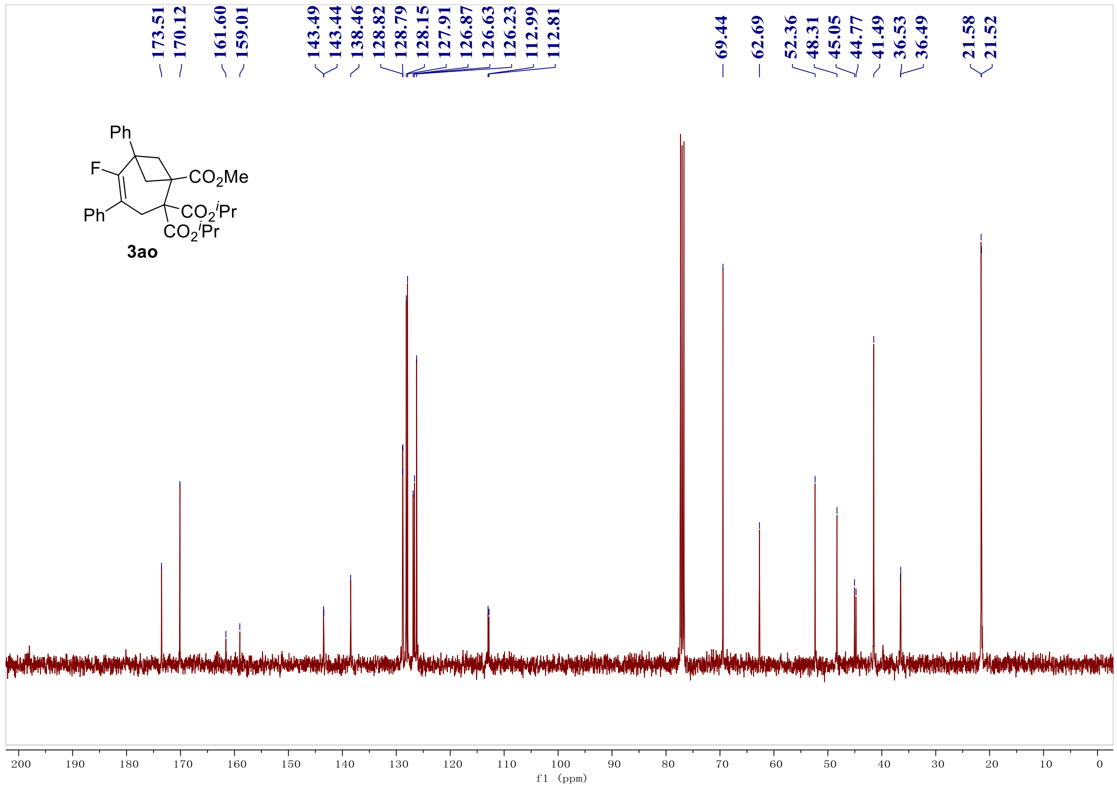

^{1}H NMR (400MHz, $CDCl_3$), ^{19}F NMR (376 MHz, $CDCl_3$) and ^{13}C NMR (100 MHz, $CDCl_3$) spectra of product **3ap**

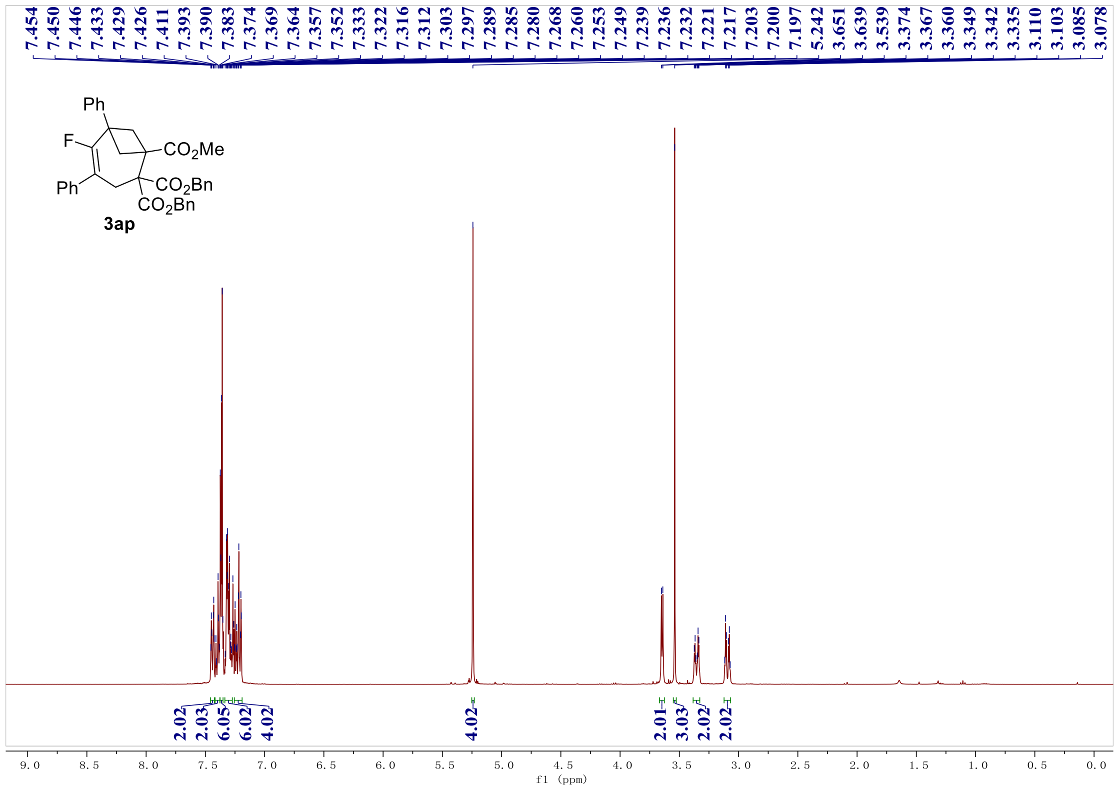

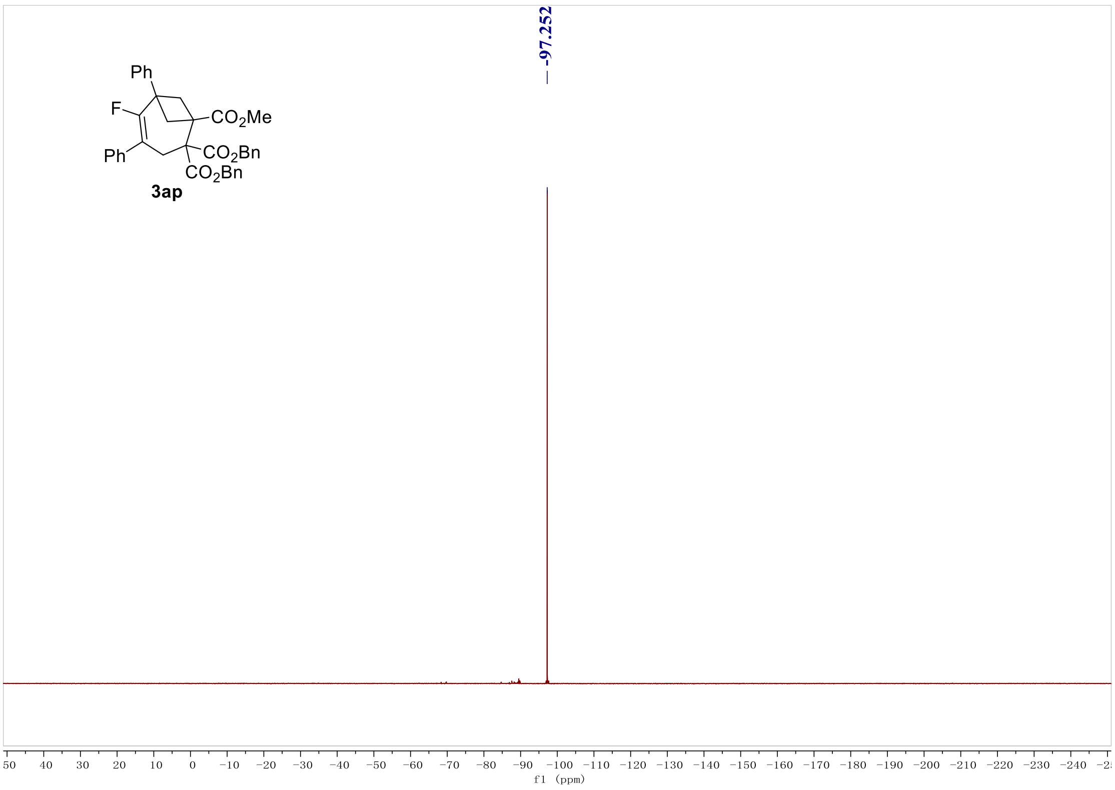
-97.252
Ph
F
CO2Me
Ph
CO2Bn
CO2Bn
3ap
f1 (ppm)

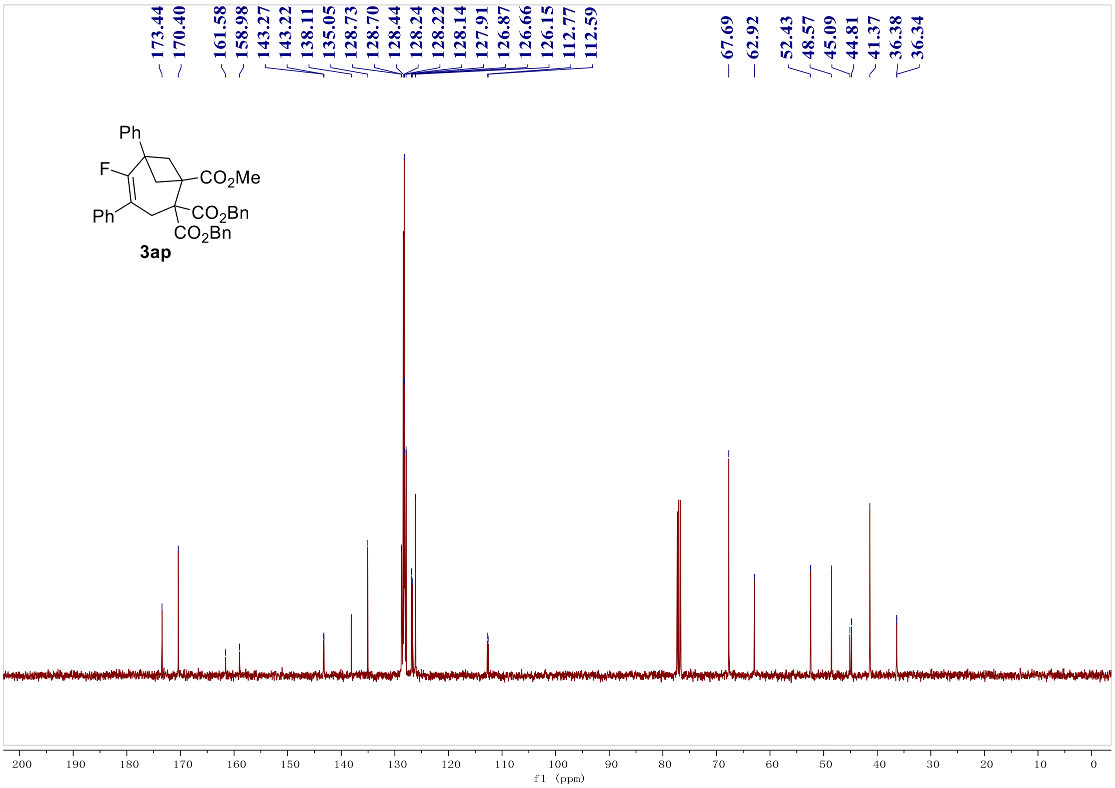
173.44
170.40
161.58
158.98
143.27
143.22
138.11
135.05
128.73
128.70
128.44
128.24
128.22
128.14
127.91
126.87
126.66
126.15
112.77
112.59
67.69
62.92
52.43
48.57
45.09
44.81
41.37
36.38
36.34
Ph
F
CO2Me
Ph
CO2Bn
CO2Bn
3ap
f1 (ppm)

^{1}H NMR (400MHz, $CDCl_3$), ^{19}F NMR (376 MHz, $CDCl_3$) and ^{13}C NMR (100 MHz, $CDCl_3$) spectra of product **3aq**

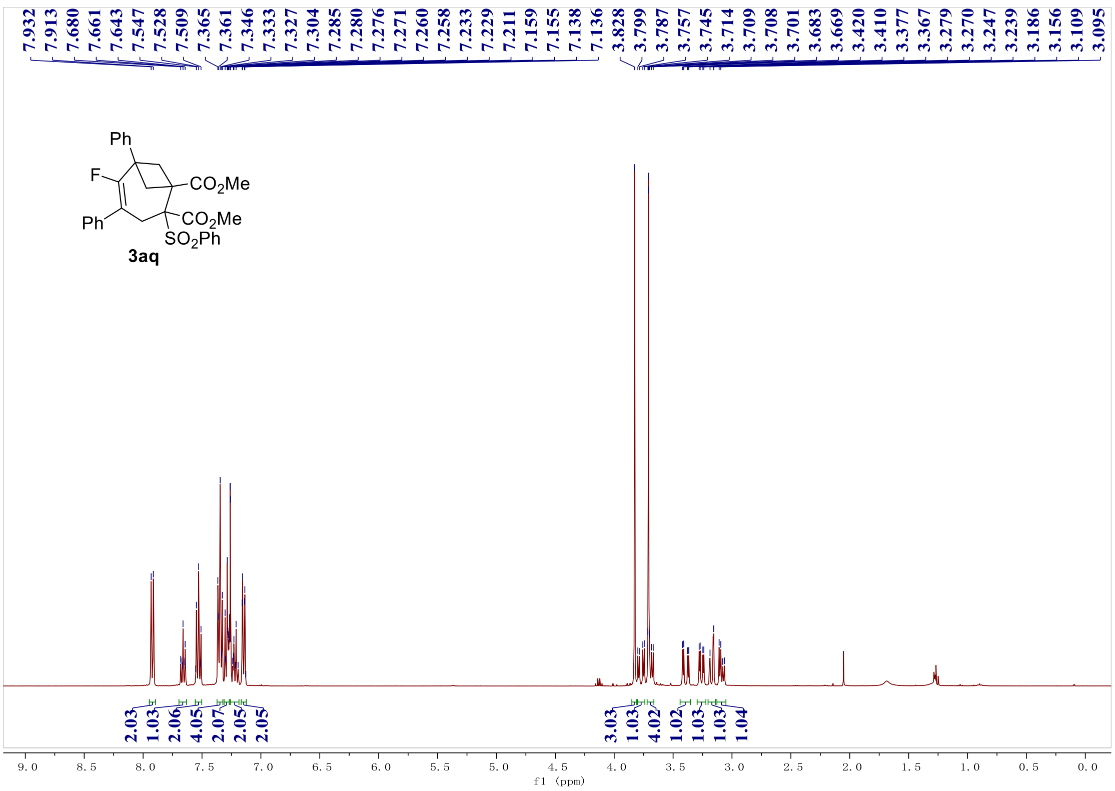

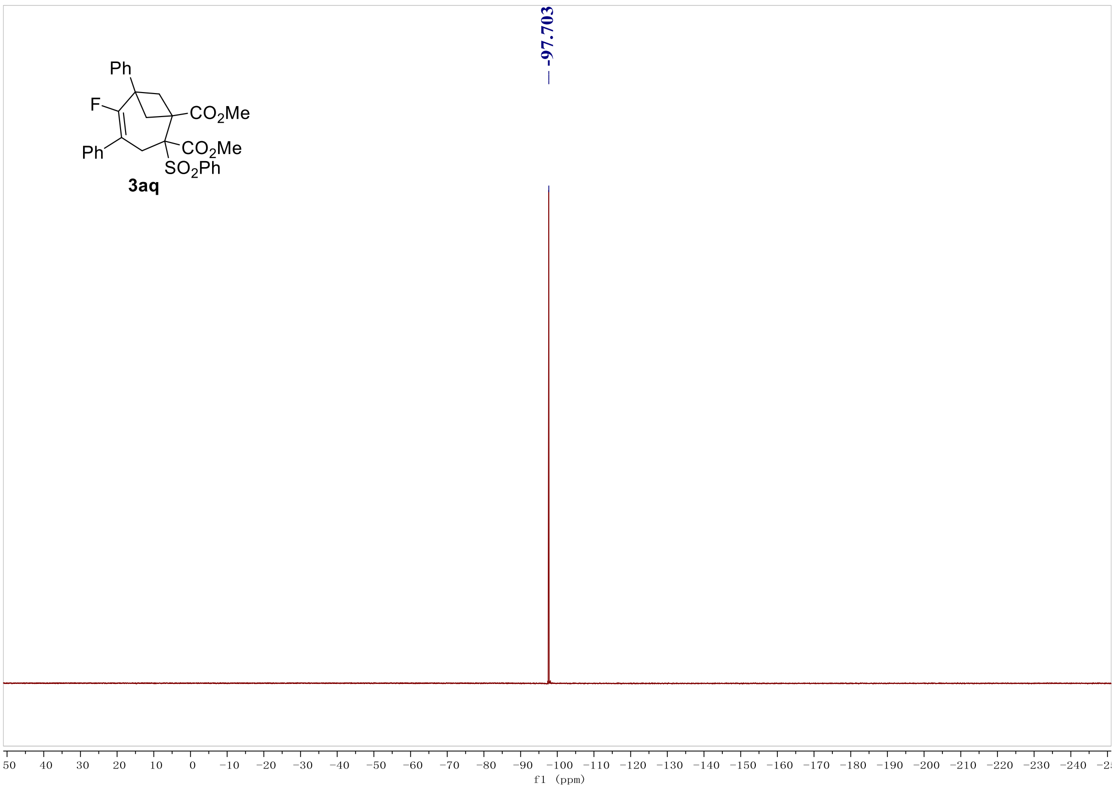

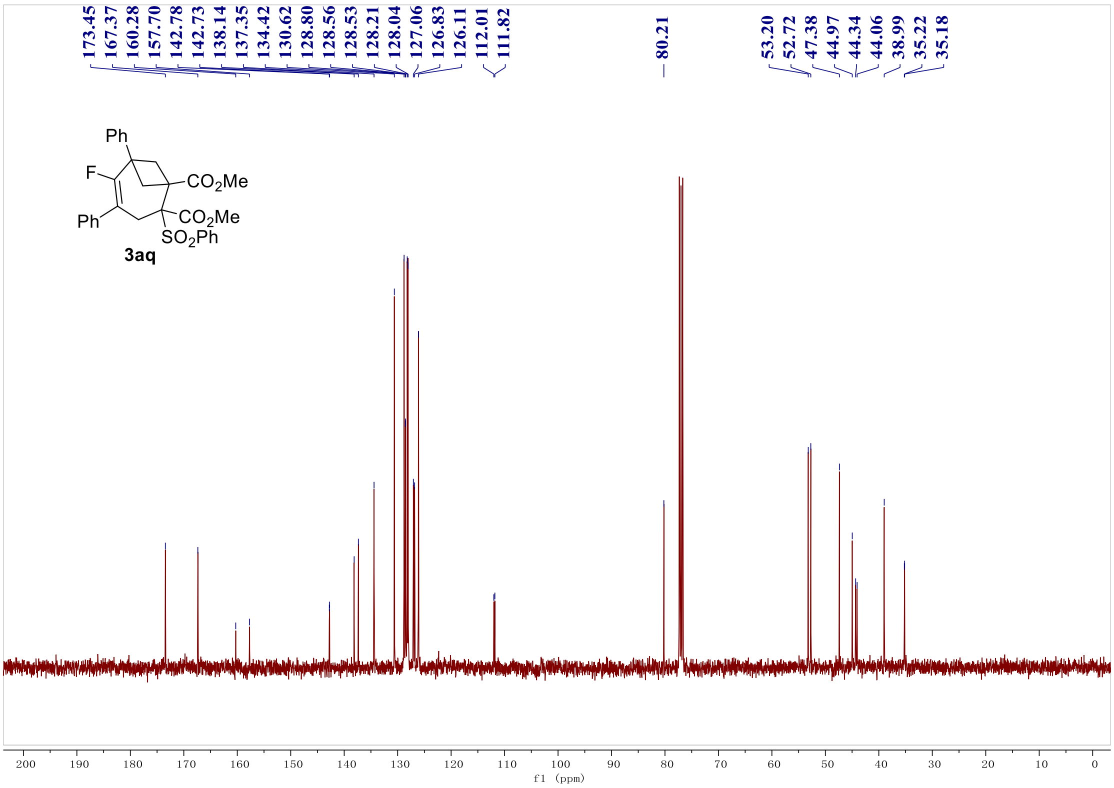

^{1}H NMR (400MHz, $CDCl_3$), ^{19}F NMR (376 MHz, $CDCl_3$) and ^{13}C NMR (100 MHz, $CDCl_3$) spectra of product **3ar**

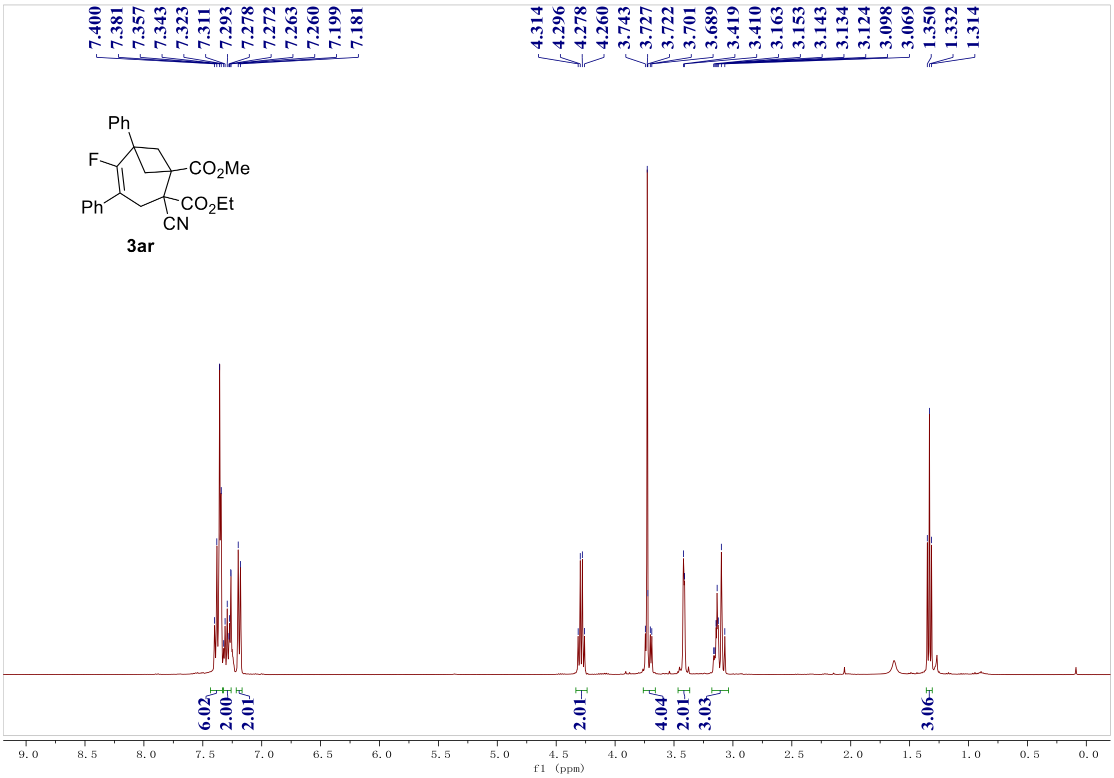

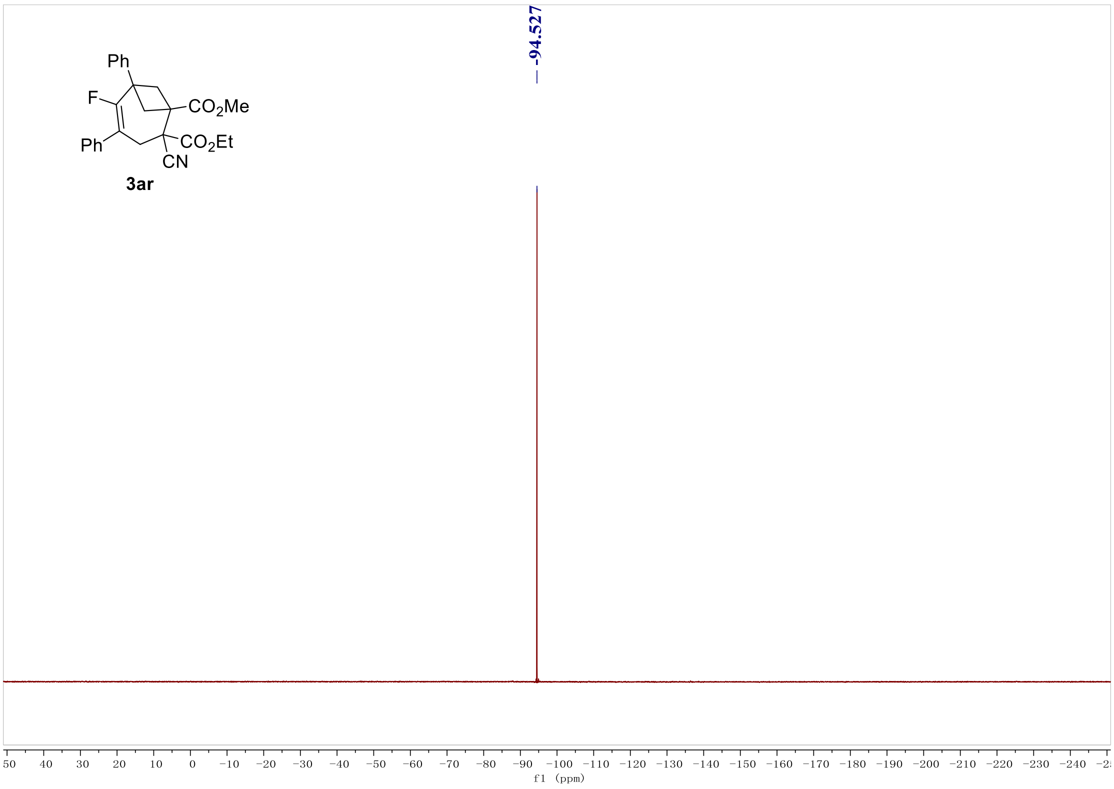
-94.527
Ph
F
CO2Me
Ph
CO2Et
CN
3ar
50 40 30 20 10 0 -10 -20 -30 -40 -50 -60 -70 -80 -90 -100 -110 -120 -130 -140 -150 -160 -170 -180 -190 -200 -210 -220 -230 -240
f1 (ppm)

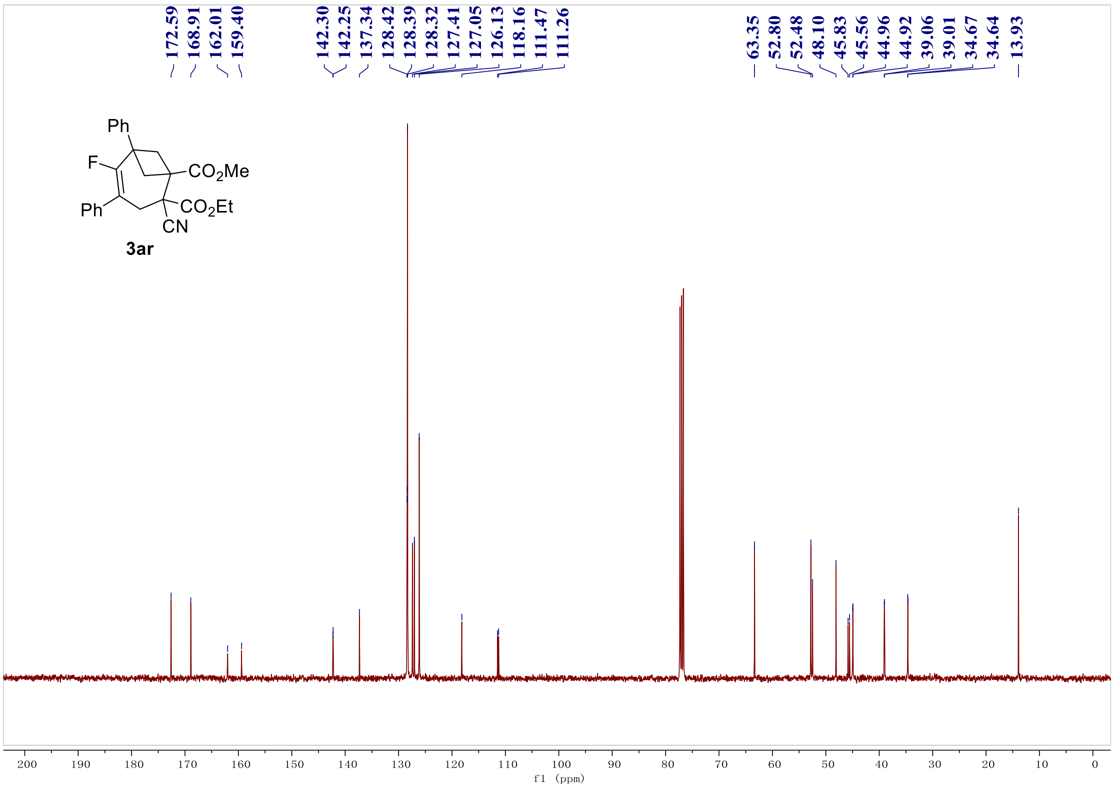
172.59
168.91
162.01
159.40
142.30
142.25
137.34
128.42
128.39
128.32
127.41
127.05
126.13
118.16
111.47
111.26
63.35
52.80
52.48
48.10
45.83
45.56
44.96
44.92
39.06
39.01
34.67
34.64
13.93
Ph
F
CO2Me
Ph
CO2Et
CN
3ar
200 190 180 170 160 150 140 130 120 110 100 90 80 70 60 50 40 30 20 10 0
f1 (ppm)

^{1}H NMR (400MHz, $CDCl_3$), ^{19}F NMR (376 MHz, $CDCl_3$) and ^{13}C NMR (100 MHz, $CDCl_3$) spectra of product **4aa**

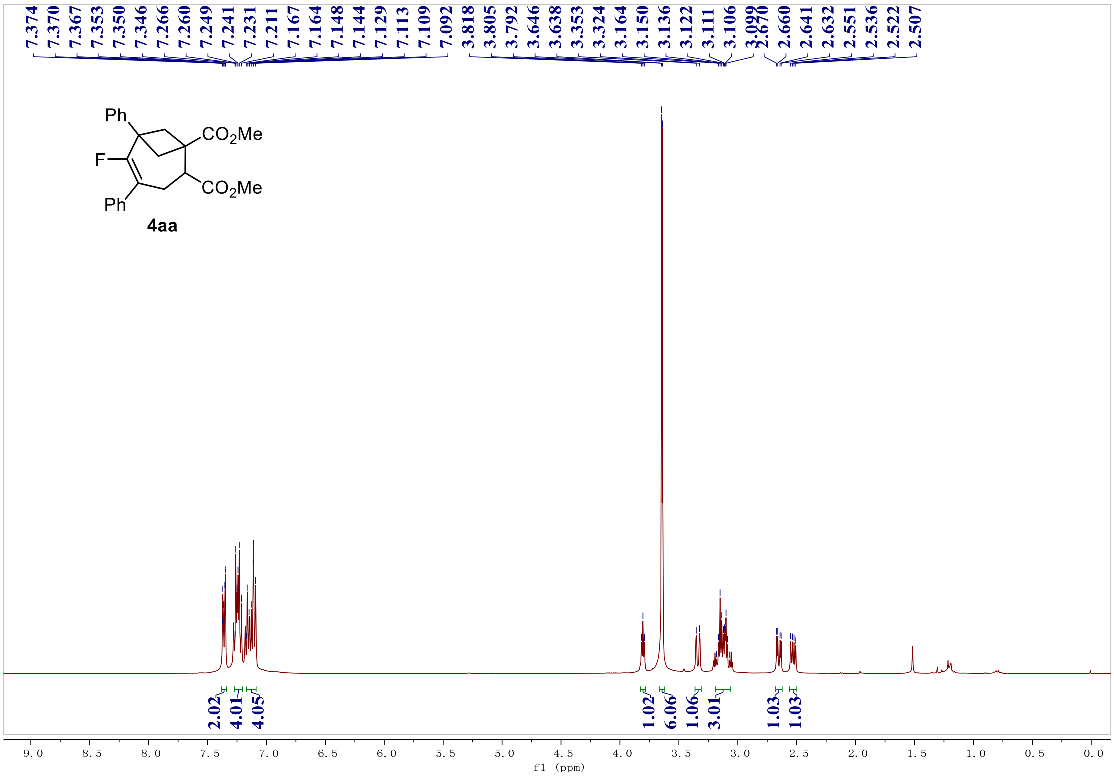

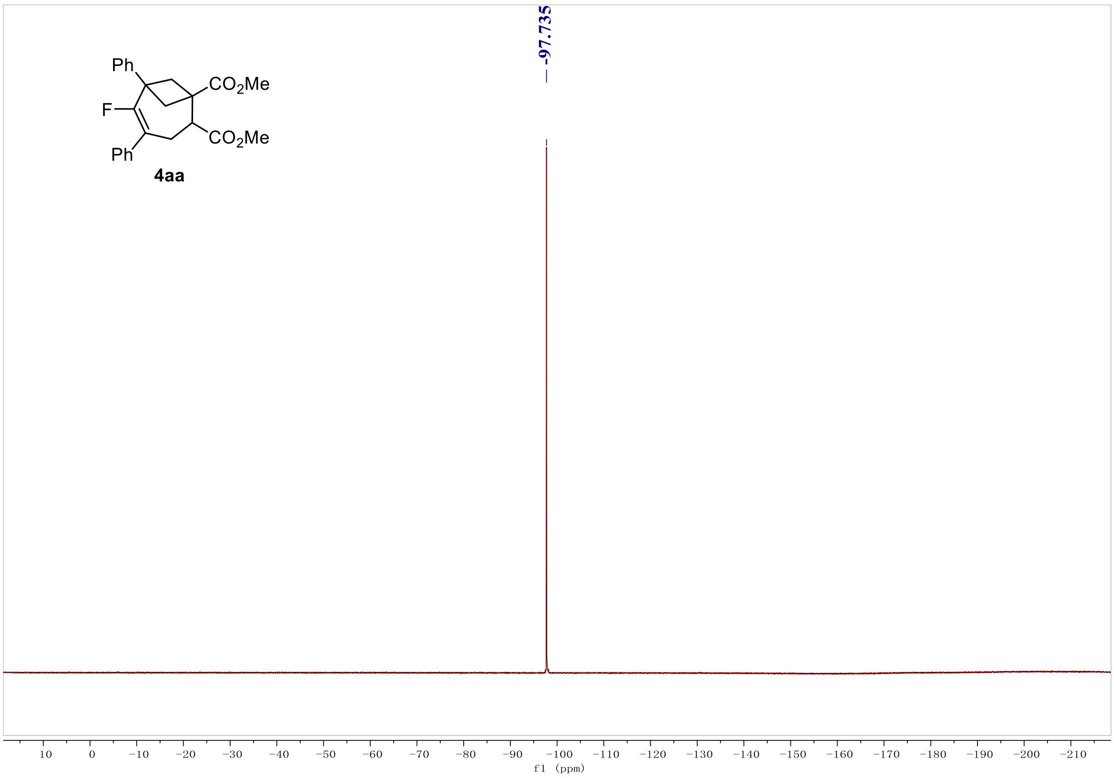

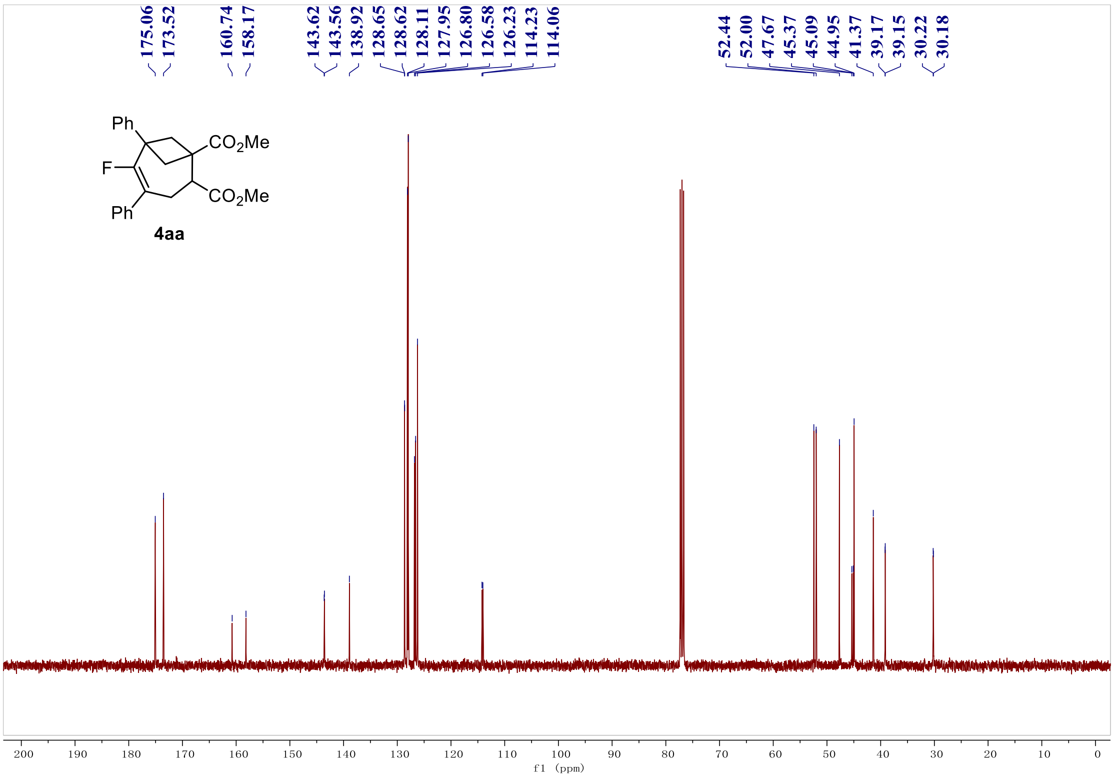

^{1}H NMR (400MHz, $CDCl_3$), ^{19}F NMR (376 MHz, $CDCl_3$) and ^{13}C NMR (100 MHz, $CDCl_3$) spectra of product **5aa**

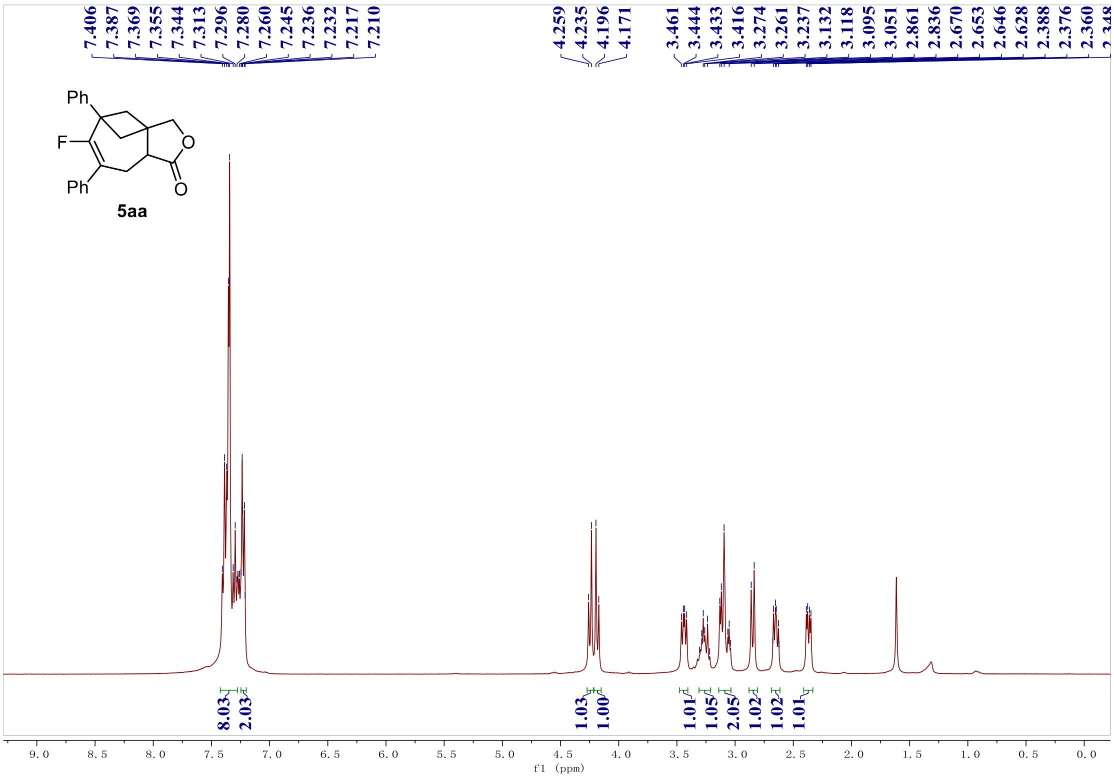

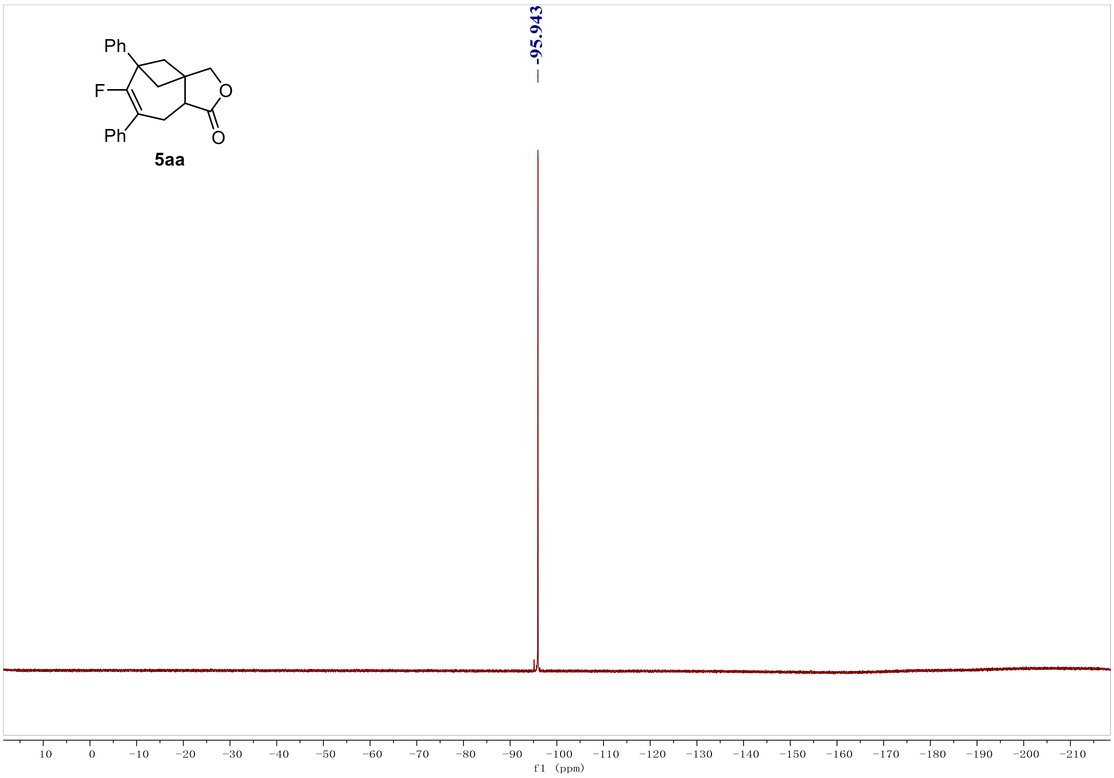

-95.943
Ph
F
Ph
O
O
5aa
f1 (ppm)

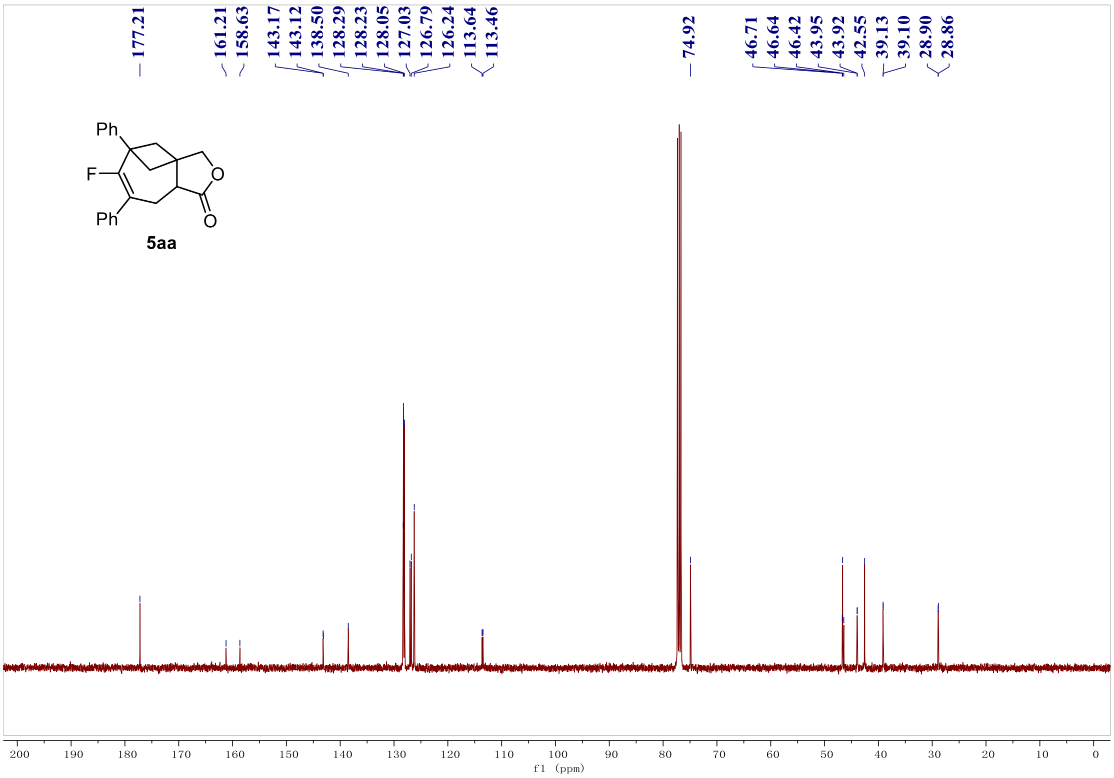

177.21
161.21
158.63
143.17
143.12
138.50
128.29
128.23
128.05
127.03
126.79
126.24
113.64
113.46
74.92
46.71
46.64
46.42
43.95
43.92
42.55
39.13
39.10
28.90
28.86
Ph
F
Ph
O
O
5aa
f1 (ppm)

^{1}H NMR (400MHz, $CDCl_3$), ^{19}F NMR (376 MHz, $CDCl_3$) and ^{13}C NMR (100 MHz, $CDCl_3$) spectra of product **6ka**

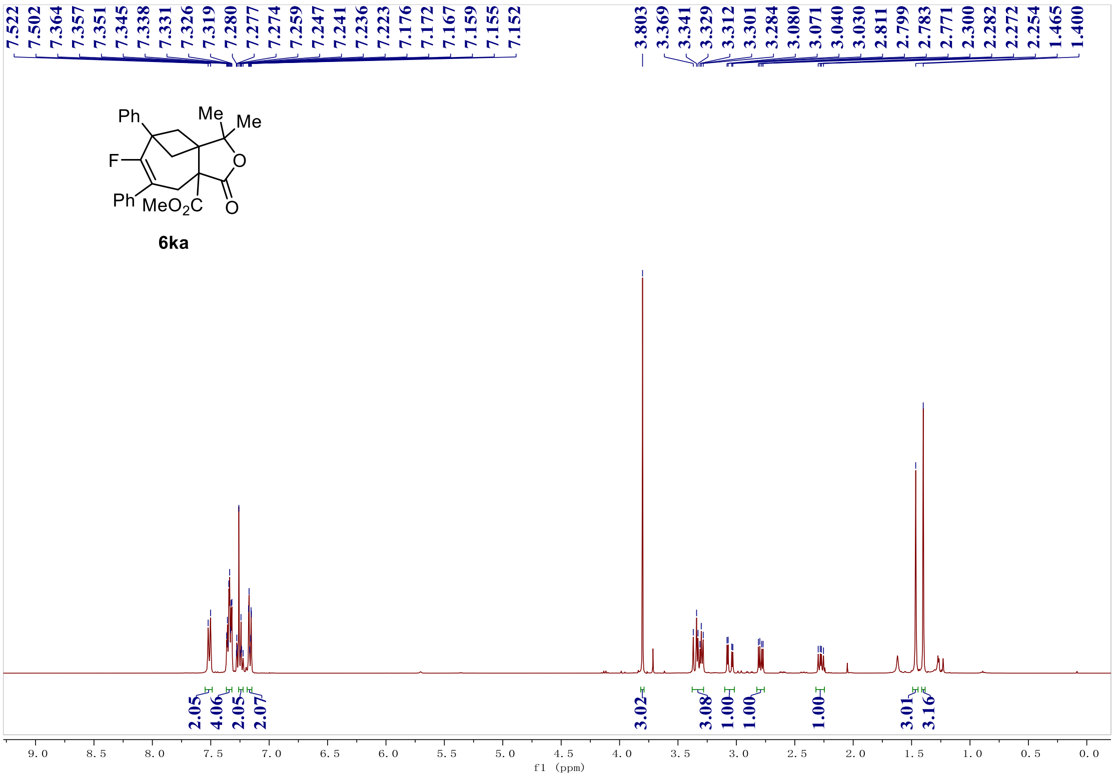

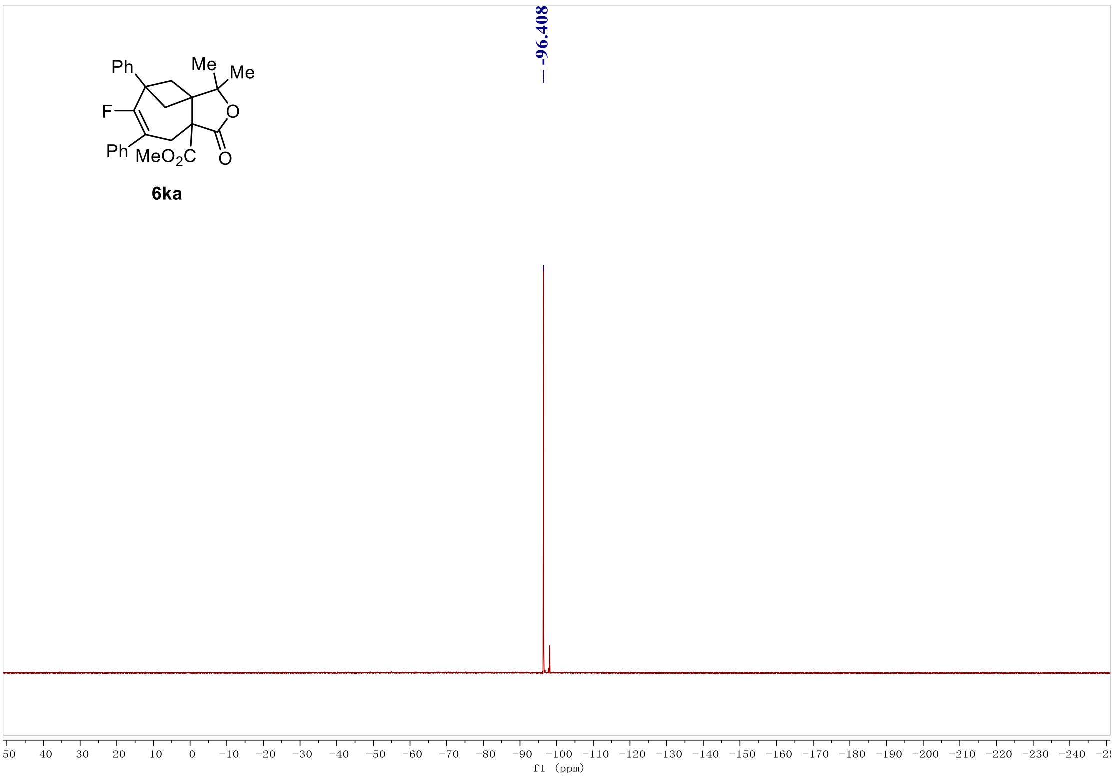

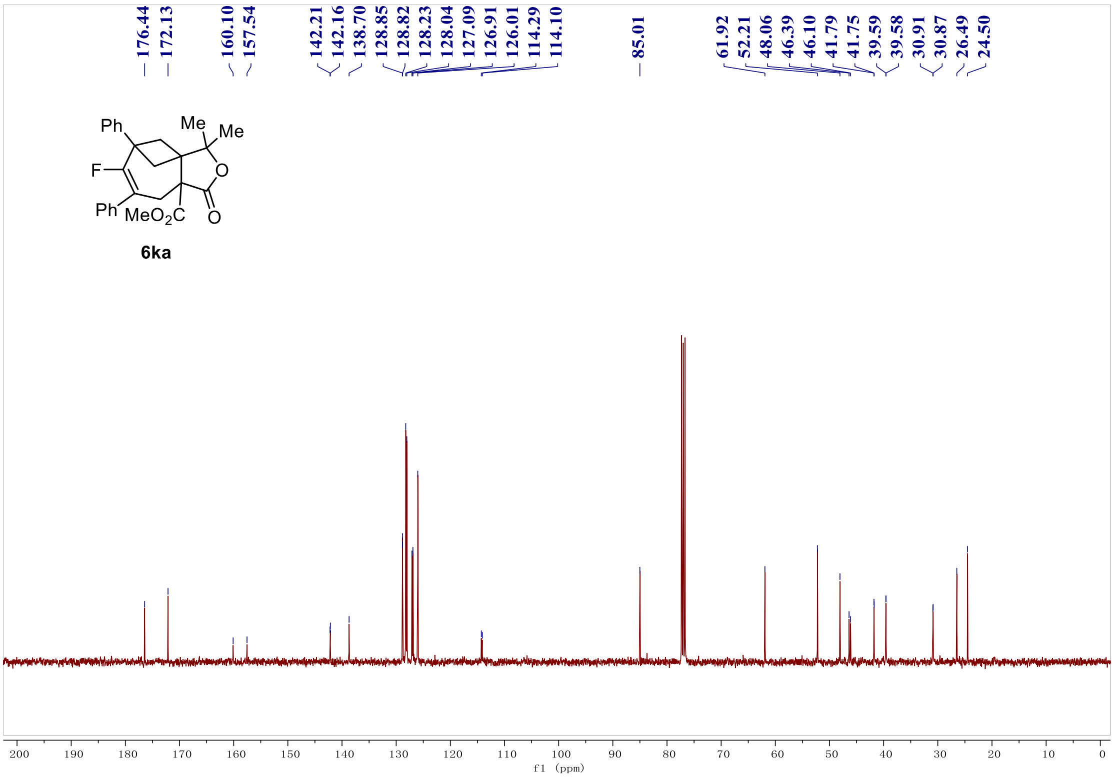

^{1}H NMR (400MHz, $CDCl_3$), ^{19}F NMR (376 MHz, $CDCl_3$) and ^{13}C NMR (100 MHz, $CDCl_3$) spectra of product **6ka'**

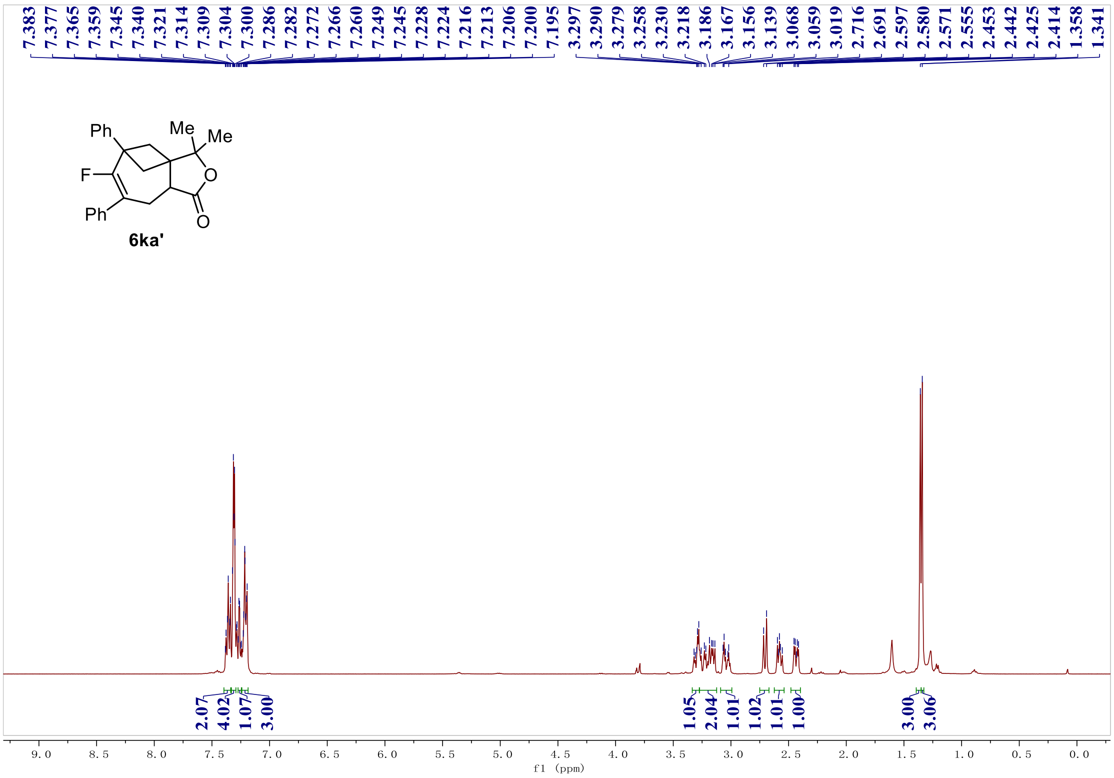

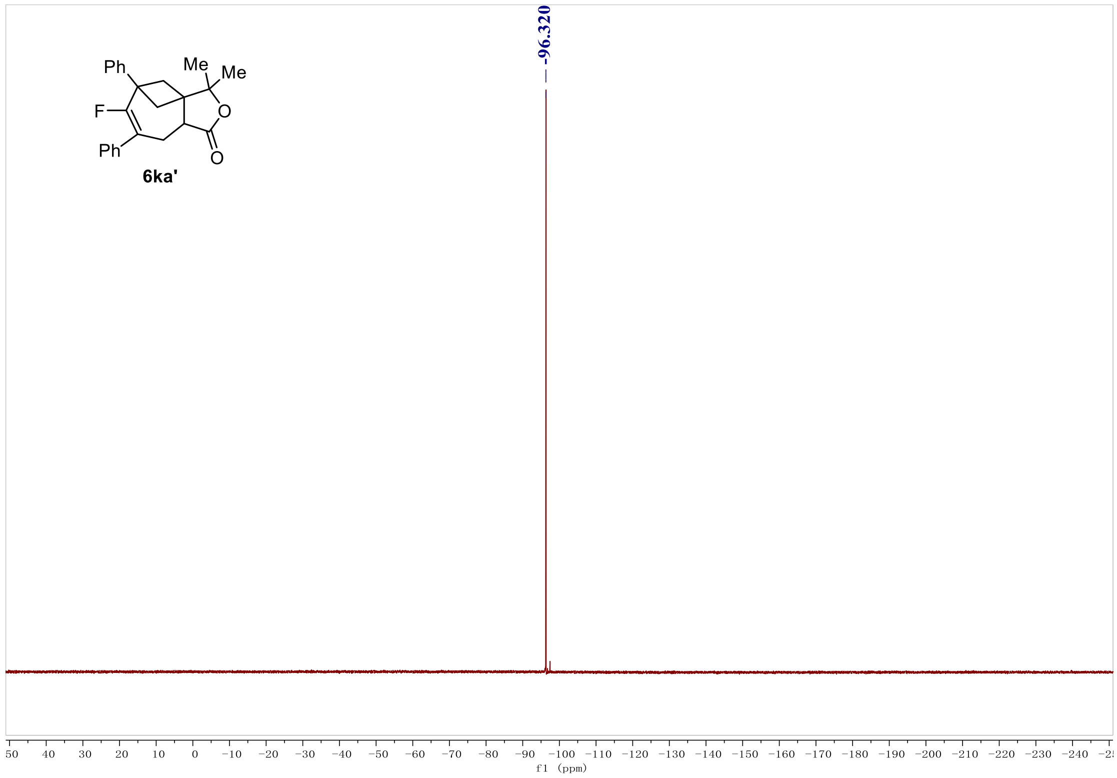
-96.320
Ph
Me
Me
F
O
Ph
O
6ka'
f1 (ppm)

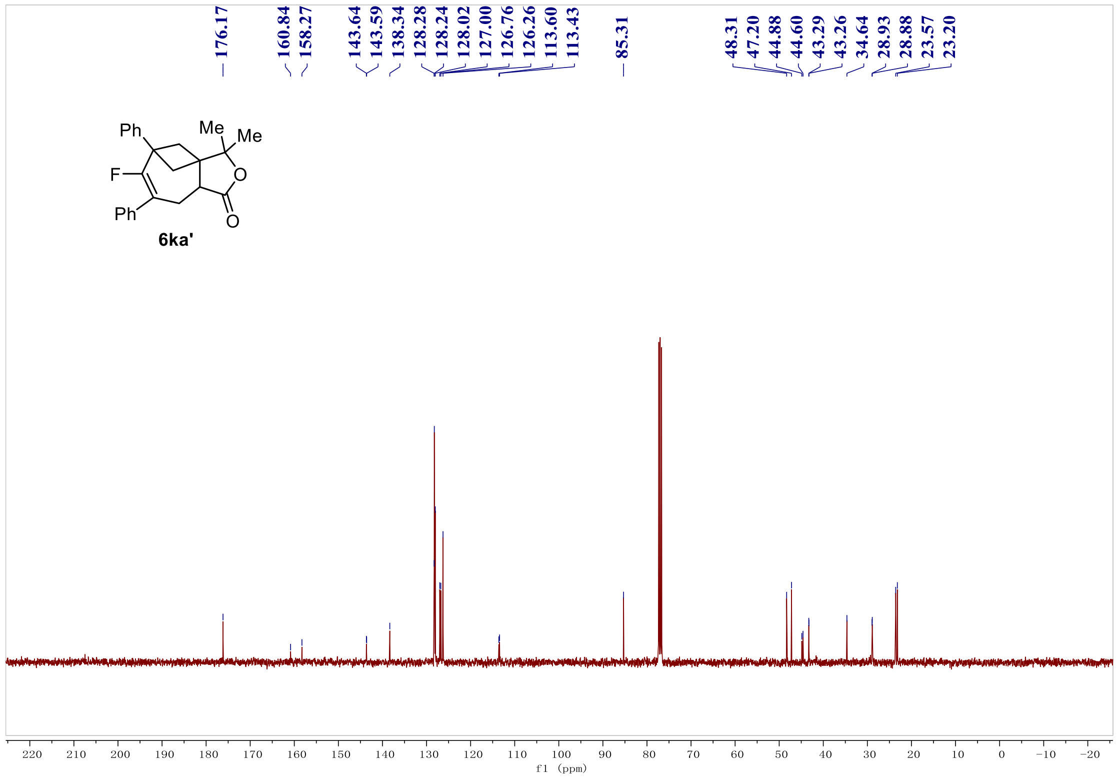
176.17
160.84
158.27
143.64
143.59
138.34
128.28
128.24
128.02
127.00
126.76
126.26
113.60
113.43
85.31
48.31
47.20
44.88
44.60
43.29
43.26
34.64
28.93
28.88
23.57
23.20
Ph
Me
Me
F
O
Ph
O
6ka'
f1 (ppm)

^{1}H NMR (400MHz, DMSO-d6), ^{19}F NMR (376 MHz, DMSO-d6) and ^{13}C NMR (100 MHz, DMSO-d6) spectra of product **7la**

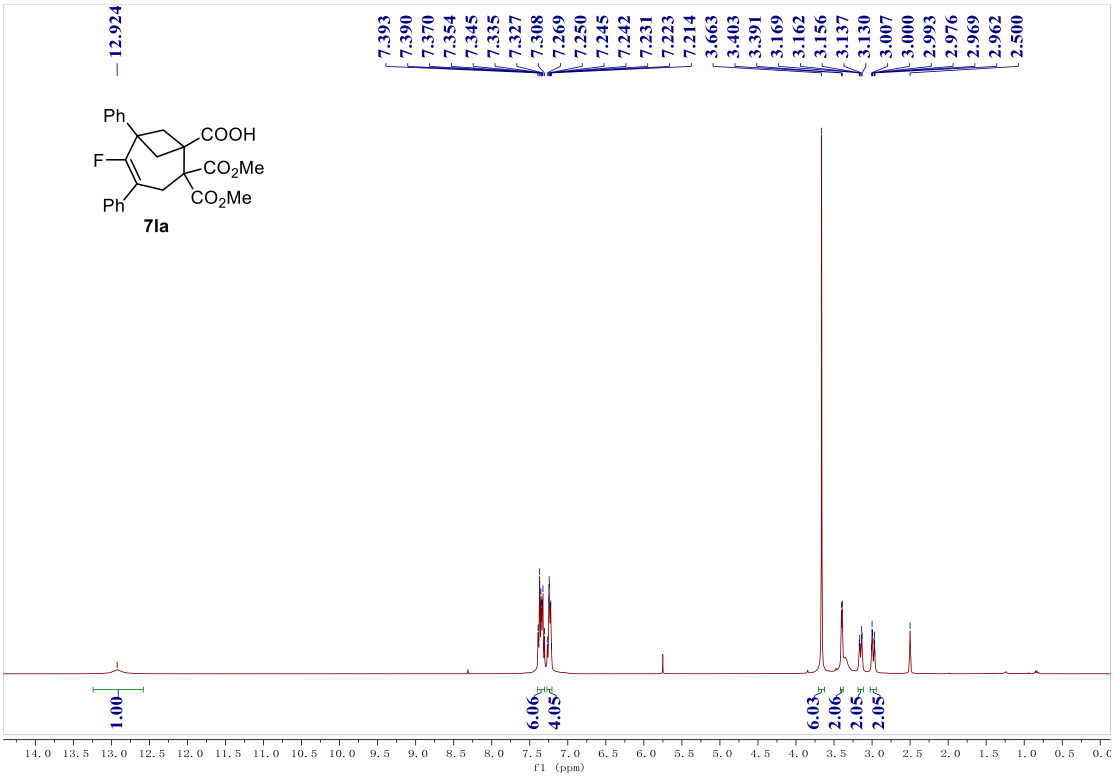

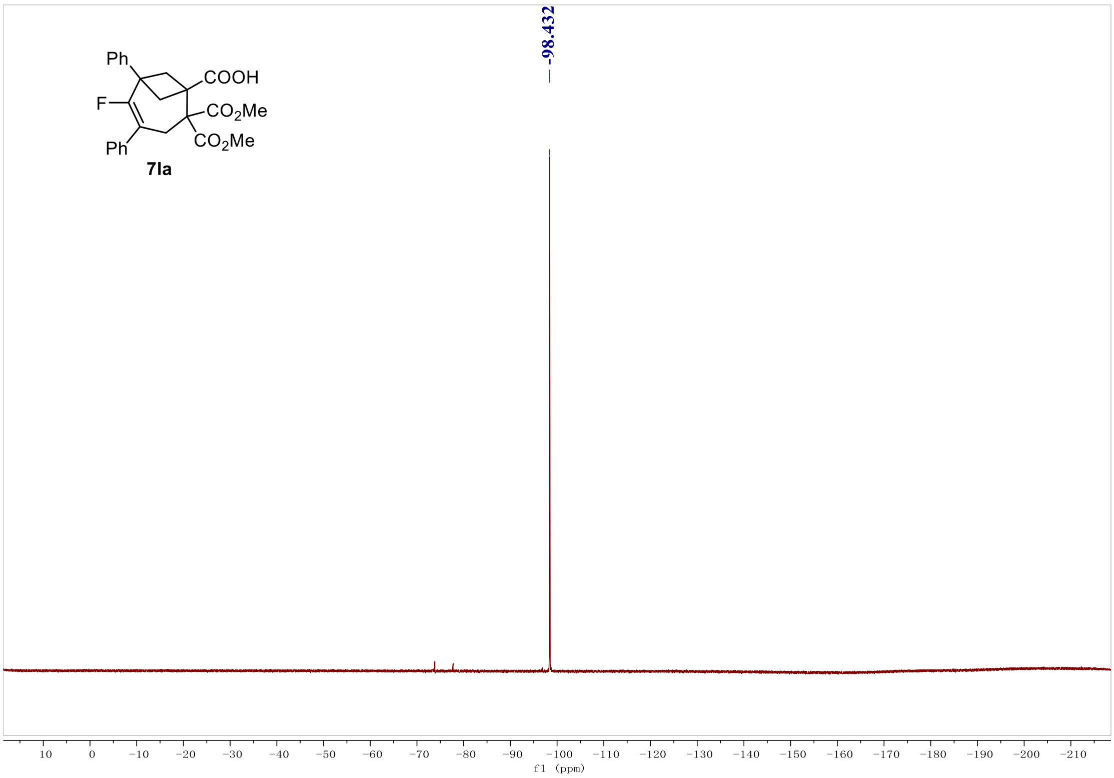

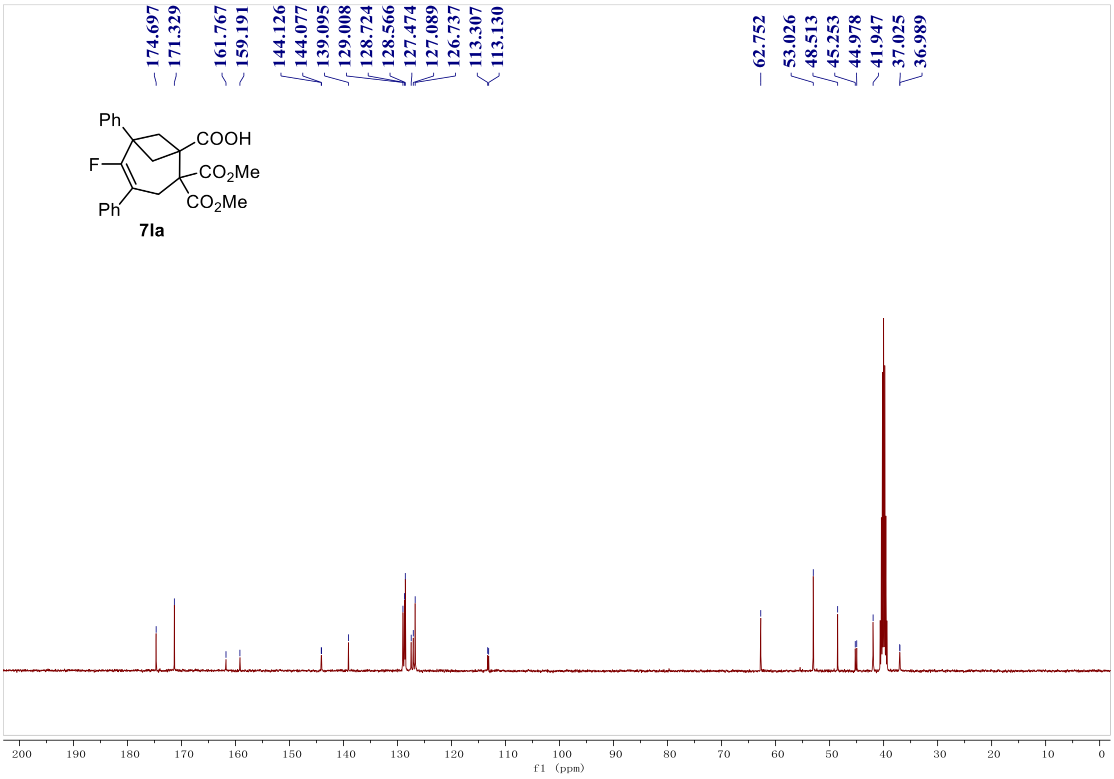

^{1}H NMR (400MHz, $CDCl_3$), ^{19}F NMR (376 MHz, $CDCl_3$) and ^{13}C NMR (100 MHz, $CDCl_3$) spectra of product **8la**

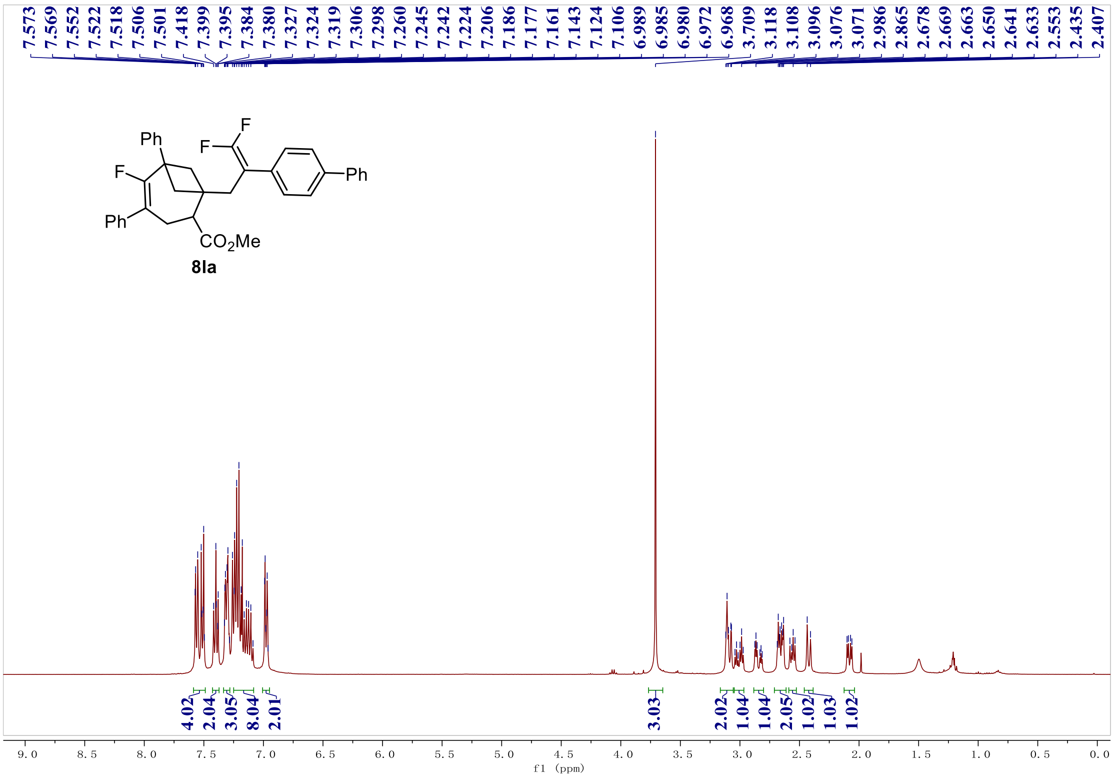

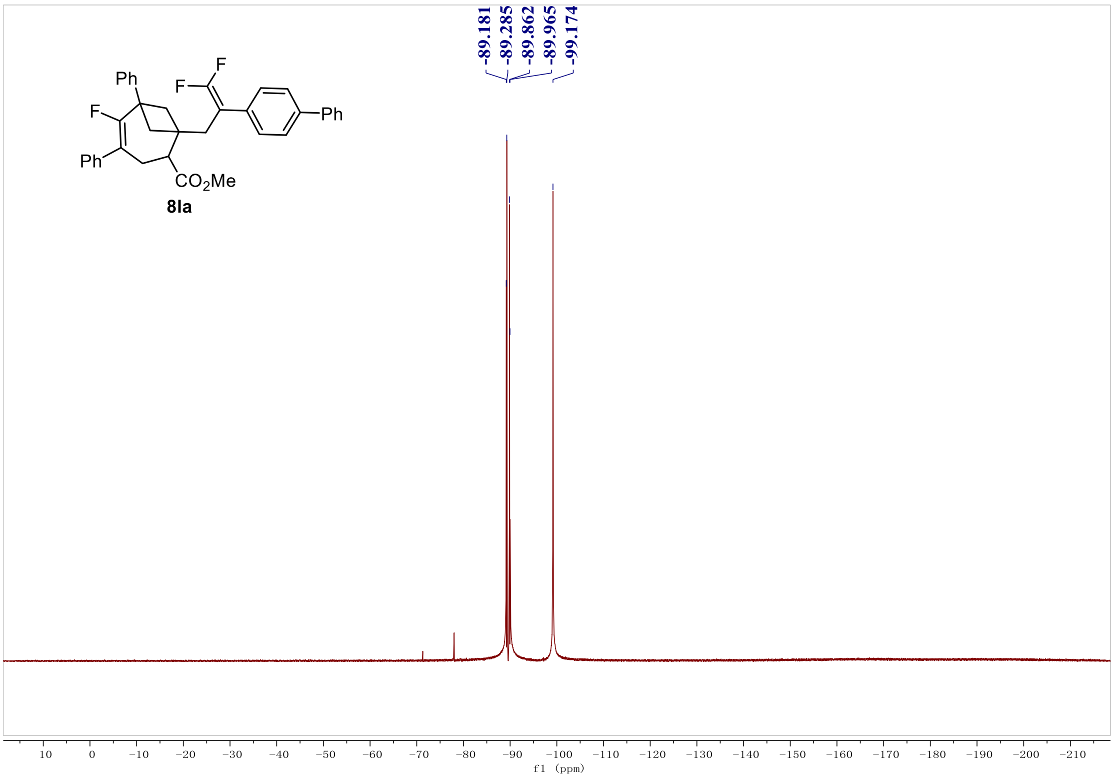
-89.181
-89.285
-89.862
-89.965
-99.174
Ph
F
F
F
Ph
Ph
CO2Me
8la
f1 (ppm)

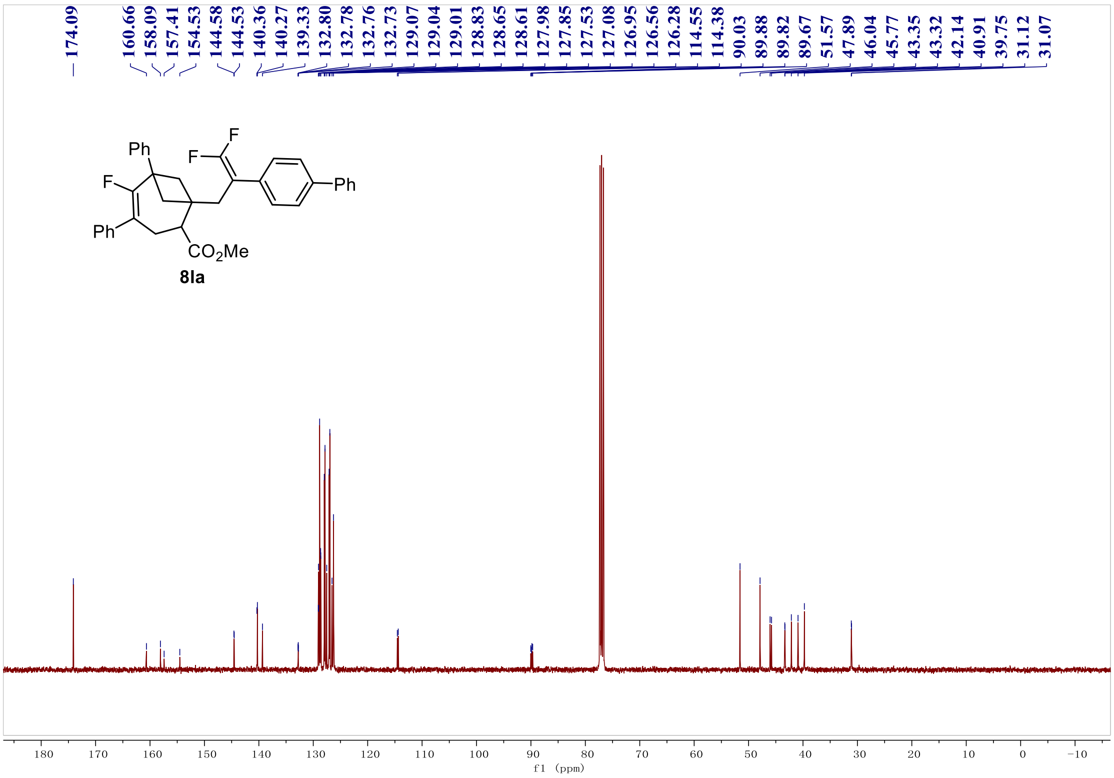
174.09
160.66
158.09
157.41
154.53
144.58
144.53
140.36
140.27
139.33
132.80
132.78
132.76
132.73
129.07
129.04
129.01
128.83
128.65
128.61
127.98
127.85
127.53
127.08
126.95
126.56
126.28
114.55
114.38
90.03
89.88
89.82
89.67
51.57
47.89
46.04
45.77
43.35
43.32
42.14
40.91
39.75
31.12
31.07
Ph
F
F
F
Ph
Ph
CO2Me
8la
f1 (ppm)

^{1}H NMR (400MHz, $CDCl_3$), ^{19}F NMR (376 MHz, $CDCl_3$) and ^{13}C NMR (100 MHz, $CDCl_3$) spectra of product **9la**

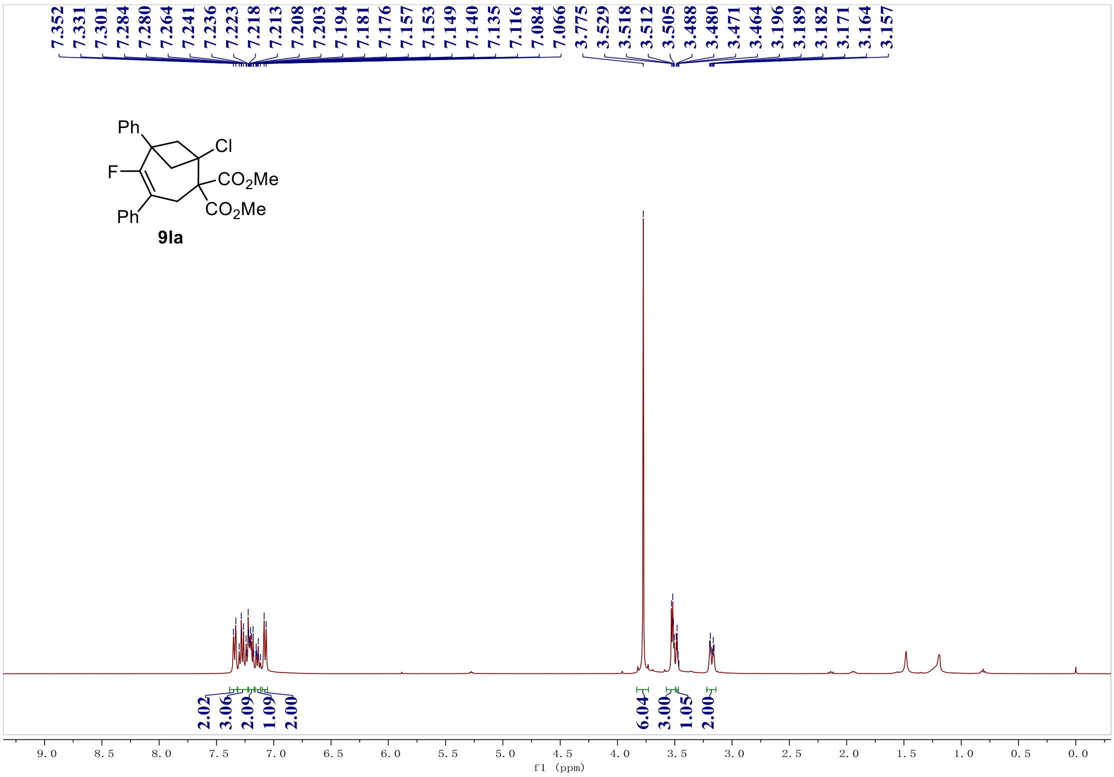

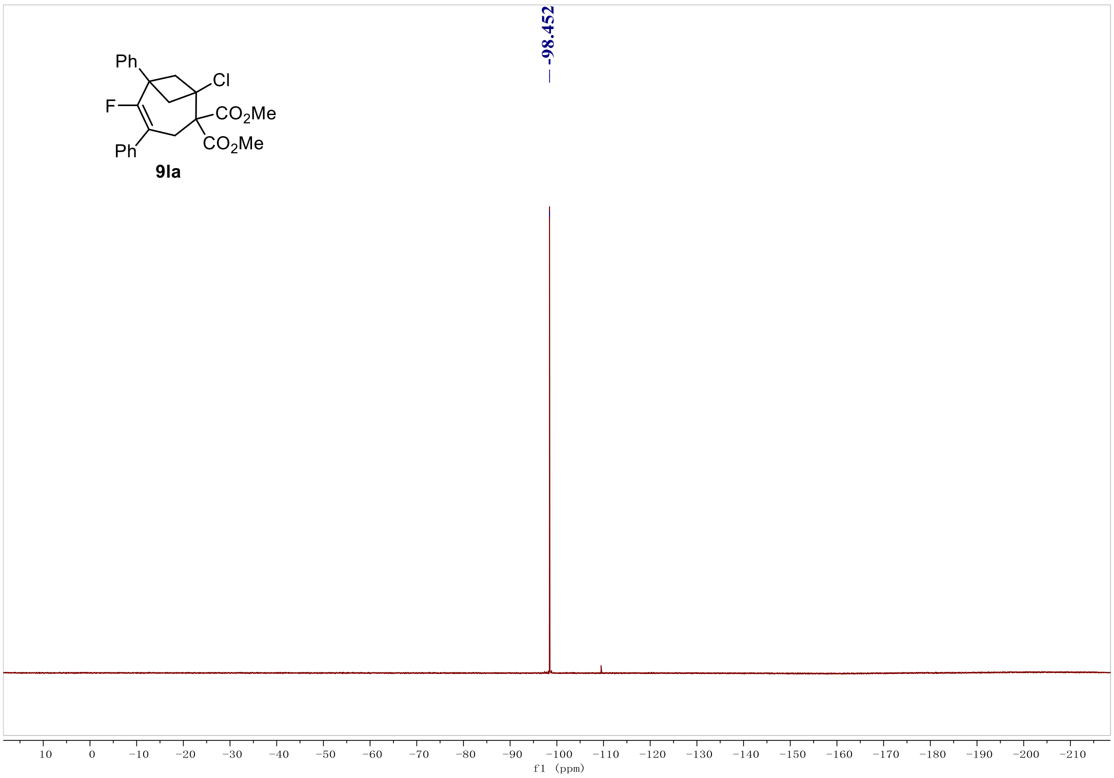

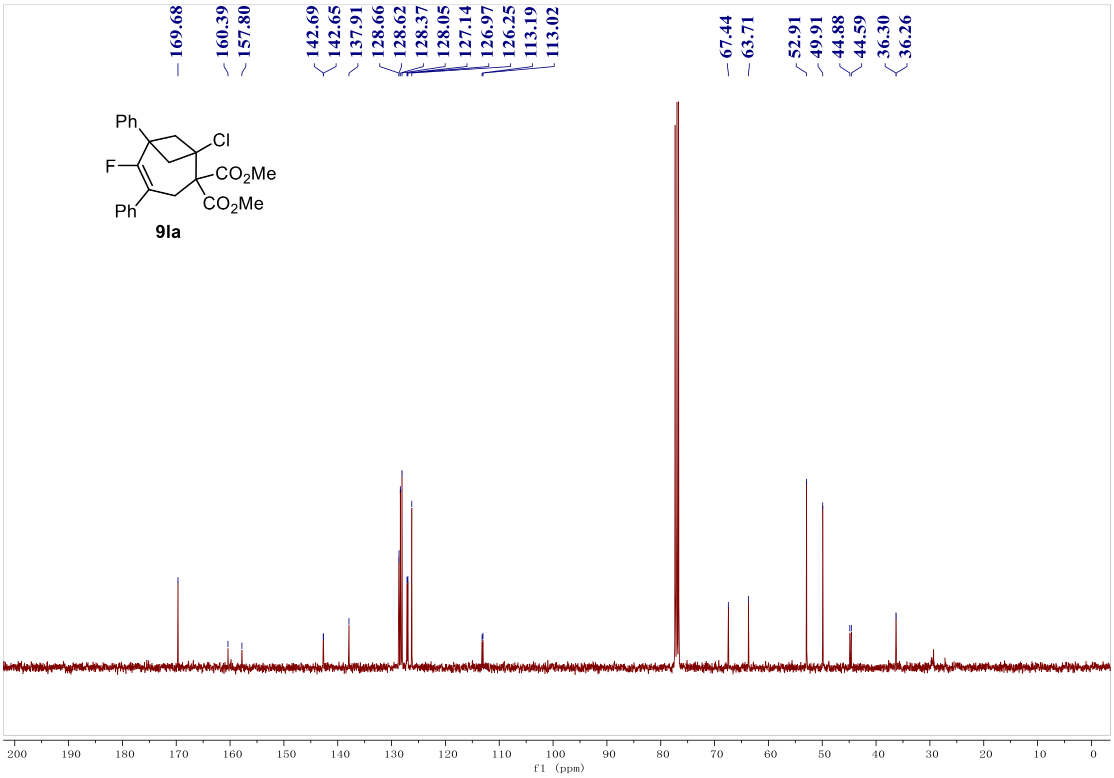

^{1}H NMR (400MHz, $CDCl_3$), ^{19}F NMR (376 MHz, $CDCl_3$) and ^{13}C NMR (100 MHz, $CDCl_3$) spectra of product **10la**

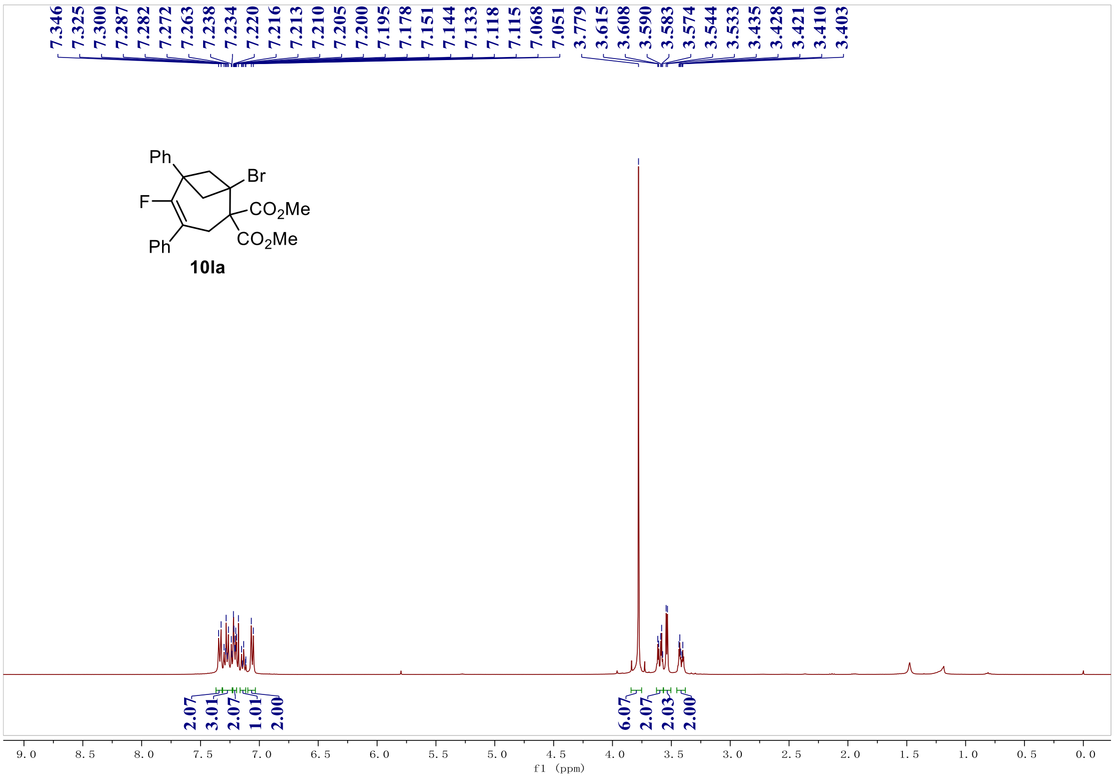

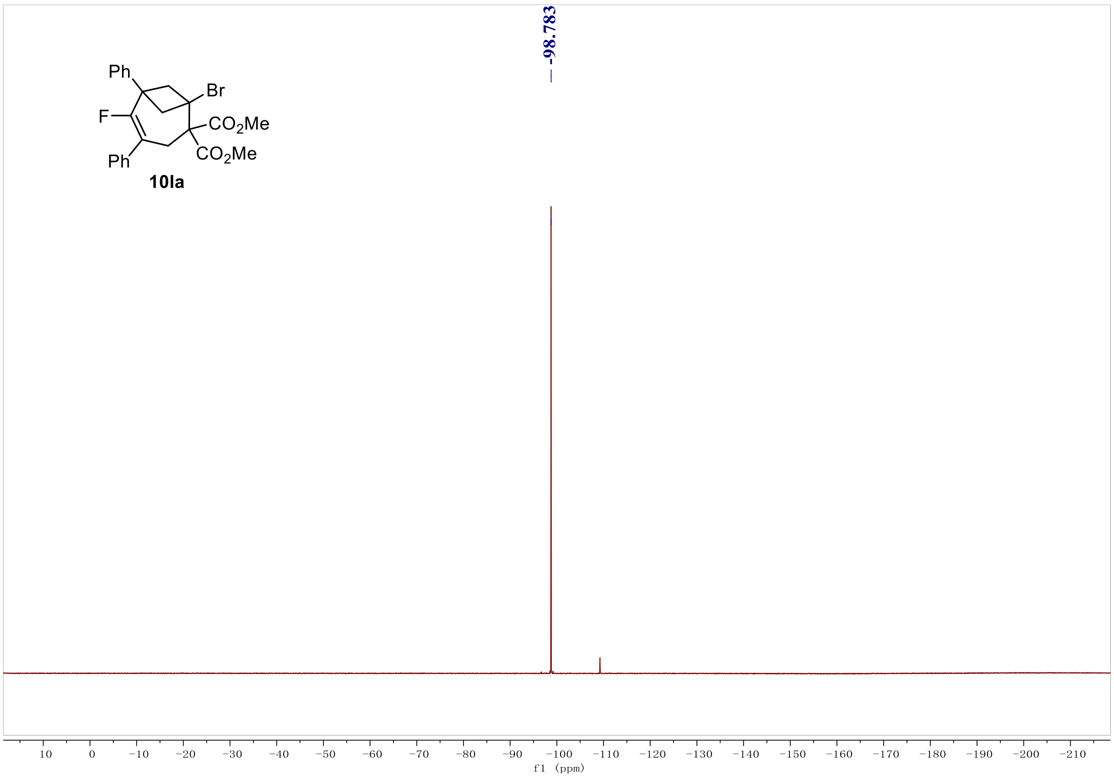
-98.783
Ph
Br
F
CO2Me
CO2Me
Ph
10la
f1 (ppm)

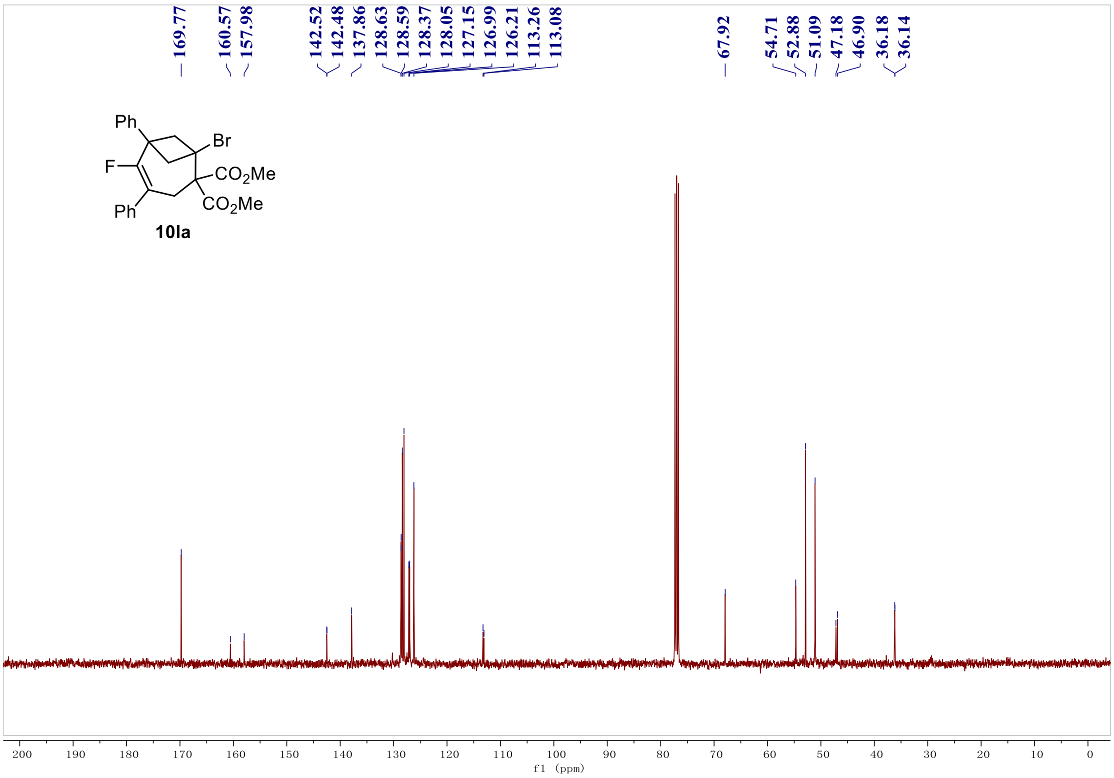
169.77
160.57
157.98
142.52
142.48
137.86
128.63
128.59
128.37
128.05
127.15
126.99
126.21
113.26
113.08
67.92
54.71
52.88
51.09
47.18
46.90
36.18
36.14
Ph
Br
F
CO2Me
CO2Me
Ph
10la
f1 (ppm)
